# Impact of the structures of macrocyclic Michael acceptors on covalent proteasome inhibition†
†Electronic supplementary information (ESI) available. See DOI: 10.1039/c7sc02941a



**DOI:** 10.1039/c7sc02941a

**Published:** 2017-08-11

**Authors:** S. Kitahata, F. Yakushiji, S. Ichikawa

**Affiliations:** a Faculty of Pharmaceutical Sciences , Hokkaido University , Kita-12, Nishi-6, Kita-ku , Sapporo 060-0812 , Japan . Email: ichikawa@pharm.hokudai.ac.jp; b Center for Research and Education on Drug Discovery , Hokkaido University , Kita-12, Nishi-6, Kita-ku , Sapporo 060-0812 , Japan

## Abstract

Molecules that have a reactive functional group within a macrocycle represent a class of covalent inhibitor. The relationship between reactivity and affinity for the target is cooperative and complicated. An understanding and characterization of this class of inhibitor are vital for the development of covalent inhibitors as drug candidates. Herein, we describe a systematic analysis of structure–activity relationships using a series of syringolin analogues, which are irreversible covalent inhibitors of proteasomes. We investigate the detailed mechanistic effects of the macrocycles on affinity and reaction rate.

## Abstract 2

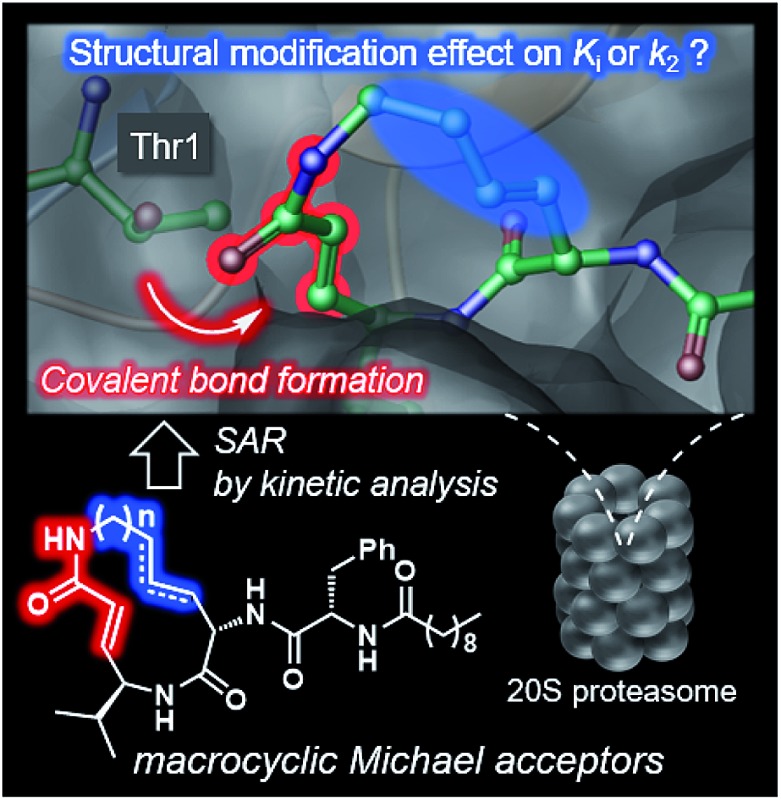
A systematic analysis of the structure–activity relationship of a series of syringolin analogues, which are irreversible covalent inhibitors of proteasomes.

## Introduction

Covalent inhibitors are compounds that form a covalent link with a functional group of the target enzyme or protein.^1^ Because the reactive functional groups of the inhibitors may react with different enzymes and proteins, resulting in potentially dangerous off-target effects, they have rarely been considered as starting points in molecularly targeted drug discovery programs.^2^ However, the field has seen recent success with the development of targeted covalent drugs such as afatinib, which was approved for metastatic non-small cell lung cancer. This has led to a resurgence in covalent inhibitors.^3^ The design of covalent inhibitors is different from that of non-covalent inhibitors. Covalent inhibitors form covalent complexes with their targets. The process involves several steps, and a generic mechanism is shown in eqn (1).^4^
1
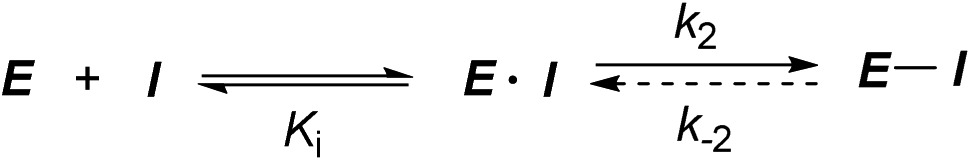
In the first step, a covalent inhibitor associates with its target protein *via* non-covalent interactions to form an inhibitor–protein complex (E·I). This step is controlled by the binding affinity between the compound and target, *K*_i_. A chemical reaction then takes place between the inhibitor and protein to form a covalent complex (E–I) and there is a conformational change in the complex. This step is defined only by the reaction rate *k*_2_ if the reaction is irreversible. Structure-based drug design using the coordinates of the complex structure of a ligand and protein is a valuable approach, which allows us to rationally design inhibitors.^5^ However, this method is not always useful for designing covalent inhibitors because an X-ray crystal structure of covalent inhibitor/protein complexes is the reaction product, E–I, and does not always reflect the association state or the transition state from E·I to E–I. Therefore, detailed analysis of each step is necessary for the rational design of covalent inhibitors. Analyzing *K*_i_ and *k*_2_ separately provides direct and quantitative information about whether the observed changes in inhibitory activity can be attributed to changes in *K*_i_, changes in *k*_2_, or changes in both the binding and reaction steps.^6^

**Fig. 1:**
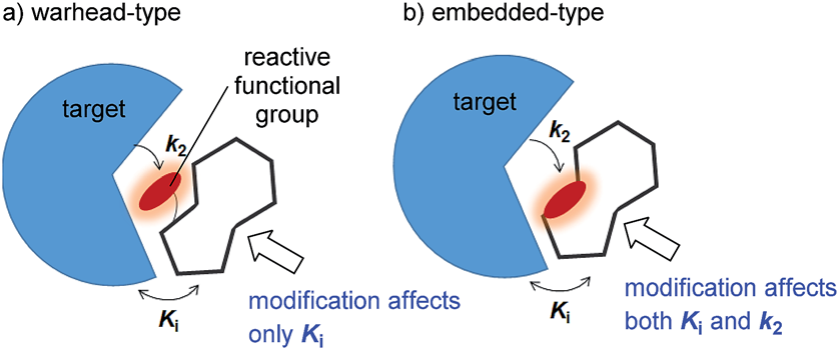
Two types of covalent inhibitor. (a) Warhead-type covalent inhibitor, and (b) embedded-type inhibitor.

Covalent inhibitors are classified into two chemotypes. One chemotype has a reactive functional group that acts as a warhead, to which a core skeleton is attached (Fig. 1a). In this case, the warhead determines the reactivity (*k*_2_), and the core determines the affinity to the target (*K*_i_). Generally, these two parameters can be independently understood, and the rational design of this type of irreversible inhibitor is relatively simple.^7^ In the design process, a reversible inhibitor is first identified for which the binding mode to the target is known. Then, structural information is used to design irreversible inhibitors with electrophilic warheads. The warhead is positioned to react specifically with the nucleophilic amino acid in the target.^8^ The other chemotype is an embedded-type covalent inhibitor, which is a class of molecule containing a reactive functional group within a macrocycle. This type of inhibitor is frequently found in natural products (Fig. 1b).^9^ A change in the size or conformation of the macrocycle is expected to affect not only the reactivity of the embedded functional group (*k*_2_) but also its affinity to the target molecule (*K*_i_). Thus, the relationship between reactivity and affinity is complex and cooperative. Although most of the covalent inhibitors that have been studied are warhead-type molecules, our understanding of the mechanistic details of embedded-type covalent inhibitors remains limited. One of the reasons for a lack of information is the absence of a set of molecules containing a reactive functional group within the macrocycle that is suitable for systematic analysis. It is vital for researchers to understand and characterize the affinity and reactivity of embedded-type covalent inhibitors so that covalent inhibitors can be developed as drug candidates.

**Fig. 2:**
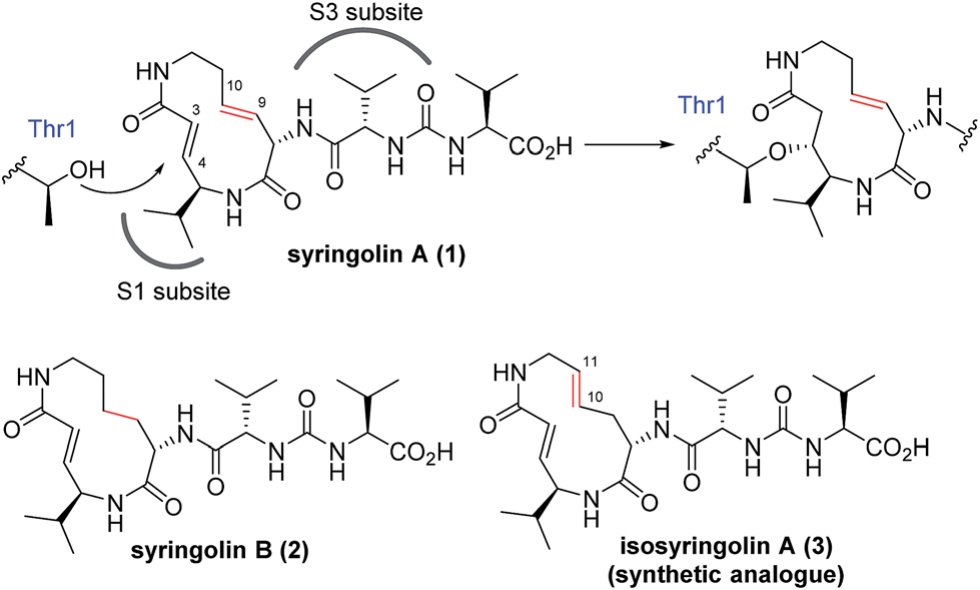
Structures of syringolins and an analogue, and their mode of irreversible inhibition.

The naturally occurring compounds syringolin A and B (**1** and **2**, Fig. 2) are 12-membered macrolactams. They irreversibly inhibit proteasomes by an oxa-Michael addition of the hydroxyl group of the N-terminal threonine (Thr) residue on the β5 subunit (chymotrypsin-like) to the α,β-unsaturated carboxamide moiety embedded in the macrolactam.^10^ The inhibition of proteasomes results in the accumulation of unnecessary proteins and ultimately causes cell death.^11,12^ Syringolin A (**1**) has stronger proteasome–inhibitory activity than **2**, which lacks the alkene at the dehydrolysine residue. Isosyringolin A (**3**) is a synthetic analogue, in which the alkene at the dehydrolysine of **1** is transposed, and its apparent β5 subunit inhibitory activity (*K*′_i_) is intermediate between **1** and **2**. The subtle structural differences in the 12-membered macrocycles affect the apparent inhibitory activity,^13^ and syringolins and their analogues can serve as model embedded-type covalent inhibitors, as shown in Fig. 1b. Herein, we describe the synthesis of a series of syringolin analogues, and we perform a systematic analysis of the structure–activity relationship (SAR) to investigate the effect of the structures of macrocyclic Michael acceptors on covalent inhibition.

## Results and discussion

**Fig. 3:**
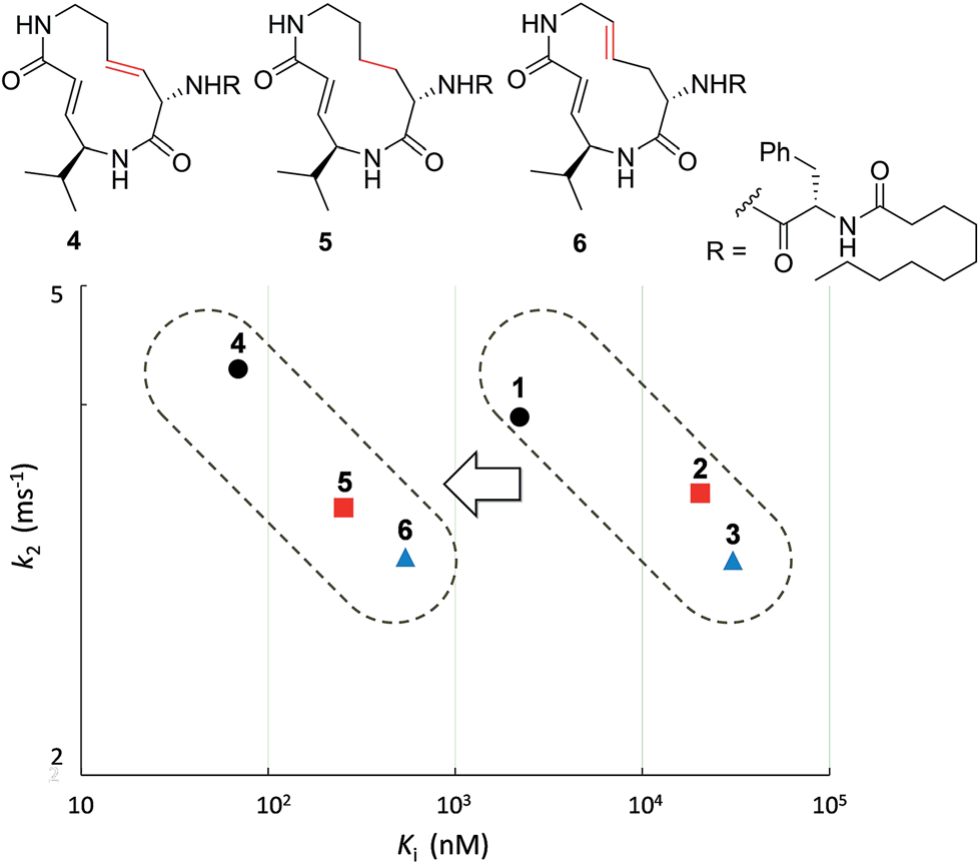
Plot of the kinetic parameters of **1–6**.

First, the effect of modifying the macrocycle was investigated for **1–3** by analyzing the inhibitory activity of the β5 subunit of the human constitutive 20S proteasome. To determine *K*_i_ and *k*_2_ for each compound, we assessed the observed rate constant (*k*_obs_) for inhibition at each concentration, and calculated values for *K*_i_ and *k*_2_ using the following equation, *k*_obs_ = *k*_2_[I]/(*K*_i_ + [I]).^14^ The *k*_2_/*K*_i_ ratio represents the second-order rate constant for the reaction of the inhibitor with the target (*k*_assoc_), and it indicates the overall inhibitory potency of the inhibitor. These values are summarized in Fig. 3 and Table 1. Syringolin A (**1**) was the most potent inhibitor based on its parameter values with *K*′_i_, *K*_i_, *k*_2_, and *k*_assoc_ equal to 170 nM, 2210 nM, 3.91 × 10^–3^ s^–1^, and 1769 s^–1^ M^–1^, respectively. The potency decreased in order from **1** to **3**, and, as expected, the position and presence of the second alkene affected both *K*_i_ and *k*_2_ even though compounds **1–3** all had a ring size of 12. The analogues **4–6**, in which the urea dipeptide side chain was replaced with *N*-decanoyl-l-phenylalanine to provide more potent β5 inhibitory activity than parents **1–3**, exhibited higher *K*_i_ values with no effect on the *k*_2_ values. These properties are in accordance with our previous data, which showed that the benzyl group outside the macrocycle is recognized by the S3 subsite of the β5 subunit, with a hydrophobic interaction affecting only *K*_i_.^15^

**Table 1:** Kinetic parameters of syringolin analogues

	Ring size	*K*′_i_ (nM)	*K*_i_ (nM)	*k*_2_ (ms^–1^)	*k*_assoc_ (s^–1^ M^–1^)
Syringolin A (**1**)	12	170	2210	3.91	1769
Syringolin B (**2**)		3700	30400	2.99	98.4
Isosyringolin A (**3**)		590	20400	3.39	166
**4**		1.6	68.9	4.28	62075
**5**		46	541	3.01	5558
**6**		21.8	252	3.30	13075
**7**		>1000	—	—	
**8**	11	20.6	432	5.51	12761
**9**		48.2	547	3.65	6673
**10**	13	79.2	527	1.34	2546
**11**		320	1227	0.875	713
**12**		402	9092	2.03	224
**13**	—	>1000	—	—	—

**Fig. 4:**
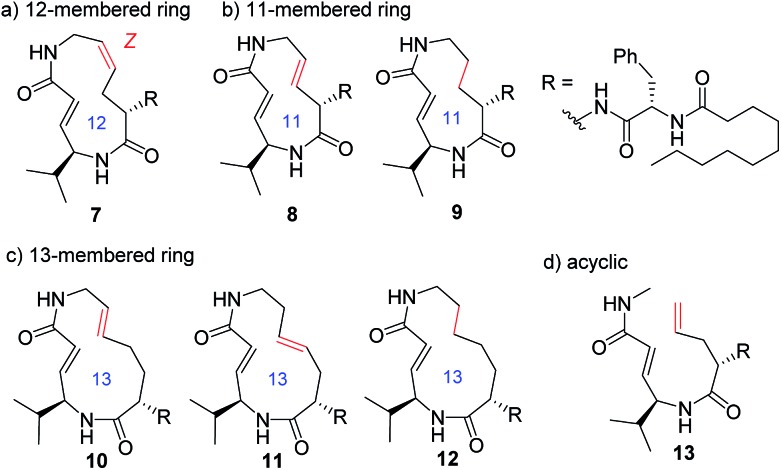
Structures of syringolin analogues.

In addition to compounds **1–6**, a set of analogues consisting of macrocycles containing α,β-unsaturated carboxamide functionality with ring sizes of 11–13 was further designed by removing or diversifying the position of the remaining alkene, as shown in Fig. 4. In this way, a systematic SAR study was performed. The acyclic analogue was also used to determine the impact of the macrocyclic structure on the inhibitory activity. The synthesis of these analogues is described in the ESI (Schemes S1–S8†).

**Fig. 5:**
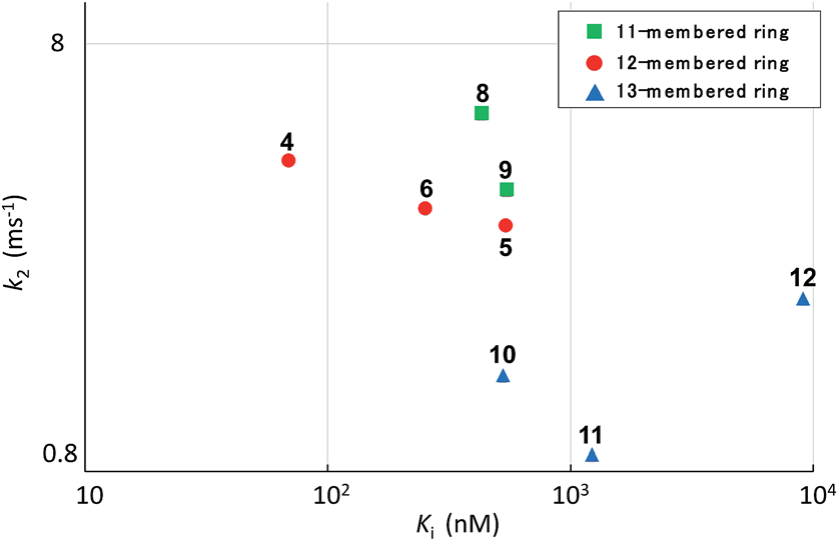
Plot of the kinetic parameters of syringolin analogues.

Systematic analysis of the set of analogues consisting of macrocycles with different ring sizes and varying the presence and/or position of the second alkene shed light on the impact of the chemical structure of the macrocycle on the affinity and reactivity (Table 1). The 12-membered ring analogue **7**, which has a *Z*-alkene, as well as the acyclic analogue **13**, showed no inhibitory activity. For the analogues exhibiting inhibitory activity, not only *K*_i_ but also *k*_2_ were highly varied, as shown in Fig. 5, and they showed variation in their properties depending on the chemical structure of the macrocycle. First, *k*_2_ is governed by the ring size of the macrocycle. Specifically, the smaller the ring, the larger the *k*_2_ value. Analogue **8**, which has an 11-membered macrocycle with an *E*-alkene, is the most reactive analogue. It has a *k*_2_ value of 5.51 ms^–1^, which is 1.29-fold greater than that of the 12-membered **4**. In the case of 11- and 12-membered analogues, introducing the second alkene increased the reactivity. The 13-membered analogues **10–12** show a decrease in *k*_2_, with values ranging from 0.875 to 2.03 ms^–1^. Presumably, the smaller and strained macrocycle is more reactive to the Thr residue because the reaction relieves the ring strain upon oxa-Michael addition, which provides a driving force to accelerate the reaction rate with the proteasome.

**Fig. 6:**
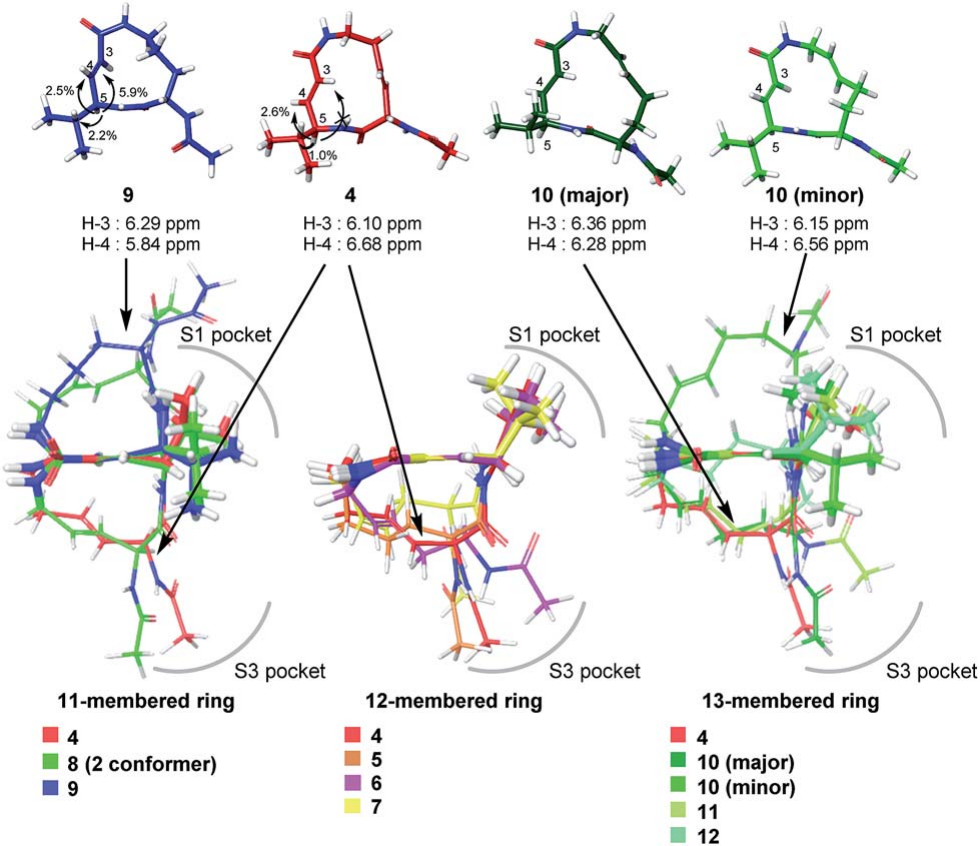
Structural comparison of conformations.

The modifications made in compound **8** decreased the affinity for the target; the *K*_i_ value was 432 nM, representing a 6.3-fold decrease relative to **4**. The decrease in affinity occurs despite the increased reactivity (*k*_2_ = 5.51 ms^–1^). The 12-membered analogues tended to have better properties, and the syringolin A-type analogue **4** had the smallest *K*_i_ value: 68.9 nM. Due to its high reactivity and affinity, **4** exhibited the most potent inhibitory activity among analogues **1–13**, with a *k*_assoc_ value of 62 075 s^–1^ M^–1^. The distributions of these parameters are informative. The *K*′_i_ values of **8** and **6** are similar, but the distributions of their *K*_i_ and *k*_2_ values are different: *K*_i_ contributes for **6**, and *k*_2_ contributes for **8**. The difference in contributions was observed for analogues with the same ring size. In particular, of the 13-membered analogues, compound **11**, which has an *E*-alkene at the 10-position, and compound **12**, which lacked a second alkene, exhibited similar *K*′_i_ values (320 nM for **11** and 402 nM for **12**), but they had very different values of *K*_i_ (1227 nM for **11** and 9092 nM for **12**) and *k*_2_ (0.875 ms^–1^ for **11** and 2.03 ms^–1^ for **12**). For all of the 11-, 12- and 13-membered analogues, introducing the second alkene increased the affinity for the target. This could be due to the entropic preference that is exhibited when there is limited conformational change upon binding to the target, because the conformation of the macrocycle is more constrained by the presence of the second alkene. To further investigate the differences in affinity for the target, we conducted a structural comparison. Stable conformers of compounds **4–12** were calculated with the help of NMR analysis by considering the vinyl-allylic proton coupling of the α,β-unsaturated carboxamide moiety.^16^ In the case of the 11-membered analogue **9**, the chemical shifts were reversed for the α- and β-alkenic protons adjacent to the carboxamide moiety (6.29 ppm for H-3 *vs.* 5.84 ppm for H-4). In conjunction with the observed increases in nuclear Overhauser effect for not only H-4 but also H-3, the dihedral angle between the vinyl and allylic protons of **9** was expected to be 90° < *θ* < 180°. A mixture of two conformers was observed for the 13-membered analogue with the (11*E*)-alkene (compound **10**) and the 11-membered analogue **8** in DMSO-*d*_6_ at 24 °C by ^1^H NMR. In both cases, the conformer that had its chemical shifts reversed for the α- and β-alkenic protons to the carboxamide moiety was the major conformer. A broadening of the peaks in the ^1^H NMR spectra was observed for analogues **7**, **8**, **11**, and **12**, indicating that they exist as a mixture of conformers at 24 °C. These structures were compared with syringolin A analogue **4**, which had the best *K*_i_ value, by merging the alkene adjacent to the carboxamide (Fig. 6). The conformations of the 12-membered analogues **5** and **6** were similar to that of **4**, and the alkene and the macrocycle moieties could be superimposed over **4**. The structural comparison is consistent with the fact that the 12-membered analogues tend to have better *K*_i_ values. Unlike the 12-membered analogues, the conformations of the 11- and 13-membered analogues differ from that of **4**. These conformational comparisons indicate that the mode of association in non-covalent interactions to form E·I is less desirable than that of **4**, and, presumably, a conformational change would be required to react with the Thr residue.

**Fig. 7:**
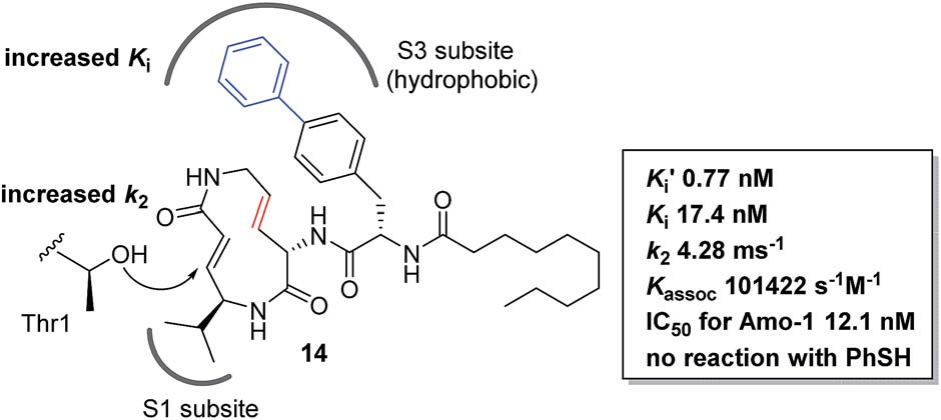
Design and biological properties of analogue **14**.

**Scheme 1:**
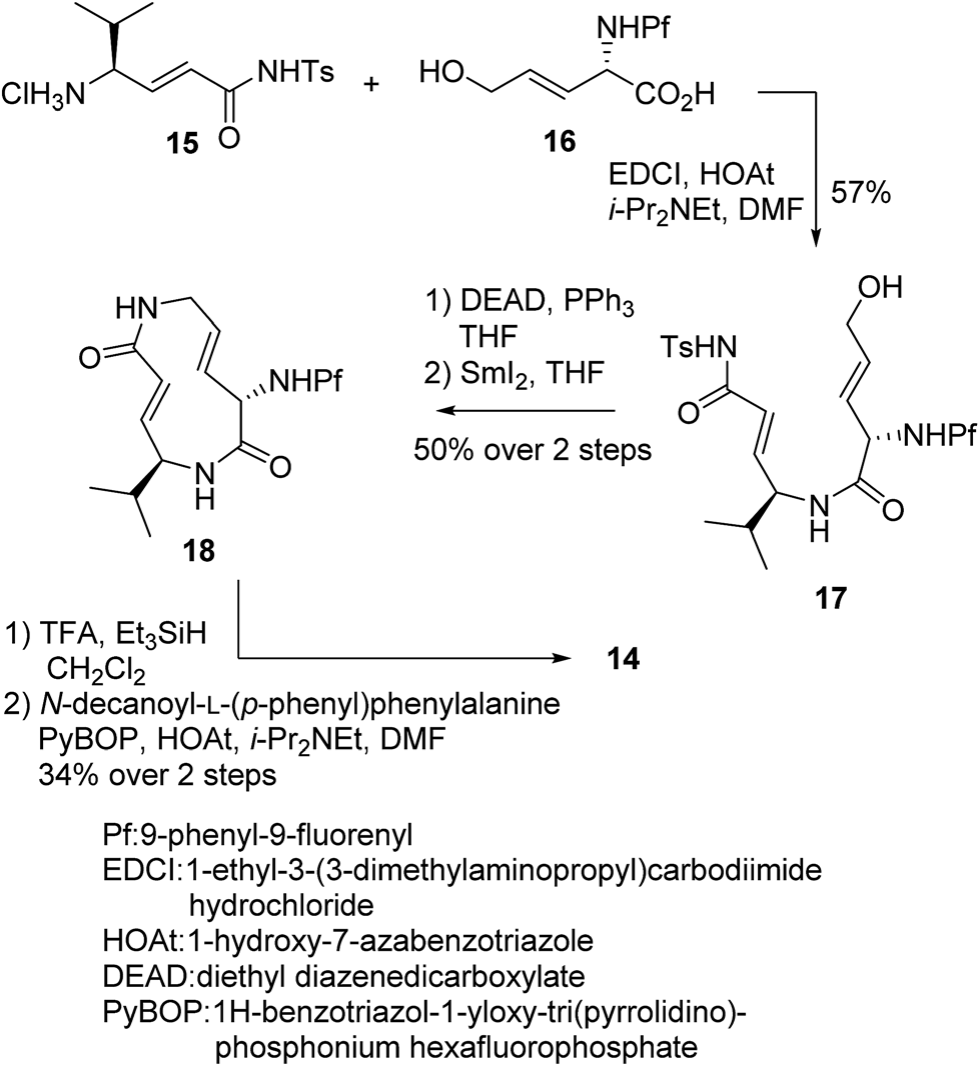
Synthesis of analogue **14**.

We prepared a set of analogues with a variety of *K*_i_ and *k*_2_ values, which allowed us to proceed with designing a new analogue. As a demonstration, **8** was chosen as a scaffold because it had the largest *k*_2_ value, and the reduced affinity of the macrocycle could be compensated for by attaching a specific side chain to the macrocycle. The side chains can be easily modulated. Thus, increasing the hydrophobic interaction to the S3 subsite of the proteasome β5 subunit by extending the phenyl group at the *p*-position of the l-phenylalanine residue of **8** led to the design of analogue **14** (Fig. 7). As shown in Scheme 1, **14** was efficiently synthesized by amide formation from the amine **15** (ref. 13) and carboxylic acid **16** followed by Mitsunobu cyclization of **17**, deprotection of the Ts group by SmI_2_ in THF, and installation of *N*-decanoyl-L-(*p*-phenyl)phenylalanine.^15*b*^ Analogue **14** was the most potent analogue based on its *K*′_i_, *K*_i_, *k*_2_, and *k*_assoc_ values of 0.77 nM, 42.2 nM, 4.28 ms^–1^, and 101 422 s^–1^ M^–1^, respectively. Although 1,4-addition of an alcohol to an α,β-unsaturated carboxamide is a very slow reaction under neutral conditions compared to thiol addition, the oxa-Michael addition between syringolins and the hydroxyl group of the Thr residue proceeds because of a proximity effect. In fact, analogue **14** did not react at all even with an excess of thiophenol in MeOH or DMSO under neutral conditions, indicating that **14** is a selective covalent inhibitor of the proteasome with very limited off-target effects. Moreover, this analogue shows a high cytotoxicity against human myeloma Amo-1 cells with an IC_50_ value of 12.1 nM.

## Conclusions

A systematic SAR study of a series of syringolin analogues was performed to elucidate detailed mechanistic information about the macrocycle and its effect on affinity and reaction rate. To the best of our knowledge, this study is the first systematic SAR study of a class of molecules embedding a reactive functional group within a macrocycle in terms of *K*_i_ and *k*_2_. A subtle change in the chemical structure of the macrocycle affects not only *K*_i_ but also *k*_2_. Once a library of macrocycles is available, one can select and modulate a compound with desired kinetic properties. A cyclic peptide is a promising scaffold for use in medicinal chemistry because multiple interactions with a target molecule can be achieved by modulating amino acid residues displayed on the macrocycle. These changes restrict the spatial orientations of these residues and contribute to the entropic changes that occur upon binding to the target. Accordingly, a set of cyclic peptides embedding a reactive functional group within the macrocycle would be a promising class of covalent inhibitor of targets reflecting the characteristics of cyclic peptides with more generality. The design, synthesis and evaluation of such a library are currently in progress.

## Conflicts of interest

There are no conflicts of interest to declare.
