# Supplementary material for: Impact of the structures of macrocyclic Michael acceptors on covalent proteasome inhibition
Source: Chem Sci. 2017 Aug 11;8(10):6959–63. doi: 10.1039/c7sc02941a (PMC5642145; doi:10.1039/c7sc02941a)
Supplement: Supplementary file 1 [file SC-008-C7SC02941A-s001.pdf]

Electronic Supplementary Material (ESI) for Chemical Science.
This journal is © The Royal Society of Chemistry 2017

Supporting Information

Impact of Structures of Macrocyclic Michael Acceptors on Covalent Proteasome Inhibition

Shun Kitahata[a,b], Fumika Yakushiji[a,b] ,and Satoshi Ichikawa[*a,b]

ichikawa@pharm.hokudai.ac.jp

[a]*Faculty of Pharmaceutical Science, Hokkaido University, Kita-12, Nishi-6, Kita-ku, Sapporo 060-0812, Japan.*
[b]*Center for Research and Education on Drug Discovery, Faculty of Pharmaceutical Science, Hokkaido University, Kita-12, Nishi-6, Kita-ku, Sapporo 060-0812, Japan.*

<u>Table of contents</u>

1) Abbreviations S2
2) General experimental methods S2
3) Experimental procedures and chemical data of compounds **5**, **7**, **8**, **9**, **10**, **11**, **12**, **13**, and **14** S3-S27
4) Evaluation of *in vitro* β5 proteasome inhibitory activity and determination of kinetic constants S28-S32
5) Evaluation of anti-proliferation activity against Amo-1 cells S33
6) Conformational calculation S34-S38
7) ^{1}H and ^{13}C NMR spectra of compounds S39-S105

1) Abbreviations

Boc: *tert*-butoxycarbonyl group

DEAD: diethyl Azodicarboxylate

DMAP: *N*,*N*-dimethyl-4-aminopyridine

EDCI: 1-(3-dimethylaminopropyl)-3-ethylcarbodiimide hydrochloride

HOAt: 1-hydroxy-7-azabenzotriazole

HOBt: 1-hydroxybenzotriazole monohydrate

KHMDS: potassium bis(trimethylsilyl)amide

NHS: *N*-hydroxysuccinimide

Pf: 9-phenyl-9-fluorenyl

Phe: phenylalanine

PyBOP: 1H-benzotriazol-1-yloxy-tri(pyrrolidino)phosphonium hexafluorophosphate

TMEDA: tetramethylethylenediamine

2) General experimental methods

All reactions except those carried out in aqueous phase were performed under argon atmosphere, unless otherwise noted. Materials were purchased from commercial suppliers and used without further purification, unless otherwise noted. Solvents were distilled according to the standard protocol. Isolated yields were calculated by weighing products. The weight of the starting materials and the products were not calibrated. Analytical thin layer chromatography (TLC) was performed on Merck silica gel $60F_{254}$ plates. Normal-phase column chromatography was performed on Merck silica gel 5715 or Wakogel 60N. Flash column chromatography was performed on Kanto Chemical Silica Gel 60N (spherical, neutral, 40-50 μm). High-flash column chromatography was performed on YAMAZEN Hi-Flash™ colomn silica gel (40 μm) or Fuji Silysia Chromatorex MB/PSQ (50-200 μm). ^{1}H NMR were measured in $CDCl_3$, DMSO-d_6 or methanol-d_4 solution, and reported in parts per million (δ) relative to tetramethylsilane (0.00 ppm) as internal standard using JEOL ECS400, ECX400, ECA500, unless otherwise noted. ^{13}C NMR were measured in $CDCl_3$, DMSO-d_6 or methanol-d_4 solution, and referenced to residual solvent peaks of $CDCl_3$ (77.0 ppm), DMSO-d_6 (39.5 ppm) or methanol-d_4 (49.0 ppm) using JEOL ECS400, ECX400, ECA500. Coupling constant (*J*) was reported in hertz (Hz). Abbreviations of multiplicity were as follows; s: singlet, d: doublet, t: triplet, q: quartet, m: multiplet, br: broad. Data were presented as follows; chemical shift (multiplicity, integration, coupling constant). Assignment was based on ^{1}H–^{1}H COSY, HMBC and HMQC NMR spectra. Mass spectra were obtained on Waters MICRO MASS LCT–premier and the mass analyzer type used for the HRMS measurements was TOF. Optical rotation was measured on a Rudolph Research Analytical Autopol IV automatic polarimeter.

3) Experimental procedures and chemical data of compounds 5, 7, 8, 9, 10, 11, 12, 13, and 14

Scheme S1. Synthesis of **5**

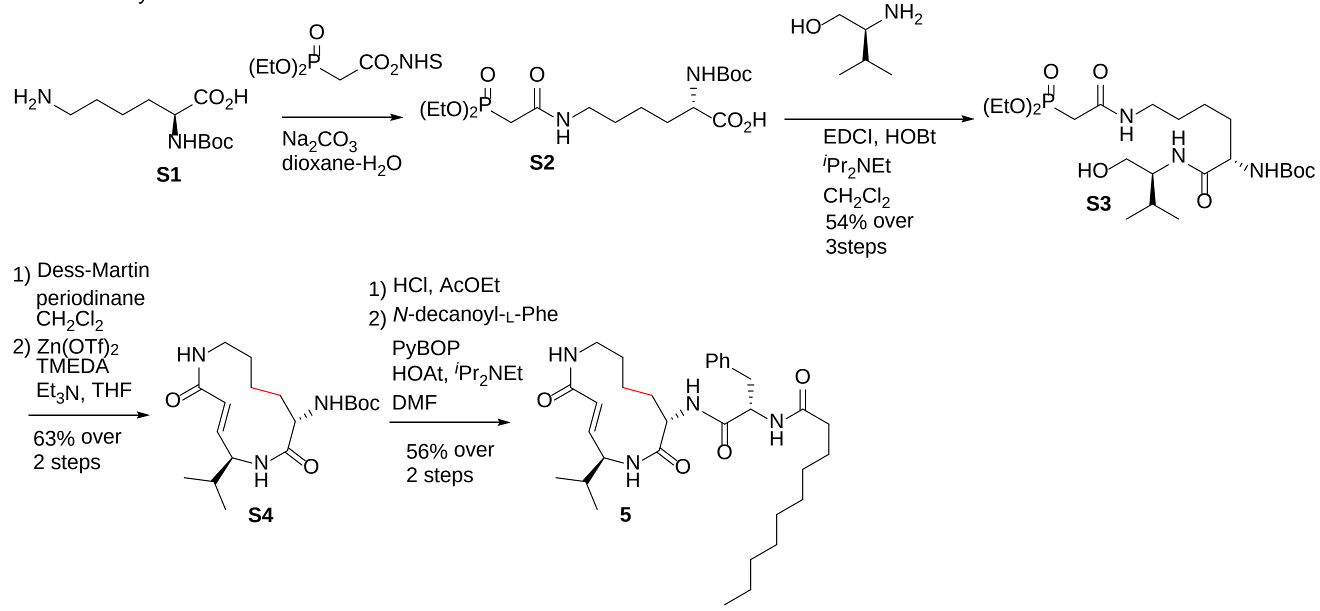

***tert*-Butyl {(*S*)-6-[2-(Diethoxyphosphoryl)acetamido]-1-{[(*R*)-1-hydroxy-3-methylbutan-2-yl]amino}-1-oxohexan-2-yl}carbamate (S3)**

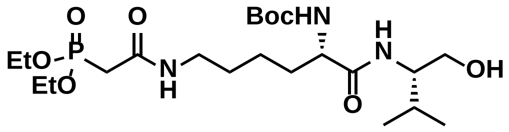

A solution of **S1** (710 mg, 2.9 mmol) and Na_2CO_3 (610 mg, 5.7 mmol) in dioxane (3 mL) and H_2O (5 mL) was added to a solution of diethylphosphonoacetic acid NHS ester (1.4 g, 5.8 mmol) in dioxane (2 mL) at 0 °C, and the mixture was stirred at room temperature for 16 h. The mixture was partitioned between Et_2O and *sat. aq.* $NaHCO_3$. The aqueous phase was acidified with 1 M *aq.* HCl, and extracted with AcOEt. The organic phase was washed with brine, dried (Na_2SO_4), filtered, and concentrated *in vacuo* to give a crude **S2**. A solution of the crude **S2**, L-valinol (520 μL, 4.60 mmol), HOBt·H_2O (630 mg, 4.6 mmol) and ^{i}Pr$_2$NEt (1.30 mL, 9.3 mmol) in DMF (16 mL) was treated with EDCI (720 mg, 4.6 mmol) at room temperature for 11 h. The mixture was partitioned between with AcOEt and H_2O. The organic phase was washed with 1 M *aq.* HCl, *sat. aq.* $NaHCO_3$, H_2O, and brine, dried (Na_2SO_4), filtered, and concentrated *in vacuo*. The residue was purified by hi-flash silica gel column chromatography (ϕ 2.6×10 cm, 0→2% MeOH/$CHCl_3$) to afford **S3** (860 mg, 1.7 mmol, 54% over 2 steps) as a colorless oil. ^{1}H NMR ($CDCl_3$, 400 MHz) δ 6.92 (m, 1H, H-7), 6.42 (d, 1H, H-5', $J_{5',1'}$ = 8.7 Hz), 5.39 (d, 1H, N*H*, $J_{NH,2}$ = 7.8 Hz), 4.15 (m, 4H, CH_3CH_2O), 4.05 (m, 1H, H-2), 3.82 (m, 2H, H-2'), 3.71 (m, 1H, H-1'), 3.30 (m, 2H, H-6), 2.84 (d, 2H, H-1", *J* = 21.1 Hz), 1.86 (m, 1H, OH), 1.53-1.81 (m, 6H, H-3, H-4, H-5), 1.53 (m, 1H, H-3'), 1.43 (s, 9H, ^{t}Bu), 1.33 (m, 6H, CH_3CH_2O), 0.95 (d, 3H, H-4', $J_{4',3'}$ = 6.9 Hz), 0.94 (d, 3H, H-4', $J_{4',3'}$ = 6.9 Hz). This is a known compound. [S3)]

***tert*-Butyl [(5*S*,8*S*,*E*)-5-Isopropyl-2,7-dioxo-1,6-diazacyclododec-3-en-8-yl]carbamate (S4)**

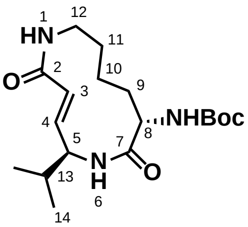

A solution of **S3** (820 mg, 1.6 mmol) in CH_2Cl_2 (16 mL) was treated with Dess-Martin periodinane (750 mg, 1.8 mmol) at room temperature for 1 h. Ethyl acetate, *sat. aq.* $NaHCO_3$, and *sat. aq.* $Na_2S_2O_3$ were added to the mixture, which was stirred for further 10 min. The organic phase was dried (Na_2SO_4), filtered, and concentrated *in vacuo* to afford a crude aldehyde. A solution of $Zn(OTf)_2$ (1.28 mg, 3.54 mmol), TMEDA (287 μL, 3.54 mmol) in THF (222 mL) was treated with Et_3N (896 μL, 6.44 mmol) at room temperature for 15 min. A solution of the crude aldehyde in THF (100 mL) was added to the mixture, which was stirred for 22 h. The mixture was concentrated *in vacuo* and the residue was partitioned between AcOEt and 1 M *aq.* HCl. The organic phase was washed with brine, dried (Na_2SO_4), filtered, and concentrated *in vacuo*. The residue was purified by silica gel column chromatography (ϕ 3×10 cm, 1→2% MeOH/$CHCl_3$) to afford **S4** (360 mg, 1.0 mmol, 63% over 2 steps) as a white solid. ^{1}H NMR (CD_3OD, 500 MHz) δ 6.81 (dd, 1H, H-4, $J_{4,3}$ = 16, $J_{4,5}$ = 5.2 Hz), 6.13 (d, 1H, H-3, $J_{3,4}$ = 15.5 Hz), 4.21 (br s, 1H, H-8), 4.01 (dd, 1H, H-5, $J_{5,4}$ = 5.2, $J_{5,13}$ = 8.1 Hz), 3.27 (dd, 1H, H-12, $J_{12,11}$ = 13.2, J_{gem} = 13.8 Hz), 2.89 (dt, 1H, H-12, $J_{12,11}$ = 4.0, J_{gem} = 12.1 Hz), 1.87 (m, 1H, H-9), 1.60 (m, 1H, H-9), 1.58 (m, 1H, H-13), 1.43 (m, 1H, H-11), 1.23 (s, 9H, ^{t}Bu), 1.18 (m, 3H, H-10, H-11), 0.84 (d, 1H, H-14, $J_{14,13}$ = 6.9 Hz), 0.81 (d, 1H, H-14, $J_{14,13}$ = 6.9 Hz); ESIMS-LR *m/z* 376 [(M+Na)$^+$]; ESIMS-HR calcd for $C_{18}H_{31}O_4N_3Na$ 376.2207, found 376.2210. This is a known compound.[S3)]

***N*-{(*S*)-1-{[(5*S*,8*S*,*E*)-5-Isopropyl-2,7-dioxo-1,6-diazacyclododec-3-en-8-yl]amino}-1-oxo-3-phenylpropan-2-yl}decanamide (5)**

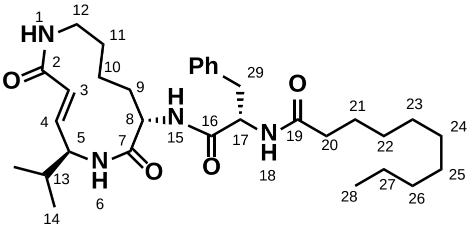

A solution of **S4** (39 mg, 0.11 mmol) was treated with 4 M HCl in AcOEt (1 mL) at room temperature for 1 h. The mixture was concentrated *in vacuo*. A solution of the residue, *N*-decanoyl-L-phenylalanine (39 mg, 120 μmol), ^{i}Pr$_2$NEt (56 μL, 330 μmol), and HOAt (26 mg, 160 μmol) in DMF (1.2 mL) was treated with PyBOP (86 mg, 160 μmol) at 0 °C, and the mixture was stirred at room temperature for 15 h. The mixture was partitioned between AcOEt and 1 M *aq.* HCl. The mixture was filtered, and the insoluble contents was washed with *sat. aq.* $NaHCO_3$, H_2O, and AcOEt. The residue was purified by preparative-TLC (10% MeOH/$CHCl_3$) to afford **5** (34 mg, 61 μmol, 56% over 2 steps) as a white solid. ^{1}H NMR (DMSO-d_6, 500 MHz) δ 8.45 (d, 1H, H-6, $J_{6,5}$ = 8.0 Hz), 8.14 (d, 1H, H-18, $J_{18,17}$ = 8.1 Hz), 7.66 (d, 1H, H-15, $J_{15,8}$ = 7.5 Hz), 7.38 (t, 1H, H-1, $J_{1,12}$ = 6.3 Hz), 7.15-7.23 (m, 5H, Ar), 6.77 (dd, 1H, H-4, $J_{4,3}$ = 15.5, $J_{4,5}$ = 4.6 Hz), 6.25 (d, 1H, H-3, $J_{3,4}$ = 14.9 Hz), 4.61 (m, 1H, H-8), 4.50 (m, 1H, H-17), 4.11 (m, 1H, H-5), 3.27 (m, 1H, H-12), 3.01 (m, 1H, H-29), 2.93 (m, 1H, H-12), 2.73 (dd, 1H, H-29, J_{gem} = 13.2, $J_{29,17}$ = 11.5 Hz), 2.13 (m, 1H, H-9), 1.98 (t, 2H, H-20, $J_{20,21}$ = 6.9 Hz), 1.79 (m, 1H, H-13), 1.62 (m, 1H, H-12), 1.44 (m, 1H, H-11), 1.04-1.34 (m, 14H, H-21, H-22, H-23, H-24, H-25, H-26, H-27), 0.95 (d, 3H, H-14, $J_{14,13}$ = 6.3 Hz), 0.91 (d, 3H, H-14, $J_{14,13}$ = 6.3 Hz), 0.86 (t, 3H, H-28, $J_{28,27}$ = 6.3 Hz); ^{13}C NMR (DMSO-d_6, 125 MHz) δ 172.3, 170.8, 170.5, 165.8, 144.9, 138.2, 129.1, 127.9, 126.1, 119.9, 56.1, 53.8, 51.3, 38.0, 37.0, 35.2, 31.4, 31.2, 30.7, 30.3, 29.9, 28.8, 28.7, 28.4, 25.2, 22.1, 20.0, 19.3, 17.2, 14.0); ESIMS-LR *m/z* 577 [(M+Na)$^+$]; ESIMS-HR calcd for $C_{32}H_{50}O_4N_4Na$ 577.3724, found 577.3732.

Scheme S2. Synthesis of **7**

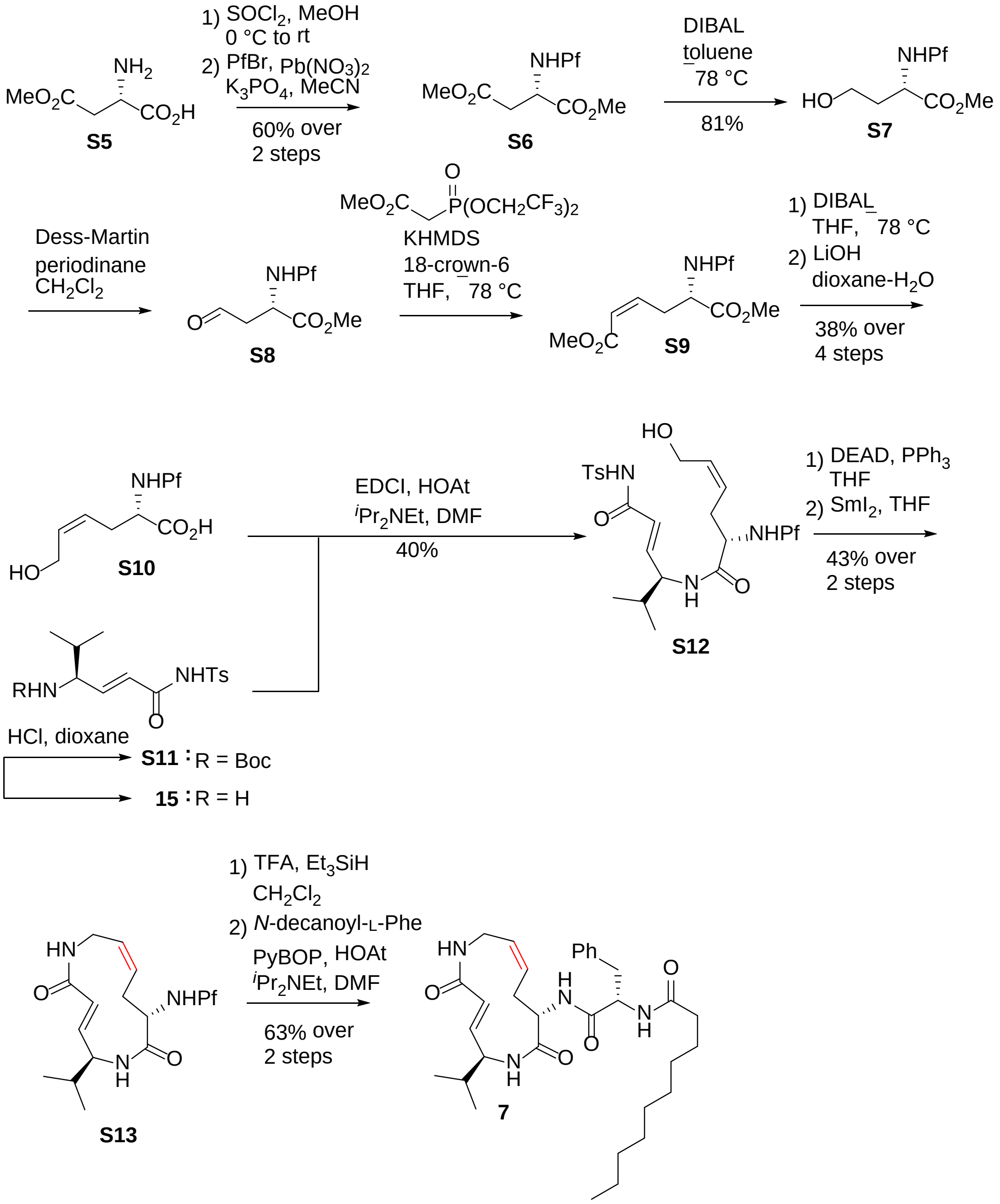

Methyl (*S*)-3-Methoxycarbonyl-2-[(9-phenyl-9H-fluoren-9-yl)amino]propanoate (S6)

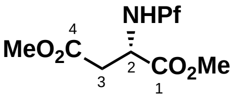

A solution of **S5** (5.0 g, 27 mmol) in MeOH (14 mL) was treated with $SOCl_2$ (2.4 mL, 32 mmol) at 0 °C, and the mixture was stirred at room temperature for 72 h. The mixture was concentrated *in vacuo*. A mixture of the residue, $Pb(NO_3)_2$ (9.2 g, 28 mmol) and K_3PO_4 (12 g, 56 mmol) in MeCN (56 mL) was treated with PfBr (12 g, 36 mmol) at room temperature for 24 h. The insoluble contents were filtered off, and the filtrate was concentrated *in vacuo*. The residue was partitioned between AcOEt and H_2O. The organic phase was washed with *sat. aq.* $NaHCO_3$, H_2O, and brine, dried (Na_2SO_4), filtered, and

concentrated *in vacuo.* The residue was purified by silica gel column chromatography (ϕ 10 × 25 cm, 13% AcOEt/hexane) to afford **S6** (6.4 g, 16 mmol, 60% over 2 steps) as a colorless oil. ^{1}H NMR ($CDCl_3$, 400 MHz) δ 7.68 (m, 2H, Ar), 7.18-7.39 (m 11H, Ar), 3.65 (s, 3H, 1-OMe), 3.34 (s, 3H, 4-OMe), 3.21 (m, 1H, N*H*), 3.01 (m, 1H, H-2), 2.51 (dd, 1H, H-3, $J_{3,\,2}$ = 15.1, J_{gem} = 6.9 Hz), 2.34 (dd, 1H, H-3, $J_{3,2}$ = 15.1, J_{gem} = 5.5 Hz). This is a known compound. [S4)]

Methyl 4-Hydroxy-2-[(9-phenyl-9H-fluoren-9-yl)amino]butanoate (S7)

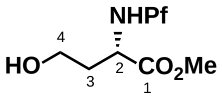

A solution of **S6** (1.2 g, 3.1 mmol) in toluene (31 mL) was treated with DIBAL (3.4 mL, 3.4 mmol, 1.0 M in toluene) at −78 °C for 30 min. DIBAL (0.61 mL, 0.61 mmol, 1.0 M in toluene) was added to the mixture, which was stirred for 30 min. Acetone (1 mL), H_2O (1 mL), and $NaHCO_3$ were added to the mixture, which was stirred for 5 min, filtered, and concentrated *in vacuo.* The residue was purified by hi-flash silica gel column chromatography (ϕ 2.6×10 cm, 17% AcOEt/hexane) to afford **S7** (920 mg, 2.5 mmol, 81%) as a colorless oil. ^{1}H NMR ($CDCl_3$, 400 MHz) δ 7.20-7.70 (m, 13H, Ar), 3.72 (m, 1H, H-4), 3.56 (t, 1H, H-4, $J_{4,3}$ = 11 Hz), 3.29 (s, 3H, CO_2Me), 2.77 (dd, 1H, H-2, $J_{2,3}$ = 8.2, $J_{2,3}$ = 3.6 Hz), 1.73 (m, 1H, H-3), 1.59 (br s, 1H, OH), 1.54 (m, 1H, H-3). This is a known compound. [S4)]

(*S*,*Z*)-6-Hydroxy-2-[(9-phenyl-9H-fluoren-9-yl)amino]hex-4-enoic acid (S10)

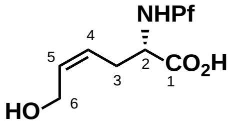

A solution of **S7** (2.2 g, 5.9 mmol) in CH_2Cl_2 (58 mL) was treated with Dess-Martin periodinane (2.7 g, 6.4 mmol) at room temperature for 2 h. Ethyl acetate, *sat. aq.* $NaHCO_3$, and *sat. aq.* $Na_2S_2O_3$ were added to the mixture, which was partitioned between AcOEt and H_2O. The organic phase was washed with *sat. aq.* $NaHCO_3$, H_2O, and brine, dried (Na_2SO_4), filtered, and concentrated *in vacuo.* The residue was purified by silica gel column chromatography (ϕ 5×10 cm, 11% AcOEt/hexane) to afford a crude **S8** (2.0 g) as a colorless oil. A solution of $(CF_3CH_2O)_2P(=O)CH_2CO_2Me$ (530 mg, 1.7 mmol) and 18-crown-6 (2.2 g, 8.4 mmol) in THF (20 mL) was treated by KHMDS (3.4 mL, 1.7 mmol, 0.5 M in toluene) at −78 °C for 40 min. A solution of **S8** (420 mg, 1.1 mmol) in THF (5 mL) was added to the mixture at −78 °C for 2 h. The reaction was quenched by addition of *sat. aq.* NH_4Cl. The mixture was partitioned between AcOEt and H_2O, and the organic phase was washed with brine, dried (Na_2SO_4), filtered, and concentrated *in vacuo.* The residue was purified by hi-flash silica gel column chromatography (ϕ 2×6.5 cm, 0→10→20% AcOEt/hexane) to afford a crude **S9** (230 mg, 0.54 mmol) as a colorless oil. A solution of a crude **S9** (230 mg) in THF (5.4 mL) was treated with DIBAL (1.4 mL, 1.4 mmol, 1.0 M in toluene) at −78 °C for 2 h. Additional DIBAL (1.4 mL, 1.4 mmol, 1.0 M in toluene) was added to the mixture, which was stirred for further 1 h. Methanol and *sat. aq.* $KNaC_4H_4O_6$ were added to the mixture, which was stirred for 30 min. The mixture was diluted with AcOEt and washed with H_2O, 1 M *aq.* HCl, *sat. aq.* $NaHCO_3$, H_2O, and brine, dried (Na_2SO_4), filtered, and concentrated *in vacuo.* A solution of the residue (220 mg, 0.54 mmol)

in dioxane (4 mL) and H_2O (2 mL) was treated with LiOH·H_2O (140 mg, 3.2 mmol) for 2 h. The mixuture was partitioned between AcOEt and 1 M *aq*. HCl. The organic phase was washed with brine, dried (Na_2SO_4), filtered, and concentrated *in vacuo*. The residue was purified by hi-flash silica gel column chromatography (ϕ 2×6.5 cm, 0→1→3% MeOH/$CHCl_3$) to afford **S10** (180 mg, 0.46 mmol, 38% over 4 steps) as a colorless oil. ^{1}H NMR (CD_3OD, 400 MHz) δ 7.79 (m, 2H, Ar), 7.33 (m, 11H, Ar), 5.74 (dt, 1H, H-5, $J_{5,4}$ = 11, $J_{5,6}$ = 6.8 Hz), 5.36 (dt, 1H, H-4, $J_{4,5}$ = 11, $J_{4,3}$ = 7.8 Hz), 4.02 (d, 2H, H-6, $J_{6,5}$ = 6.4 Hz), 2.63 (t, 1H, H-2, $J_{2,3}$ = 6.0 Hz), 2.32 (m, 1H, H-3) 2.18 (m, 1H, H-3); ^{13}C NMR (DMSO-d_6, 100 MHz) δ 177.1, 148.2, 147.6, 143.9, 142.3, 142.2, 132.9, 130.4, 130.3, 129.6, 129.2, 129.0, 128.8, 128.4, 127.4, 127.0, 126.9, 121.3, 121.2, 74.4, 58.1, 57.5, 32.8, 30.2; ESIMS-LR (negative mode) *m*/*z* 384 [(M-H)$^-$]; ESIMS-HR calcd for $C_{25}H_{22}O_3N$ 384.1605, found 384.1607.; $[\alpha]^{20}_D$ −16.4 (*c* 1.10, MeOH).

(4*S*,2*E*)-4-{(2*S*,4*Z*)-6-Hydroxy-2-[(9-phenyl-9H-fluoren-9-yl)amino]hex-4-enamido}-5-methyl-*N*-(*p*-toluenesulfonamide)hex-2-enamide (S12)

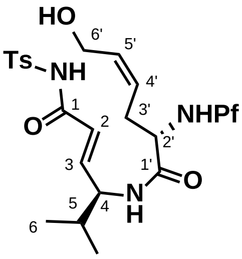

Compound **S11**[S2)] (260 mg, 0.65 mmol) was treated with 4 M HCl in dioxane (6.5 mL) at room temperature for 40 min. The mixture was concentrated *in vacuo* to give **15**. A solution of **S10** (170 mg, 0.43 mmol), **15** (220 mg, 0.65 mmol), ^{i}Pr$_2$NEt (360 μL, 2.6 mmol), and HOAt (180 mg, 1.3 mmol) in DMF (6.5 mL) was treated with EDCI (200 mg, 1.3 mmol) at room temperature for 22 h. The mixture was partitioned between AcOEt and H_2O. The organic phase was washed with 1 M *aq*. HCl, *sat*. *aq*. $NaHCO_3$, H_2O, and brine, dried (Na_2SO_4), filtered, and concentrated *in vacuo*. The residue was purified by hi-flash silica gel column chromatography (ϕ 2×6.5 cm, 0→1→2% MeOH/$CHCl_3$) to afford **S12** (120 mg, 0.18 mmol, 40%) as a colorless oil. ^{1}H NMR (CD_3OD, 400 MHz) δ 7.91 (m, 2H, Ar), 7.70 (m, 2H, Ar), 6.92-7.38 (m, 15H, Ar), 6.63 (dd, 1H, H-3, $J_{3,2}$ = 15.6, $J_{3,4}$ = 8.2 Hz), 5.80 (d, 1H. H-2, $J_{2,3}$ = 15.6 Hz), 5.66 (m, 1H, H-4'), 5.32 (m, 1H, H-5'), 4.02 (d, 2H, H-6', $J_{6',5'}$ = 4.6 Hz), 3.89 (dd, 1H, H-4, $J_{4,3}$ = 6.9, $J_{3,5}$ = 6.4 Hz), 2.56 (m, 2H, H-2'), 2.41 (s, 3H, Ts-Me), 2.27 (m, 1H, H-3'), 2.17 (m, 1H, H-3'), 1.63 (m, 1H, H-5), 0.77 (d, 3H, H-6, $J_{6,5}$ = 6.4 Hz), 0.72 (d, 3H, H-6, $J_{6,5}$ = 6.4 Hz); ^{13}C NMR (DMSO-d_6, 100 MHz) δ 176.6, 165.1, 150.6, 149.9, 147.4, 146.2, 145.8, 142.2, 141.7, 138.0, 132.7, 130.5, 129.7, 129.6, 129.3, 128.9, 128.7, 128.2, 127.3, 127.1, 126.6, 124.3, 121.0, 120.9, 74.3, 58.1, 57.6, 57.4, 34.4, 32.7, 21.6, 19.5, 19.1; ESIMS-LR *m*/*z* 664 [(M+H)$^+$]; ESIMS-HR calcd for $C_{39}H_{42}O_5N_3S$ 664.2840, found 664.2850. $[\alpha]^{20}_D$ −123.4 (*c* 0.54, MeOH).

(3*E*,5*S*,8*S*,10*Z*)-5-Isopropyl-8-[(9-phenyl-9H-fluoren-9-yl)amino]-1,6-diazacyclododeca-3,10-diene-2,7-dione (S13)

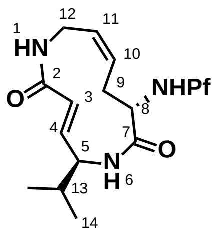

A solution of **S12** (110 mg, 170 µmol) and PPh_3 (87 mg, 330 µmol) in THF (83 mL) was treated with DEAD (150 µL, 330 µmol, 2.2 M in toluene) at room temperature for 10 min. The mixture was concentrated *in vacuo*. The residue was purified by hi-flash silica gel column chromatography (ϕ 2.6×10 cm, 0→1% MeOH/$CHCl_3$) to afford a crude sulfonamide as a white solid. A solution of the crude sulfonamide in THF (1.6 mL) was treated with 0.1 M SmI_2 solution in THF (10 mL) at room temperature for 1 min. The mixture was partitioned between AcOEt and 1 M *aq.* HCl. The organic phase was washed with brine, dried (Na_2SO_4), filtered, and concentrated *in vacuo*. The residue was purified by hi-flash silica gel column chromatography (ϕ 2.6×10 cm, 0→1% MeOH/$CHCl_3$) to afford **S13** (35 mg, 72 µmol, 43% over 2 steps) as a white solid. ^{1}H NMR (DMSO-d_6, 500 MHz) δ 7.84 (m, 2H, Ar), 7.46 (m, 1H, H-1), 7.41 (d, 1H, H-6, $J_{6,5}$ = 7.4 Hz), 7.36 (m, 11H, Ar), 6.47 (dd, 1H, H-4, $J_{4,3}$ = 14.9, $J_{4,5}$ = 3.4 Hz), 5.82 (d, 1H, H-3, $J_{3,4}$ = 14.9 Hz), 5.74 (dd, 1H, H-10, $J_{10,11}$ = $J_{10,9a}$ = 11.5 Hz), 5.20 (dd,1H, H-11, $J_{11,12b}$ = 11.5 Hz), 3.94 (br s, 1H, H-5), 3.81 (ddd, 1H, H-12b, $J_{12b,12a}$ = $J_{12b,11}$ = 12, $J_{12b,1}$ = 5.2 Hz), 3.67 (br s, 1H, H-5), 3.16 (d, 1H, H-12a, $J_{12a,12b}$ = 12 Hz), 2.86 (br s, 1H, H-8), 2.74 (dd, 1H, H-9a, $J_{9a,9b}$ = 13, $J_{9a,8}$ = 12 Hz), 1.87 (d, 1H, H-9b, $J_{9b,9a}$ = 13 Hz), 0.76 (d, 3H, H-14, $J_{14,13}$ = 6.9 Hz), 0.64 (d, 3H, H-14, $J_{14,13}$ = 6.9 Hz); ^{13}C NMR (DMSO-d_6, 125 MHz) δ 172.9, 166.0, 149.6, 144.9, 142.3, 140.3 139.7, 128.3, 128.2, 127.7, 127.6, 127.1, 125.8, 120.4, 120.2, 120.0, 72.9, 55.8, 53.9, 34.4, 30.5, 19.4, 19.0, 14.0; ESIMS-LR *m/z* 514 [$(M+Na)^+$]; ESIMS-HR calcd for $C_{32}H_{33}O_2N_3Na$ 514.2465, found 514.2473.; $[\alpha]^{20}_{D}$ −167.0 (*c* 1.10, MeOH).

***N*-{(*S*)-1-{[(3*E*,5*S*,8*S*,10*Z*)-5-Isopropyl-2,7-dioxo-1,6-diazacyclododeca-3,10-dien-8-yl]amino}-1-oxo-3-phenylpropan-2-yl}decanamide (7)**

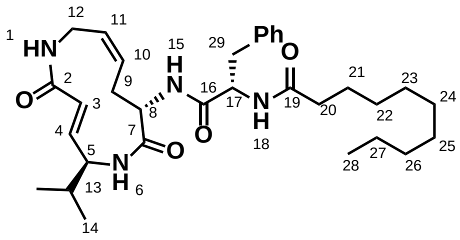

A solution of **S13** (21 mg, 43 µmol) and Et_3SiH (17 µL, 110 mmol) in CH_2Cl_2 (0.85 mL) was treated with TFA (0.85 mL) at 0 °C, and the mixture was stirred at room temperature for 1 h. The mixture was concentrated *in vacuo*. A solution of the residue, *N*-decanoyl-L-phenylalanine (16 mg, 51 µmol), $^{i}Pr_2NEt$ (15 µL, 85 mmol), and HOAt (10 mg, 64 mmol) in DMF (0.43 mL) was treated with PyBOP (33 mg, 64 mmol) at room temperature for 18 h. The mixture was partitioned between AcOEt and H_2O. The organic phase was washed with 1 M *aq.* HCl, *sat. aq.* $NaHCO_3$, H_2O, and brine, dried (Na_2SO_4), filtered, and concentrated *in vacuo*. The residue was purified by hi-flash silica gel column chromatography (ϕ 1.6 × 6 cm, 0 → 1 → 2% MeOH/$CHCl_3$) followed by preparative-TLC (10% MeOH/$CHCl_3$) to afford **7** (15 mg, 27 µmol, 63% over 2 steps) as a white solid. ^{1}H NMR (DMSO-d_6, 500 MHz) δ 8.12 (d, 1H, H-15, $J_{15,8}$ = 8.1 Hz), 8.07 (d, 1H, H-6, $J_{6,5}$ = 8.6 Hz), 7.92 (d, 1H, H-18, $J_{18,17}$ = 6.3 Hz), 7.58 (br s, 1H, H-1), 7.23 (m, 4H, Ar), 7.16 (m, 1H, Ar), 5.58 (d, 1H, H-4, $J_{4,3}$ = 14.9 Hz), 6.02 (d, 1H, H-3, $J_{3,4}$ = 14.9 Hz), 5.51 (t, 1H, H-10, $J_{10,11}$ = 11.5), 5.26 (m, 1H, H-11), 4.71 (br s, 1H, H-17), 4.56 (dt, 1H, H-8, $J_{8,15}$ = 14.9, $J_{8,9}$ = 4.1 Hz), 4.19 (br s, 1H, H-5), 3.99 (br s, 1H, H-12), 3.25 (br s, 1H, H-12), 3.08 (m, 1H, H-29), 3.03 (d, 1H, H-9, $J_{9,10}$ = 10.4 Hz), 2.75 (dd, 1H, H-9, J_{gem} = 10.9, $J_{9,8}$ = 10.9 Hz), 2.31 (d, 1H, H-29, $J_{29,17}$ = 14.4 Hz), 2.00 (t, 2H, H-20, $J_{20,21}$ = 7.5 Hz), 1.80 (dt, 1H, H-5, $J_{5,3}$ = 13, $J_{5,4}$ = 6.3 Hz), 1.35 (tt, 2H, H-21, $J_{21,20}$ = 7.5, $J_{21,22}$ = 7.5 Hz), 1.06-1.27 (m, 12H,

H-22, H-23, H-24, H-25, H-26, H-27), 0.93 (d, 6H, H-14, $J_{14,13}$ = 7.5 Hz), 0.86 (t, 3H, H-28, $J_{28,27}$ = 7.5 Hz); ^{13}C NMR (DMSO-d_6, 125 MHz) δ 172.2, 170.7, 170.0, 166.0, 142.1, 138.1, 129.1, 128.4, 127.9, 126.1, 125.1, 120.4, 55.8, 53.8, 50.9, 37.0, 35.2, 31.3, 30.6, 30.3, 28.8, 28.7, 28.4, 25.2, 22.1, 19.3, 19.2, 15.8, 14.0; ESIMS -LR *m/z* 575 [(M+Na)$^+$]; ESIMS-HR calcd for $C_{32}H_{48}O_4N_4Na$ 575.3568, found 575.3570.

Scheme S3. Synthesis of **8**

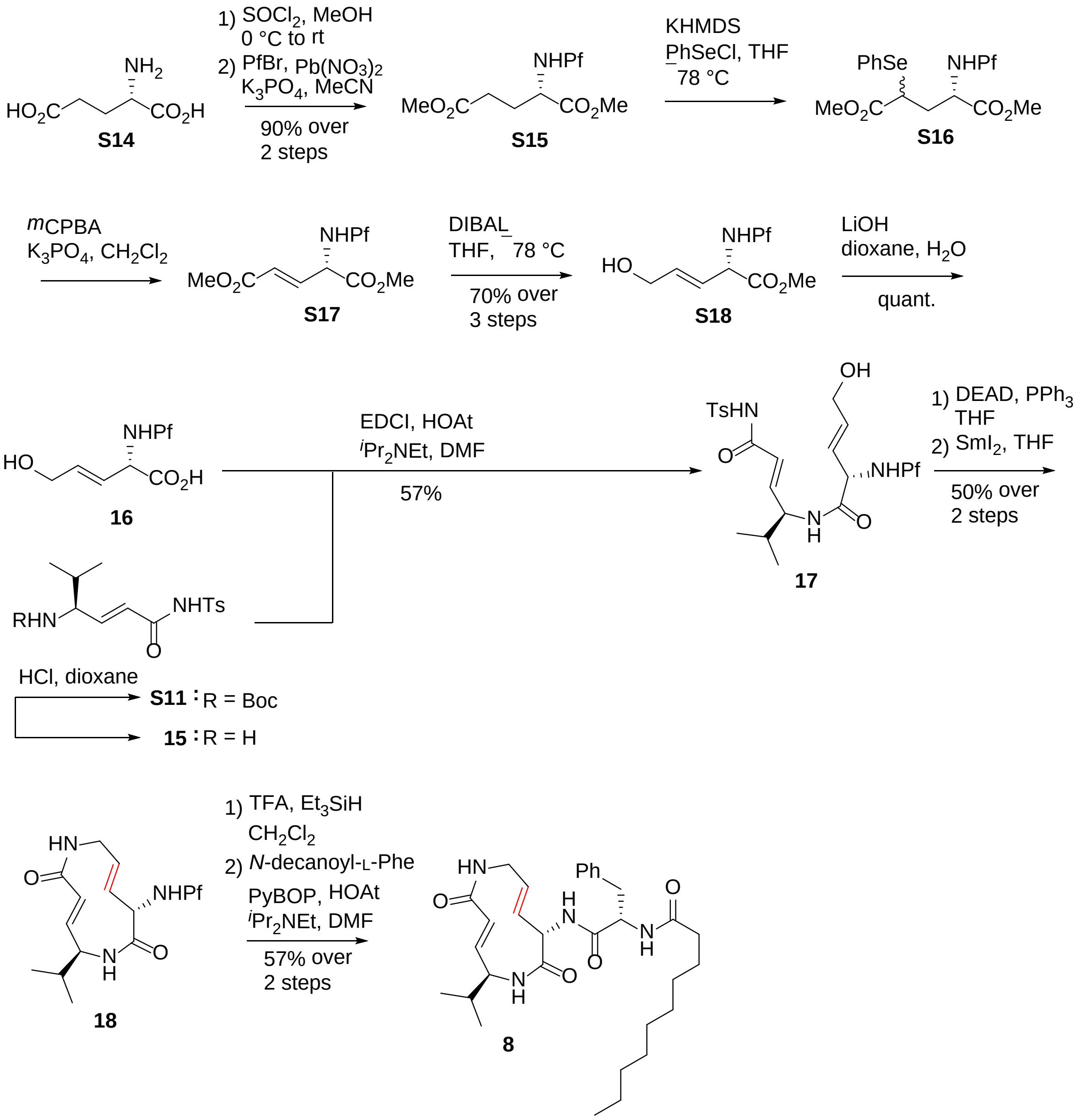

Methyl (*S*)-4-Methoxycarbonyl-2-[(9-phenyl-9H-fluoren-9-yl)amino)]butanoate (S15)

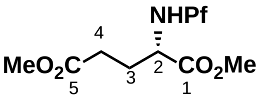

A solution of **S14** (6.2 g, 42 mmol) in MeOH (20 mL) was treated with $SOCl_2$ (3.8 mL, 52 mmol) at 0 °C, and the mixture was stirred at room temperature for 68 h. The mixture was concentrated *in vacuo* to afford a crude dimethyl glutamate (9.2 g). A mixture of the dimethyl glutamate (8.0 g, 46 mmol), $Pb(NO_3)_2$ (15 g, 46 mmol) and K_3PO_4 (19 g, 91 mmol) in MeCN (91 mL) was treated with PfBr (15 g, 46 mmol) at room temperature for 48 h. The insoluble contents were filtered off, and the filtrate was concentrated *in vacuo*. The residue was partitioned between Et_2O and H_2O, and the organic phase was washed with 1 M *aq.* HCl, *sat. aq.* $NaHCO_3$, H_2O, and brine, dried (Na_2SO_4), filtered, and concentrated *in vacuo.* The residue was purified by silica gel column chromatography (ϕ 10 × 15 cm, 11% AcOEt/hexane) to afford **S15** (14 g, 33 mmol, 90% over 2 steps) as a yellow oil. ^{1}H NMR ($CDCl_3$, 400 MHz) δ 7.66 (m, 2H, Ar), 7.15-7.40 (m, 11H,

Ar), 3.63 (s, 3H, 1-OMe), 3.25 (s, 3H, 5-OMe), 2.95 (br s, 1H, H-2), 2.58 (br s, 1H, NH), 2.47 (ddd, 1H, H-4, J_{gem} = 17, $J_{4,3}$ = 8.3, $J_{4,3}$ = 6.4 Hz), 2.35 (ddd, 1H, H-4, J_{gem} = 17, $J_{4,3}$ = 7.8 Hz). This is a known compound. [S1)]

Methyl (*S*,*E*)-5-Hydroxy-2-[(9-phenyl-9H-fluoren-9-yl)amino]pent-3-enoate (S18)

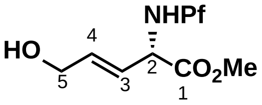

A solution of **S15** (1.0 g, 2.4 mmol) in THF (8 mL) was treated with KHMDS (11 mL, 5.3 mmol, 0.5 M in toluene) at −78 °C for 30 min. A solution of PhSeCl (1.0 g, 5.3 mmol) in THF (2 mL) was added to the mixture, and the solution was stirred at the same temperature for 3 h. The reaction was quenched by addition of AcOH (1 mL) at room temperature, and the mixture was partitioned between AcOEt and H_2O. The organic phase was washed with *sat. aq.* $NaHCO_3$, H_2O, and brine, dried (Na_2SO_4), filtered, and concentrated *in vacuo* to give **S16**. A mixture of the crude **S16** and K_3PO_4 (1.5 g, 7.2 mmol) in CH_2Cl_2 (12 mL) was treated with *m*CPBA (1.0 g, 6.0 mmol) at 0 °C for 30 min. Saturated *aq.* $Na_2S_2O_3$ was added to the mixture and the resulting mixture was filtered. The filtrate was partitioned between AcOEt and H_2O, and the organic phase was washed with *sat. aq.* $NaHCO_3$, and brine, dried (Na_2SO_4), filtered, and concentrated *in vacuo* ro give **S17**. A solution of the crude **S17** in THF (24 mL) was treated with DIBAL (7.1 mL, 7.1 mmol, 1.0 M in toluene) at −40 °C for 1 h. Methanol and *sat*. *aq*. $KNaC_4H_4O_6$ were added to the mixture, which was stirred for 30 min. The mixture was partitioned between AcOEt and H_2O, and the organic phase was washed with 1 M *aq.* HCl, H_2O, and brine, dried (Na_2SO_4), filtered, and concentrated *in vacuo.* The residue was purified by silica gel flash column chromatography (ϕ 4×13 cm, 25% AcOEt/hexane) to afford **S18** (650 mg, 1.7 mmol, 70% over 3 steps) as a colorless oil. ^{1}H NMR ($CDCl_3$, 400 MHz) δ 7.70 (m, 2H, Ar), 7.20-7.45 (m, 11H, Ar), 5.58 (dt, 1H, H-4, $J_{4,3}$ = 15.6, $J_{4,5}$ = 5.5 Hz), 5.43 (dd, 1H, H-3, $J_{3,4}$ = 15.6, $J_{3,2}$ = 6.4 Hz), 3.93 (m, 2H, H-5), 3.43 (s, 3H, Me), 3.36 (d, 1H, H-2, $J_{2,3}$ = 6.9 Hz). This is a known compound. [S1)]

(*S*,*E*)-5-Hydroxy-2-[(9-phenyl-9H-fluoren-9-yl)amino]pent-3-enoic acid (16)

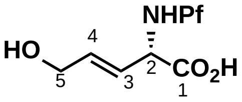

A solution of **S18** (190 mg, 0.50 mmol) in dioxane (4 mL) and H_2O (2 mL) was treated with $LiOH \cdot H_2O$ (130 mg, 3.0 mmol) for 2.5 h. The mixuture was partitioned between AcOEt and 1 M *aq*. HCl. The organic phase was washed with brine, dried (Na_2SO_4), filtered, and concentrated *in vacuo* to afford **16** (200 mg, quant.) as a yellow solid. ^{1}H NMR ($CDCl_3$, 400 MHz) δ 7.83 (m, 2H, Ar), 7.24-7.45 (m, 11H, Ar), 7.73 (m, 2H, Ar), 7.17-7.55 (m, 11H, Ar), 5.51 (m, 1H, H-4), 5.00 (d, 1H, H-3, $J_{3,4}$ = 12.4 Hz), 3.71 (m, 2H, H-5), 3.20 (d, 1H, H-2, $J_{2,3}$ = 7.8 Hz); $[\alpha]^{27}_{D}$ −73.8(*c* 1.01, $CHCl_3$). This is a known compound. [S1)]

(4*S*,2*E*)-4-{(2*S*,3*E*)-5-Hydroxy-2-[(9-phenyl-9H-fluoren-9-yl)amino]pent-3-enamido}-5-methyl-*N*-(*p*-toluenesulfonamido)hex-2-enamide (17)

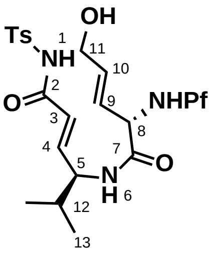

Compound **S11**[S2)] (150 mg, 0.38 mmol) was treated with 4 M HCl in dioxane (3.8 mL) at room temperature for 1 h. The mixture was concentrated *in vacuo* to give crude **15**. A solution of **15**, **16** (130 mg, 0.35 mmol), $^{i}Pr_2NEt$ (290 μL, 2.1 mmol), and HOAt (140 mg, 1.1 mmol) in DMF (3.5 mL) was treated with EDCI (160 mg, 1.1 mmol) at room temperature for 20 h. Additional EDCI (54 mg, 0.35 mmol), $^{i}Pr_2NEt$ (97 μL, 0.70 mmol), and HOAt (47 mg, 0.35 mmol) were added to the mixture, which was stirred for 2.5 h. The mixture was partitioned between AcOEt and *sat. aq.* $NaHCO_3$. The organic phase was washed with 1 M *aq.* HCl and brine, dried (Na_2SO_4), filtered, and concentrated *in vacuo*. The residue was purified by hi-flash silica gel column chromatography (ϕ 2×6.5 cm, 0→1% MeOH/$CHCl_3$) to afford **17** (130 mg, 0.20 mmol, 57% over 2 steps) as a colorless oil. ^{1}H NMR (CD_3OD, 500 MHz) δ 7.89 (m, 2H, Ar), 7.12-7.73 (m, 15H, Ar), 6.65 (dd, 1H, H-3, $J_{3,2}$ = 12.4, $J_{3,4}$ = 6.4 Hz), 5.78 (d, 1H, H-2, $J_{2,3}$ = 12.4 Hz), 5.51 (m, 2H, H-3', H-4'), 4.13 (m, 1H, H-4), 3.90-3.95 (m, 2H, H-5'), 3.13 (m, 1H, H-2'), 2.41 (s, 3H, Ts-Me), 1.66 (m, 1H, H-5), 0.76 (m, 6H, H-6); ^{13}C NMR (CD_3OD, 125 MHz) δ 187.9, 185.7, 172.1, 150.4, 145.9, 141.7, 133.1, 133.0, 132.9, 130.8, 130.5, 130.0, 129.6, 129.3, 128.8, 128.2, 127.1, 127.0, 126.5, 120.9, 74.3, 62.8, 62.7, 60.1, 57.4, 32.9, 21.4, 19.3, 14.8; ESIMS-LR *m/z* 650 [$(M+H)^+$]; ESIMS-HR calcd for $C_{38}H_{40}O_5N_3S$ 560.2683, found 560.2693; $[\alpha]^{28}_{D}$ −55.2 (*c* 0.99, MeOH).

(3*E*,5*S*,8*S*,9*E*)-5-Isopropyl-8-[(9-phenyl-9H-fluoren-9-yl)amino]-1,6-diazacycloundeca-3,9-diene-2,7-dione (18)

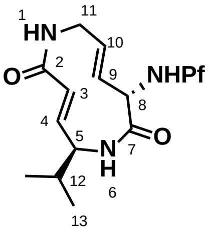

A solution of **17** (68 mg, 0.10 mmol) and PPh_3 (55 mg, 0.21 mmol) in THF (52 mL) was treated with DEAD (95 μL, 0.21 mmol) at room temperature for 1 h. The mixuture was concentrated *in vacuo*, and the residue was purified by hi-flash silica gel column chromatography (ϕ 2×6.5 cm, 20→40% AcOEt/hexane) to afford a crude sulfonamide (100 mg) as a white solid. A solution of the sulfonamide in THF (1 mL) was treated with SmI_2 (10 mL, 10 mmol, 1.0 M in THF) at room temperature for 10 min. The mixture was partitioned between AcOEt and *sat. aq.* NH_4Cl. The organic phase was washed with 1 M *aq.* HCl, *sat. aq.* $NaHCO_3$, H_2O, and brine, dried (Na_2SO_4), filtered, and concentrated *in vacuo*. The residue was purified by hi-flash silica gel column chromatography (ϕ 2×6.5 cm, 0→1% MeOH/$CHCl_3$) to afford **18** (25 mg, 52 μmol, 50% over 2 steps) as a white solid. ^{1}H NMR (CD_3OD, 500 MHz) δ 7.73 (m, 2H, Ar), 7.19-7.33 (m, 11H, Ar), 6.02 (m, 1H, H-4), 5.85 (d, 1H, H-3, $J_{3,4}$ = 16.1 Hz), 5.20 (m, 1H, H-9), 5.15 (m, 1H, H-10), 3.79 (m, 1H, H-5), 3.70 (dd, 1H, H-11, J_{gem} = 15.5, $J_{11,10}$ = 6.9 Hz), 3.64 (dd, 1H, H-11, J_{gem} = 15.5, $J_{11,10}$ = 4.0 Hz), 3.26 (d, 1H, H-8, $J_{8,9}$ = 8.0 Hz), 1.50 (m, 1H, H-12), 0.84 (d, 3H, H-13, $J_{13,12}$ = 6.3 Hz), 0.76 (d, 3H, H-13, $J_{13,12}$ = 6.3 Hz); ^{13}C NMR (CD_3OD, 125 MHz) δ 174.1, 173.4, 151.1, 150.1, 145.9, 142.0, 141.0, 134.3, 131.0, 129.6, 129.5, 129.3, 128.8, 128.1, 127.3, 127.1, 126.6, 120.9, 120.8, 74.0, 59.3, 45.7, 39.9, 39.4, 32.8, 19.9, 19.7; ESIMS-LR *m/z* 500 [$(M+Na)^+$]; ESIMS-HR calcd for $C_{31}H_{31}O_2N_3Na$ 500.2309, found 500.2319; $[\alpha]^{28}_{D}$ −26.2(*c* 1.12, MeOH).

***N*-{(*S*)-1-{[(3*E*,5*S*,8*S*,9*E*)-5-Isopropyl-2,7-dioxo-1,6-diazacycloundeca-3,9-dien-8-yl]amino}-1-oxo-3-phenylpropan-2-yl}decanamide (8)**

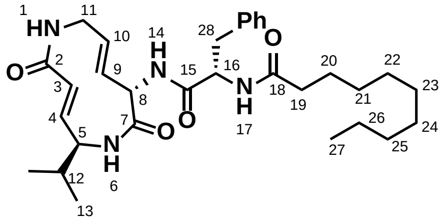

A solution of **18** (51 mg, 0.11 mmol) and Et_3SiH (0.73 mL, 0.27 mmol) in CH_2Cl_2 (1.1 mL) was treated with TFA (1.1 mL) at 0 °C, and the mixture was stirred at room temperature for 4 h. The mixture was concentrated *in vacuo*. A solution of the residue, *N*-decanoyl-L-phenylalanine (45 mg, 0.14 mmol), $^{i}Pr_2NEt$ (36 μL, 0.21 mmol), and HOAt (25 mg, 0.16 mmol) in DMF (1.0 mL) was treated with PyBOP (83 mg, 0.16 mmol) at room temperature for 20 h. Additional PyBOP (83 mg, 0.16 mmol), $^{i}Pr_2NEt$ (150 μL, 0.88 mmol), and HOAt (25 mg, 0.16 mmol) was added to the mixture, and the whole mixture was stirred for 14 h. The mixture was partitioned between AcOEt and H_2O. The organic phase was washed with 1 M *aq.* HCl, *sat. aq.* $NaHCO_3$, H_2O, and brine, dried (Na_2SO_4), filtered, and concentrated *in vacuo*. The residue was purified by hi-flash silica gel column chromatography (ϕ 1.6× 6 cm, 1% MeOH/$CHCl_3$) to afford **8** (130 mg, 0.20 mmol, 57% over 2 steps) as a white solid. ^{1}H NMR (DMSO-d_6, 500 MHz) δ 8.06 (m, 1H, H-14), 7.87 (d, 1H, H-17, $J_{17,16}$ = 8.0 Hz), 7.54 (br s, 1H, H-6), 7.41 (br s, 1H, H-1), 7.23 (m, 5H, Ar), 6.17 (dd, 1H, H-4, $J_{4,3}$ = 16, $J_{4,5}$ = 6.3 Hz), 5.99 (d, 1H, H-3, $J_{3,4}$ = 16 Hz), 5.60 (br s, 1H, H-10), 5.53 (br s, 1H, H-11), 4.79 (br s, 1H, H-8), 4.58 (m, 1H, H-16), 3.89 (m, 1H, H-5), 3.71 (m, 2H, H-11), 3.03 (dd, 1H, H-28, J_{gem} = 11.5, $J_{28,16}$ = 4.1 Hz), 2.74 (dd, 1H, H-28, J_{gem} = $J_{28,16}$ = 11.5 Hz), 2.00 (t, 2H, H-19, $J_{19,20}$ = 7.5 Hz), 1.73 (m, 1H, H-12), 1.09-1.37 (m, 14H, H-20, H-21, H-22, H-23, H-24, H-25, H-26), 0.88 (m, 9H, H-13, H-27); ^{13}C NMR (DMSO-d_6, 125 MHz) δ 171.9, 170.9, 169.0, 168.7, 138.0, 129.1, 127.9, 126.1, 53.5, 43.8, 37.3, 35.0, 31.3, 28.8, 28.7, 28.4, 25.2, 22.1,19.7, 19.3, 14.0; ESIMS-LR *m/z* 539 [$(M+Na)^+$]; ESIMS-HR calcd for $C_{31}H_{47}O_4N_4$ 539.3592, found 539.3595.

Scheme S4. Synthesis of **9**

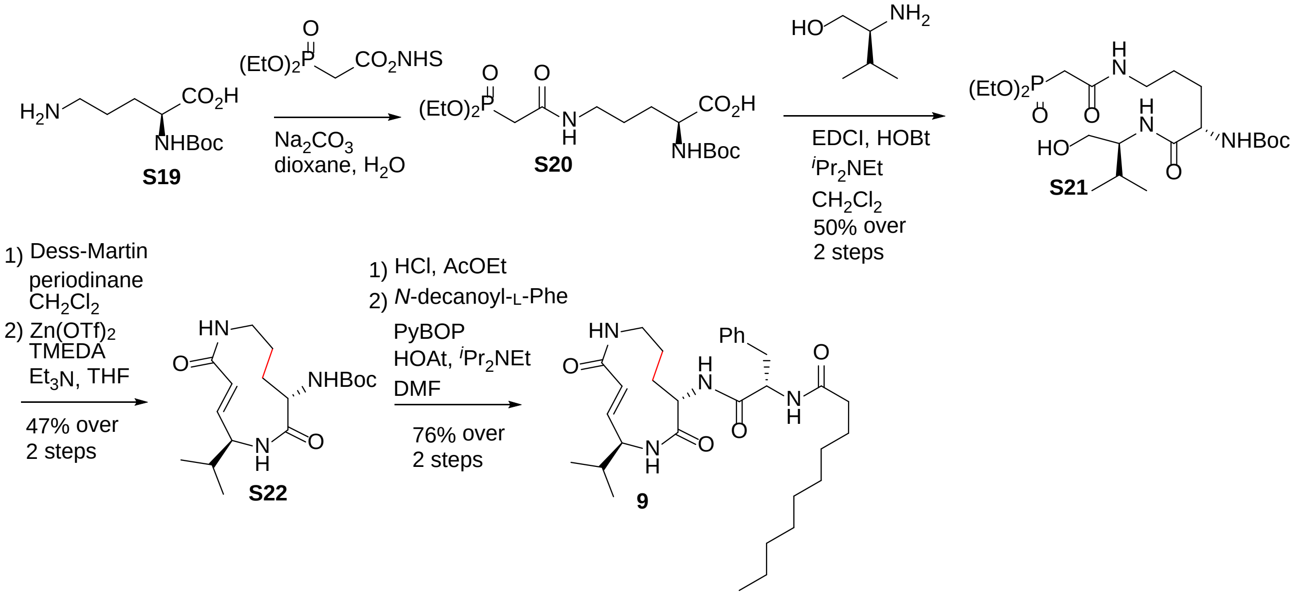

tert-Butyl {(*S*)-5-[2-(Diethoxyphosphoryl)acetamido]-1-{[(*S*)-1-hydroxy-3-methylbutan-2-yl]amino}-1-oxopentan-2-yl}carbamate (S21)

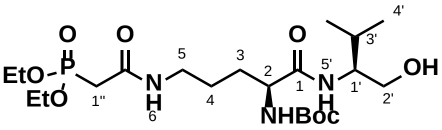

A mixture of **S19** (710 mg, 3.1 mmol) and Na_2CO_3 (640 mg, 6.1 mmol) in dioxane (3.0 mL) and H_2O (5 mL) was treated with a solution of $(EtO)_2P(=O)CH_2CO_2NHS$ (1.5 g, 6.1 mmol) in dioxane (2 mL) at 0 °C, and the mixture was stirred at room temperature for 23 h. The mixture was partitioned between Et_2O and *sat. aq.* $NaHCO_3$. The aqueous phase was acidified with 1 M *aq.* HCl, and extracted with AcOEt. The organic phase was washed with brine, dried (Na_2SO_4), filtered, and concentrated *in vacuo* to give **S20**. A solution of **S20**, L-valinol (670 μL, 6.0 mmol), HOBt·H_2O (810 mg, 6.0 mmol), and $^i Pr_2NEt$ (1.7 mL, 12 mmol) in DMF (20 mL) was treated with EDCI (930 mg, 6.0 mmol) at room temperature for 22 h. The mixture was partitioned between with AcOEt and H_2O. The organic phase was washed with 1 M *aq.* HCl, *sat. aq.* $NaHCO_3$, H_2O, and brine, dried (Na_2SO_4), filtered, and concentrated *in vacuo*. The residue was purified by silica gel column chromatography (ϕ 3 × 10 cm, 2% MeOH/$CHCl_3$) to afford **S21** (990 mg, 2.0 mmol, 50% over 3 steps) as a colorless oil. ^1H NMR ($CDCl_3$, 400 MHz) δ 7.14 (m, 1H, H-6), 7.03 (d, 1H, H-5', $J_{5',1'}$ = 8.3 Hz), 5.45 (d, 1H, BocN*H*, $J_{NH,2}$ = 8.2 Hz), 4.37 (m, 1H, H-2), 4.15 (m, 4H, CH_3CH_2O), 3.80 (m, 2H, H-2'), 3.65 (m, 4H, O*H*, H-1', H-2'), 3.47 (m, 1H, H-5), 3.27 (m, 1H, H-5), 2.91 (d, 1H, H-1", *J* = 15.1 Hz), 2.85 (d, 1H, H-1", $J_{1'',P}$ = 15.6 Hz), 1.88 (m, 1H, H-3'), 1.69 (m, 2H, H-3), 1.59 (m, 2H, H-4), 1.47 (s, 9H, ^{t}Bu), 1.32 (m, 6H, CH_3CH_2O), 0.94 (m, 6H H-4'); ^{13}C NMR ($CDCl_3$, 100 MHz) δ 172.6, 164.6, 156.2, 79.9, 63.6, 63.2, 57.9, 57.2, 53.5, 39.1, 36.1, 34.8, 30.7, 29.2, 29.0, 28.4, 24.4, 19.6, 19.2, 18.8, 16.5, 16.4; ESIMS-LR *m/z* 518 $[(M+Na)^+]$; ESIMS-HR calcd for $C_{21}H_{42}O_8N_3NaP$ 518.2602, found 518.2600. $[\alpha]^{20}_D$ −14.4 (*c* 1.27, $CHCl_3$).

tert-Butyl [(5*S*,8*S*,*E*)-5-Isopropyl-2,7-dioxo-1,6-diazacycloundec-3-en-8-yl]carbamate (S22)

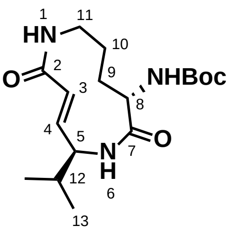

A solution of **S21** (970 mg, 2.0 mmol) in CH_2Cl_2 (20 mL) was treated with Dess-Martin periodinane (910 mg, 2.2 mmol) at room temperature for 1 h. Ethyl acetate, *sat. aq.* $NaHCO_3$, and *sat. aq.* $Na_2S_2O_3$ were added to the mixture, which was stirred for further 10 min. The organic phase was dried (Na_2SO_4), filtered, and concentrated *in vacuo* to afford a crude aldehyde. A solution of $Zn(OTf)_2$ (1.6 g, 4.3 mmol), TMEDA (350 μL, 2.4 mmol) in THF (260 mL) was treated with Et_3N (1.1 mL, 7.8 mmol) at room temperature for 15 min. A solution of the crude aldehyde in THF (130 mL) was added to the mixture, which was stirred for further 19 h. The mixture was concentrated *in vacuo*, and the residue was partitioned between AcOEt and 1 M *aq.* HCl. The organic phase was washed with brine, dried (Na_2SO_4), filtered, and concentrated *in vacuo*. The residue was purified by silica gel column chromatography (ϕ 3×11 cm, 1→3% MeOH/$CHCl_3$) to afford **S22** (310 mg, 0.91 mmol, 47% over 2 steps) as a white solid. ^{1}H NMR (CD_3OD, 500 MHz) δ 6.43 (d, 1H, H-3, $J_{3,4}$ = 16.6 Hz), 6.00 (dd, 1H, H-4, $J_{4,3}$ = 16.6, $J_{4,5}$ = 9.2 Hz), 4.11 (dd, 1H, H-5, $J_{5,4}$ = 9.2, $J_{5.12}$ = 9.8 Hz), 4.00 (dd, 1H, H-8, J = 5.2, J = 4.6 Hz), 3.15 (ddd, 1H, H-11, $J_{11,10}$ = 11.5, $J_{11,NH}$ = 3.5, J_{gem} = 14.9 Hz), 2.92 (ddd, 1H, H-11, $J_{11,10}$ = 8.1, $J_{11,NH}$ = 6.3, J_{gem} = 14.4 Hz), 1.82 (m, 1H, H-12), 1.81 (m, 1H, H-9), 1.66 (m, 1H, H-9), 1.43 (s, 9H, ^{t}Bu), 1.34 (m, 2H, H-10), 1.04 (d, 3H, H-13, $J_{13,12}$ = 6.3 Hz), 0.98 (d, 3H, H-13, $J_{13,12}$ = 6.3 Hz); ^{13}C NMR (CD_3OD, 100 MHz) δ 176.1, 174.1, 157.1, 141.8, 125.5, 80.4, 60.6, 55.4, 44.2, 31.6, 29.5, 28.7, 24.9, 20.0, 19.8; ESIMS-LR *m/z* 362 [(M+Na)$^+$]; ESIMS-HR calcd for $C_{17}H_{29}O_4N_3Na$ 362.2050, found 362.2059. $[\alpha]^{20}_D$ −124.2 (*c* 0.81, MeOH).

***N*-{(*S*)-1-{[(5*S*,8*S*,*E*)-5-Isopropyl-2,7-dioxo-1,6-diazacycloundec-3-en-8-yl]amino}-1-oxo-3-phenylpropan-2-yl}decanamide (9)**

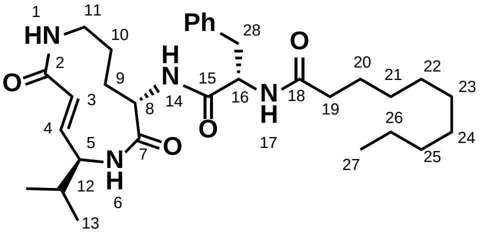

A solution of **S22** (37 mg, 0.11 mmol) was treated with 4 M HCl in AcOEt (1 mL) at room temperature for 1 h. The mixture was concentrated *in vacuo*. A solution of the residue, *N*-decanoyl-L-phenylalanine (39 mg, 120 μmol), ^{i}Pr$_2$NEt (56 μL, 330 μmol), and HOAt (26 mg, 160 μmol) in DMF (1.2 mL) was treated with PyBOP (86 mg, 164 μmol) at 0 °C, and the mixture was stirred at room temperature for 15 h. The mixture was partitioned between AcOEt and 1 M *aq.* HCl. The mixture was filtered, and the insoluble contents was washed with *sat. aq.* $NaHCO_3$, H_2O, and AcOEt. The residue was purified by preparative-TLC (10% MeOH/$CHCl_3$) to afford **9** (45 mg, 83 μmol, 76% over 2 steps) as a white solid. ^{1}H NMR (DMSO-d_6, 500 MHz) δ 8.42 (d, 1H, H-6, $J_{6,5}$ = 9.2 Hz), 7.96 (d, 1H, H-14, $J_{14,8}$ = 6.9 Hz), 7.93 (d, 1H, H-17, $J_{17,16}$ = 8.1 Hz), 7.37 (m, 1H, H-11), 7.14-7.20 (m, 5H, Ar), 6.29 (d, 1H, H-3, $J_{3,4}$ = 16 Hz), 5.84 (dd, 1H, H-4, $J_{4,3}$ = 16, $J_{4,5}$ = 8.6 Hz), 4.51 (m, 1H, H-16), 4.26 (m, 1H, H-8), 4.00 (ddd, 1H, H-5, $J_{5,4}$ = 8.6, $J_{5,6}$ = $J_{5,12}$ = 9.2 Hz), 3.00 (m, 1H, H-11), 2.93 (m, 1H, H-28), 2.77 (m, 1H, H-11), 2.69 (dd, 1H, H-28, $J_{28,16}$ = 10.9, J_{gem} = 12.6 Hz), 1.98 (t, 2H, H-19, $J_{19,20}$ = 6.3 Hz), 1.76 (m, 1H, H-9), 1.73 (m, 1H, H-12), 0.84-1.43 (m, 27H, H-9, H-10, H-13, H-20, H-21, H-22, H-23, H-24, H-25, H-26, H-27); ^{13}C NMR (DMSO-d_6, 125 MHz) δ 172.7, 172.1, 170.7, 170.0, 138.9, 137.9, 129.2, 127.9, 126.1, 125.2, 58.4, 53.5, 52.2, 42.5, 37.5, 35.2, 31.3, 29.8, 28.9, 28.8, 28.7, 28.4, 25.2, 25.2, 22.1, 19.6, 19.3, 14.0; ESIMS-LR *m/z* 563 [(M+Na)$^+$]; ESIMS-HR calcd for $C_{31}H_{48}O_4N_4Na$ 563.3568, found 563.3575.

Scheme S5. Synthesis of **10**

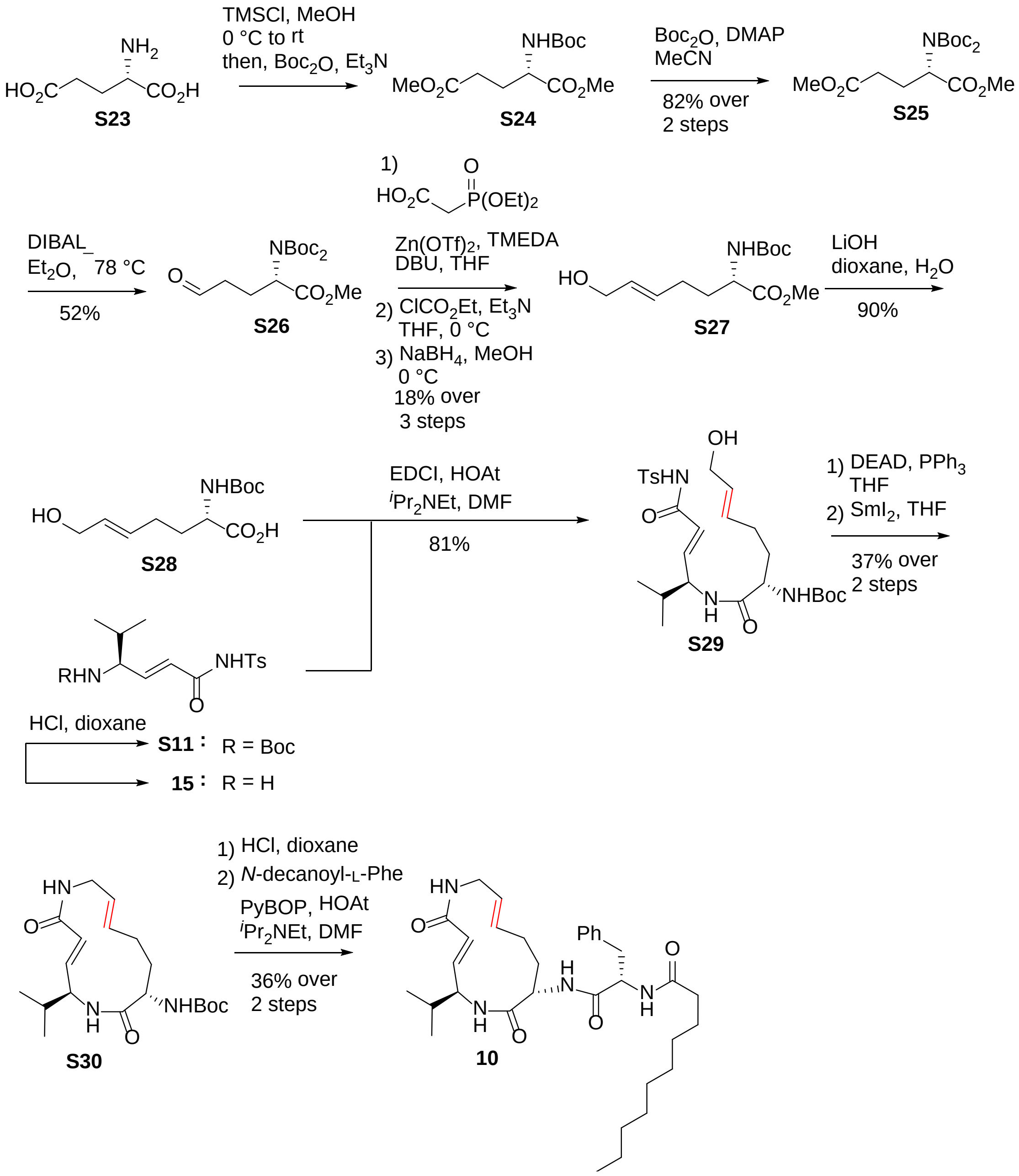

Methyl (*S*)-4-Methoxycarbonyl-2-(di-*tert*-butoxycarbonylamino)butanoate (S25)

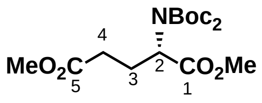

A solution of **S23** (2.9 g, 20 mmol) in MeOH (50 mL) was treated with TMSCl (11 mL, 88 mmol) at 0 °C, the mixture was stirred at room temperature for 19 h. Triethylamine (18 mL, 130 mmol) and Boc_2O (5.1 mL, 22 mmol) were added to the mixture at 0 °C. After stirring for 18 h at room temperature, the mixture was filtered, and partitioned between AcOEt and H_2O. The organic phase was washed with 1 M *aq*. HCl, *sat. aq*. $NaHCO_3$, H_2O, and brine, dried (Na_2SO_4), filtered, and concentrated *in vacuo* to give **S24**. A solution of **S24** and DMAP (480 mg, 4.0 mmol) in MeCN (58 mL) was treated with Boc_2O (5.6 mL, 22 mmol) at room temperature for 14 h. The mixture was partitioned between AcOEt and H_2O. The organic

phase was washed with 1 M *aq.* HCl, *sat. aq.* $NaHCO_3$, H_2O, and brine, dried (Na_2SO_4), filtered, and concentrated *in vacuo*. The residue was purified by hi-flash silica gel column chromatography (ϕ 4.6 × 12 cm, 10→30% AcOEt/hexane) to afford **S25** (6.2 g, 16 mmol, 82% over 2 steps) as a yellow oil. ^1H NMR ($CDCl_3$, 400 MHz) δ 4.95 (dd, 1H, H-2, $J_{2,3a}$ = 9.6, $J_{2,3b}$ =4.1 Hz), 3.73 (s, 3H, CO_2Me), 3.69 (s, 3H, CO_2Me), 2.46 (m, 2H, H-4), 2.41 (m, 1H, H-3), 2.18 (m, 1H, H-3), 1.52 (s, 18H, *^t^*Bu). This is a known compound. [S5)]

Methyl 2-di-*tert*-butoxycarbonylamino-5-oxo-pentanoate (S26)

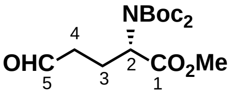

A solution of **S25** (6.2 g, 16 mmol) in Et_2O (150 mL) was treated with a solution of DIBAL (25 mL, 25 mmol, 1.0 M in toluene) in Et_2O (10 mL) at −78 °C for 1 h. Diisobutylaluminium hydride (4.9 mL) was further added to the mixture, which was stirred for 20 min. The reaction was quenched with $Na_2SO_4 \cdot 10\ H_2O$, and the mixture was stirred for 15 min, filtered, and the filtrate was concentrated *in vacuo*. The residue was purified by silica gel column chromatography (18→25% AcOEt/hexane) to afford **S26** (2.9 g, 8.5 mmol 52%) as a colorless oil. ^1H NMR ($CDCl_3$, 400 MHz) δ 9.76 (s, 1H, C*H*O), 4.87 (dd, 1H, H-2, $J_{2,3a}$ = 9.2, $J_{2,3b}$ = 4.6 Hz), 3.71 (s, 3H, CO_2Me), 2.58 (m, 1H, H-3), 2.48 (m, 2H, H-4), 2.16 (m, 1H, H-3), 1.49 (s, 18 H, ^{t}Bu). This is a known compound. [S5)]

Methyl (*S*,*E*)-2-[(*tert*-Butoxycarbonyl)amino]-7-hydroxyhept-5-enoate (S27)

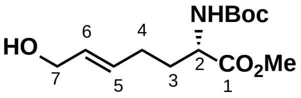

A solution of diethylphosphonoacetic acid (1.7 g, 4.9 mmol), $Zn(OTf)_2$ (3.2 g, 8.9 mmol), TMEDA (0.72 mL, 4.9 mmol), and DBU (2.4 mL, 16 mmol) in THF (31 mL) was treated with a solution of **S26** (1.4 g, 4.1 mmol) in THF (10 mL) at room temperature for 16 h. The reaction was quenched with 1 M *aq.* HCl, and partitioned between AcOEt and H_2O. The organic phase was washed with brine, dried (Na_2SO_4), filtered, and concentrated *in vacuo*. A solution of the residue and Et_3N (1.1 mL, 8.1 mmol) in THF (41 mL) was treated with ethyl chloroformate (770 μL, 8.1 mmol) at 0 °C for 15 min. A solution of $NaBH_4$ (1.5 g, 41 mmol) in MeOH (13 mL) was added to the mixture, which was stirred for 2 h. The reaction was quenched with 1 M *aq.* HCl, and the mixture was partitioned between AcOEt and H_2O. The organic phase was washed with brine, dried (Na_2SO_4), filtered, and concentrated *in vacuo*. The residue was purified by silica gel column chromatography (ϕ 3×10 cm, 50% AcOEt/hexane) to afford **S27** (200 mg, 0.73 mmol 18% over 3 steps) as a colorless oil. ^1H NMR (CD_3OD, 500 MHz) δ 5.65 (m, 2H, H-5, H-6), 4.11 (m, 1H, H-2), 4.00 (d, 2H, H-7, $J_{7,6}$ = 4.1 Hz), 3.71 (s, 3H, CO_2Me), 2.13 (m, 2H, H-4), 1.85 (m, 1H, H-3), 1.71 (m, 1H, H-3), 1.44 (s, 9H, ^{t}Bu); ^{13}C NMR (CD_3OD, 125 MHz) δ 174.6, 158.2, 131.9, 131.2, 80.6, 63.5, 54.4, 52.6, 32.2, 29.5, 28.7; ESIMS-LR *m*/*z* 296 [$(M+Na)^+$]; ESIMS-HR calcd for $C_{13}H_{23}O_5NNa$ 296.1468, found 296.1471. $[\alpha]^{19}_D$ −16.2 (*c* 0.42, MeOH).

(*S*,*E*)-2-[(*tert*-Butoxycarbonyl)amino]-7-hydroxyhept-5-enoic acid (S28)

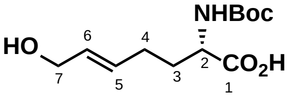

A solution of **S27** in dioxane (4 mL) and H_2O (2 mL) was treated with $LiOH \cdot H_2O$ (160 mg, 3.7 mmol) at room temperature for 3 h. The mixture was partitioned between AcOEt and 1M *aq.* HCl. The organic phase was washed with brine, dried (Na_2SO_4), filtered, and concentrated *in vacuo* to afford **S28** (150 mg, 0.56 mmol, 90%) as a colorless oil. ^1H NMR (CD_3OD, 400 MHz) δ 5.66 (m, 2H, H-5, H-6), 4.07 (m, 1H, H-2), 4.01 (m, 2H, H-7), 2.14 (m, 2H, H-4), 1.87 (m, 1H, H-3), 1.69 (m, 1H,

H-3), 1.48 (s, 9H, ^{t}Bu); ^{13}C NMR (CD_3OD, 100 MHz) δ 131.7, 131.4, 101.0, 80.4, 63.6, 62.8, 32.4, 29.6, 28.7; ESIMS-LR (negative mode) *m/z* 258 [(M-H)$^-$]; ESIMS-HR calcd for $C_{12}H_{20}O_5N$ 258.1347, found 128.1349. $[\alpha]^{19}_D$ −5.33 (*c* 0.30, MeOH).

***tert*-Butyl {(2*S*,5*E*)-7-Hydroxy-1-{{(3*S*,4*E*)-2-methyl-6-[(4-methylphenyl)sulfonamide]-6-oxohex-4-en-3-yl}amino}-1-oxohept-5-en-2-yl}carbamate (S29)**

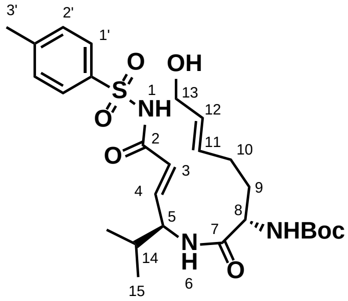

Compound **S11**[S2)] (320 mg, 0.80 mmol) was treated with 4 M HCl in dioxane (8.0 mL) at room temperature for 30 min. The mixture was concentrated *in vacuo* to give **15**. A solution of **15**, **S28** (150 mg, 0.53 mmol), ^{i}Pr$_2$NEt (450 μL, 3.2 mmol), and HOAt (220 mg, 1.6 mmol) in DMF (5.3 mL) was treated with EDCI (250 mg, 1.6 mmol) at room temperature for 21 h. The mixture was partitioned between AcOEt and 1 M *aq.* HCl. The organic phase was washed with 1 M *aq.* HCl, *sat. aq.* $NaHCO_3$, H_2O, and brine, dried (Na_2SO_4), filtered, and concentrated *in vacuo*. The residue was purified by hi-flash silica gel column chromatography (ϕ 2.6×10 cm, 0→1→2% MeOH/$CHCl_3$) to afford **S29** (230 mg, 0.43 mmol, 81% over 2 steps) as a colorless oil. ^{1}H NMR (CD_3OD, 500 MHz) δ 7.92 (d, 1, H-6, $J_{6,5}$ = 8.1 Hz), 7.88 (d, 2H, H-1', $J_{1',2'}$ = 6.4 Hz), 7.38 (d, 2H, H-2', $J_{2',1'}$ = 6.4 Hz), 6.78 (dd, 1H, H-4, $J_{4,3}$ = 15.5, $J_{4,5}$ = 6.9 Hz), 5.93 (d, 1H, H-3 $J_{3,4}$ = 15.5 Hz), 5.64 (dt, 1H, H-11, $J_{11,12}$ = 15.5, $J_{11,10}$ = 5.7 Hz), 5.61 (dt, 1H, H-12, $J_{12,11}$ = 15.5, $J_{12,13}$ = 5.7 Hz), 4.25 (m, 1H, H-5), 4.00 (m, 1H, H-8), 3.99 (m, 2H, H-13), 2.43 (s, 3H, H-3'), 2.08 (m, 2H, H-10), 1.85 (m, 1H, H-14), 1.67-1.82 (m, 2H, H-9), 1.42 (s, 9H, ^{t}Bu), 0.89 (t, 6H, H-15, $J_{15,14}$ = 8.6 Hz); ^{13}C NMR (CD_3OD, 100 MHz) δ 175.3, 165.8, 158.3, 148.5, 146.4, 138.4, 132.0, 130.9, 129.6, 124.2, 81.1, 80.0, 79.6, 79.3, 63.9, 57.7, 56.0, 33.7, 33.6, 33.1, 30.0, 29.1, 26.9, 22.0, 19.9, 19.2; ESIMS-LR *m/z* 560 [(M+Na)$^+$]; ESIMS-HR calcd for $C_{26}H_{39}O_7N_3NaS$ 560.2401, found 560.2405. $[\alpha]^{20}_D$ −9.3 (*c* 0.13, MeOH).

***tert*-Butyl [(3*E*,5*S*,8*S*,11*E*)-5-Isopropyl-2,7-dioxo-1,6-diazacyclotrideca-3,11-dien-8-yl]carbamate (S30)**

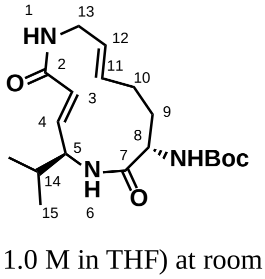

A solution of **S29** (200 mg, 0.37 mmol) and PPh_3 (200 mg, 0.74 mmol) in THF (180 mL) was treated with DEAD (340 μL, 0.74 mmol) for 30 min. The mixuture was concentrated *in vacuo*. The residue was purified by hi-flash silica gel column chromatography (ϕ 2.6×10 cm, 20→40% AcOEt/hexane) to afford a crude sulfonamide (220 mg) as a white solid. A solution of the crude sulfonamide in THF (3.7 mL) was treated with SmI_2 (10 mL, 1.0 mmol, 1.0 M in THF) at room temperature for 10 min. The mixture was partitioned between AcOEt and 1 M *aq.* HCl. The organic phase was washed with brine, dried (Na_2SO_4), filtered, and concentrated *in vacuo*. The residue was purified by hi-flash silica gel column chromatography (ϕ 2×6.5 cm, 1% MeOH/$CHCl_3$) to afford **S30** (50 mg, 140 μmol, 37% over 2 steps) as a white solid. ^{1}H NMR (CD_3OD, 500 MHz) δ 6.48 (m, 2H, H-3, H-4), 5.50 (m, 1H, H-11), 5.34 (m, 1H, H-12), 4.25 (dd, 1H, H-5, J = 6.3, J = 2.9 Hz), 4.10 (d, 1H, H-8, J = 5.7 Hz), 3.75 (dd, 1H, H-13, J_{gem} = 16.6, $J_{13,12}$ = 8.1 Hz), 3.64 (d, 1H, H-13, J_{gem} = 16.6 Hz), 2.32 (m, 1H, H-10), 2.12 (m, 1H, H-10), 2.09 (m, 1H, H-9), 1.85 (m, 1H, H-14), 1.74 (m, 1H, H-9), 1.44 (s, 9H, ^{t}Bu), 0.94 (m, 6H, H-15); ^{13}C NMR (CD_3OD, 125 MHz) δ 173.5, 172.5, 157.3, 145.5, 143.6, 134.1, 130.2, 126.1, 122.8, 80.5, 57.6, 57.3, 55.0, 45.3, 32.7, 32.6, 28.7, 28.4, 19.8, 19.3;

ESIMS-LR *m/z* 388 [(M+Na)$^+$]; ESIMS-HR calcd for $C_{19}H_{31}O_4N_3Na$ 388.2207, found 388.2212. $[\alpha]^{20}_{D}$ −52.3 (*c* 0.69, MeOH).

N-{(S)-1-{[(3E,5S,8S,11E)-5-Isopropyl-2,7-dioxo-1,6-diazacyclotrideca-3,11-dien-8-yl]amino}-1-oxo-3-phenylpropan-2-yl}decanamide (10)

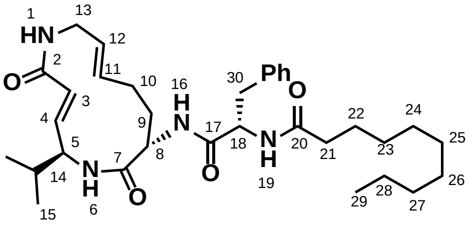

A solution of **S30** (25 mg, 68 μmol) was treated with 4 M HCl in dioxane (0.68 mL) at room temperature for 20 min. The mixture was concentrated *in vacuo*. A solution of the residue, *N*-decanoyl-L-phenylalanine (43 mg, 140 μmol), $^{i}Pr_2NEt$ (46 μL, 270 μmol), and HOAt (21 mg, 140 μmol) in DMF (0.68 mL) was treated with PyBOP (71 mg, 140 μmol) at room temperature for 24 h. The mixture was partitioned between AcOEt and 1 M *aq.* HCl. The mixture was filtered, and the insoluble contents was washed with *sat. aq.* $NaHCO_3$, H_2O, and AcOEt. The residue was purified by hi-flash silica gel column chromatography (ϕ 1.6×6 cm, 10% MeOH/$CHCl_3$) to afford **10** (14 mg, 250 μmol, 36% over 2 steps) as a white solid. ^{1}H NMR (DMSO-d_6, 500 MHz) δ 8.06 (d, 1H, H-6, $J_{6,5}$ = 9.2 Hz), 8.06 (d, 1H, H-19, $J_{19,18}$ = 9.2 Hz), 7.96 (d, 1H, H-16, $J_{16,8}$ = 7.5 Hz), 7.42 (m, 1H, H-1), 7.23 (m, 5H, Ar), 6.36 (d, 1H, H-3, $J_{3,4}$ = 15.5 Hz), 6.28 (dd, 1H, H-4, $J_{4,3}$ = 15.5, $J_{4,5}$ = 6.3 Hz), 5.50 (dt, 1H, H-11, $J_{11,12}$ = 15.5, $J_{11,10}$ = 6.9 Hz), 5.25 (dt, 1H, H-12, $J_{12, 11}$ = 14.9, $J_{12,13}$ = 5.8 Hz), 4.54 (ddd, 1H, H-18, $J_{18,19}$ = 8.6, $J_{18,30}$ = 4.1 Hz), 4.36 (t, 1H, H-8, $J_{8,16}$ = 7.5 Hz), 4.18 (ddd, 1H, H-5, $J_{5,4}$ = 6.3, $J_{5,6}$ = $J_{5,14}$ = 8.0 Hz), 3.58 (ddd, 1H, H-13, J_{gem} = 16, $J_{13,12}$ = 6.9, $J_{13,1}$ = 6.9 Hz), 3.48 (t, 1H, H-13, J_{gem} = 16 Hz), 2.98 (dd, 1H, H-30, J_{gem} = 13.8, $J_{30, 18}$ = 4.0 Hz), 2.72 (dd. 1H, H-30, J_{gem} = 13.8, $J_{30,18}$ = 4.0 Hz), 2.15 (m, 2H, H-10), 1.99 (m, 3H, H-9, H-10), 1.72 (m, 1H, H-14), 0.86-1.34 (m, 27H, H-14, H-15, H-21, H-22, H-23, H-24, H-25, H-26, H-27, H-28, H-29); ^{13}C NMR (DMSO-d_6, 125 MHz) δ 172.2, 170.7, 170.4, 168.7, 166.9, 142.6, 140.8, 138.0, 132.7, 129.1, 127.9, 126.1, 125.3, 122.1, 55.0, 53.7, 51.7, 51.4, 43.7, 35.2, 31.7, 31.3, 30.8, 28.8, 28.7, 28.4, 26.4, 25.2, 22.1, 19.2, 18.9, 13.9; ESIMS-LR *m/z* 589 [(M+Na)$^+$]; ESIMS-HR calcd for $C_{33}H_{50}O_4N_4Na$ 589.3724, found 589.3729.

Scheme S6. Synthesis of **11**

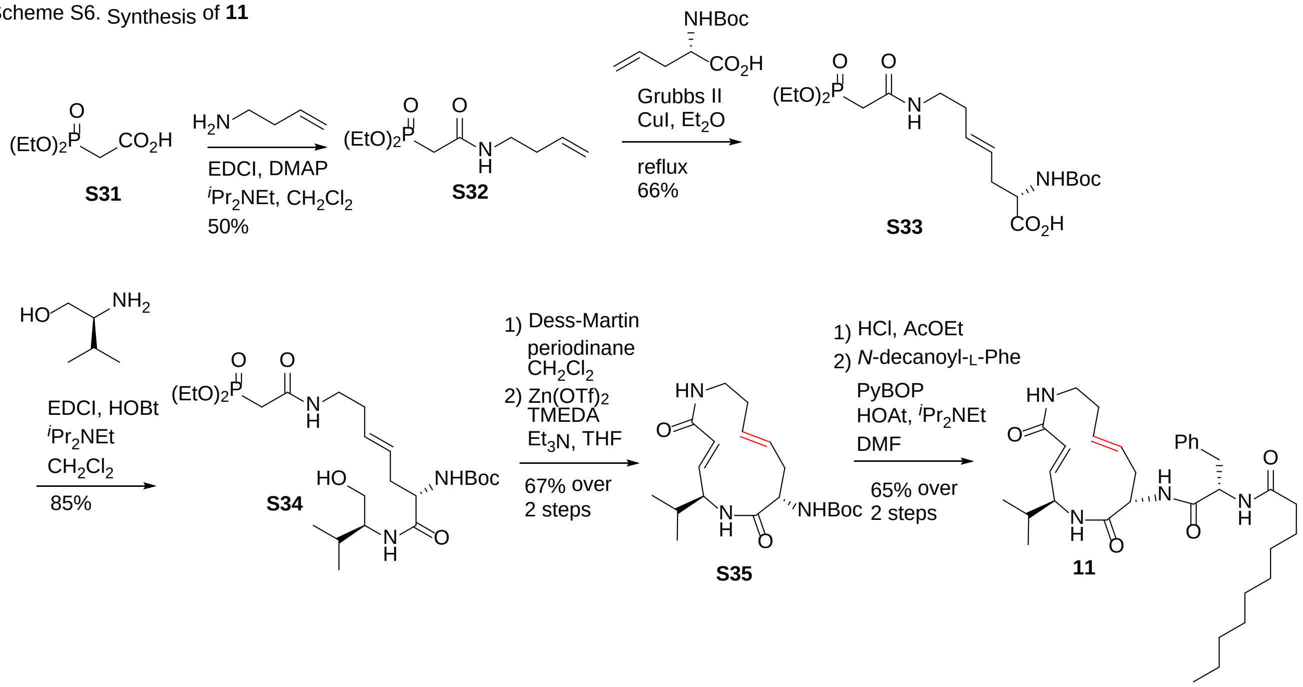

Diethyl [2-(But-3-en-1-ylamino)-2-oxoethyl]phosphonate (S32)

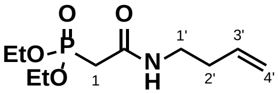

A solution of **S31** (1.6 g, 8.1 mmol), DMAP (300 mg, 2.4 mmol), homoallylamine hydrochloride (0.96 g, 8.9 mmol) and ^{i}Pr$_2$NEt (4.1 mL, 24 mmol) in CH_2Cl_2 (27 mL) was treated with EDCI (1.9 g, 12 mmol) at room temperature for 17 h. The mixture was partitioned between AcOEt and H_2O. The organic phase was washed with 1 M *aq.* HCl, *sat. aq.* $NaHCO_3$, H_2O, and brine, dried (Na_2SO_4), filtered, and concentrated *in vacuo* to afford **S32** (1.0 g, 4.0 mmol, 50%) as a colorless oil. ^{1}H NMR ($CDCl_3$, 400 MHz) δ 6.78 (br s, 1H, N*H*), 5.77 (ddt, 1H, H-3', $J_{3',4'b}$ = 17.4, $J_{3',4'a}$ = 10.6, $J_{3',2'}$ = 6.9 Hz), 5.12 (dd, 1H, H-4'b, $J_{4'b,3'}$ = 17.4, $J_{4'b,4'a}$ = 1.4 Hz), 5.10 (dd, 1H, H-4'a, $J_{4'a,3'}$ = 10.6, $J_{4'a,4'b}$ = 1.4 Hz), 4.14 (dt, 4H, CH_3CH_2O, $J_{CH_3CH_2O,P}$ = $J_{CH_3CH_2O,CH_3CH_2O}$= 7.4 Hz), 3.36 (t, 2H, H-1', $J_{1',2'}$ = 6.8 Hz), 2.86 (s, 1H, H-1), 2.80 (s, 1H, H-1), 2.26 (dt, 2H, H-2', $J_{2',3'}$ = $J_{2'1'}$ = 6.8 Hz), 1.34 (t, 6H, OCH_2CH_3, $J_{OCH_2CH_3, OCH_2CH_3}$ = 6.9 Hz); ^{13}C NMR ($CDCl_3$, 100 MHz) δ 164.0, 135.1, 117.4, 62.9, 62.8, 39.1, 35.8, 34.5, 33.7, 16.5; ESIMS-LR *m/z* 272 [(M+Na)$^+$]; ESIMS-HR calcd for $C_{10}H_{20}O_4NNaP$ 272.1022, found 272.1023.

(*S*,*E*)-2-(*tert*-Butoxycarbonyl)amino-7-[2-(diethoxyphosphoryl)acetamido]hept-4-enoic acid (S33)

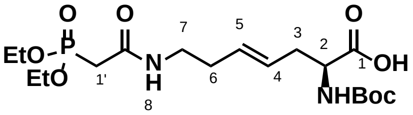

A solution of **S32** (830 mg, 3.3 mmol), Boc-allylGly-OH (790 mg, 3.7 mmol), and CuI (13 mg, 67 μmol) in Et_2O (33 mL) was treated with Grubbs II (85 mg, 0.10 mol) at room temperature, and the mixture was heated at under reflux temperature for 24 h[S6)]. The mixture was partitioned between Et_2O and *sat. aq.* $NaHCO_3$. The aqueous phase was acidified with 1 M *aq.* HCl and extracted with AcOEt. The organic phase was washed with brine, dried (Na_2SO_4), filtered, and concentrated *in vacuo*. The residue was purified by silica gel column chromatography (ϕ 3×12 cm, 10% MeOH/$CHCl_3$) to afford **S33** (970 mg, 2.2 mmol, 66%) as a colorless oil. ^{1}H NMR ($CDCl_3$, 400 MHz) δ 6.09 (br s, 1H, H-8), 5.56 (m, 1H, H-4), 5.41 (m, 1H, H-5), 5.35 (m, 1H, BocN*H*), 4.46 (m, 1H, H-2), 4.14 (m, 4H, CH_3CH_2O),

3.37 (m, 1H, H-7), 3.16 (m, 1H, H-7), 3.15 (dd, 1H, H-1', $J_{1',P}$ = 22.8, J_{gem} = 14.2 Hz), 2.84 (dd, 1H, H-1', $J_{1',P}$ = 22.8, J_{gem} = 14.2 Hz), 2.51 (m, 2H, H-3), 2.33 (m, 1H, H-6), 2.12 (m, 1H, H-6), 1.43 (s, 9H, ^{t}Bu), 1.31 (t, 6H, CH_3CH$_2$O, J = 6.8 Hz); ^{13}C NMR (CDCl$_3$, 400 MHz) δ 174.9, 164.0, 154.9, 131.1, 128.2, 110.4, 79.9, 63.3, 54.4, 38.7, 36.5, 31.0, 28.5, 16.4; ESIMS-LR *m/z* 459 [(M+Na)$^+$]; ESIMS-HR calcd for $C_{18}H_{33}O_8N_2NaP$ 459.1867, found 459.1869. $[\alpha]^{18}_D$ +105.9 (*c* 0.19, CHCl$_3$).

tert-Butyl {(*S*,*E*)-7-[2-(Diethoxyphosphoryl)acetamido]-1-{[(*S*)-1-hydroxy-3-methylbutan-2-yl]amino}-1-oxohept-4-en-2-yl}carbamate (S34)

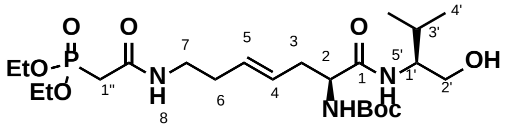

A solution of **S33** (370 mg, 0.84 mmol), L-valinol (140 μL, 1.3 mmol), HOBt·H$_2$O (170 mg, 1.3 mmol), ^{i}Pr$_2$NEt (350 μL, 2.5 mmol) in DMF (8.4 mL) was treated with EDCI (200 mg, 1.3 mmol) at room temperature for 24 h. *N*,*N*-Diisopropylethylamine (120 μL, 0.84 mmol), HOBt·H$_2$O (57 μg, 0.42 mmol) and EDCI (65 mg, 0.42 mmol) were further added to the mixture, which was stirred for further 11 h. The mixture was partitioned between with AcOEt and H$_2$O. The organic phase was washed with 1 M *aq.* HCl, *sat. aq.* NaHCO$_3$, H$_2$O, and brine, dried (Na$_2$SO$_4$), filtered, and concentrated *in vacuo*. The residue was purified by silica gel column chromatography (ϕ 4×12 cm, 0→2→3% MeOH/CHCl$_3$) to afford **S34** (370 mg, 0.71 mmol, 85%) as a colorless oil. ^{1}H NMR (CDCl$_3$, 400 MHz) δ 7.01 (br s, 1H, H-7), 6.83 (d, 1H, H-5', $J_{5',1'}$ = 9.2 Hz), 5.59 (d, 1H, BocNH, $J_{NH,1}$ = 6.9 Hz), 5.47 (m, 2H, H-4, H-5), 4.16 (m, 1H, H-2), 4.13 (m, 4H, CH$_3$CH_2O), 3.74 (m, 1H, H-1'), 3.68 (m, 2H, H-2'), 3.29 (m, 2H, H-7), 2.98 (d, 2H, H-1", $J_{1'',P}$ = 21.5 Hz), 2.49 (m, 2H, H-3), 2.42 (m, 1H, H-6), 2.20 (br s, 1H, H-6), 1.89 (m, 1H, H-3'), 1.43 (s, 9H, ^{t}Bu), 1.34 (t, 6H, CH_3CH$_2$O, J = 6.9 Hz), 0.94 (d, 6H, H-4', $J_{4',3'}$ = 6.9 Hz); ^{13}C NMR (CDCl$_3$, 100 MHz) δ 172.1, 164.1, 155.7, 130.6, 128.1, 63.5, 63.2, 62.9, 57.2, 54.9, 38.8, 36.3, 35.7, 34.4, 32.0, 29.0, 28.5, 19.7, 18.9, 16.5, 16.4; ESIMS-LR *m/z* 544 [(M+Na)$^+$]; ESIMS-HR calcd for $C_{23}H_{44}O_8N_3NaP$ 544.2758, found 544.2757. $[\alpha]^{19}_D$ −12.0 (*c* 0.88, CHCl$_3$).

tert-Butyl [(3*E*,5*S*,8*S*,10*E*)-5-Isopropyl-2,7-dioxo-1,6-diazacyclotrideca-3,10-dien-8-yl]carbamate (S35)

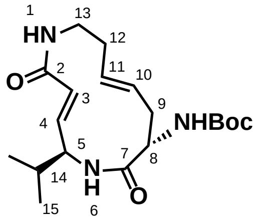

A solution of **S34** (310 mg, 0.59 mmol) in CH$_2$Cl$_2$ (5.9 mL) was treated with Dess-Martin periodinane (270 mg, 0.65 mmol) at room temperature for 1 h. Ethyl acetate, *sat. aq.* NaHCO$_3$ and *sat. aq.* Na$_2$S$_2$O$_3$ were added to the mixture, which was stirred for further 10 min. The organic phase was dried (Na$_2$SO$_4$), filtered, and concentrated *in vacuo* to afford a crude aldehyde. A solution of Zn(OTf)$_2$ (470 mg, 1.3 mmol), TMEDA (110 μL, 0.71 mmol) in THF (78 mL) was treated with Et$_3$N (330 μL, 2.4 mmol) at room temperature for 15 min. A solution of the crude aldehyde in THF (39 mL) was added to the mixture, which was further stirred for 20 h. The mixture was concentrated *in vacuo* and the residue was partitioned between AcOEt and 1 M *aq.* HCl. The organic phase was washed with brine, dried (Na$_2$SO$_4$), filtered, and concentrated *in vacuo*. The residue was purified by silica gel column chromatography (ϕ 3×10 cm, 1→3% MeOH/CHCl$_3$) to afford **S35** (150 mg, 0.40 mmol, 67% over 2 steps) as a white solid. ^{1}H NMR (CD$_3$OD, 500 MHz) δ 6.56 (dd, 1H, H-4, $J_{4,3}$ = 15.5, $J_{4,5}$ = 5.8 Hz), 6.40 (d, 1H, H-3, $J_{3,4}$ = 15.5 Hz), 5.43 (dt, 1H, H-10, $J_{10,11}$ = 15.5, $J_{10,9}$ = 7.4 Hz), 5.32 (dt, 1H, H-11, $J_{11,10}$ = 15.5, $J_{11,12}$ = 6.9 Hz), 4.05 (dd, 1H, H-5, $J_{5,4}$ = 5.8, $J_{5,14}$ = 6.3 Hz), 3.98 (dd, 1H, H-8, $J_{8,9}$ = 8.1, $J_{8,NH}$ = 2.9 Hz), 3.12 (m, 2H, H-13), 2.29 (ddd, 1H, H-9, $J_{9,10}$ = 7.5, $J_{9,8}$ =

8.0, J_{gem} = 14.3 Hz), 2.14 (m, 1H, H-9), 1.98 (m, 2H, H-12), 1.23 (s, 9H, ^{t}Bu), 0.80 (d, 6H, H-15, $J_{15,14}$ = 5.2 Hz); ^{13}C NMR (CD_3OD, 100 MHz) δ 174.0, 170.6, 157.3, 146.3, 133.0, 129.1, 123.5, 80.5, 57.8, 54.6, 44.2, 37.7, 35.5, 32.7, 30.7, 28.7, 19.7; ESIMS-LR *m*/*z* 388 [(M+Na)$^{+}$]; ESIMS-HR calcd for $C_{19}H_{31}O_4N_3Na$ 388.2207, found 388.2208.

***N*-{(*S*)-1-{[(3*E*,5*S*,8*S*,10*E*)-5-Isopropyl-2,7-dioxo-1,6-diazacyclotrideca-3,10-dien-8-yl]amino}-1-oxo-3-phenylpropan-2-yl}decanamide (11)**

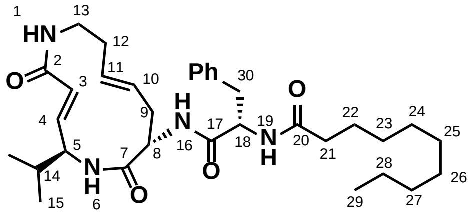

A solution of **S35** (40 mg, 0.11 mmol) was treated with 4 M HCl in AcOEt (1 mL) at room temperature for 1 h. The mixture was concentrated *in vacuo*. A solution of the residue, *N*-decanoyl-L-phenylalanine (38 mg, 120 μmol), $^{i}Pr_2NEt$ (56 μL, 330 μmol), and HOAt (25 mg, 160 μmol) in DMF (1.2 mL) was treated with PyBOP (85 mg, 160 μmol) at 0 °C, and the mixture was stirred at room temperature for 14 h. The mixture was partitioned between AcOEt and 1 M *aq.* HCl. The mixture was filtered, and the insoluble contents was washed with *sat. aq.* $NaHCO_3$, H_2O, and AcOEt. The residue was purified by preparative-TLC (10% MeOH/$CHCl_3$) to afford **11** (37 mg, 65 μmol, 65% over 2 steps) as a white solid. ^{1}H NMR (DMSO-d_6, 500 MHz) δ 8.24 (m, 1H, H-6), 8.02 (m, 2H, H-16, H-19), 7.39 (m, 1H, H-1), 7.15-7.22 (m, 5H, Ar), 6.61 (m, 1H, H-4), 6.51 (m, 1H, H-3), 5.58 (m, 1H, H-10), 5.42 (m, 1H, H-11), 4.52 (m, 1H, H-18), 4.43 (m, 1H, H-8), 4.19 (m, 1H, H-5), 3.13 (m, 2H, H-13), 2.98 (m, 1H, H-30), 2.74 (m, 1H, H-30), 2.39 (m, 1H, H-9), 2.28 (m, 1H, H-9), 2.09 (m, 1H, H-12), 2.00 (m, 2H, H-21), 1.75 (m, 1H, H-14), 0.84-1.34 (m, H-10, 11, 22-29); ^{13}C NMR (DMSO-d_6, 125 MHz) δ 171.2, 170.7, 167.4, 143.9, 142.4, 129.7, 128.7, 128.4, 126.8, 123.3, 55.7, 54.2, 52.1, 50.9, 42.8, 35.6, 32.0 31.2, 29.4, 29.2, 28.8, 25.7, 22.7, 19.8, 14.6; ESIMS-LR *m*/*z* 589 [(M+Na)$^{+}$]; ESIMS-HR calcd for $C_{33}H_{50}O_4N_4Na$ 589.3724, found 589.3727.

Scheme S7. Synthesis of **12**

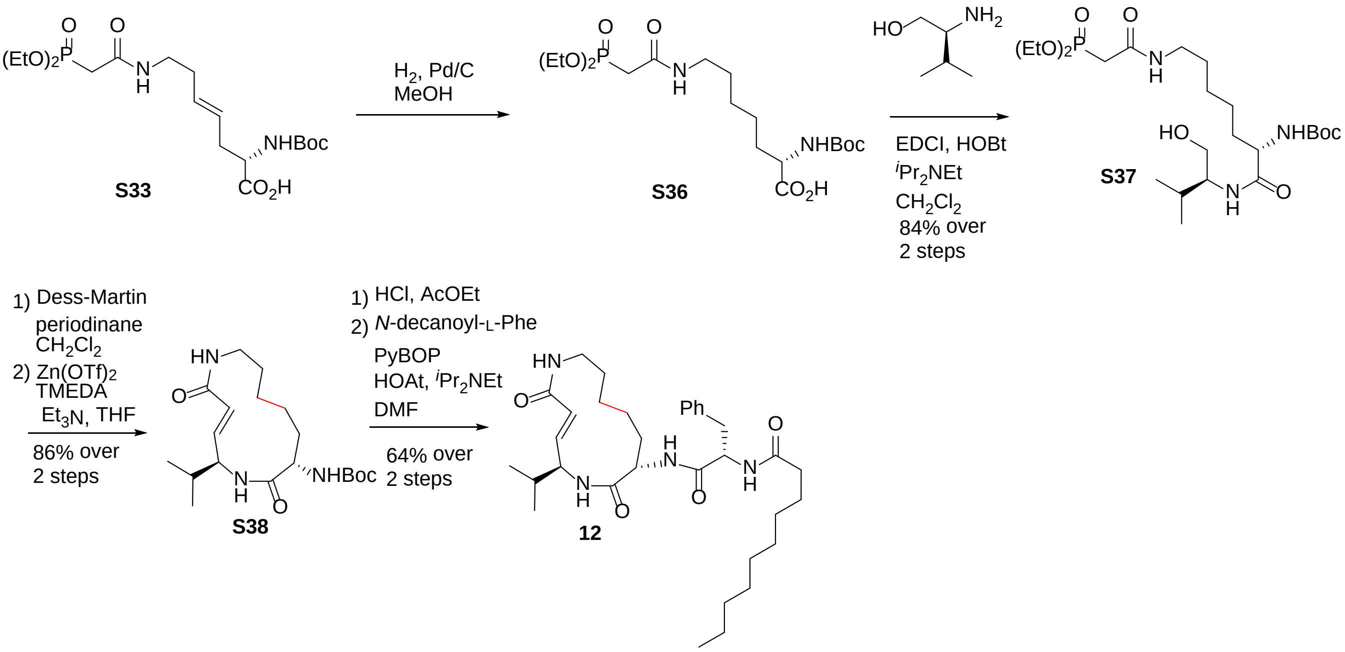

tert-Butyl {(*R*)-7-[2-(Diethoxyphosphoryl)acetamido]-1-{[(*S*)-1-hydroxy-3-methylbutan-2-yl]amino}-1-oxoheptan-2-yl}carbamate (S37)

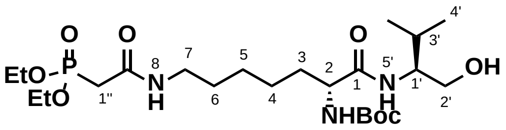

A mixture of **S33** (500 mg, 1.1 mmol) and Pd/C (110 mg, 0.10 mmol) in MeOH (8.0 mL) was stirred at room temperature under H_2 atmosphere for 1.5 h. The catalyst was filtered off through a Cerite pad, and the filtrate was concentrated *in vacuo* to give **S36**. A solution of **S36** (500 mg), L-valinol (250 μL, 2.3 mmol), HOBt·H_2O (310 mg, 2.3 mmol), and $^{i}Pr_2NEt$ (640 μL, 4.6 mmol) in DMF (10 mL) was treated with EDCI (350 mg, 2.3 mmol) at room temperature for 24 h. The mixture was partitioned between with AcOEt and H_2O. The organic phase was washed with 1 M *aq.* HCl, *sat. aq.* $NaHCO_3$, H_2O, and brine, dried (Na_2SO_4), filtered, and concentrated *in vacuo*. The residue was purified by hi-flash silica gel column chromatography (ϕ 2.6×10 cm, 2% MeOH/$CHCl_3$) to afford **S37** (500 mg, 0.96 mmol, 84%) as a colorless oil. ^{1}H NMR ($CDCl_3$, 400 MHz) δ 6.68 (m, 1H, H-8), 6.70 (d, 1H, H-5', $J_{5',1'}$ = 8.2 Hz), 5.21 (d, 1H, H-BocN*H*, $J_{NH,2}$ = 8.2 Hz), 4.16 (m, 1H, H-2), 4.12 (m, 4H, CH_3CH_2O), 3.68 (m, 3H, H-2', O*H*), 3.51 (m, 1H, H-1'), 3.28 (m, 2H, H-7), 2.85 (d, 2H, H-1", $J_{1'',P}$ = 18.3 Hz), 1.88 (m, 2H, H-3), 1.55 (m, 1H, H-3'), 1.35-1.45 (m, H-4, H-5, H-6, ^{t}Bu, H-4'), 0.95 (t, 6H, CH_3CH_2O); ^{13}C NMR ($CDCl_3$, 100 MHz) δ 172.6, 164.0, 145.2, 139.1, 92.7, 63.5, 62.7, 57.2, 54.7, 39.3, 29.1, 28.5, 24.4, 19.6, 19.1, 16.5; ESIMS-LR *m/z* 546 [$(M+Na)^+$]; ESIMS-HR calcd for $C_{23}H_{46}O_8N_3NaP$ 546.2915, found 546.2925. $[\alpha]^{19}_{D}$ −28.3 (*c* 0.36, $CHCl_3$).

tert-Butyl [(5*S*,8*S*,*E*)-5-Isopropyl-2,7-dioxo-1,6-diazacyclotridec-3-en-8-yl]carbamate (S38)

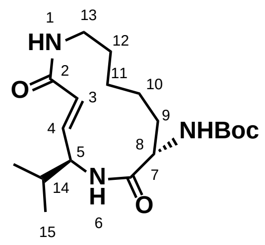

A solution of **S37** (170 mg, 0.32 mmol) in CH_2Cl_2 (3.2 mL) was treated with Dess-Martin periodinane (150 mg, 0.36 mmol) at room temperature for 1 h. Ethyl acetate, *sat. aq.* $NaHCO_3$, and *sat. aq.* $Na_2S_2O_3$ were added to the mixture, which was stirred for further 10 min. The organic phase was dried (Na_2SO_4), filtered, and concentrated *in vacuo* to afford a crude aldehyde. A solution of $Zn(OTf)_2$ (260 mg, 0.70 mmol), and TMEDA (57 μL, 0.38 mmol) in THF (44 mL) was treated with Et_3N (180 μL, 1.3 mmol) at room temperature for 15 min. A solution of the crude aldehyde in THF (20 mL) was added to the mixture, and the whole mixture was stirred for 15 h. The mixture was concentrated *in vacuo*, and the residue was partitioned between AcOEt and 1 M *aq.* HCl. The organic phase was washed with brine, dried (Na_2SO_4), filtered, and concentrated *in vacuo*. The residue was purified by silica gel column chromatography (ϕ 3 × 10 cm, 1→3% MeOH/$CHCl_3$) to afford **S38** (100 mg, 0.27 mmol, 86% over 2 steps) as a white solid. ^{1}H NMR (CD_3OD, 400 MHz) δ 6.73 (dd, 1H, H-4, $J_{4,3}$ = 15.6, $J_{4,5}$ = 4.6 Hz), 6.30 (d, 1H, H-3 $J_{3,4}$ = 15.6 Hz), 4.37 (dd, 1H, H-5, $J_{5,4}$ = $J_{5,14}$ = 5.5 Hz), 4.30 (m, 1H, H-8), 3.20 (ddd, 1H, H-13, $J_{13,12}$ = 6.4, $J_{13,\mathrm{N}H}$ = 8.7, J_{gem} = 15.1 Hz), 3.03 (ddd, 1H, H-13, $J_{13,12}$ = $J_{13,\mathrm{N}H}$ = 7.8, J_{gem} = 15.1 Hz), 1.90 (m, 1H, H-14), 1.81 (m, 2H, H-9), 1.43 (s, 9H, ^{t}Bu), 1.29-1.60 (m, 6H, H-10, H-11, H-12), 1.02 (t, 6H, H-15, $J_{15,14}$ = 5.5 Hz); ^{13}C NMR (CD_3OD, 100 MHz) δ 174.1, 170.9, 157.2, 145.4, 122.9, 80.5, 57.6, 54.9, 41.1, 32.8, 32.6, 29.5, 28.7, 24.9, 21.1, 19.7, 19.5; ESIMS-LR *m/z* 390 [(M+Na)$^+$]; ESIMS-HR calcd for $C_{19}H_{33}O_4N_3Na$ 390.2363, found 390.2368. $[\alpha]^{18}_{D}$ −58.2 (*c* 0.60, MeOH).

_N_-{(_S_)-1-{[(5_S_,8_S_,_E_)-5-isopropyl-2,7-dioxo-1,6-diazacyclotridec-3-en-8-yl]amino}-1-oxo-3-phenylpropan-2-yl}decanamide (12)

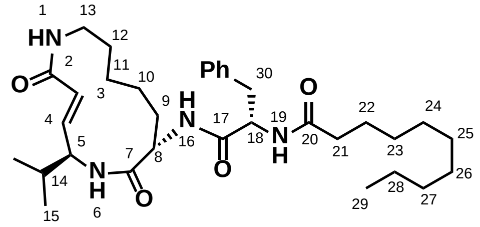

A solution of **S38** (40 mg, 0.11 mmol) was treated with 4.0 M HCl in AcOEt (1.0 mL) at room temperature for 1 h. The mixture was concentrated *in vacuo*. A solution of the residue, *N*-decanoyl-L-phenylalanine (39 mg, 120 μmol), ^{i}Pr$_2$NEt (56 μL, 330 μmol), and HOAt (26 mg, 160 μmol) in DMF (1.2 mL) was treated with PyBOP (86 mg, 160 μmol) at 0 °C, and the mixture was stirred at room temperature for 17 h. The mixture was partitioned between AcOEt and 1 M *aq.* HCl. The mixture was filtered, and the insoluble contents was washed with *sat. aq.* $NaHCO_3$, H_2O, and AcOEt. The residue was purified by preparative-TLC (10% MeOH/$CHCl_3$) to afford **12** (40 mg, 70 μmol, 64% over 2 steps) as a white solid. ^{1}H NMR (DMSO-d_6, 500 MHz) δ 8.13 (m, 2H, H-6, H-19), 7.70 (m, 1H, H-16), 7.44 (m, 1H, H-1), 7.15-7.23 (m, 5H, Ar), 6.55 (d, 1H, H-4, $J_{4,3}$ = 13.8 Hz), 6.13 (d, 1H, H-3, $J_{3,4}$ = 15.5 Hz), 4.51 (m, 2H, H-8, H-18), 4.28 (br s, 1H, H-5), 3.01 (m, 1H, H-30), 2.88 (m, 2H, H-13), 2.75 (m, 1H, H-30), 2.00 (br s, 2H, H-21), 1.81 (m, 2H, H-9), 1.77 (m, 1H, H-14), 0.85-1.47 (m, 29H, H-10, H-11, H-12, H-15, H-22, H-23, H-24, H-25, H-26, H-27, H-28, H-29); ^{13}C NMR (DMSO-d_6, 125 MHz) δ 172.3, 170.6, 166.9, 142.7, 138.2, 133.7, 129.1, 127.9, 126.1, 123.3, 122.1, 120.3, 95.6, 55.0, 53.7, 51.7, 37.0, 35.2, 31.3, 30.9, 28.9, 28.7, 28.4, 25.3, 23.4, 22.2, 19.4, 19.3, 18.8, 14.0; ESIMS-LR *m/z* 591 [(M+Na)$^+$]; ESIMS-HR calcd for $C_{33}H_{52}O_4N_4Na$ 591.3881, found 591.3893.

Scheme S8. Synthesis of **13**

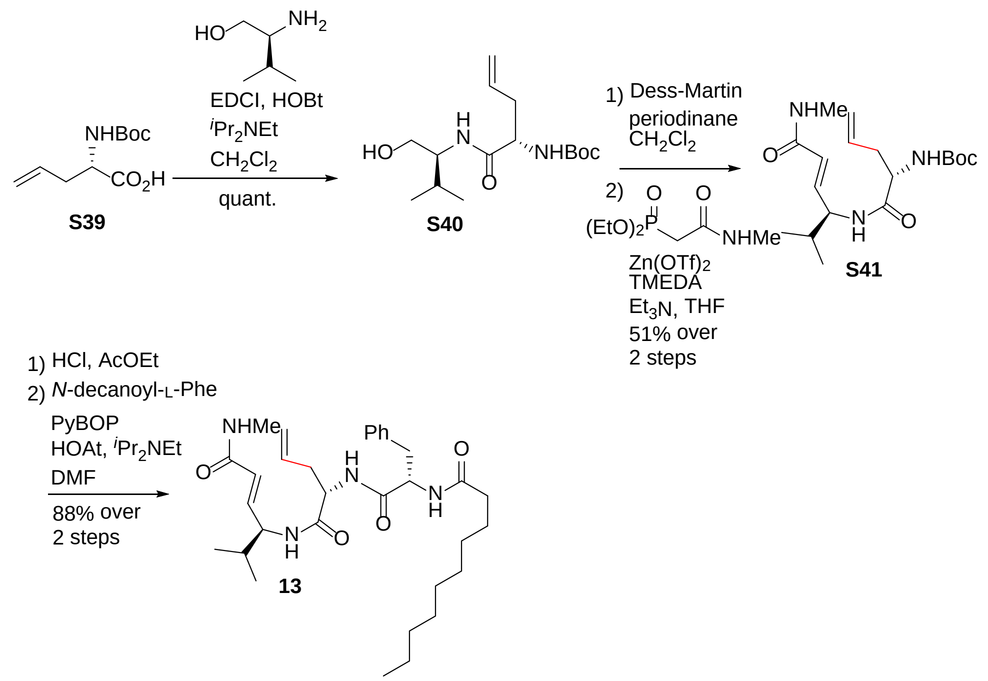

tert-Butyl {(_S_)-1-{[(_R_)-1-Hydroxy-3-methylbutan-2-yl]amino}-1-oxopent-4-en-2-yl}carbamate (S40)

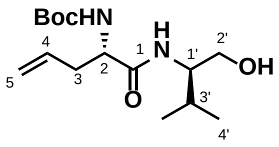

A solution of **S39** (500 mg, 2.3 mmol), L-valinol (260 μL, 2.3 mmol), HOBt·H_2O (470 mg, 3.5 mmol), and ^{i}Pr_2NEt (0.97 mL, 7.0 mmol) in DMF (12 mL) was treated with EDCI (540 mg, 3.5 mmol) at room temperature for 24 h. The mixture was partitioned between with AcOEt and H_2O. The organic phase was washed with 1 M *aq*. HCl, *sat. aq*. $NaHCO_3$, H_2O, and brine, dried (Na_2SO_4), filtered, and concentrated *in vacuo* to afford **S40** (700 mg, quant.) as a colorless oil. ^{1}H NMR ($CDCl_3$, 400 MHz) δ 6.32 (m, 1H, N*H*), 5.76 (ddt, 1H, H-4, $J_{4,5}$ = 17.9, $J_{4,5}$ = 10.1, $J_{4,3}$ = 7.8 Hz), 5.20 (d, 1H, H-5, $J_{5,4}$ = 10.1 Hz), 5.00 (br s, 1H, BocN*H*), 4.10 (dd, 1H, H-2, $J_{2,3}$ = 12.8, $J_{2,3}$ = 6.4 Hz), 3.71 (m, 1H, H-1'), 3.60-3.70 (m, 2H, H-2'), 2.67 (br s, 1H, O*H*), 2.52 (m, 2H, H-3), 1.87 (dq, 1H, H-3', $J_{3',1'}$ = 14, $J_{3',4'}$ = 6.9 Hz), 1.45 (s, 9H, ^{t}Bu), 0,93 (d, 6H, H-4', $J_{4',3'}$ = 6.8 Hz); ^{13}C NMR ($CDCl_3$, 100 MHz) δ 172.2, 162.7, 156.0, 133.3, 119.2, 80.7, 63.7, 57.4, 54.4, 36.6, 36.4, 31.6, 29.0, 28.4, 19.7, 18.8; ESIMS-LR *m*/*z* 323 [(M+Na)$^+$]; ESIMS-HR calcd for $C_{15}H_{28}O_4N_2Na$ 323.1941, found 323.1943. $[\alpha]^{20}_D$ −65.2 (*c* 1.00, $CHCl_3$).

tert-Butyl {(_S_)-1-{[(_S_,_E_)-2-Methyl-6-(methylamino)-6-oxohex-4-en-3-yl]amino}-1-oxopent-4-en-2-yl}carbamate (S41)

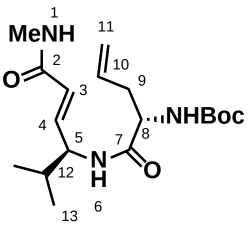

A solution of **S40** (310 mg, 1.0 mmol) in CH_2Cl_2 (10 mL) was treated with Dess-Martin periodinane (470 mg, 1.1 mmol) at room temperature for 1 h. Ethyl acetate, *sat. aq.* $NaHCO_3$, and *sat. aq.* $Na_2S_2O_3$ were added to the mixture, which was vigorously stirred for further 10 min. The organic phase was dried (Na_2SO_4), filtered, and concentrated *in vacuo*. A solution of the residue (210 mg, 1.1 mmol), $Zn(OTf)_2$ (800 mg, 2.2 mmol), and TMEDA (180 μL, 1.2

mmol) in THF (8.0 mL) was treated with Et_3N (560 μL, 4.0 mmol) at room temperature for 15 min. A solution of the crude aldehyde in THF (4 mL) was added to the mixture, which was stirred for 18 h. The mixture was concentrated *in vacuo* and the residue was partitioned between AcOEt and 1 M *aq*. HCl. The organic phase was washed with brine, dried (Na_2SO_4), filtered, and concentrated *in vacuo*. The residue was purified by trituration with Et_2O to afford **S41** (180 mg, 0.51 mmol, 51% over 2 steps) as a white solid. ^{1}H NMR ($CDCl_3$, 500 MHz) δ 6.71 (dd, 1H, H-4, $J_{4,3}$ = 14.9, $J_{4,5}$ = 5.7 Hz), 6.59 (d, 1H., H-6, $J_{6,5}$ = 8.0 Hz), 5.92 (d, 1H, H-3, $J_{3,4}$ = 14.9 Hz), 5.72 (m, 1H, H-10), 5.15 (m, 1H, H-11), 5.11 (m, 1H, H-11), 4.41 (m, 1H, H-5), 4.14 (m, 1H, H-8), 2.84 (d, 3H, NMe, J = 5.2 Hz), 2.51 (m, 2H, H-9), 1.85 (m, 1H, H-12), 1.42 (s, 9H, ^{t}Bu), 0.89 (t, 6H, H-13, $J_{13,12}$ = 6.9 Hz); ^{13}C NMR ($CDCl_3$, 125 MHz) δ 171.4, 166.2, 156.2, 141.9, 133.4, 124.7, 119.4, 80.7, 55.7, 54.3, 36.3, 32.3, 28.6, 26.6, 19.2, 18.3; ESIMS-LR *m/z* 376 [(M+Na)$^+$]; ESIMS-HR calcd for $C_{18}H_{31}O_4N_3Na$ 376.2207, found 376.2209. $[\alpha]^{18}_D$ −43.4 (*c* 1.02, MeOH).

***N*-{(*S*)-1-{{(*S*)-1-{[(*S*,*E*)-2-Methyl-6-(methylamino)-6-oxohex-4-en-3-yl]amino}-1-oxopent-4-en-2-yl}amino}-1-oxo-3-phenylpropan-2-yl}decanamide (13)**

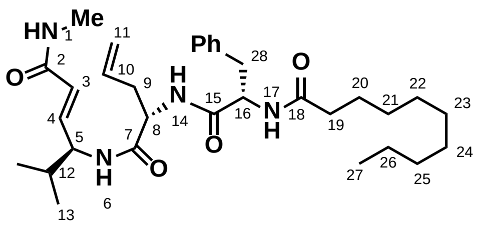

A solution of the **S41** (35 mg, 0.10 mmol) was treated with 4.0 M HCl in AcOEt (1.0 mL) at room temperature for 1 h, and the mixture was concentrated *in vacuo*. A solution of the residue, *N*-decanoyl-L-phenylalanine (35 mg, 110 μmol), ^{i}Pr_2NEt (51 μL, 300 μmol), and HOAt (23 mg, 150 μmol) in DMF (1 mL) was treated with PyBOP (78 mg, 150 μmol) at 0 °C, and the mixture was stirred at room temperature for 15 h. The mixture was partitioned between AcOEt and 1 M *aq*. HCl. The organic phase was washed with *sat. aq.* $NaHCO_3$ and H_2O, filtered, and the residue was washed with AcOEt. The residue was purified by preparative-TLC (10% MeOH/$CHCl_3$) to afford **13** (49 mg, 88 μmol, 88% over 2 steps) as a white solid. ^{1}H NMR (DMSO-d_6, 500 MHz) δ 8.12 (m, 1H, H-14), 8.11 (m, 1H, H-1), 8.06 (m, 1H, H-17), 8.05 (m, 1H, H-6), 7.14-7.23 (m, 5H, Ar), 6.48 (ddd, 1H, H-4, $J_{4,3}$ = 15.5, $J_{4,5}$ = 5.0, $J_{4,12}$ = 3.2 Hz), 5.87 (d, 1H, H-3, $J_{3,4}$ = 15.5 Hz), 5.70 (m, 1H, H-10), 5.08 (d, 1H, H-11, $J_{11,10}$ = 16.6 Hz), 5.00 (d, 1H, H-11, $J_{11,10}$ = 10.3 Hz), 4.50 (m, 1H, H-16), 4.35 (m, 1H, H-8), 4.17 (m, 1H, H-5), 3.00 (dd, 1H, H-28, $J_{28,16}$ = J_{gem} = 13.7 Hz), 2.72 (dd, 1H, H-28, $J_{28,16}$ = 10.9, J_{gem} = 13.7 Hz), 2.61 (m, 3H, NMe), 2.42 (m, 1H, H-9), 2.35 (m, 1H, H-9), 2.00 (m, 2H, H-19), 1.76 (m, 1H, H-12), 0.83-1.36 (m, 23H, H-13, H-20, H-21, H-22, H-23, H-24, H-25, H-26, H-27); ^{13}C NMR (DMSO-d_6, 125 MHz) δ 172.2, 171.1, 164.9, 140.4, 137.9, 133.9, 129.0, 127.7, 125.9, 124.4, 117.4, 54.8, 53.7, 52.3, 37.1, 36.3, 35.1, 31.5, 31.1, 28.7, 28.5, 28.3, 25.3, 25.0, 21.9, 18.8, 18.3, 13.8; ESIMS-LR *m/z* 577 [(M+Na)$^+$]; ESIMS-HR calcd for $C_{32}H_{50}O_4N_4Na$ 577.3724, found 577.3726.

Scheme S9. Synthesis of **14**

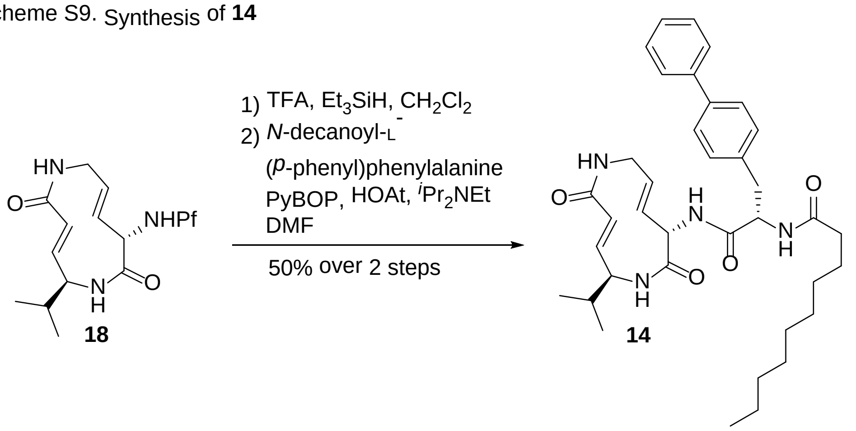

***N*-{(*S*)-3-[(1,1'-Biphenyl)-4-yl]-1-{[(3*E*,5*S*,8*S*,9*E*)-5-isopropyl-2,7-dioxo-1,6-diazacycloundeca-3,9-dien-8-yl]amino}-1-oxopropan-2-yl}decanamide (14)**

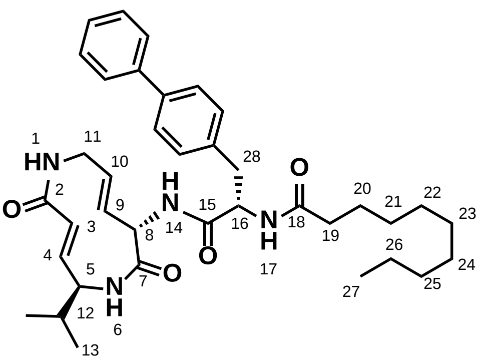

A solution of **18** (20 mg, 42 μmol) and Et_3SiH (17 μL, 110 μmol) in CH_2Cl_2 (0.50 mL) was treated with TFA (0.50 mL) at 0 °C, and the mixture was stirred at room temperature for 3 h. The mixture was concentrated *in vacuo*. A solution of the residue, *N*-decanoyl-L-(*p*-phenyl)phenylalanine (25 mg, 130 μmol), $^i Pr_2NEt$ (22 μL, 130 μmol), and HOAt (10 mg, 63 μmol) in DMF (0.50 mL) was treated with PyBOP (33 mg, 63 μmol) at room temperature for 20 h. The mixture was partitioned between AcOEt and 1 M *aq.* HCl. The mixture was filtered, and the insoluble contents was washed with *sat. aq.* $NaHCO_3$, H_2O, and AcOEt. The residue was purified by preparative-TLC (10% MeOH/$CHCl_3$) to afford **14** (13 mg, 21 μmol, 50% over 2 steps) as a white solid. ^1H NMR (DMSO-d_6, 500 MHz) δ 8.32 (m, 1H, H-14), 8.05 (d, 1H, H-17, $J_{17,16}$ = 6.9 Hz), 7.33-7.62 (m, 11H, H-Ar, H-1, H-6), 6.18 (m, 1H, H-4), 5.98 (d, 1H, H-3, $J_{3,4}$ =15.5 Hz), 5.61 (m, 2H, H-9, H-10), 4.79 (m, 1H, H-8), 4.60 (m, H-16), 3.87 (m, 1H, H-5), 3.73 (m, 2H, H-11), 3.05 (d, 1H, H-28, J_{gem} = 13.8 Hz), 2.74 (t, 1H, H-28, J = 11.5 Hz), 1.99 (m, 1H, H-19), 1.72 (m, 1H, H-12), 1.31 (m, 2H, H-20), 0.80-1.9 (m, 21H, H-13, H-21-27) ; ^{13}C NMR (DMSO-d_6, 125 MHz) δ 185.6, 171.2, 170.8, 169.1, 168.3, 139.9, 137.8, 137.2, 129.6, 128.7, 127.0, 126.3, 126.0, 53.3, 43.8, 36.9, 35.1, 31.2, 31.1, 28.7, 28.6, 28.2, 25.1, 21.9, 19.5, 19.1, 13.8; ESIMS-LR *m*/*z* 637 [(M+Na)$^+$]; ESIMS-HR calcd for $C_{37}H_{51}O_4N_4$ 615.391, found 615.391.

4) Evaluation of *in vitro* β5 proteasome inhibitory activity and determination of kinetic constants

In vitro proteasome inhibitory activity was measured on 96-well microtiter plates using human erythrocyte 20S proteasome (R&D Systems, Inc.) with the AK-740 Assay Kit for Drug Discovery (Biomol). Reactions were performed at 37 °C in 100 μL volumes containing a serial dilution of test samples, 2 μg/mL 20S proteasome, and 100 μM Suc-LLVY-AMC for assaying chymotrypsin-like activity, according to the manufacture's instructions. Fluorescence was monitored with an infinite M200 microplate reader (Tecan) equipped with 360 nm excitation and 465 nm emission filters, and the results were graphically represented in Figures S1. The observed rate constant for inhibition, k_{obs}, at each concentration was determined from the slope of a semi-logarithmic plot of inhibition versus time. The k_{obs} values were re-plotted against inhibitor concentrations as shown in Figure S1, and fitted to a hyperbolic equation, $k_{obs} = k_2[I]/(K_i+[I])$, to obtain values for K_i and k_2. The k_2/K_i ratio represents the second-order rate constant for the reaction of the inhibitor with the target. [S7)]

Data for analogue **1**

(a)

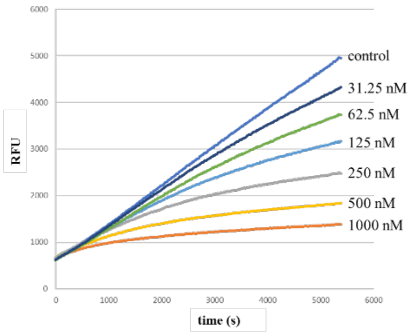

(b)

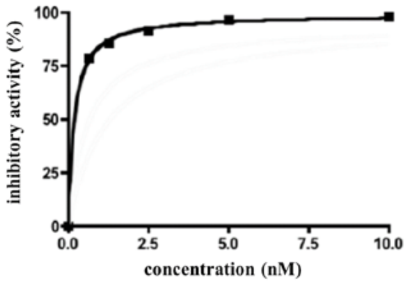

(c)

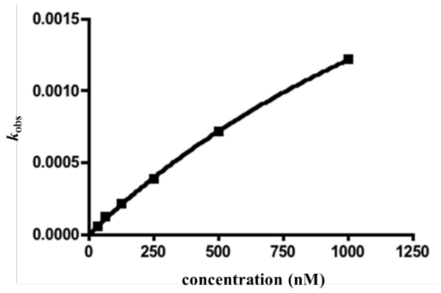

Data for analogue **2**

(d)

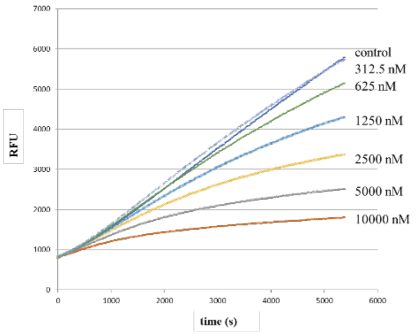

(e)

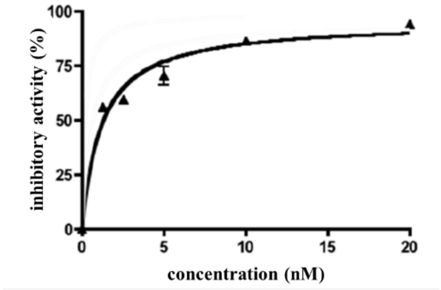

(f)

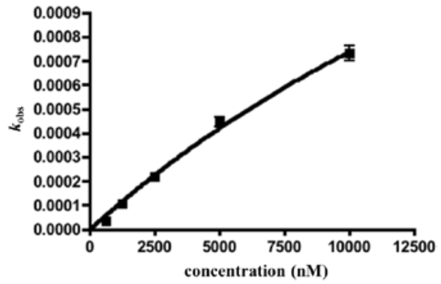

Data for analogue **3**

(g)

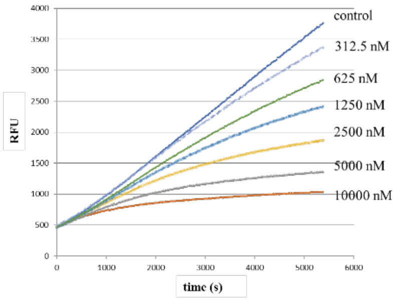

(h)

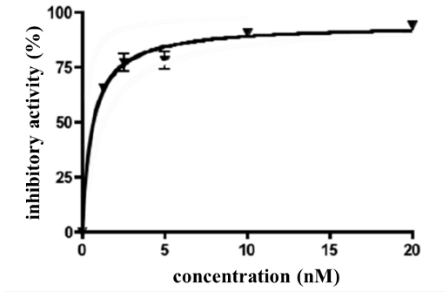

(i)

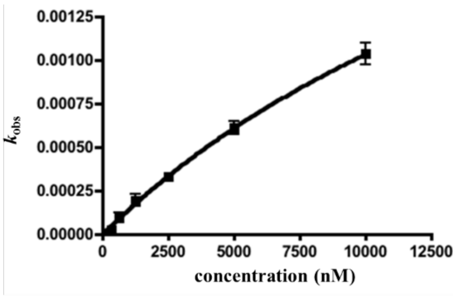

Figure S1-1. In vitro β5 proteasome inhibitory activity and k_{obs} plots. (a) (d) (g) Comparative data of the β5 proteasome inhibitory activity. (b) (e) (h) Proteasome β5 inhibitory activity. (c) (f) (i) The dependence of the observed k_{obs} on the concentration of inhibitor.

Data for analogue **4**

(j)

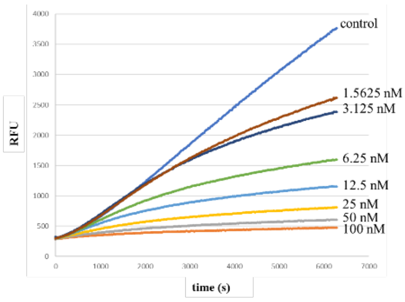

(k)

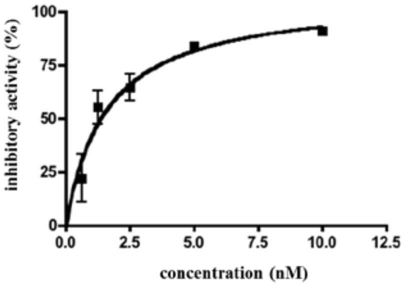

(l)

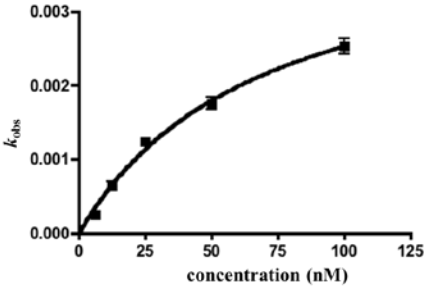

Data for analogue **5**

(m)

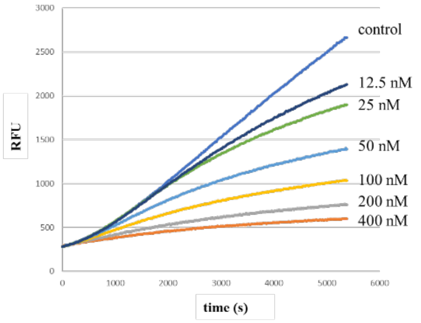

(n)

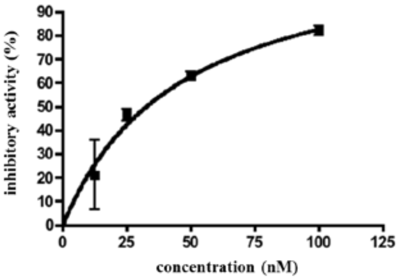

(o)

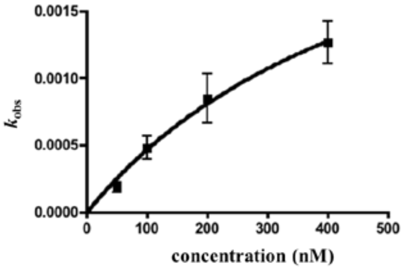

Data for analogue **6**

(p)

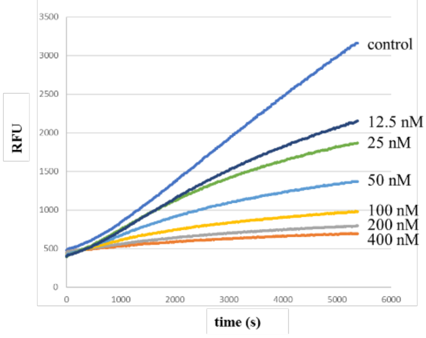

(q)

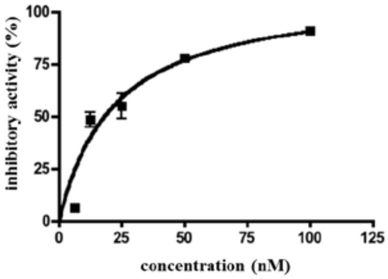

(r)

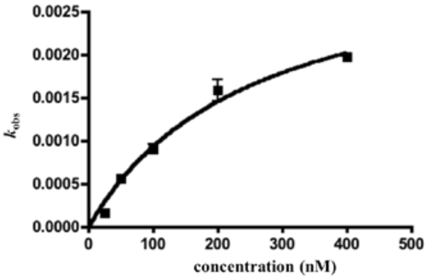

Figure S1-2. In vitro β5 proteasome inhibitory activity and k_{obs} plots. (j) (m) (p) Comparative data of the β5 proteasome inhibitory activity. (k) (n) (q) Proteasome β5 inhibitory activity. (l) (o) (r) The dependence of the observed k_{obs} on the concentration of inhibitor.

Data for analogue **8**

(s)

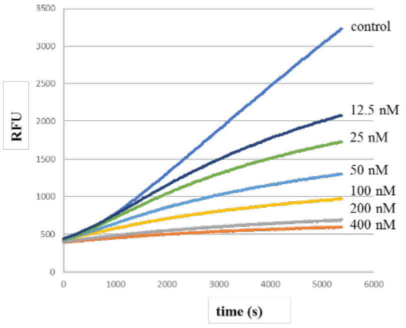

(t)

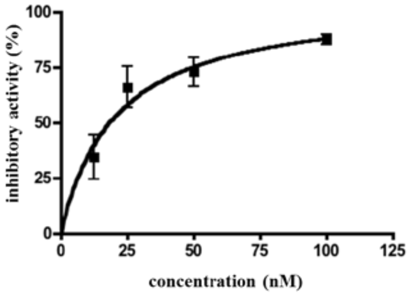

(u)

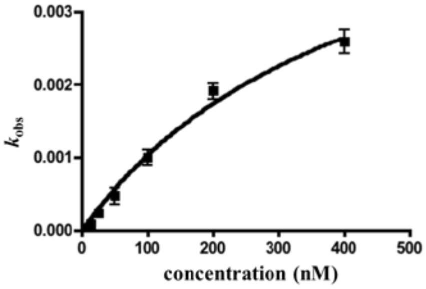

Data for analogue **9**

(v)

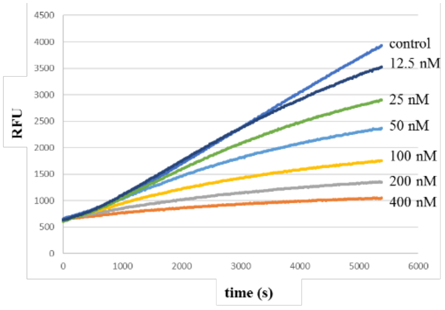

(w)

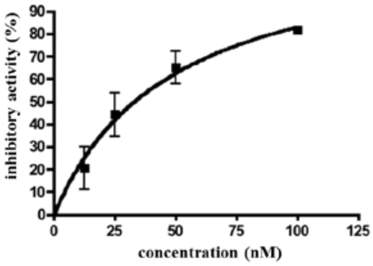

(x)

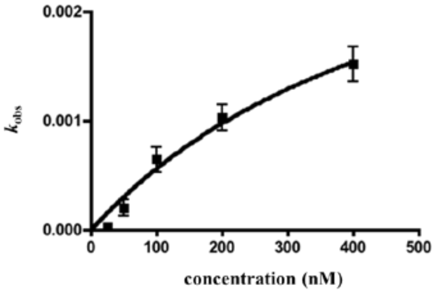

Data for analogue **10**

(y)

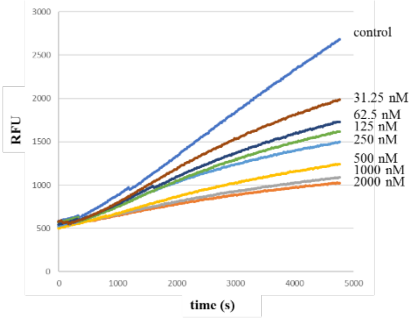

(z)

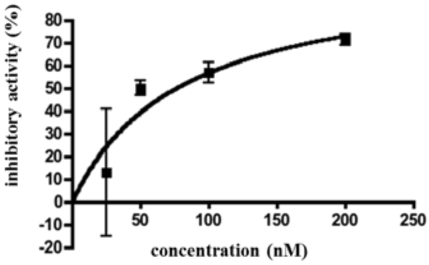

(aa)

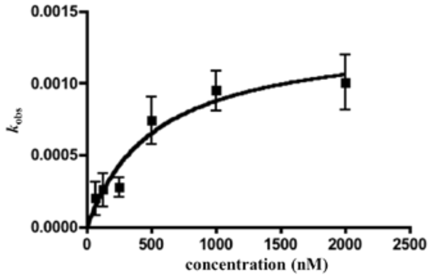

Figure S1-3. In vitro β5 proteasome inhibitory activity and k_{obs} plots. (s) (v) (y) Comparative data of the β5 proteasome inhibitory activity. (t) (w) (z) Proteasome β5 inhibitory activity. (u) (x) (aa) The dependence of the observed k_{obs} on the concentration of inhibitor.

Data for analogue **11**

(bb)

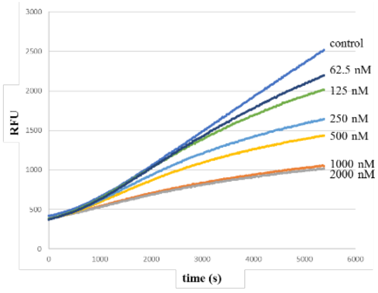

(cc)

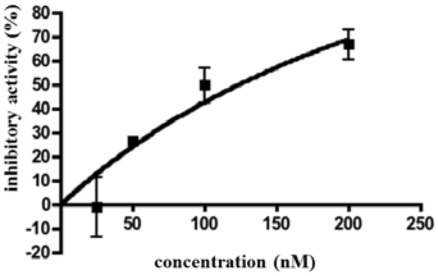

(dd)

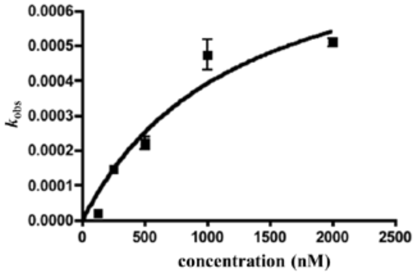

Data for analogue **12**

(ee)

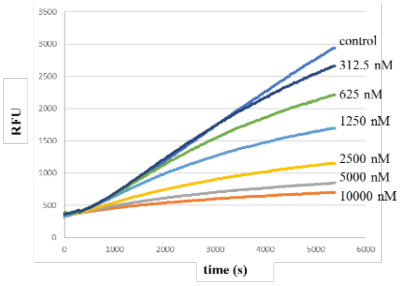

(ff)

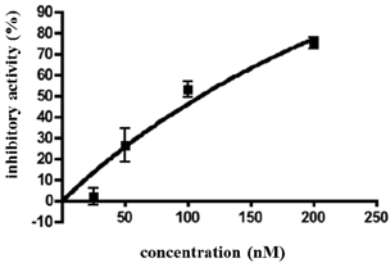

(gg)

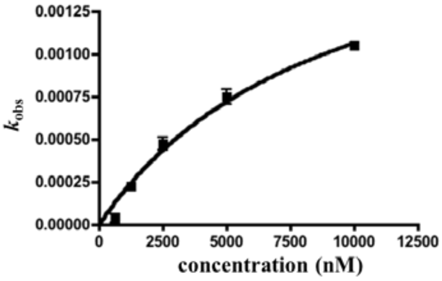

Data for analogue **14**

(hh)

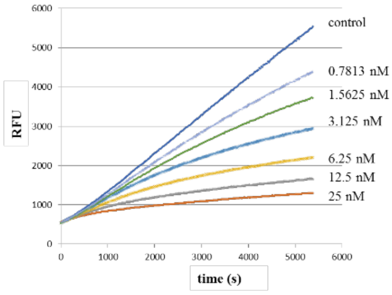

(ii)

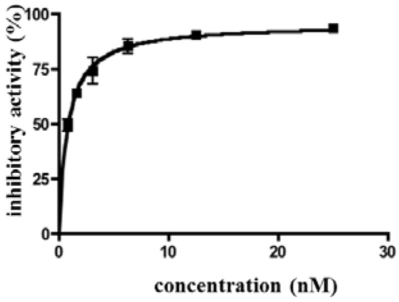

(jj)

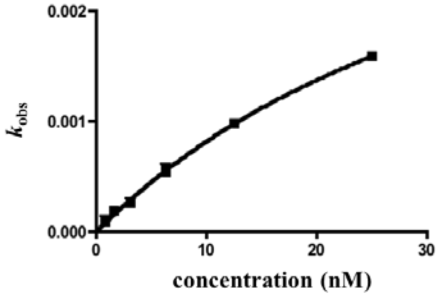

Figure S1-4. In vitro β5 proteasome inhibitory activity and k_{obs} plots. (bb) (ee) (hh) Comparative data of the β5 proteasome inhibitory activity. (cc) (ff) (ii) Proteasome β5 inhibitory activity. (dd) (gg) (jj) The dependence of the observed k_{obs} on the concentration of inhibitor.

5) Evaluation of anti-proliferation activity against Amo-1 cells

Cell proliferation assays were performed using the Cell Counting Kit-8 (CCK-8) (Dojindo, CK04) according to the manufacturer's protocol. In brief, Amo-1 cells were cultured in RPMI1640 medium containing 10% FBS and 1% penicillin-streptomycin. A cell suspension at 1×10^5 cells/ml (100 µL) was seeded into a 96-well plate in each well. After culturing for 24 h at 37 °C under atmosphere of 5% CO_2, the cells were treated with a solution of the compound in DMSO (1 µL) at 37 °C for 72 h. A solution of the CCK-8 (10 µL) was then added to each well and the cells were incubated at 37 °C for 3 h. The absorbance at 450 nm was measured using an Infinite M200 PRO multimode microplate reader (Tecan). The data points were calculated from the average (±standard deviation) of three assays and plotted using Microsoft Excel 2016. Compound concentrations that exhibited 50% inhibition of cell proliferation (IC_{50}) were calculated from curves constructed by plotting cell proliferation (%) versus the log of compound concentration (nM). IC_{50} (nM): **4** = 4.33±1.6, **8** = 26.3±9.3, **14** = 12.1±3.2.

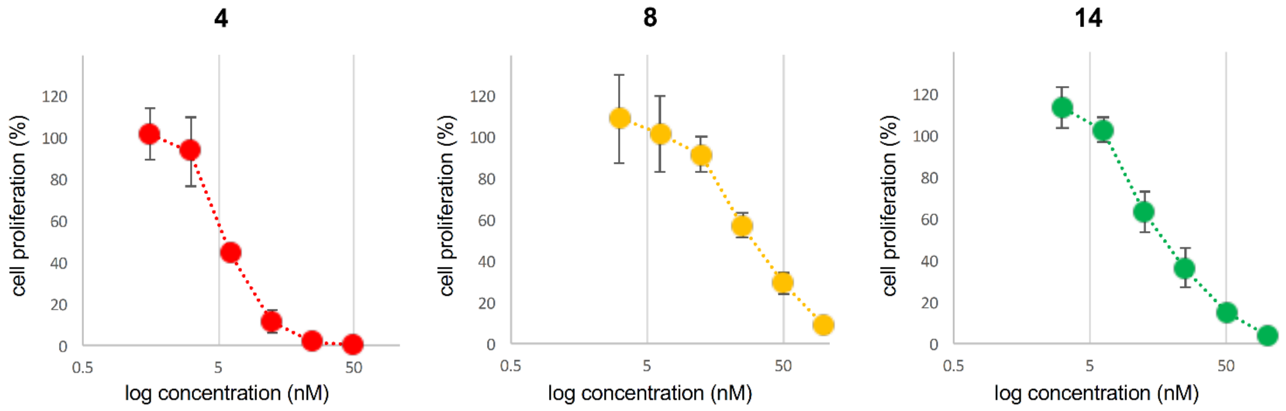

Figure S2. Anti-cell proliferative activity of **4**, **8**, and **14** on Amo-1 cells.

6) Conformational calculation

Conformational search of **7**, **8**, **11**, and **12** was carried out using the Monte Carlomultiple minimum (MCMM) method (1000 steps), followed by Polak-Ribiere conjugate gradient (PGCG) minimization with the OPLS 2005 force field. The other settings were used as default.

Conformational search of **4**, **5**, **6**, **9**, and **10** was carried out using the Monte Carlomultiple minimum (MCMM) method (1000 steps), followed by Polak-Ribiere conjugate gradient (PGCG) minimization with the OPLS 2005 force field with constraints on to build structures that fulfill tortional angles derived from $J_{4,5}$ measured at 400 or 500 MHz ^{1}H NMR. The other settings were used as default. To calculate tortional angles, the vinyl-allylic proton coupling constants in analogues were calculated using the Garbish equations: $J_{4,5} = 6.6\cos^2\theta + 2.6\sin^2\theta$ (0°<θ<90°), $J_{4,5} = 11.6\cos^2\theta + 2.6\sin^2\theta$ (θ>90°).[S8)] The other settings were used as default. Side chains of all structures were replaced by acetyl group.

**compound**	$J_{4,5}$ (Hz)	dihedral angle(°)
**4**	5.4	33, 123
**5**	5.2	36, 122
**6**	5	39, 121
**7**	-	-
**8**	-	-
**9**	8.6	145
**10**	6.3	16, 130
**11**	-	-
**12**	-	-

Table S1. Coupling constants of analogues and calculated dihedral angles.

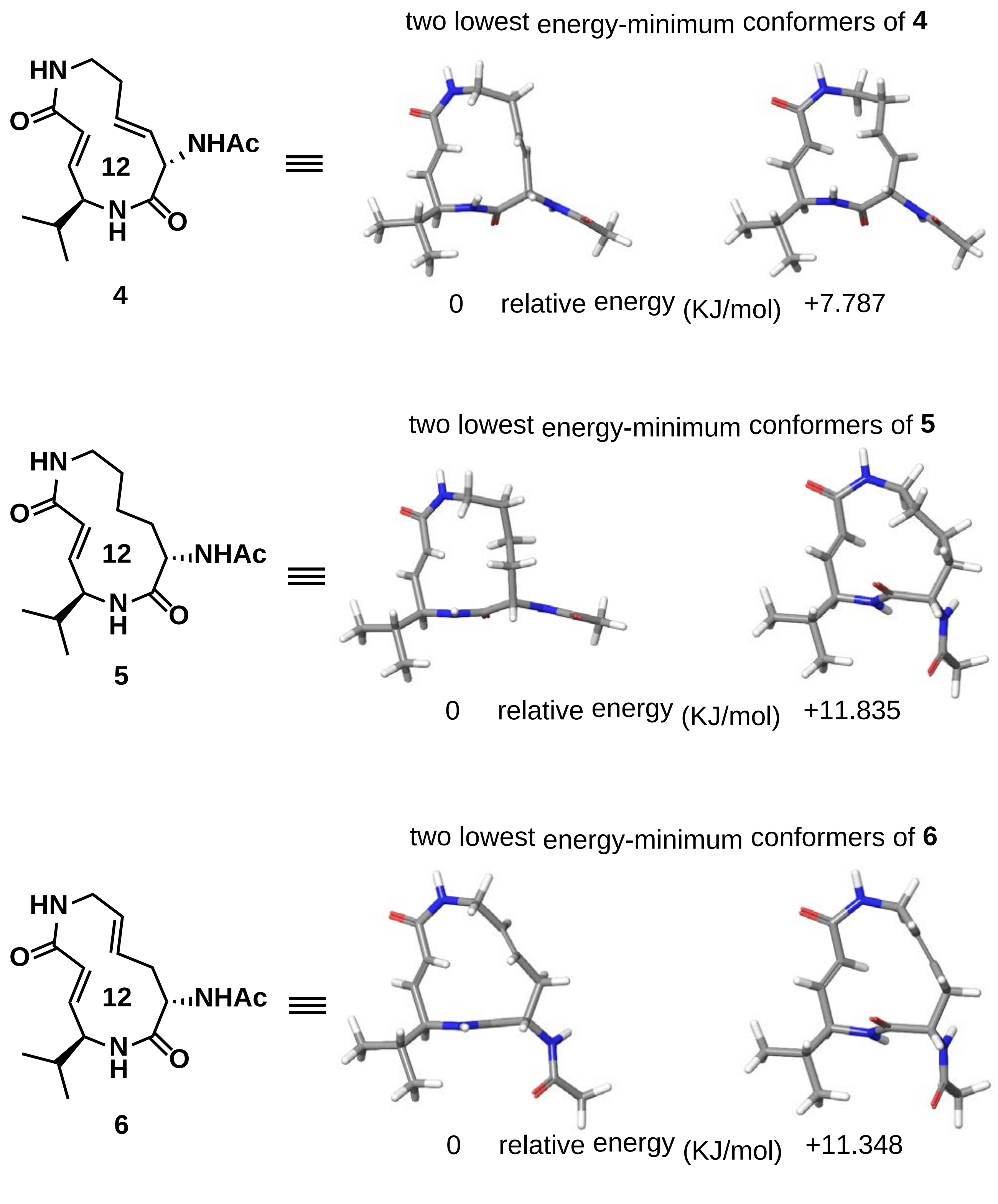

Figure S3-1. Calculated lowest energy-minimum conformers of the core structures of analogues **4-6**.

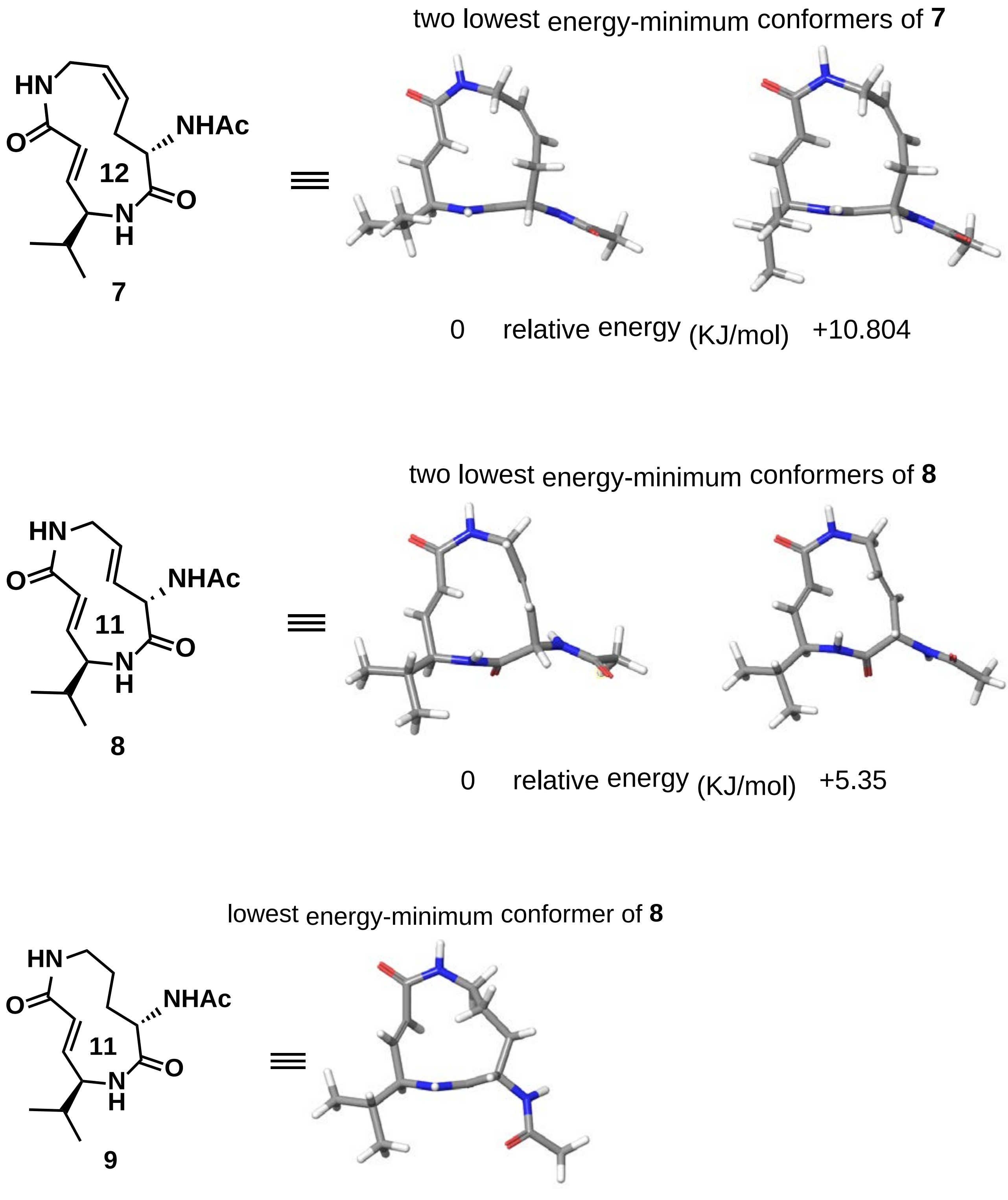

Figure S3-2. Calculated lowest energy-minimum conformers of the core structures of analogues **7-9**.

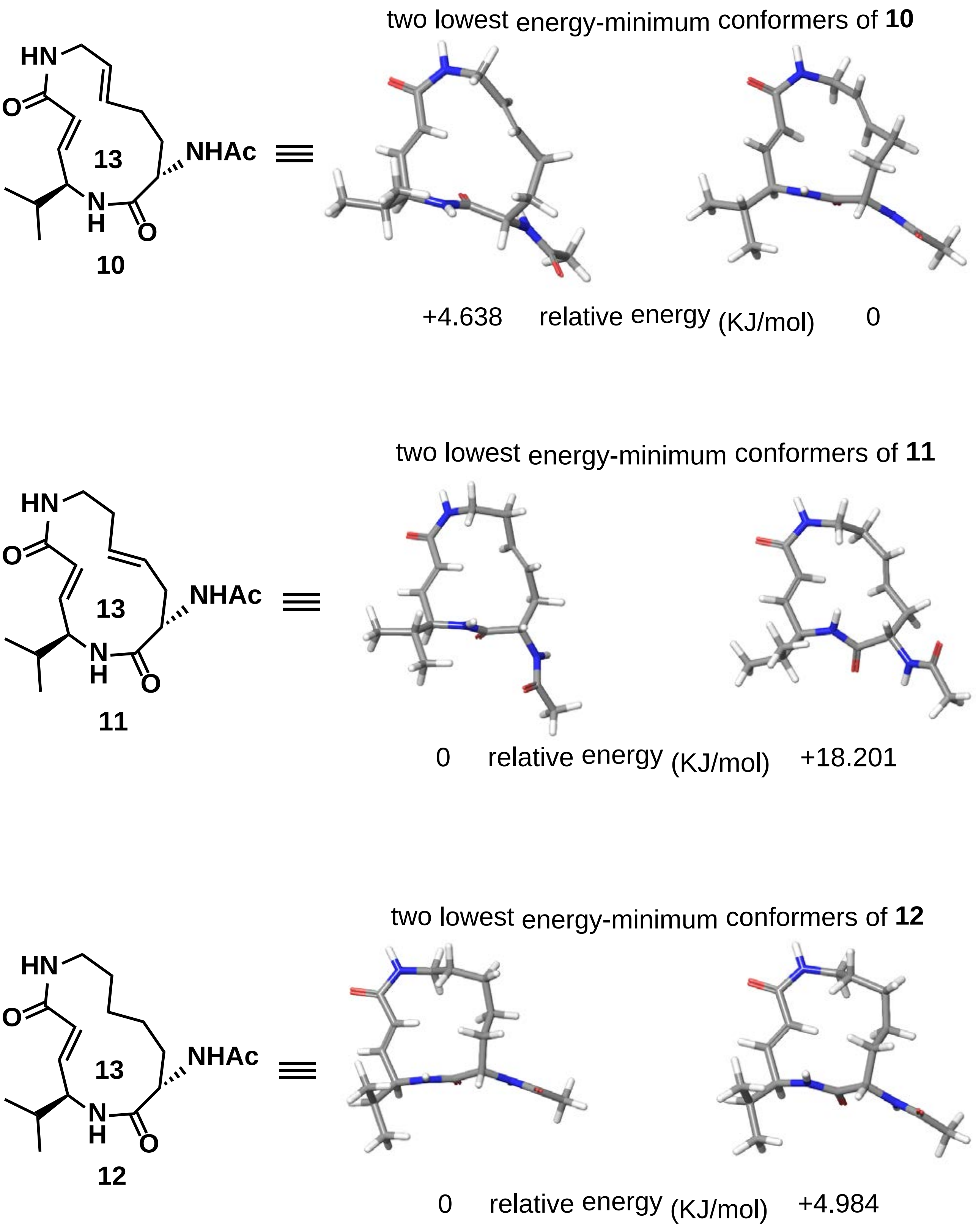

Figure S3-3. Calculated lowest energy-minimum conformers of the core structures of analogues **10-12**.

References

S1) M. M. Paz, F. J. Sardina, *J. Org. Chem.* **1993**, *58*, 6990-6995.

S2) S. Kitahata, T. Chiba, T. Yoshida, M. Ri, S. Iida, A. Matsuda, S. Ichikawa, *Org. Lett.* **2016**, *18*, 2312-2315.

S3) M. Pirrung, G. Biswas, T. Ibarra-Rivera, *Org. Lett.* **2010**, *12*, 2402-2405.

S4) P. Gmeiner, P. L. Feldman, M. Chu-Moyer, H. Rapoport, *J. Org. Chem.* **1990**, *55*, 3068-3074.

S5) S. Ortial, R. Dave, Z. Benfodda, D. Bénimélis, P. Meffre, *Synlett* **2014**, *25*, 569-573.

S6) K. Voigtritter, S. Ghorai, B. Lipshutz, *J. Org. Chem.* **2011**, *76*, 4697-4702.

S7) a) G. Fanteany, R. F. Standaert, W. S. Lane, S. Choi, E. J. Corey, S. L. Schreiber, *Science* **1995**, *268*, 726-731.
b) J. Singh, E. M. Dobrusin, D. W. Fry, T. Haske, A. Whitty, J. McNamara, *J. Med. Chem.* **1997**, *40*, 1130-1135.

S8) E. W. Garbisch, *J. Am. Chem. Soc*. **1964**, *86*, 5561-5564.

7) ^{1}H and ^{13}C NMR spectra of compounds

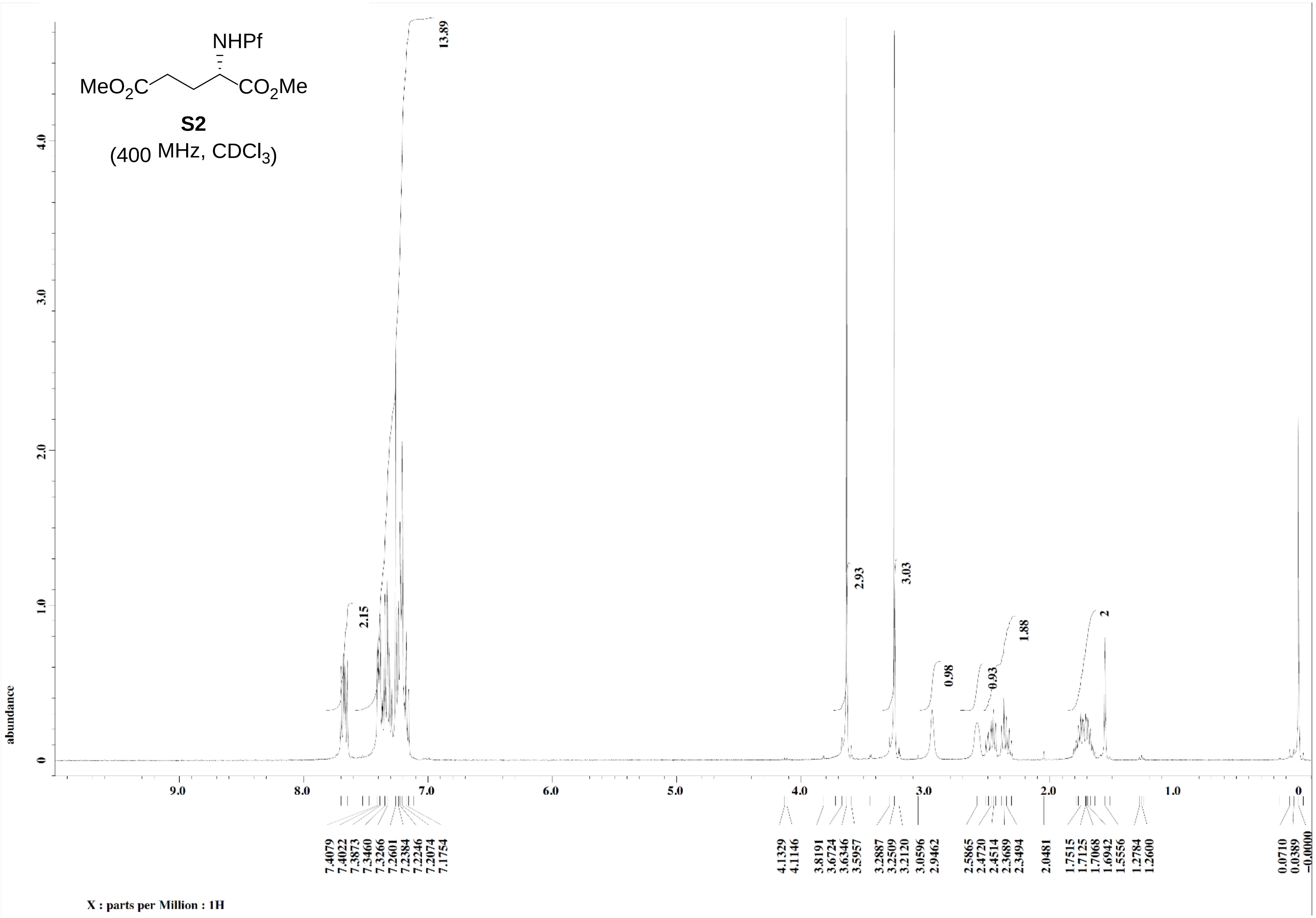

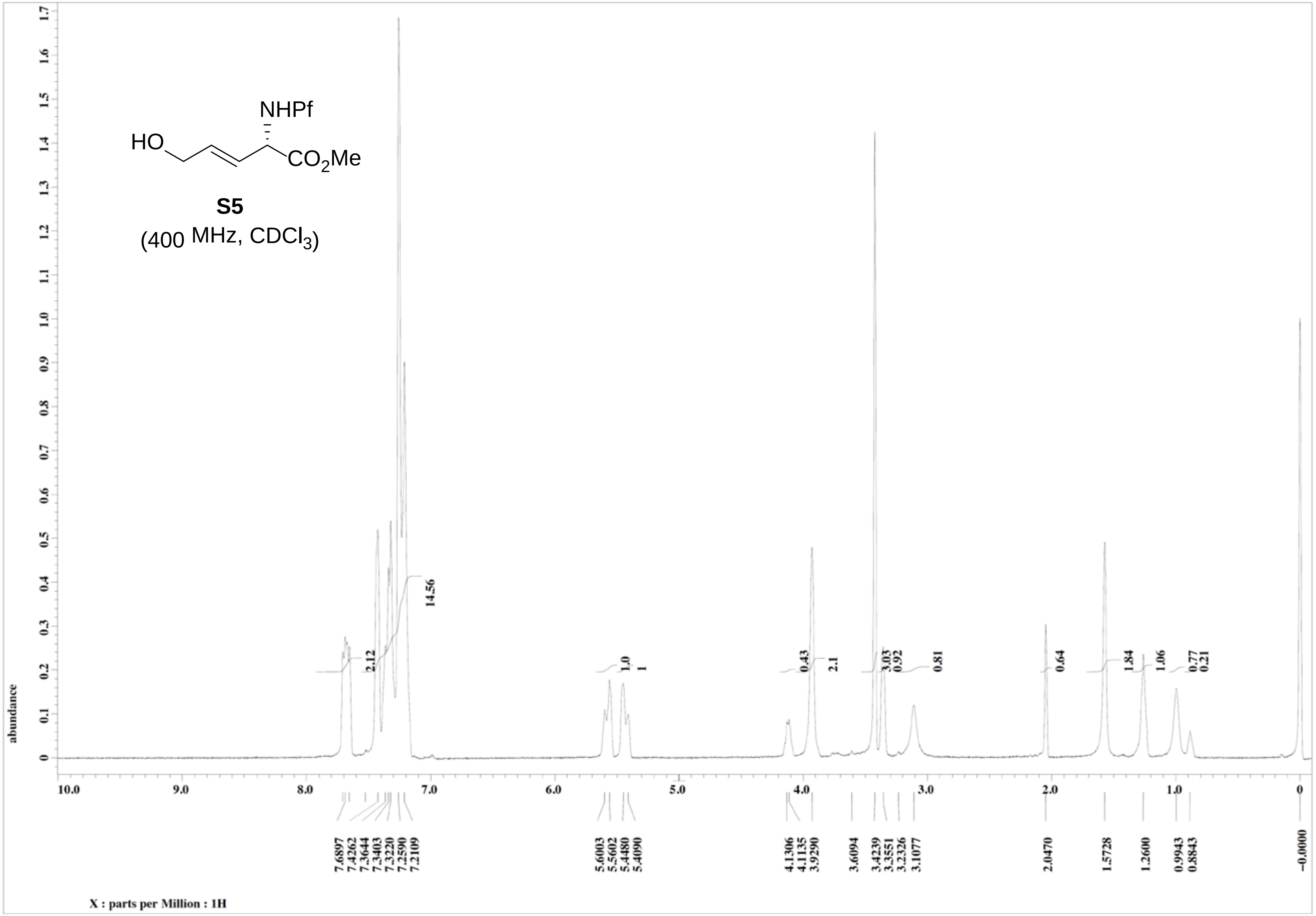
NHPf
HO
CO2Me
S5
(400 MHz, CDCl3)
abundance
X : parts per Million : 1H
7.6897
7.4262
7.3644
7.3403
7.3220
7.2590
7.2109
5.6003
5.5602
5.4480
5.4090
4.1306
4.1135
3.9290
3.6094
3.4239
3.3551
3.2326
3.1077
2.0470
1.5728
1.2600
0.9943
0.8843
-0.0000
2.12
14.56
1.0
1
0.43
2.1
3.03
0.92
0.81
0.64
1.84
1.06
0.77
0.21

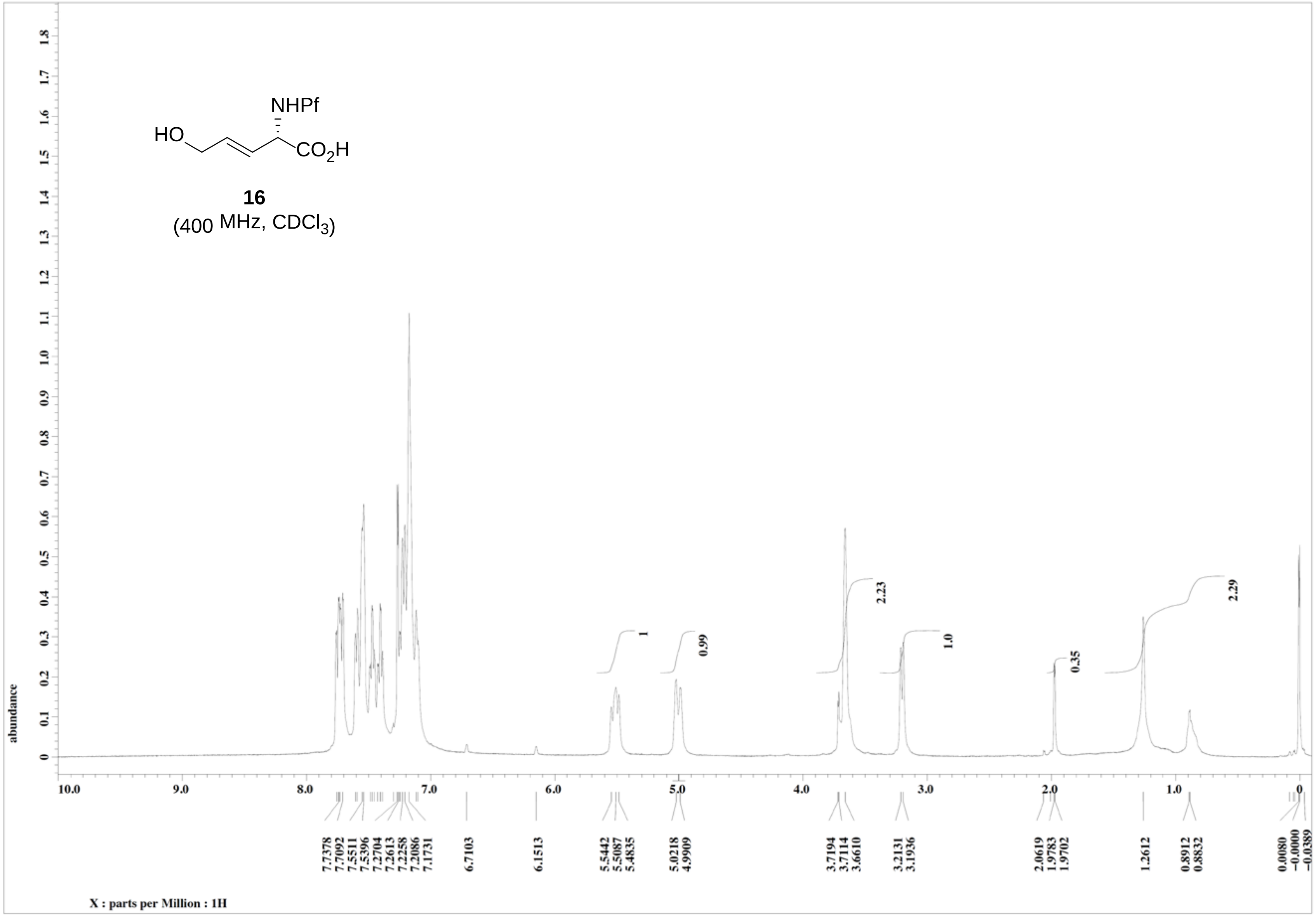

NHPf
HO
CO2H
16
(400 MHz, CDCl3)
abundance
X : parts per Million : 1H

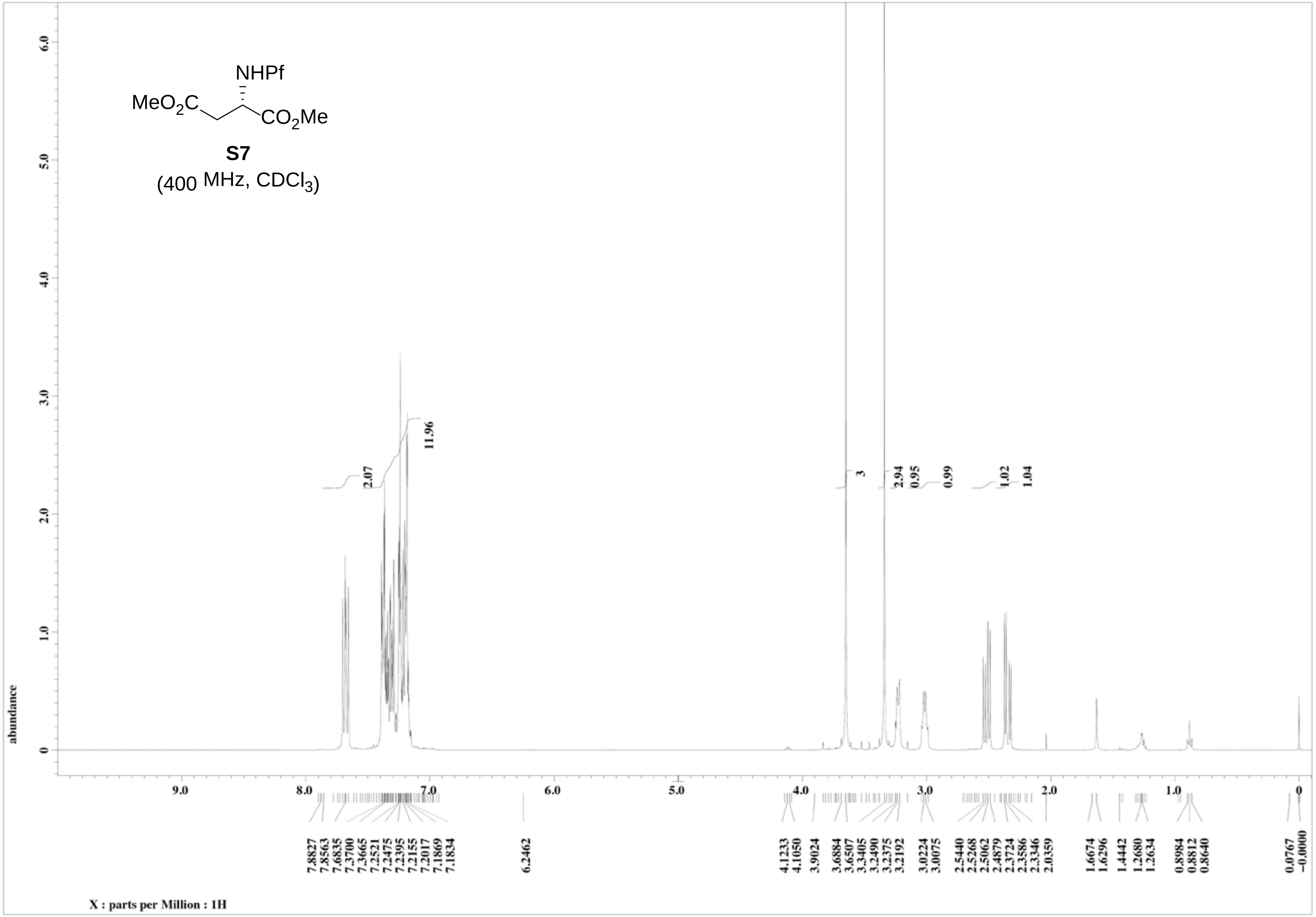

NHPf
MeO2C
CO2Me
S7
(400 MHz, CDCl3)
abundance
X : parts per Million : 1H
2.07
11.96
3
2.94
0.95
0.99
1.02
1.04
7.8827
7.8563
7.6835
7.3700
7.3665
7.2521
7.2475
7.2395
7.2155
7.2017
7.1869
7.1834
6.2462
4.1233
4.1050
3.9024
3.6884
3.6507
3.3405
3.2490
3.2375
3.2192
3.0224
3.0075
2.5440
2.5268
2.5062
2.4879
2.3724
2.3586
2.3346
2.0359
1.6674
1.6296
1.4442
1.2680
1.2634
0.8984
0.8812
0.8640
0.0767
−0.0000

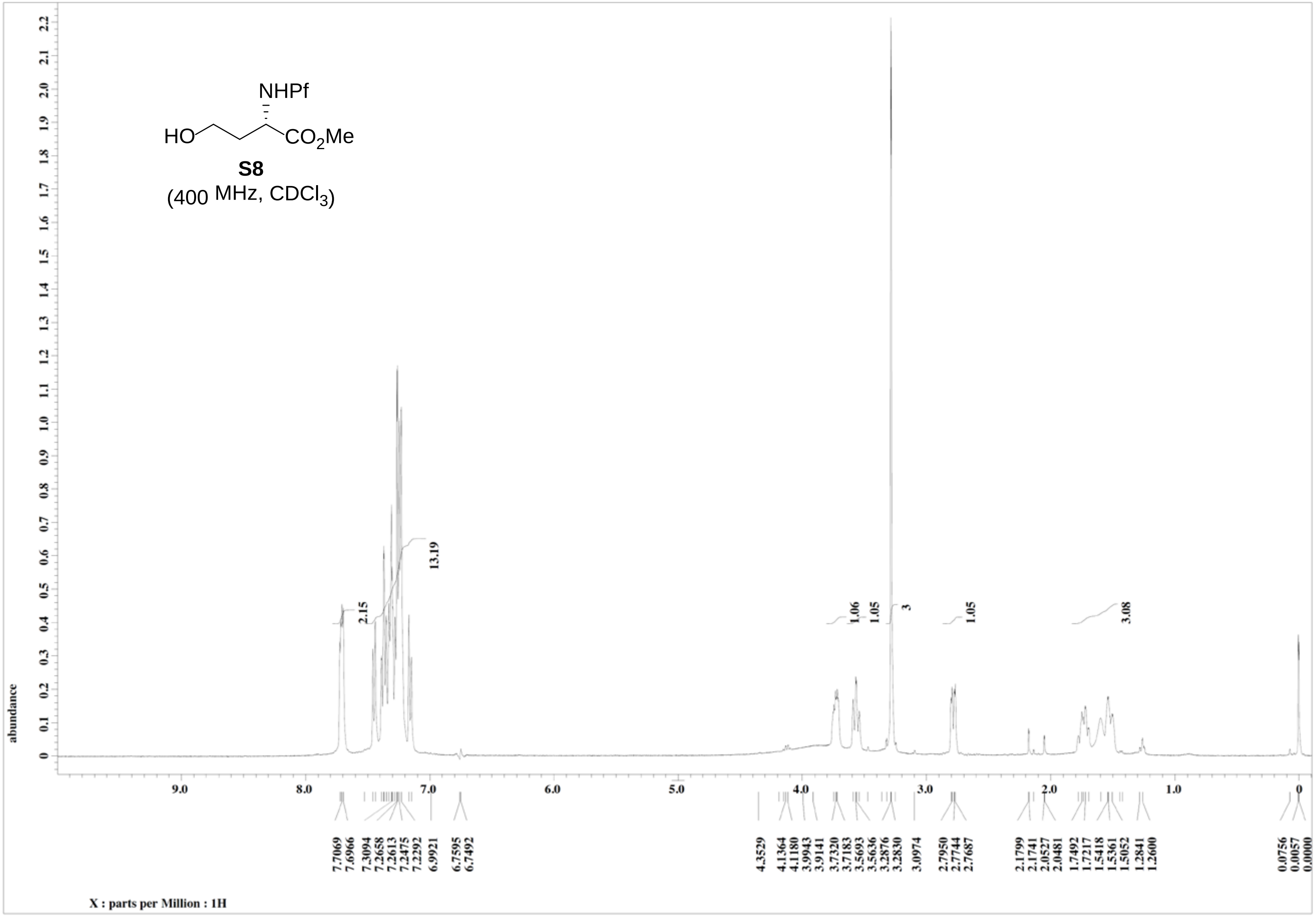
NHPf
HO
CO2Me
S8
(400 MHz, CDCl3)
abundance
X : parts per Million : 1H
2.15
13.19
1.06
1.05
3
1.05
3.08
7.7069
7.6966
7.3094
7.2658
7.2613
7.2475
7.2292
6.9921
6.7595
6.7492
4.3529
4.1364
4.1180
3.9943
3.9141
3.7320
3.7183
3.5693
3.5636
3.2876
3.2830
3.0974
2.7950
2.7744
2.7687
2.1799
2.1741
2.0527
2.0481
1.7492
1.7217
1.5418
1.5361
1.5052
1.2841
1.2600
0.0756
0.0057
0.0000

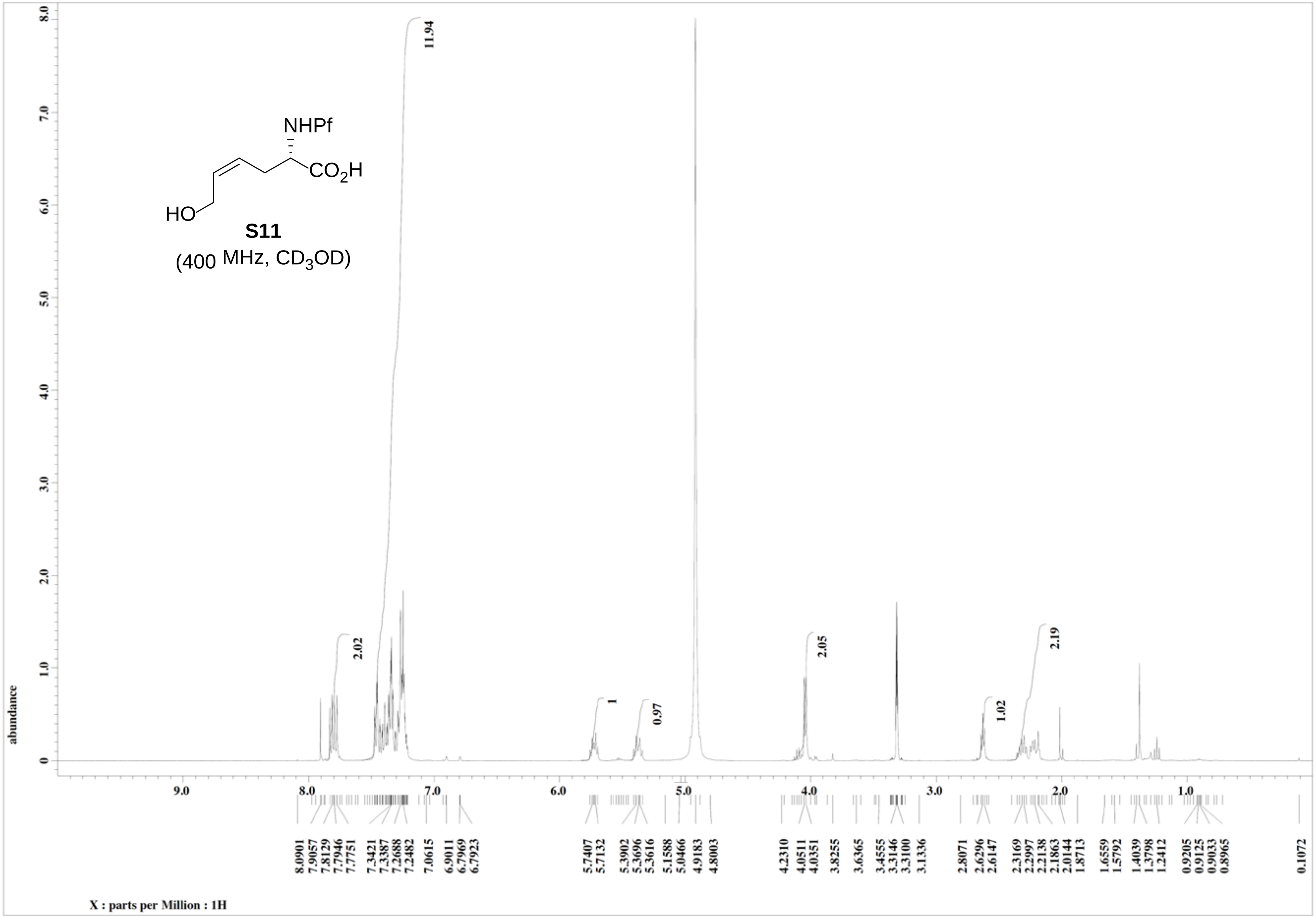
NHPf
CO2H
HO
S11
(400 MHz, CD3OD)
abundance
11.94
2.02
1
0.97
2.05
1.02
2.19
X : parts per Million : 1H
8.0901
7.9057
7.8129
7.7946
7.7751
7.3421
7.3387
7.2688
7.2482
7.0615
6.9011
6.7969
6.7923
5.7407
5.7132
5.3902
5.3696
5.3616
5.1588
5.0466
4.9183
4.8003
4.2310
4.0511
4.0351
3.8255
3.6365
3.4555
3.3146
3.3100
3.1336
2.8071
2.6296
2.6147
2.3169
2.2997
2.2138
2.1863
2.0144
1.8713
1.6559
1.5792
1.4039
1.3798
1.2412
0.9205
0.9125
0.9033
0.8965
0.1072

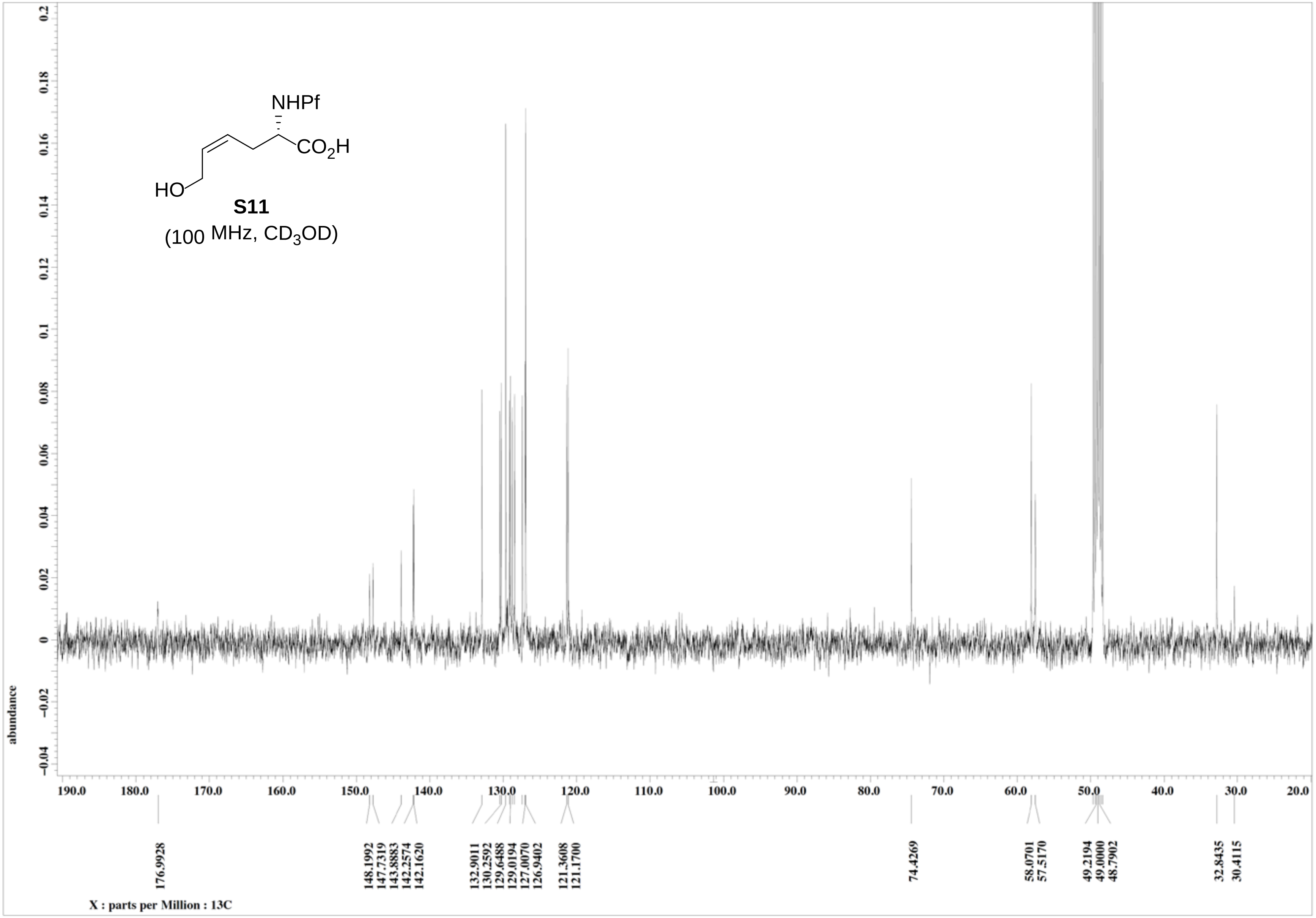
NHPf
CO_2H
HO
S11
(100 MHz, CD_3OD)
abundance
X : parts per Million : 13C
176.9928
148.1992
147.7319
143.8883
142.2574
142.1620
132.9011
130.2592
129.6488
129.0194
127.0070
126.9402
121.3608
121.1700
74.4269
58.0701
57.5170
49.2194
49.0000
48.7902
32.8435
30.4115

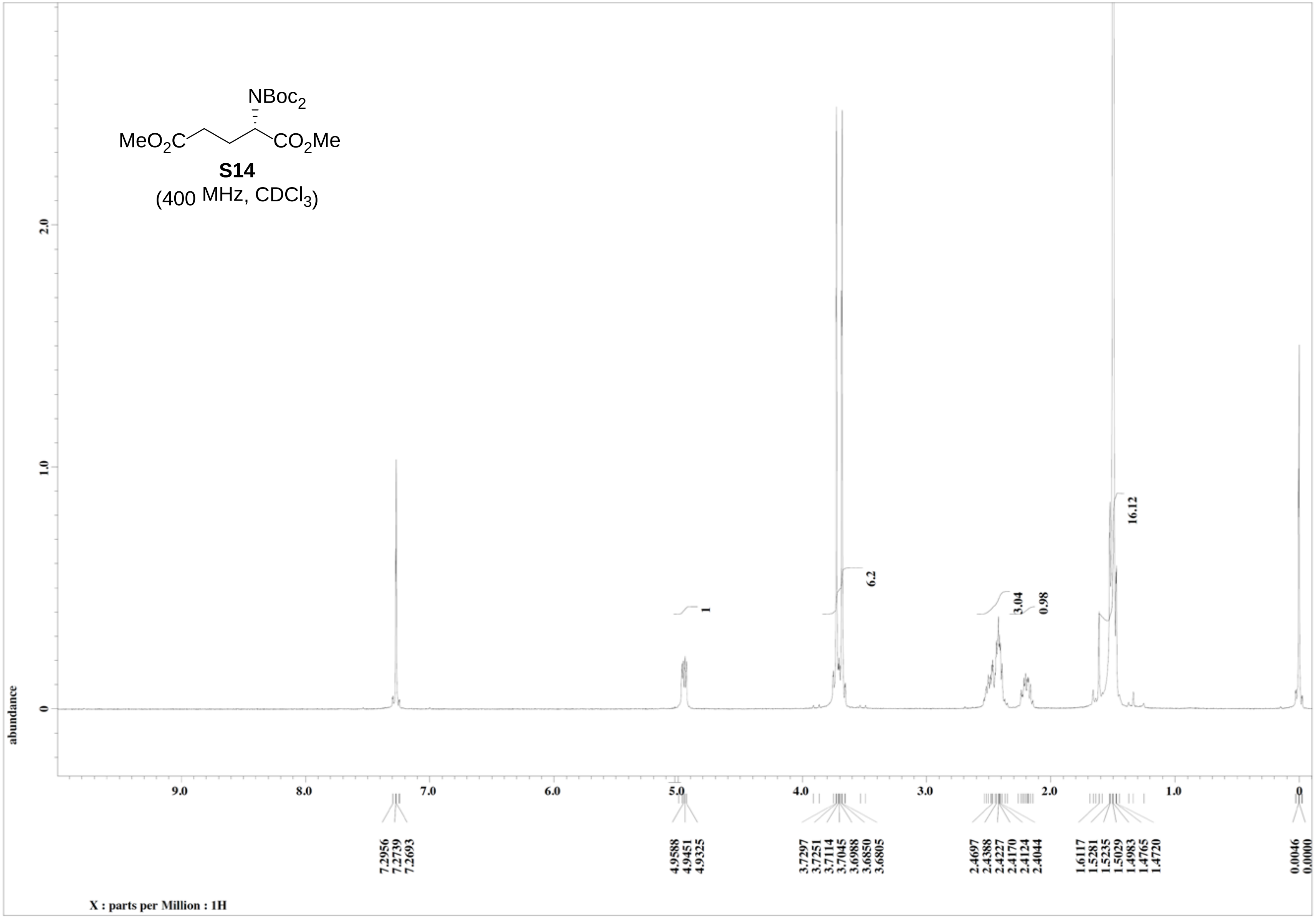
NBoc2
MeO2C
CO2Me
S14
(400 MHz, CDCl3)
abundance
X : parts per Million : 1H
7.2956
7.2739
7.2693
4.9588
4.9451
4.9325
3.7297
3.7251
3.7114
3.7045
3.6988
3.6850
3.6805
2.4697
2.4388
2.4227
2.4170
2.4124
2.4044
1.6117
1.5281
1.5235
1.5029
1.4983
1.4765
1.4720
0.0046
0.0000
1
6.2
3.04
0.98
16.12

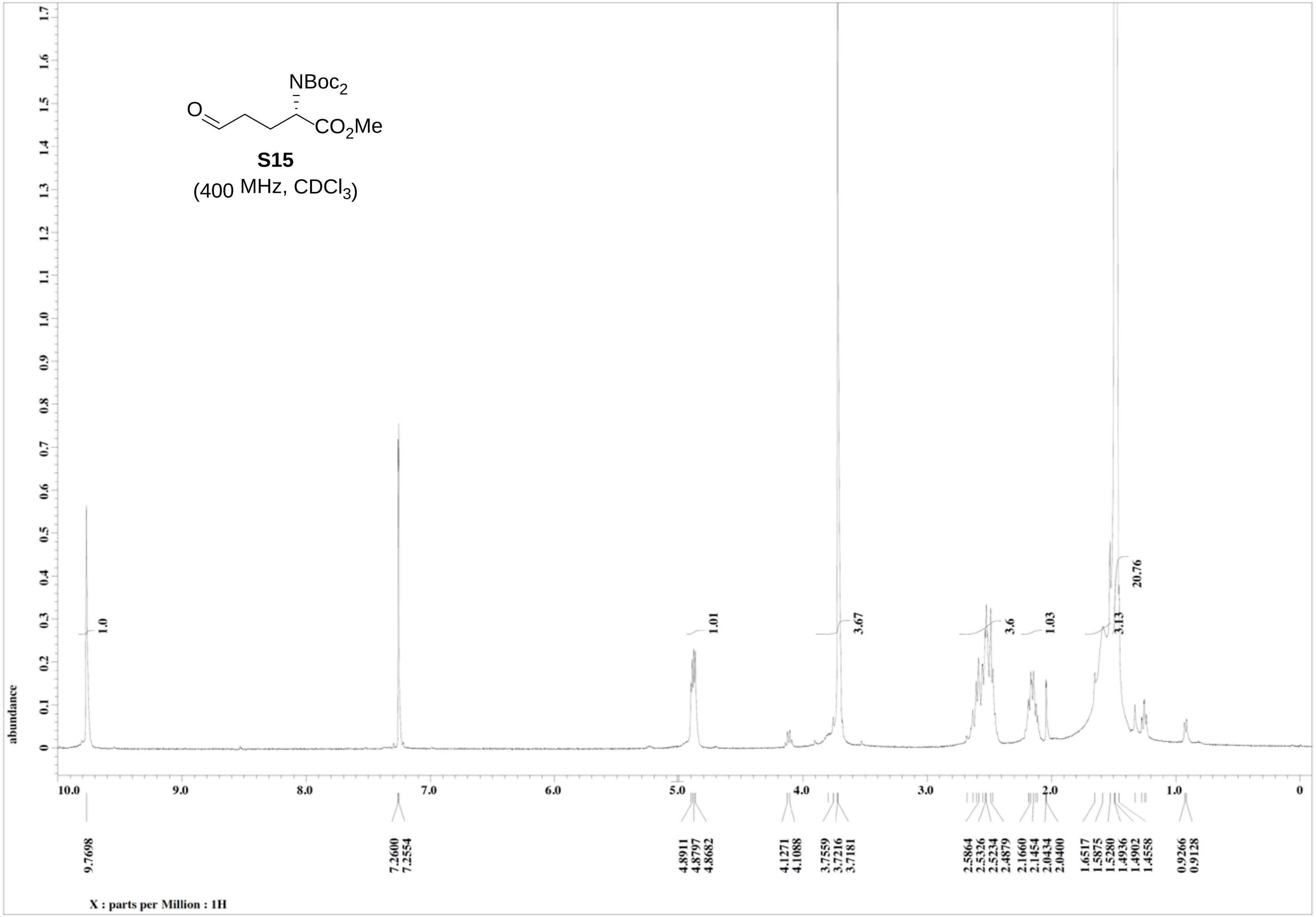
NBoc2
O
CO2Me
S15
(400 MHz, CDCl3)
abundance
X : parts per Million : 1H
9.7698
7.2600
7.2554
4.8911
4.8797
4.8682
4.1271
4.1088
3.7559
3.7216
3.7181
2.5864
2.5326
2.5234
2.4879
2.1660
2.1454
2.0434
2.0400
1.6517
1.5875
1.5280
1.4936
1.4902
1.4558
0.9266
0.9128
1.0
1.01
3.67
3.6
1.03
3.13
20.76

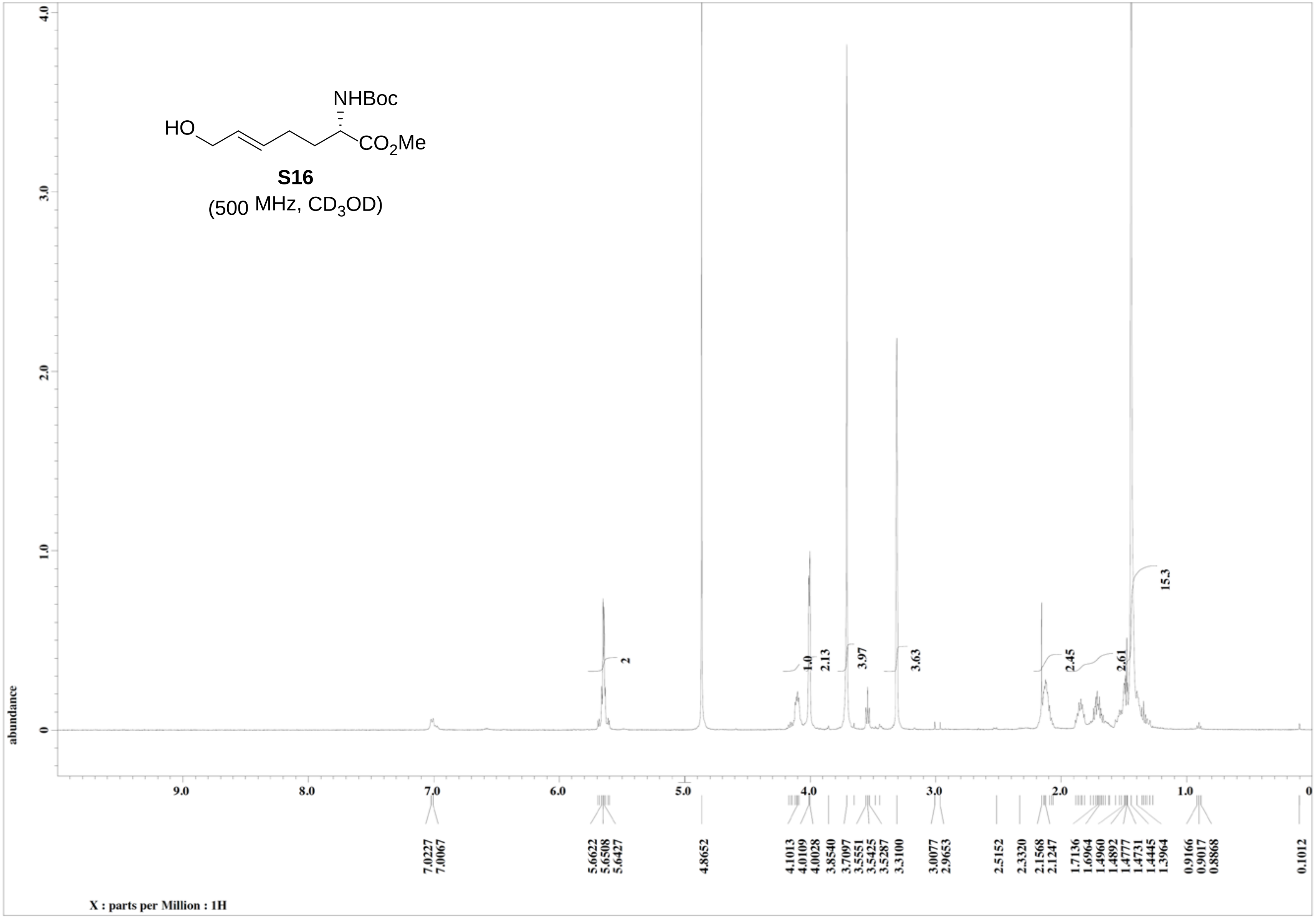
NHBoc
HO
CO2Me
S16
(500 MHz, CD3OD)
abundance
X : parts per Million : 1H

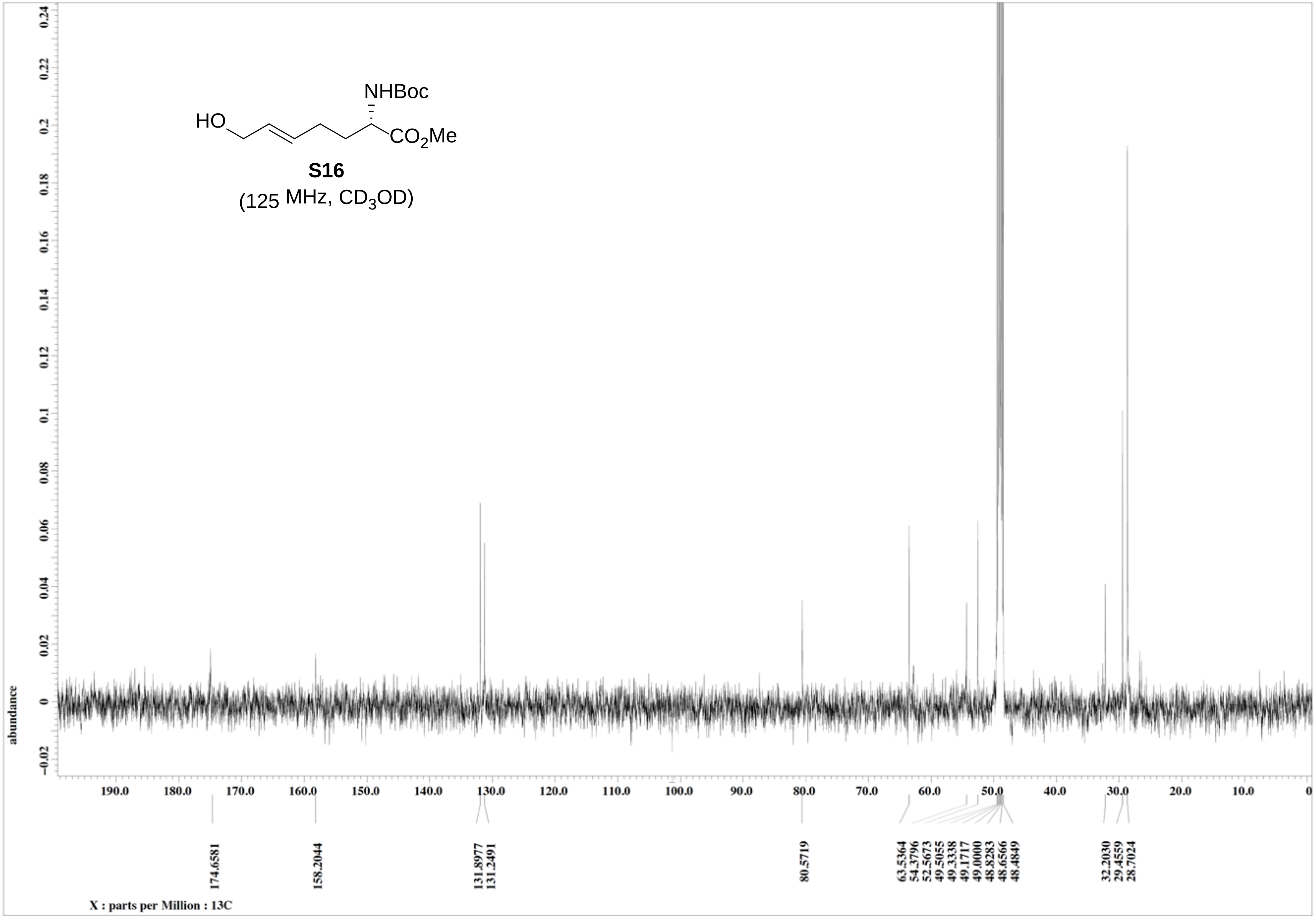

NHBoc
HO
CO2Me
S16
(125 MHz, CD3OD)
abundance
X : parts per Million : 13C
174.6581
158.2044
131.8977
131.2491
80.5719
63.5364
54.3796
52.5673
49.5055
49.3338
49.1717
49.0000
48.8283
48.6566
48.4849
32.2030
29.4559
28.7024

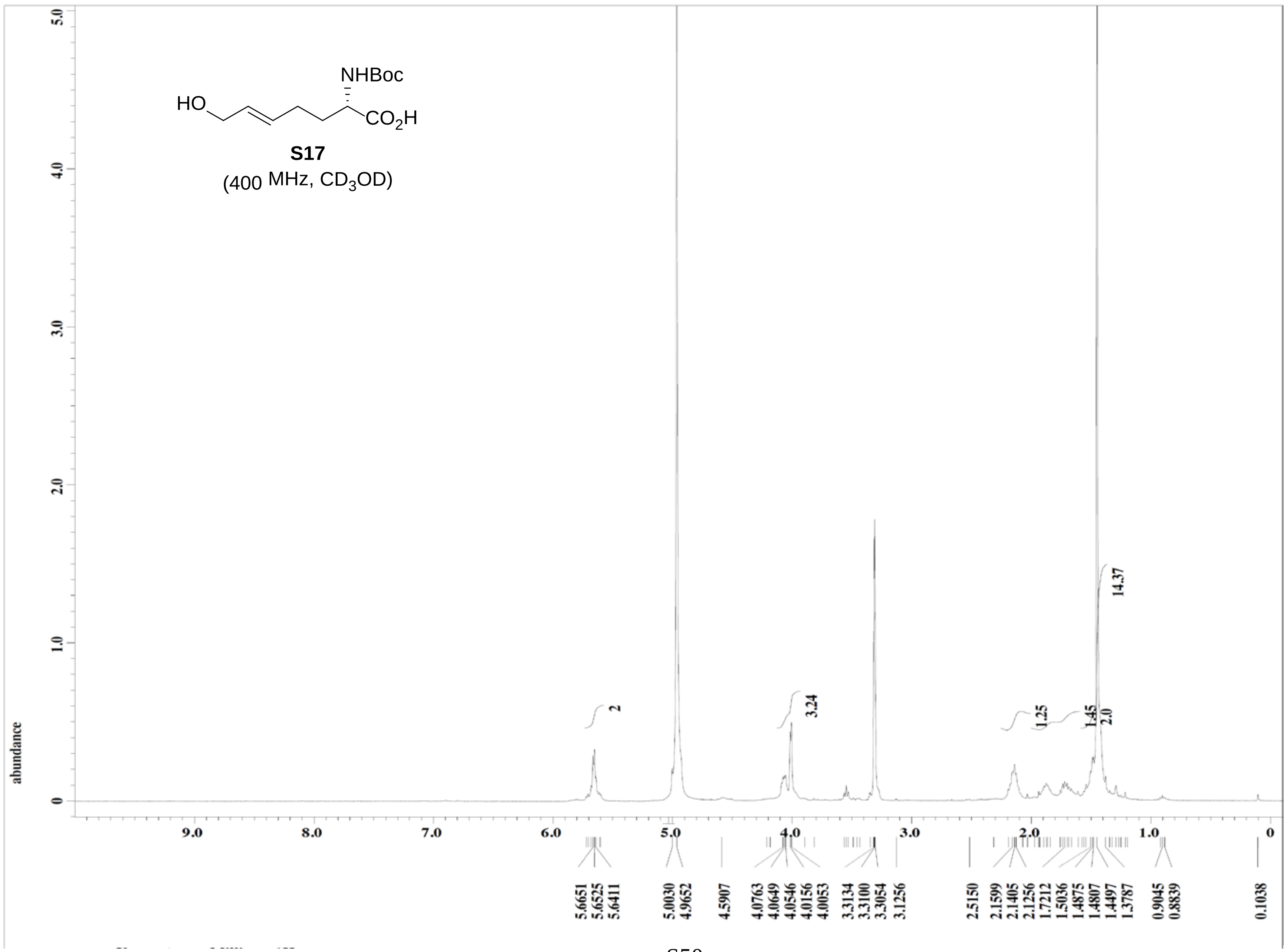

NHBoc
HO
CO2H
S17
(400 MHz, CD3OD)
abundance
5.0
4.0
3.0
2.0
1.0
0
9.0
8.0
7.0
6.0
5.0
4.0
3.0
2.0
1.0
0
2
3.24
1.25
1.45
2.0
14.37
5.6651
5.6525
5.6411
5.0030
4.9652
4.5907
4.0763
4.0649
4.0546
4.0156
4.0053
3.3134
3.3100
3.3054
3.1256
2.5150
2.1599
2.1405
2.1256
1.7212
1.5036
1.4875
1.4807
1.4497
1.3787
0.9045
0.8839
0.1038

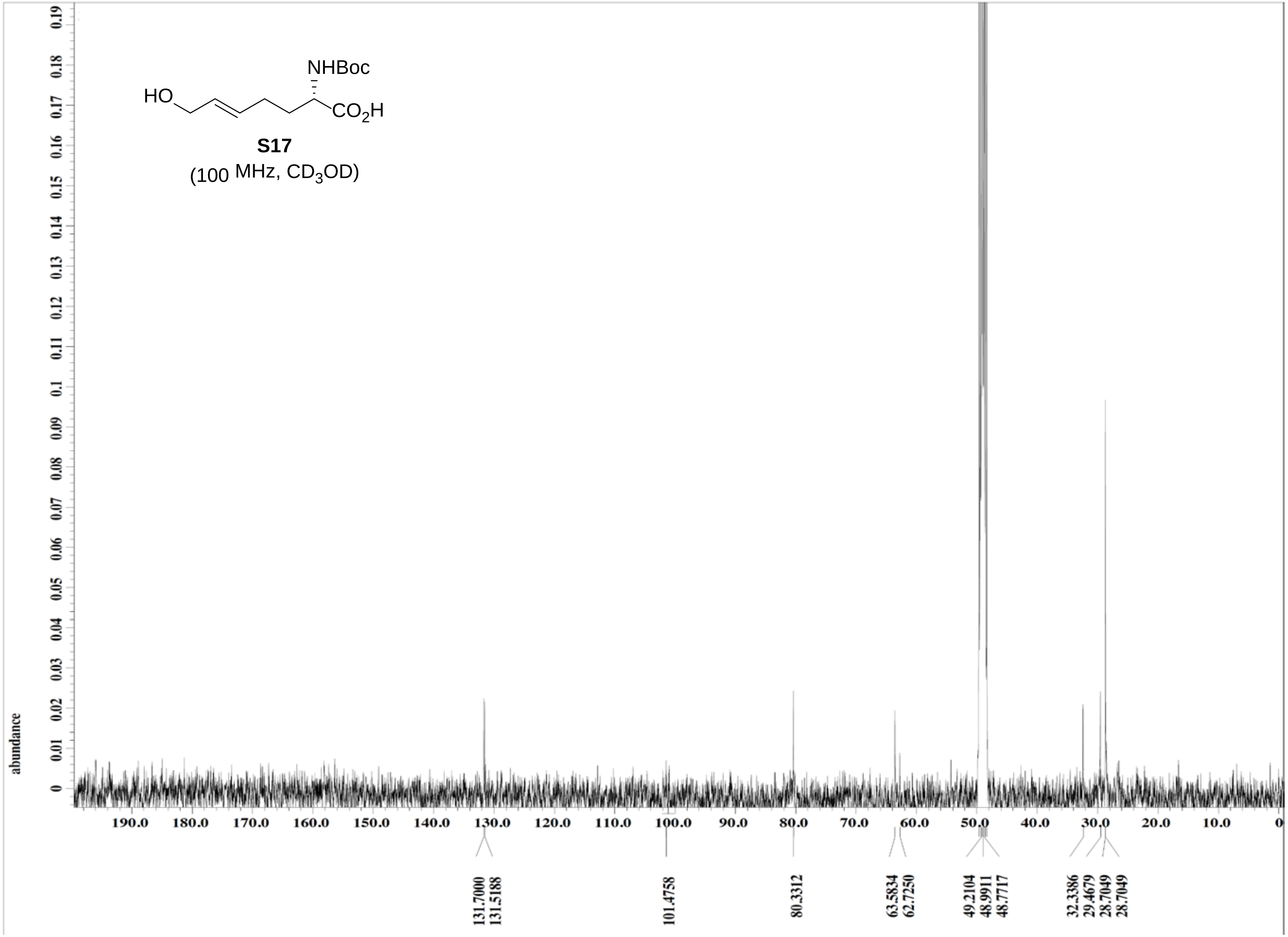
NHBoc
HO
CO2H
S17
(100 MHz, CD3OD)
abundance
131.7000
131.5188
101.4758
80.3312
63.5834
62.7250
49.2104
48.9911
48.7717
32.3386
29.4679
28.7049
28.7049

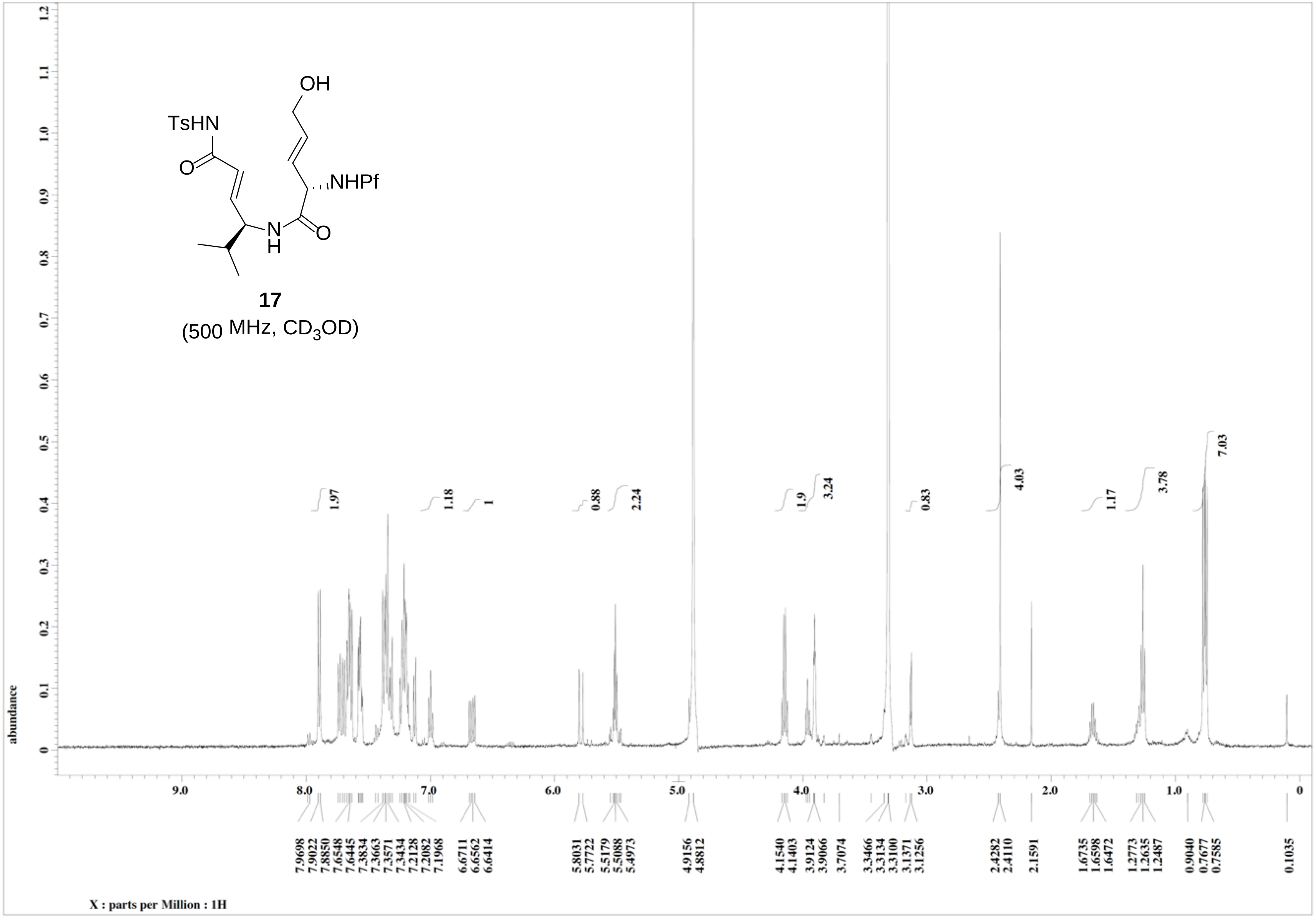

17

(500 MHz, CD_3OD)

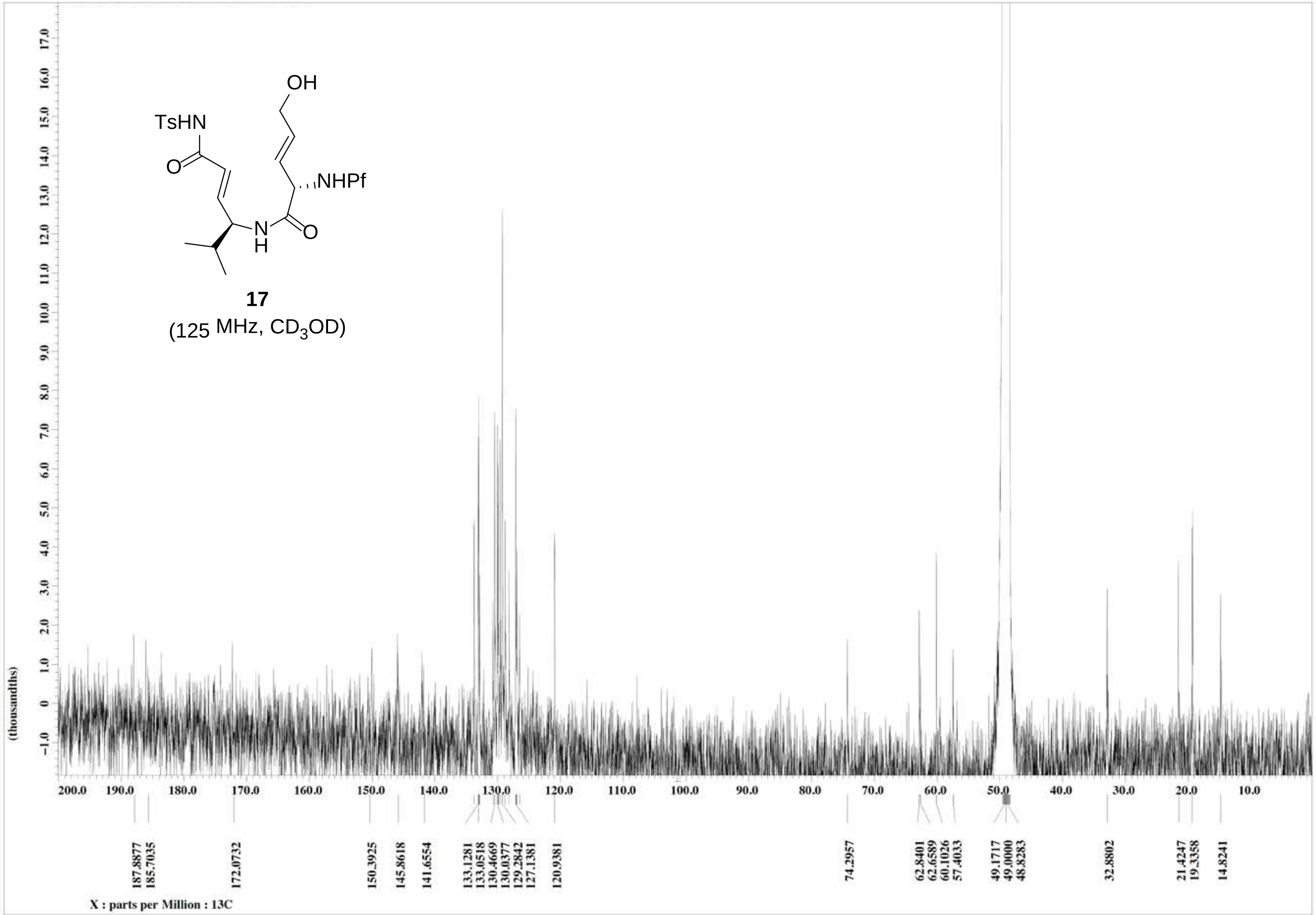

TsHN
O
NHPf
OH
N
H
O
17
(125 MHz, CD_3OD)
(thousandths)
X : parts per Million : 13C
187.8877
185.7035
172.0732
150.3925
145.8618
141.6554
133.1281
133.0518
130.4669
130.0377
129.2842
127.1381
120.9381
74.2957
62.8401
62.6589
60.1026
57.4033
49.1717
49.0000
48.8283
32.8802
21.4247
19.3358
14.8241

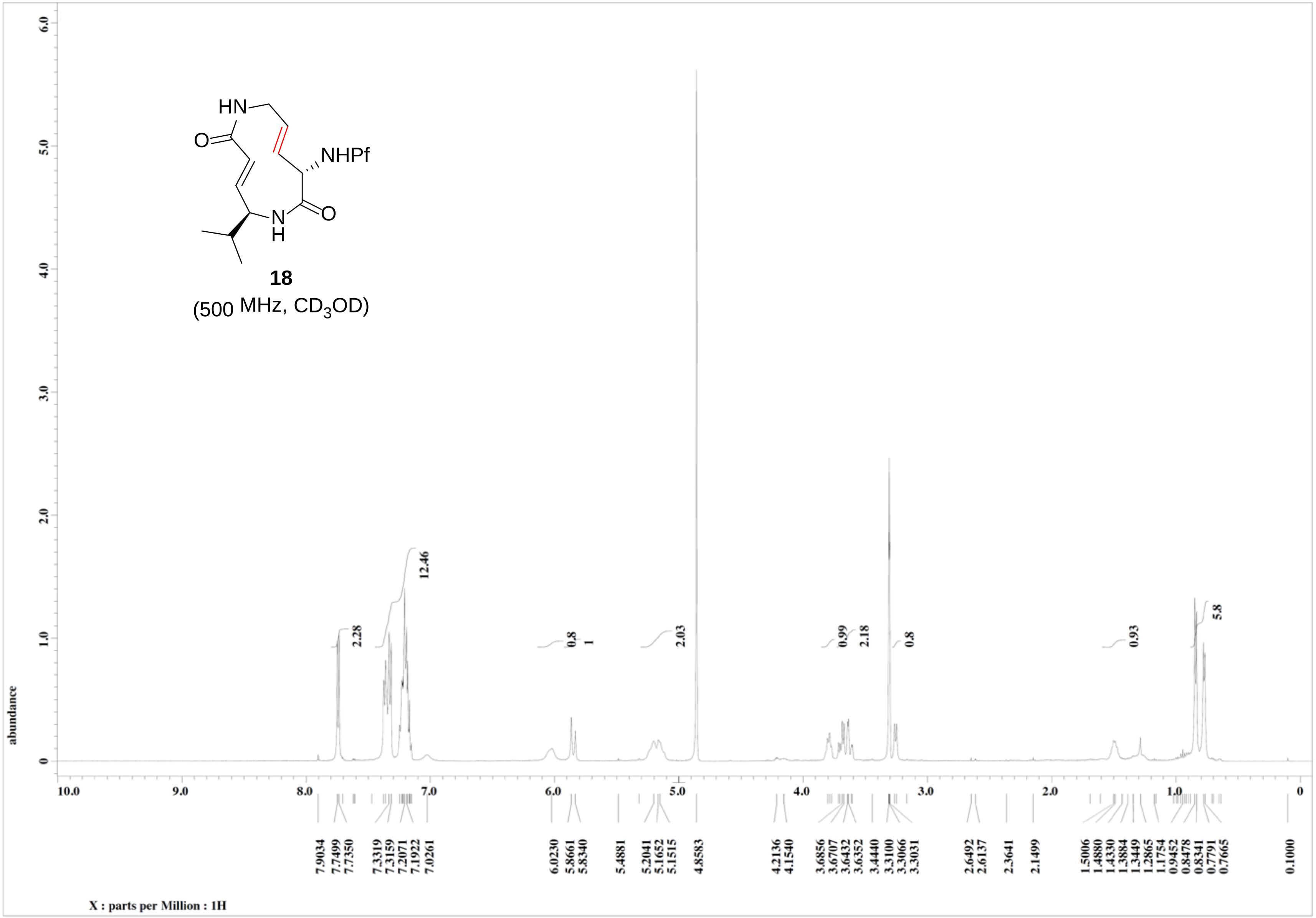
HN
O
NHPf
N
H
O
18
(500 MHz, CD$_3$OD)
abundance
X : parts per Million : 1H
2.28
12.46
0.8
1
2.03
0.99
2.18
0.8
0.93
5.8
7.9034
7.7499
7.7350
7.3319
7.3159
7.2071
7.1922
7.0261
6.0230
5.8661
5.8340
5.4881
5.2041
5.1652
5.1515
4.8583
4.2136
4.1540
3.6856
3.6707
3.6432
3.6352
3.4440
3.3100
3.3066
3.3031
2.6492
2.6137
2.3641
2.1499
1.5006
1.4880
1.4330
1.3884
1.3449
1.2865
1.1754
0.9452
0.8478
0.8341
0.7791
0.7665
0.1000

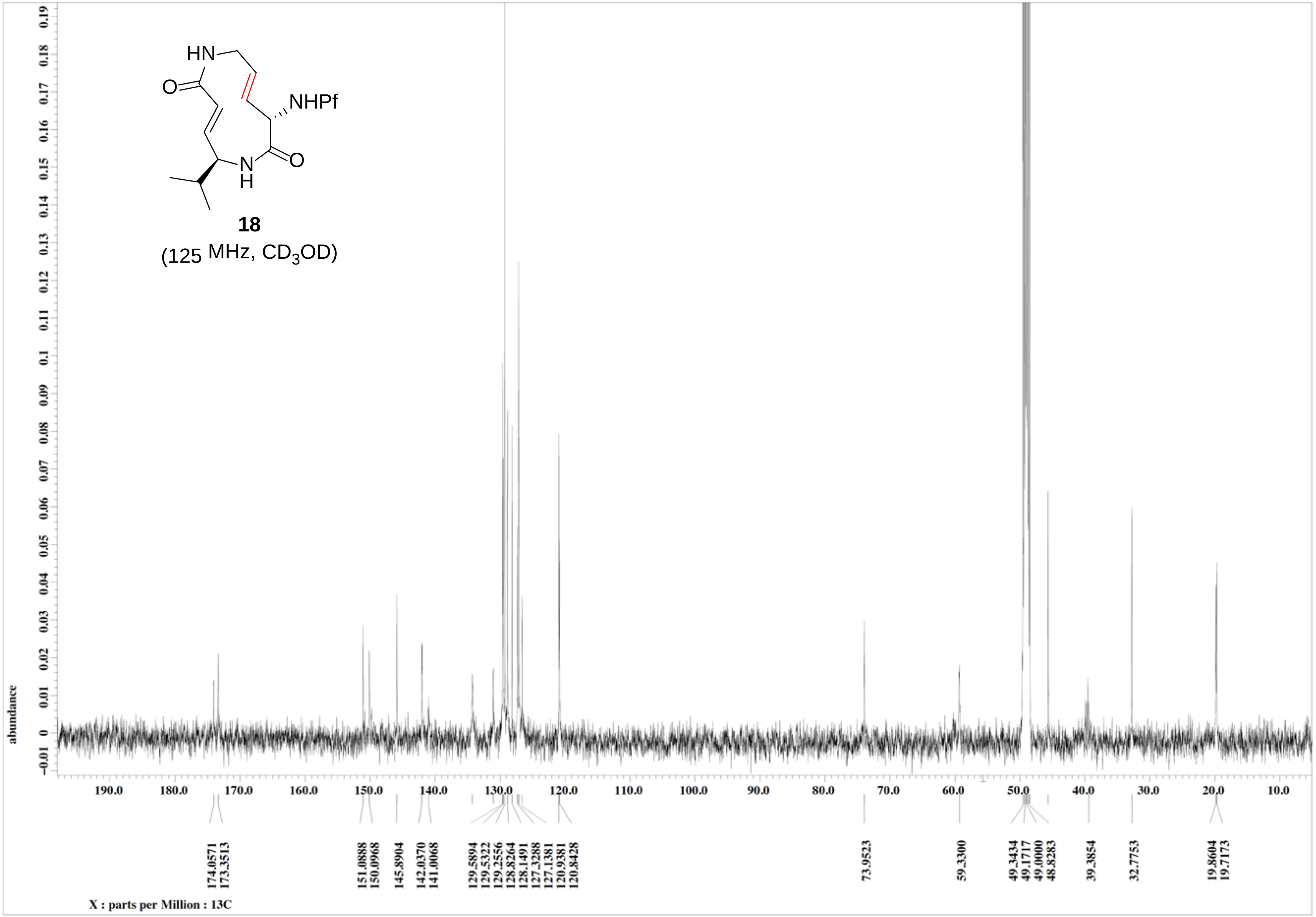
HN
O
NHPf
N
H
O
18
(125 MHz, CD3OD)
abundance
X : parts per Million : 13C
174.0571
173.3513
151.0888
150.0968
145.8904
142.0370
141.0068
129.5894
129.5322
129.2556
128.8264
128.1491
127.3288
127.1381
120.9381
120.8428
73.9523
59.3300
49.3434
49.1717
49.0000
48.8283
39.3854
32.7753
19.8604
19.7173

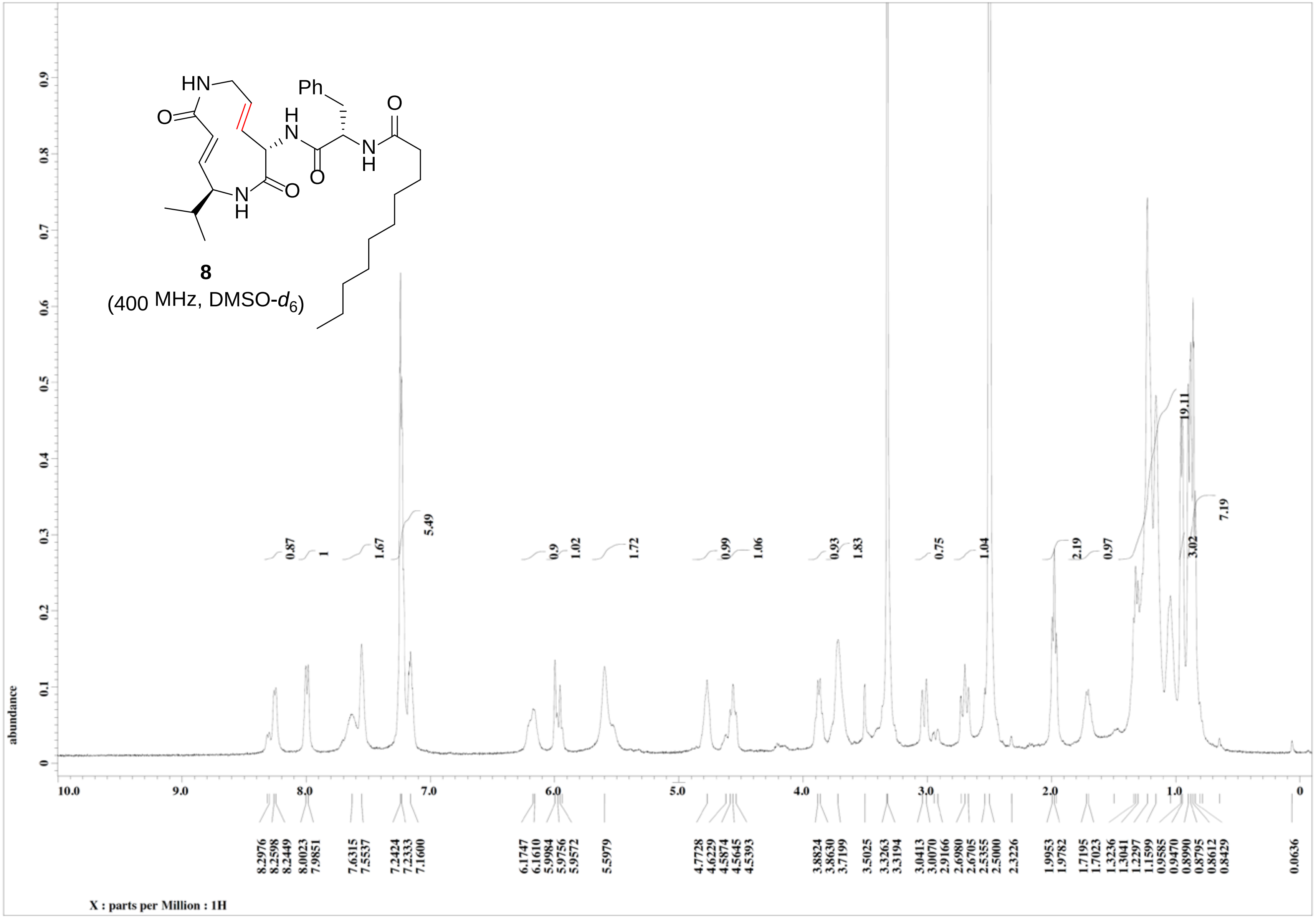
HN
O
Ph
O
H
N
N
H
O
N
H
O
8
(400 MHz, DMSO-d_6)
abundance
X : parts per Million : 1H

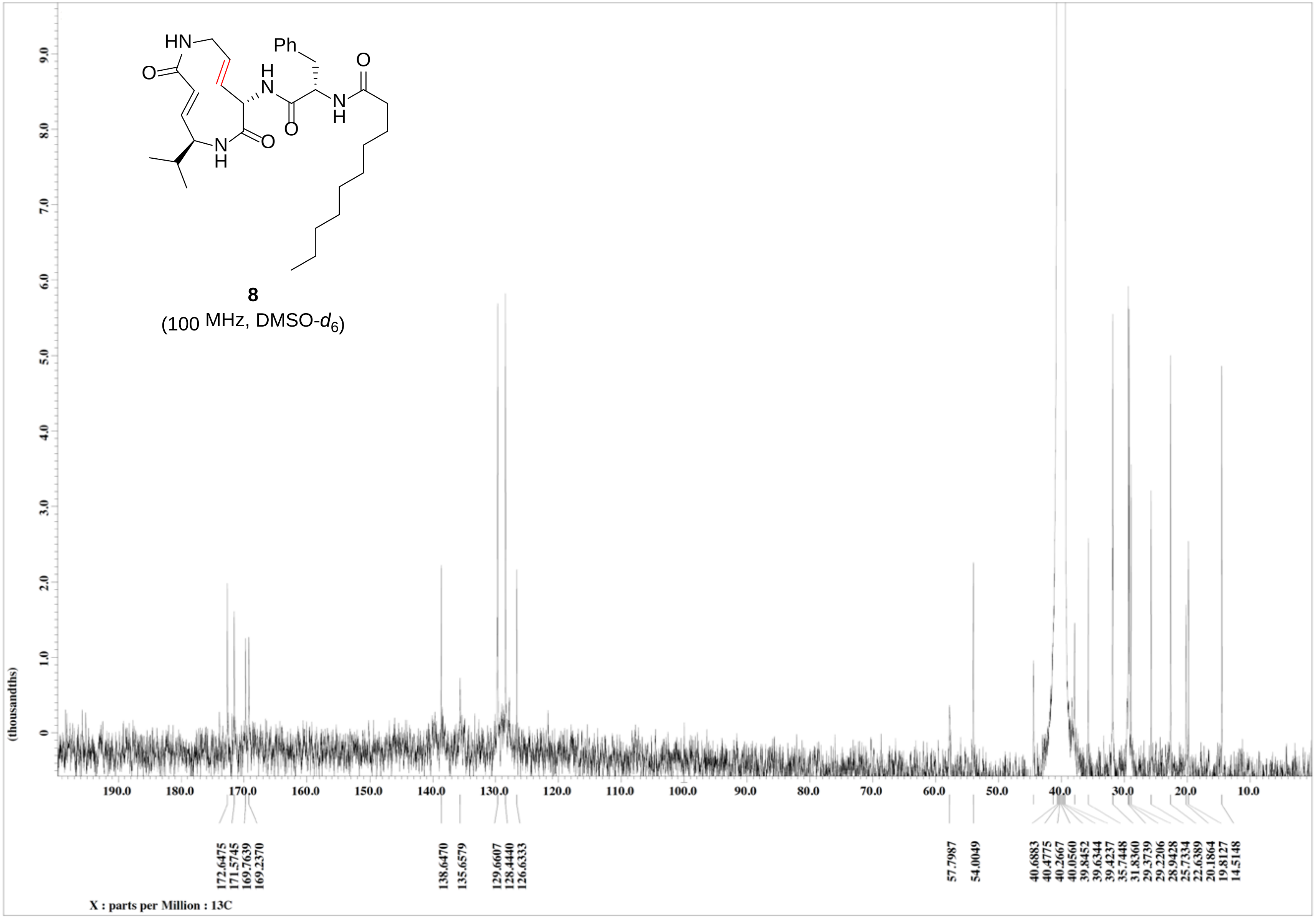
HN
Ph
O
H
N
O
N
H
N
H
O
O
8
(100 MHz, DMSO-d_6)
(thousandths)
X : parts per Million : 13C
172.6475
171.5745
169.7639
169.2370
138.6470
135.6579
129.6607
128.4440
126.6333
57.7987
54.0049
40.6883
40.4775
40.2667
40.0560
39.8452
39.6344
39.4237
35.7448
31.8360
29.3739
29.2206
28.9428
25.7334
22.6389
20.1864
19.8127
14.5148

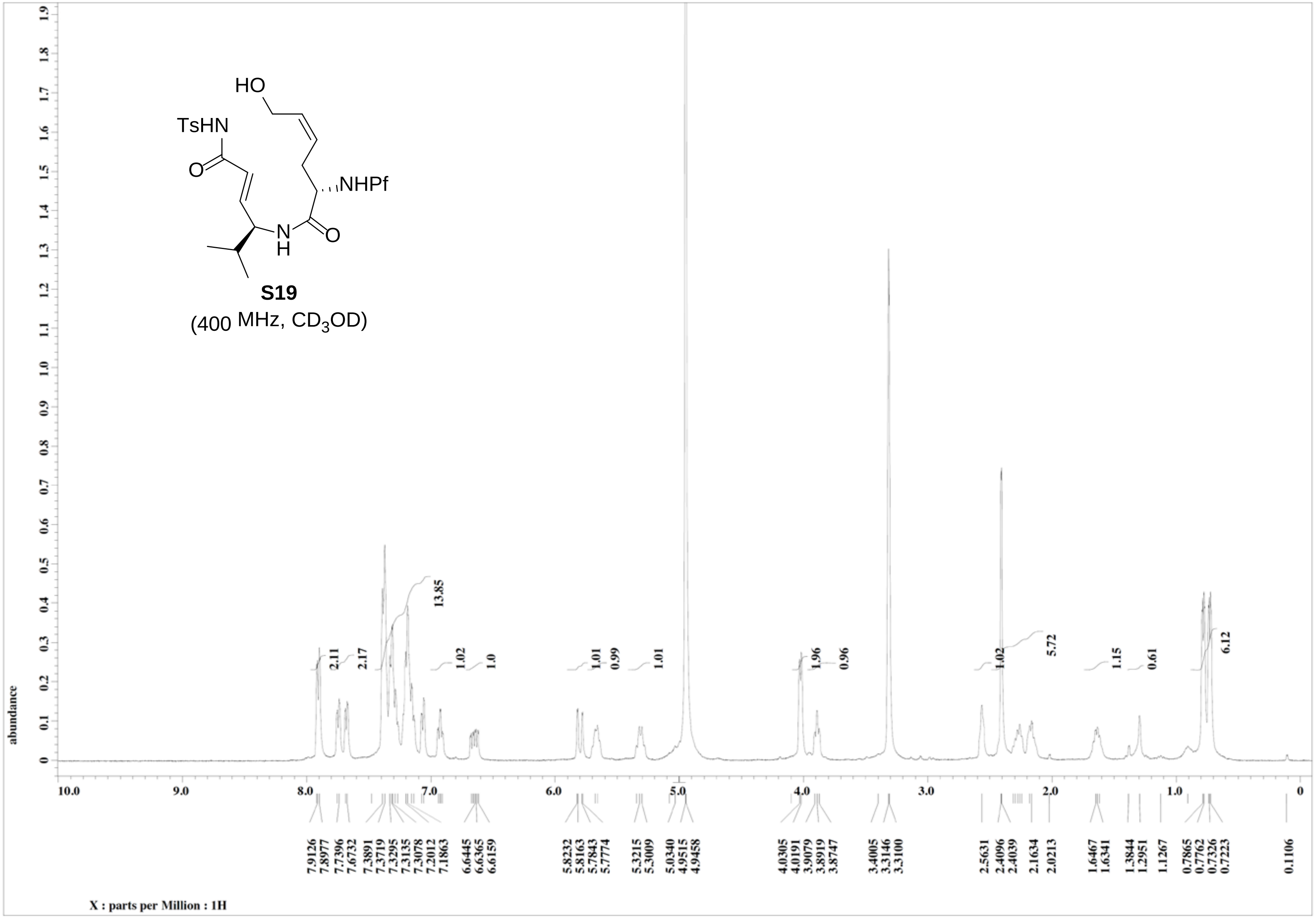

S19

(400 MHz, CD_3OD)

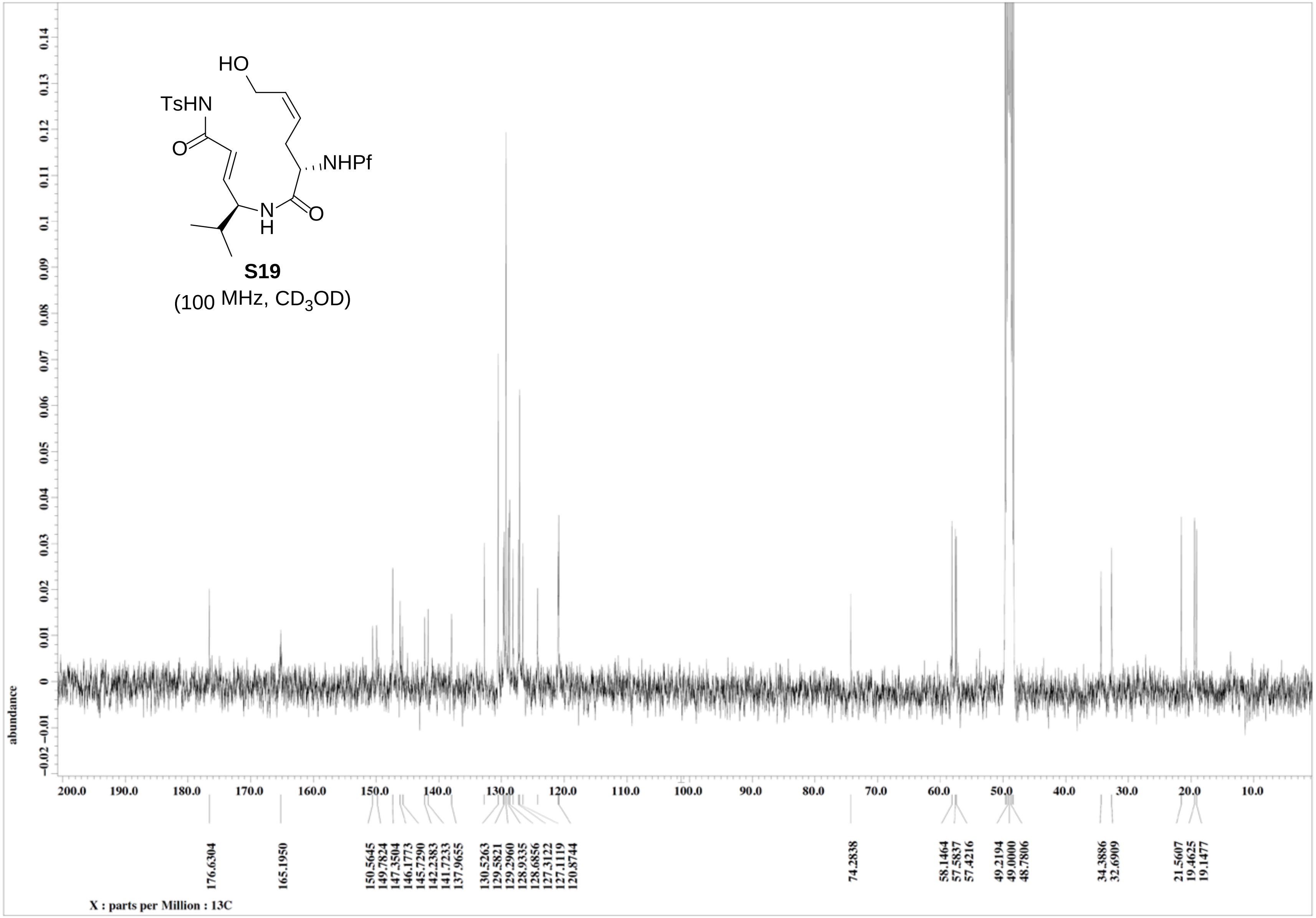
HO
TsHN
O
NHPf
N
H
O
S19
(100 MHz, CD3OD)
abundance
X : parts per Million : 13C
176.6304
165.1950
150.5645
149.7824
147.3504
146.1773
145.7290
142.2383
141.7233
137.9655
130.5263
129.5821
129.2960
128.9335
128.6856
127.3122
127.1119
120.8744
74.2838
58.1464
57.5837
57.4216
49.2194
49.0000
48.7806
34.3886
32.6909
21.5607
19.4625
19.1477

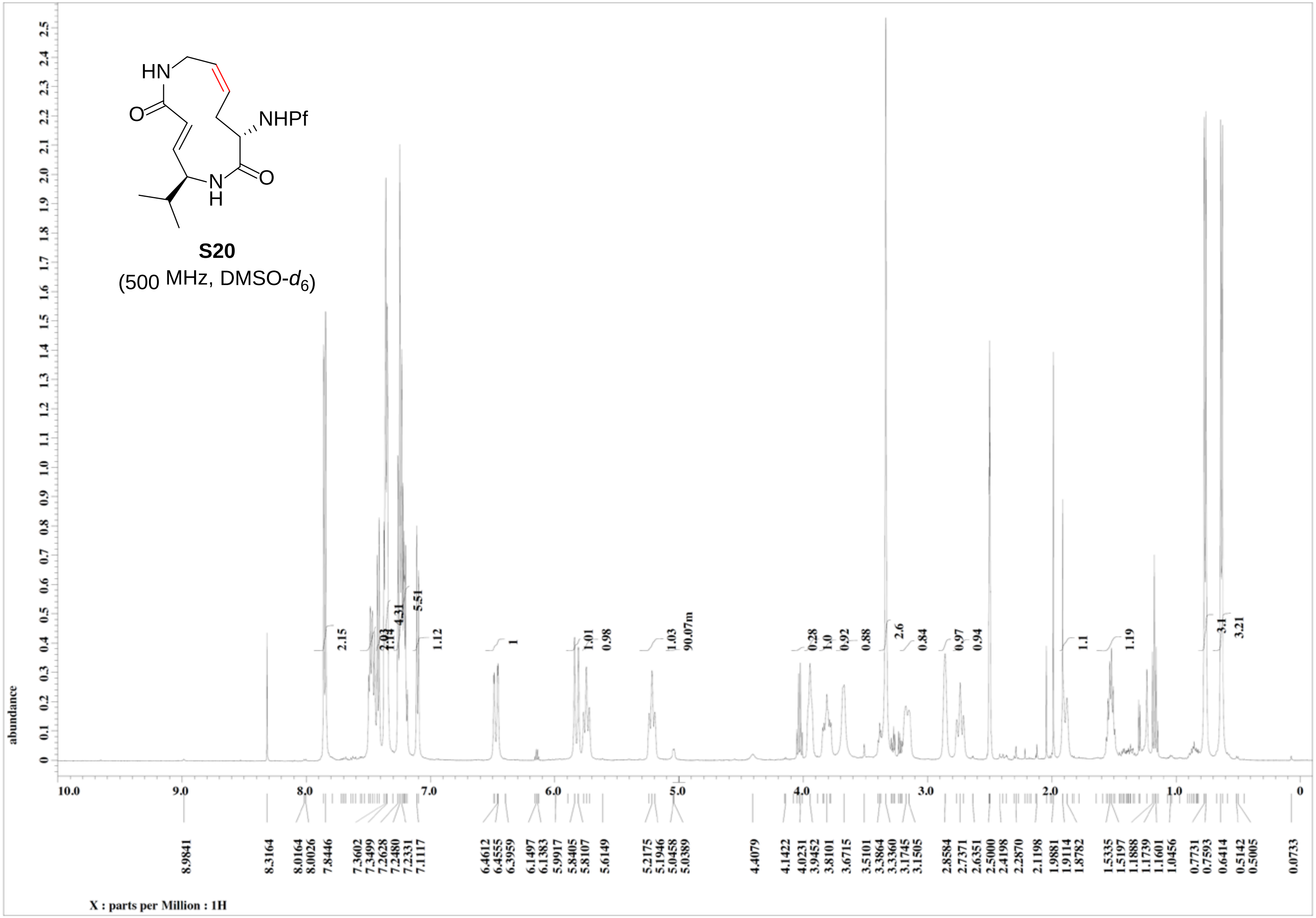
HN
O
NHPf
N
H
O
S20
(500 MHz, DMSO-d_6)
abundance
X : parts per Million : 1H

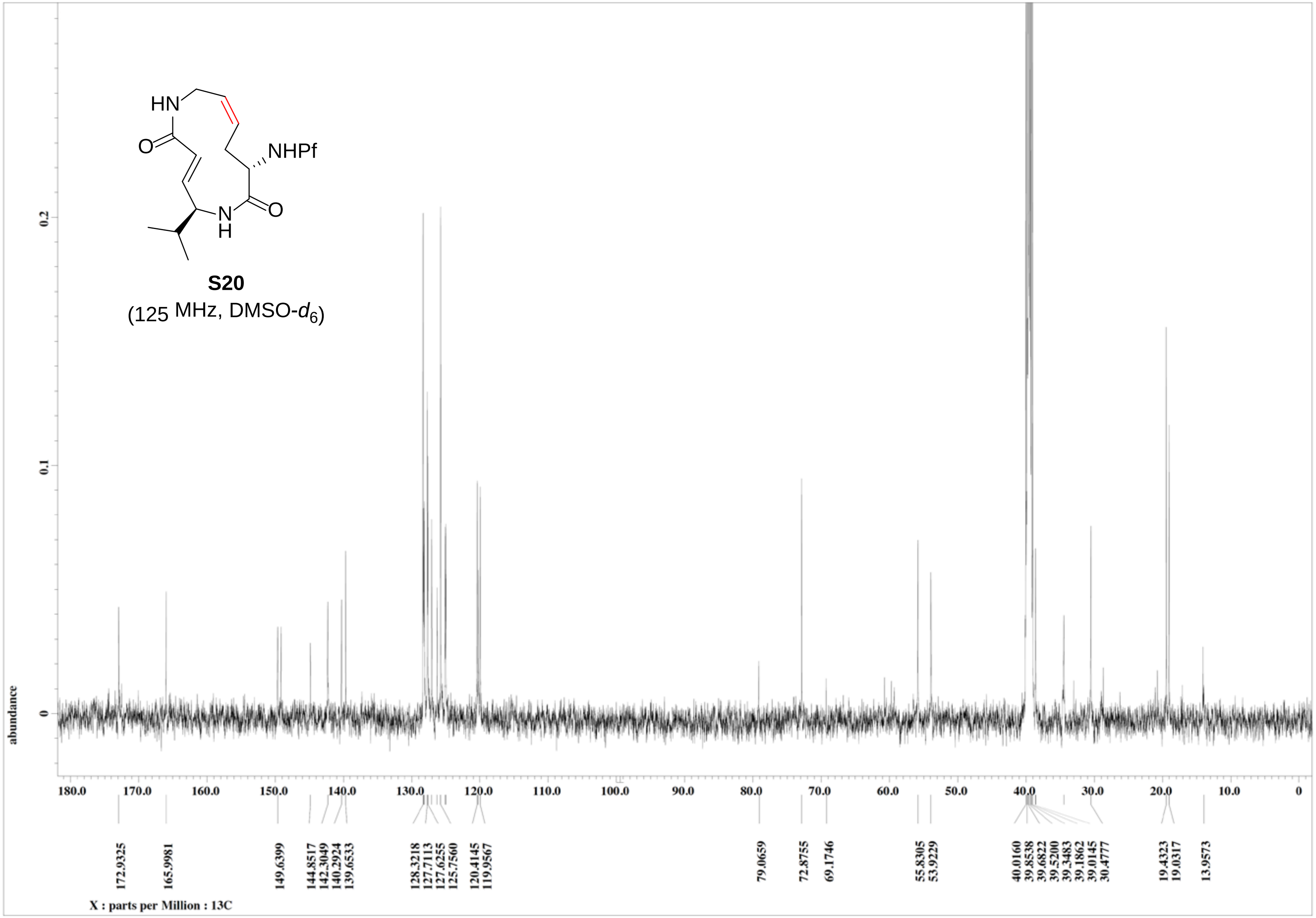
HN
O
NHPf
N
H
O
S20
(125 MHz, DMSO-d_6)
abundance
0.2
0.1
0
180.0
170.0
160.0
150.0
140.0
130.0
120.0
110.0
100.0
90.0
80.0
70.0
60.0
50.0
40.0
30.0
20.0
10.0
0
172.9325
165.9981
149.6399
144.8517
142.3049
140.2924
139.6533
128.3218
127.7113
127.6255
125.7560
120.4145
119.9567
79.0659
72.8755
69.1746
55.8305
53.9229
40.0160
39.8538
39.6822
39.5200
39.3483
39.1862
39.0145
30.4777
19.4323
19.0317
13.9573
X : parts per Million : 13C

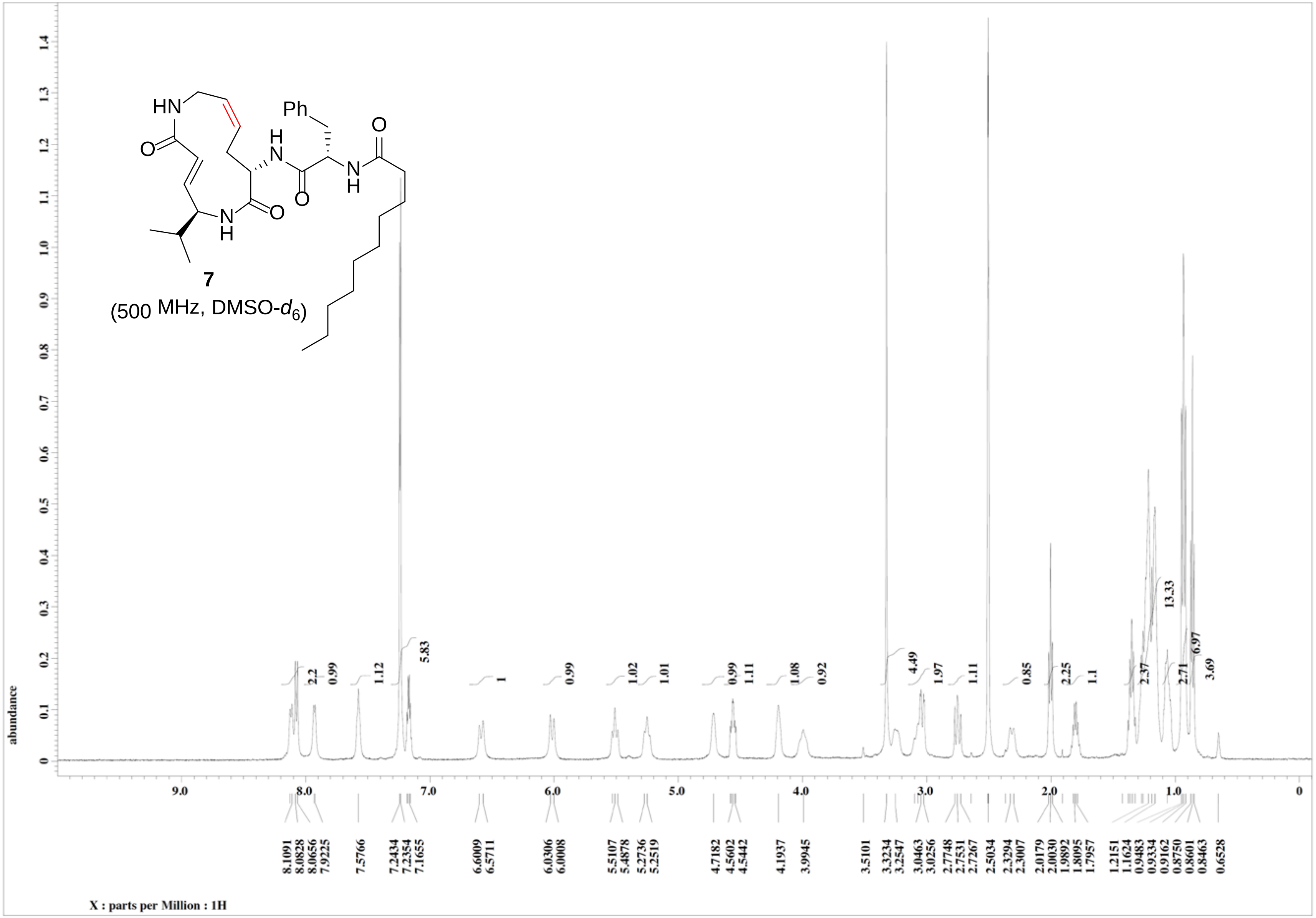

HN
Ph
O
H
N
O
N
H
O
N
H
O
7
(500 MHz, DMSO-d_6)
abundance
X : parts per Million : 1H
9.0
8.0
7.0
6.0
5.0
4.0
3.0
2.0
1.0
0
2.2
0.99
1.12
5.83
1
0.99
1.02
1.01
0.99
1.11
1.08
0.92
4.49
1.97
1.11
0.85
2.25
1.1
2.37
13.33
2.71
6.97
3.69
8.1091
8.0828
8.0656
7.9225
7.5766
7.2434
7.2354
7.1655
6.6009
6.5711
6.0306
6.0008
5.5107
5.4878
5.2736
5.2519
4.7182
4.5602
4.5442
4.1937
3.9945
3.5101
3.3234
3.2547
3.0463
3.0256
2.7748
2.7531
2.7267
2.5034
2.3294
2.3007
2.0179
2.0030
1.9892
1.8095
1.7957
1.2151
1.1624
0.9483
0.9334
0.9162
0.8750
0.8601
0.8463
0.6528

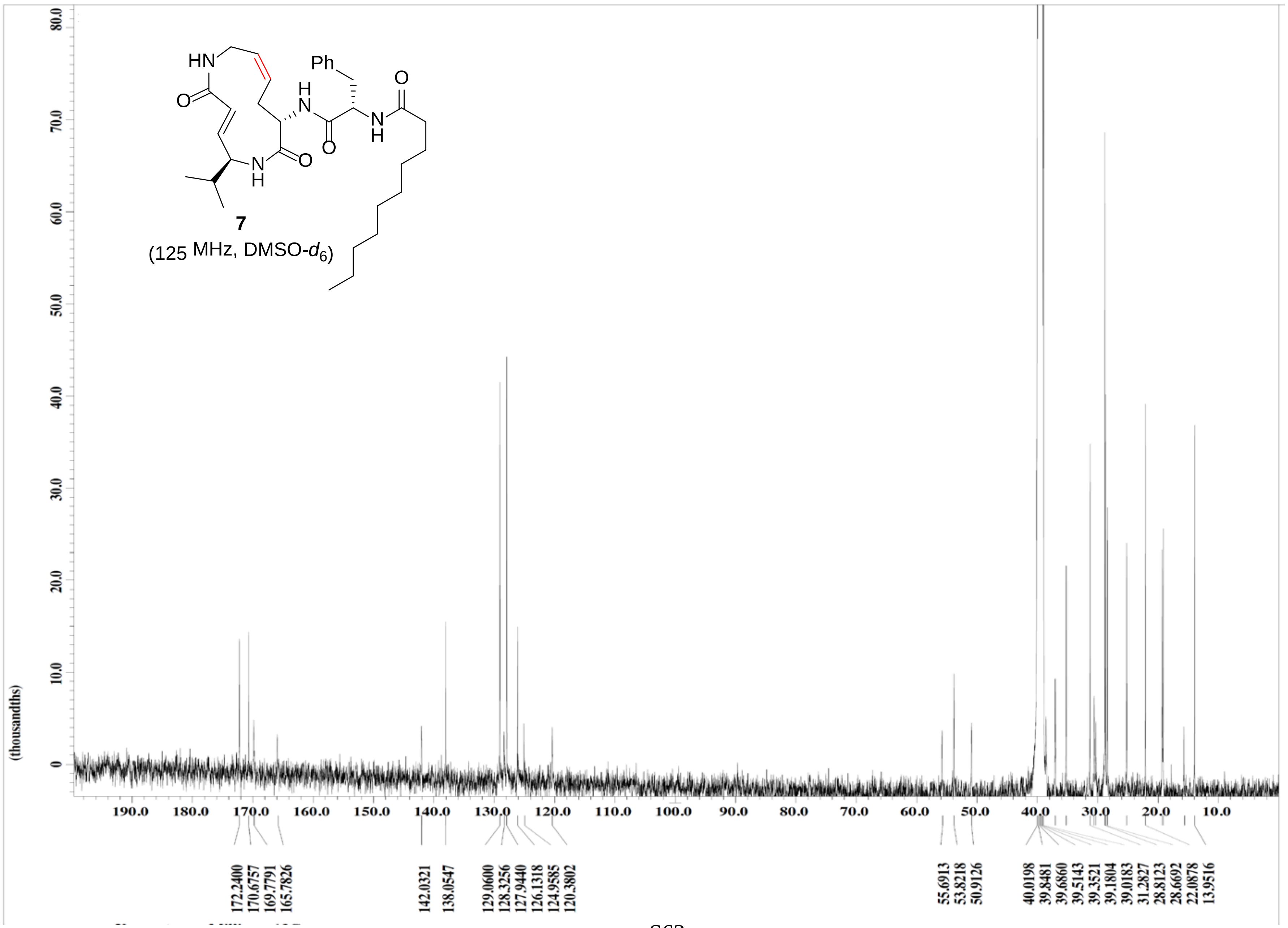
Ph
HN
O
N
H
O
N
H
O
N
H
O
7
(125 MHz, DMSO-d6)
(thousandths)
172.2400
170.6757
169.7791
165.7826
142.0321
138.0547
129.0600
128.3256
127.9440
126.1318
124.9585
120.3802
55.6913
53.8218
50.9126
40.0198
39.8481
39.6860
39.5143
39.3521
39.1804
39.0183
31.2827
28.8123
28.6692
22.0878
13.9516

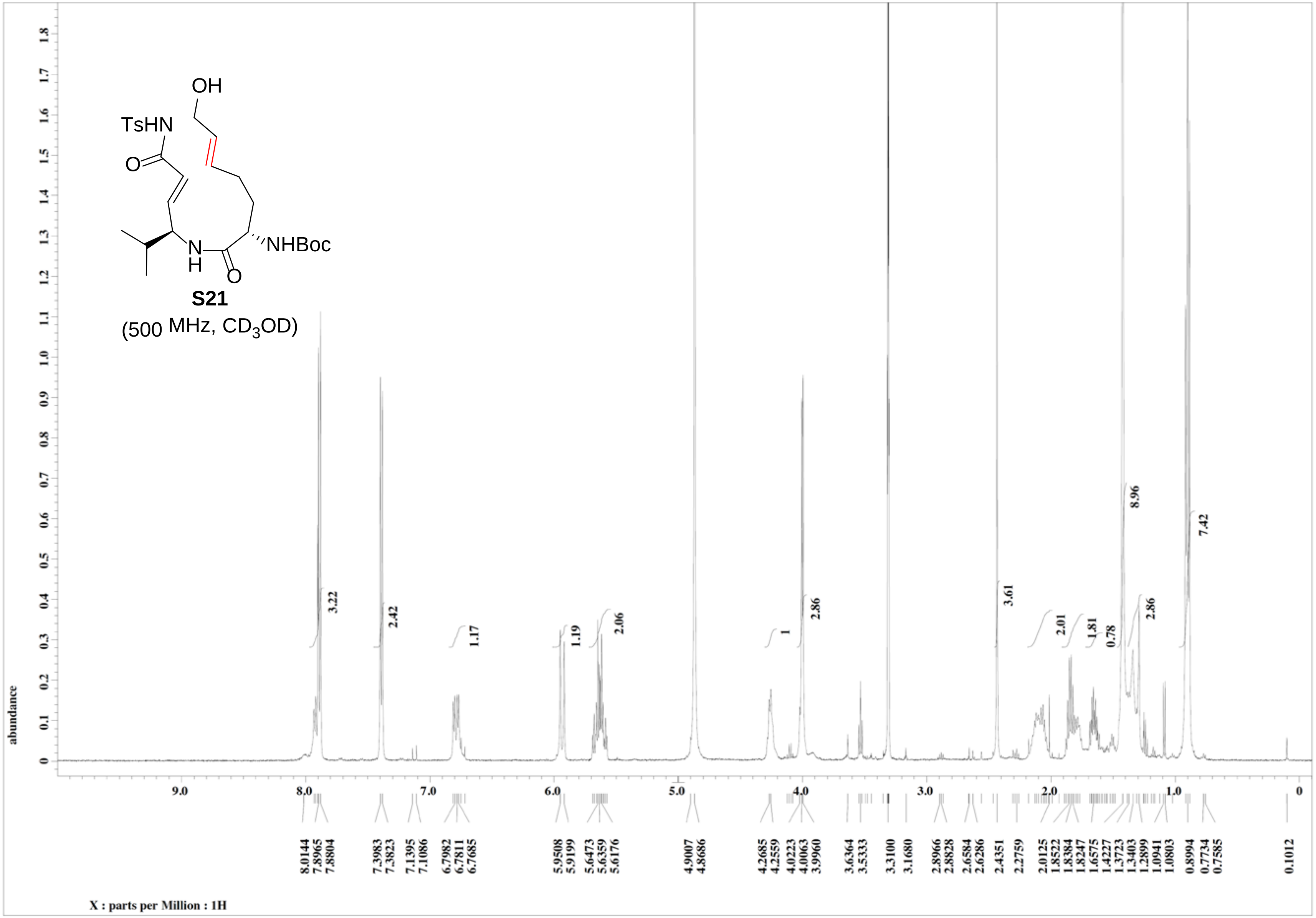
OH
TsHN
O
N
H
O
NHBoc
S21
(500 MHz, CD3OD)
abundance
X : parts per Million : 1H
3.22
2.42
1.17
1.19
2.06
1
2.86
3.61
2.01
1.81
0.78
8.96
2.86
7.42

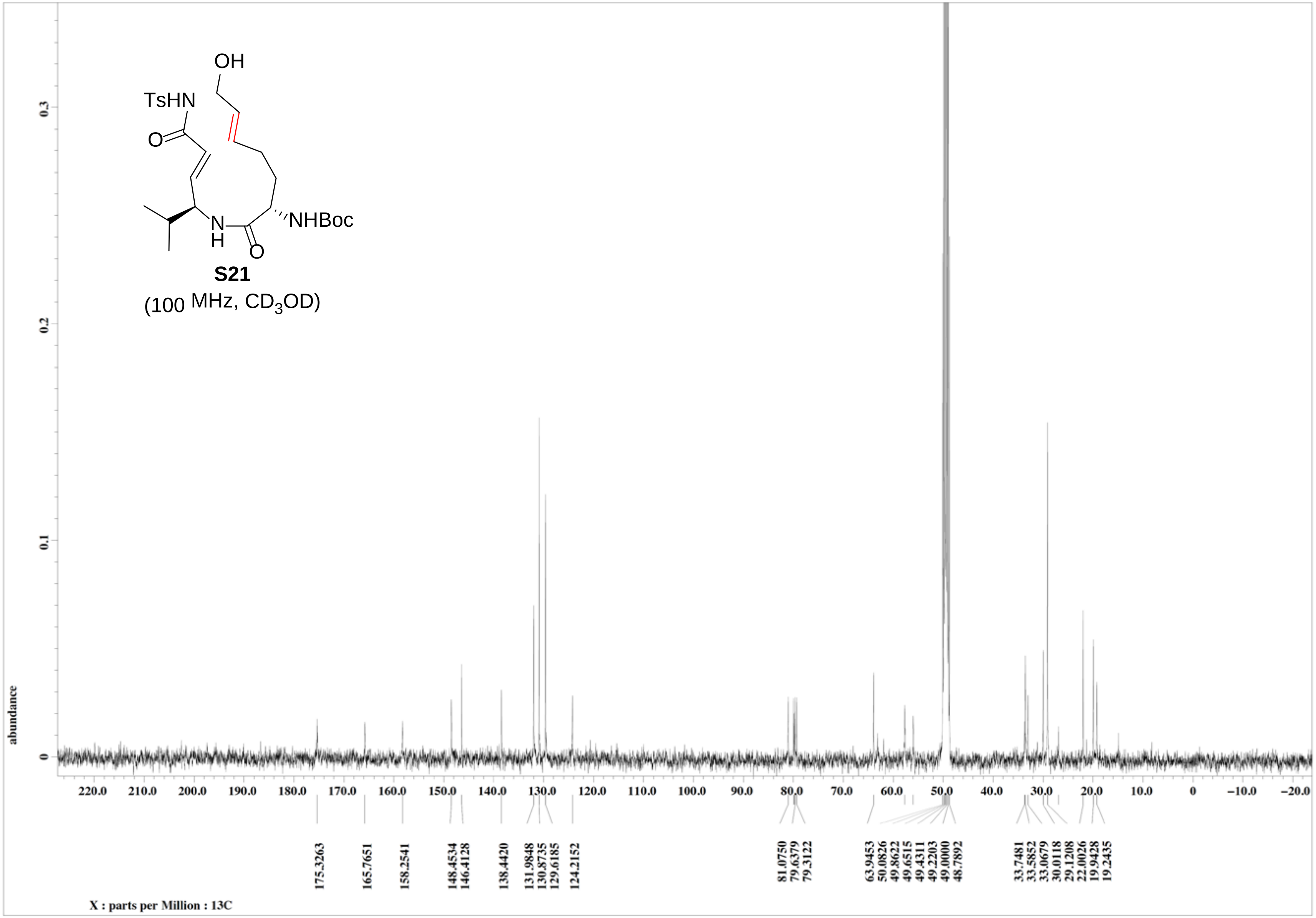

OH
TsHN
O
N
H
O
NHBoc
S21
(100 MHz, CD3OD)
abundance
0.3
0.2
0.1
0
220.0 210.0 200.0 190.0 180.0 170.0 160.0 150.0 140.0 130.0 120.0 110.0 100.0 90.0 80.0 70.0 60.0 50.0 40.0 30.0 20.0 10.0 0 −10.0 −20.0
175.3263
165.7651
158.2541
148.4534
146.4128
138.4420
131.9848
130.8735
129.6185
124.2152
81.0750
79.6379
79.3122
63.9453
50.0826
49.8622
49.6515
49.4311
49.2203
49.0000
48.7892
33.7481
33.5852
33.0679
30.0118
29.1208
22.0026
19.9428
19.2435
X : parts per Million : 13C

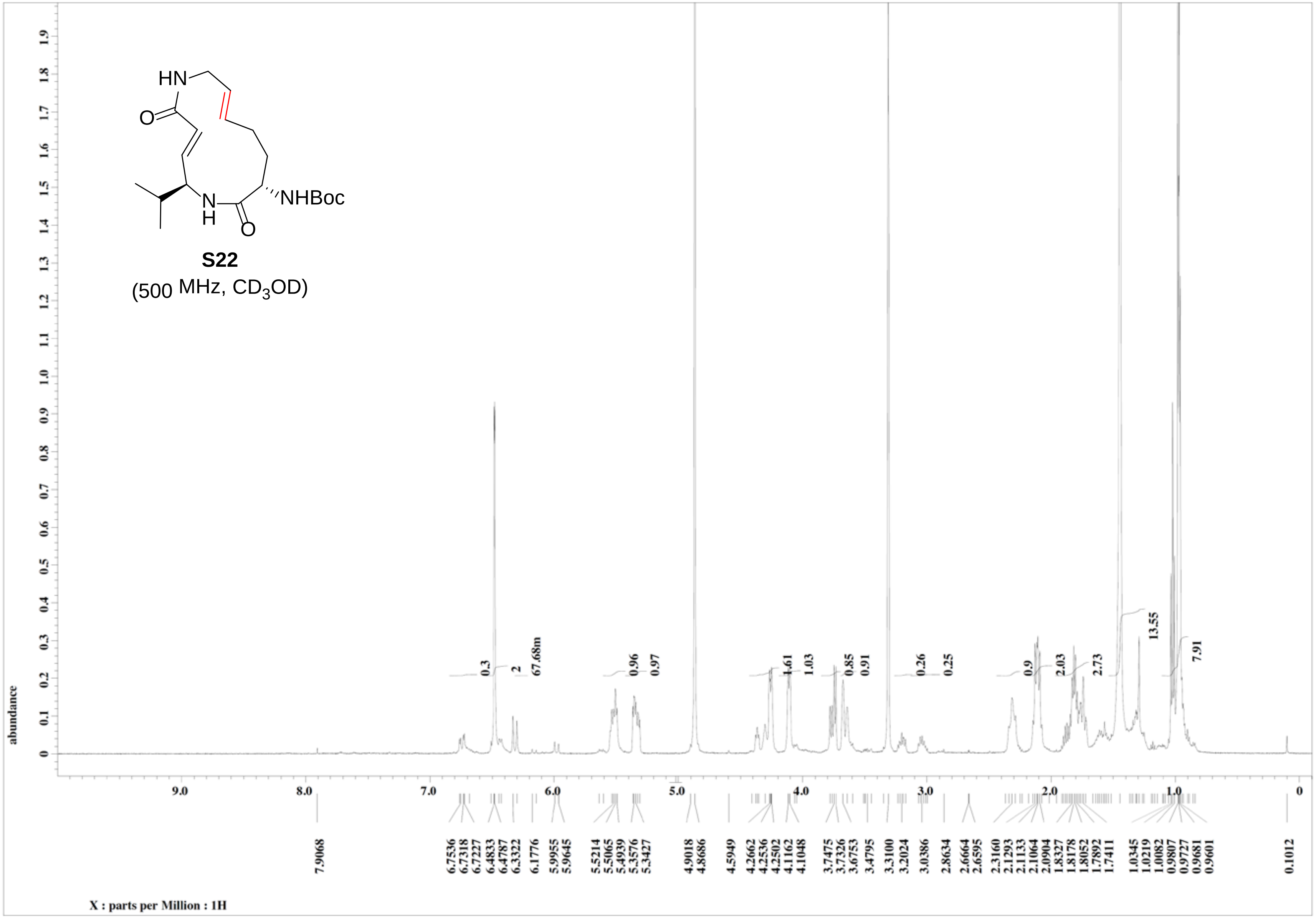
HN
O
NHBoc
N
H
O
S22
(500 MHz, CD3OD)
abundance
X : parts per Million : 1H
0.3
2
67.68m
0.96
0.97
1.61
1.03
0.85
0.91
0.26
0.25
0.9
2.03
2.73
13.55
7.91
7.9068
6.7536
6.7318
6.7227
6.4833
6.4787
6.3322
6.1776
5.9955
5.9645
5.5214
5.5065
5.4939
5.3576
5.3427
4.9018
4.8686
4.5949
4.2662
4.2536
4.2502
4.1162
4.1048
3.7475
3.7326
3.6753
3.4795
3.3100
3.2024
3.0386
2.8634
2.6664
2.6595
2.3160
2.1293
2.1133
2.1064
2.0904
1.8327
1.8178
1.8052
1.7892
1.7411
1.0345
1.0219
1.0082
0.9807
0.9727
0.9681
0.9601
0.1012

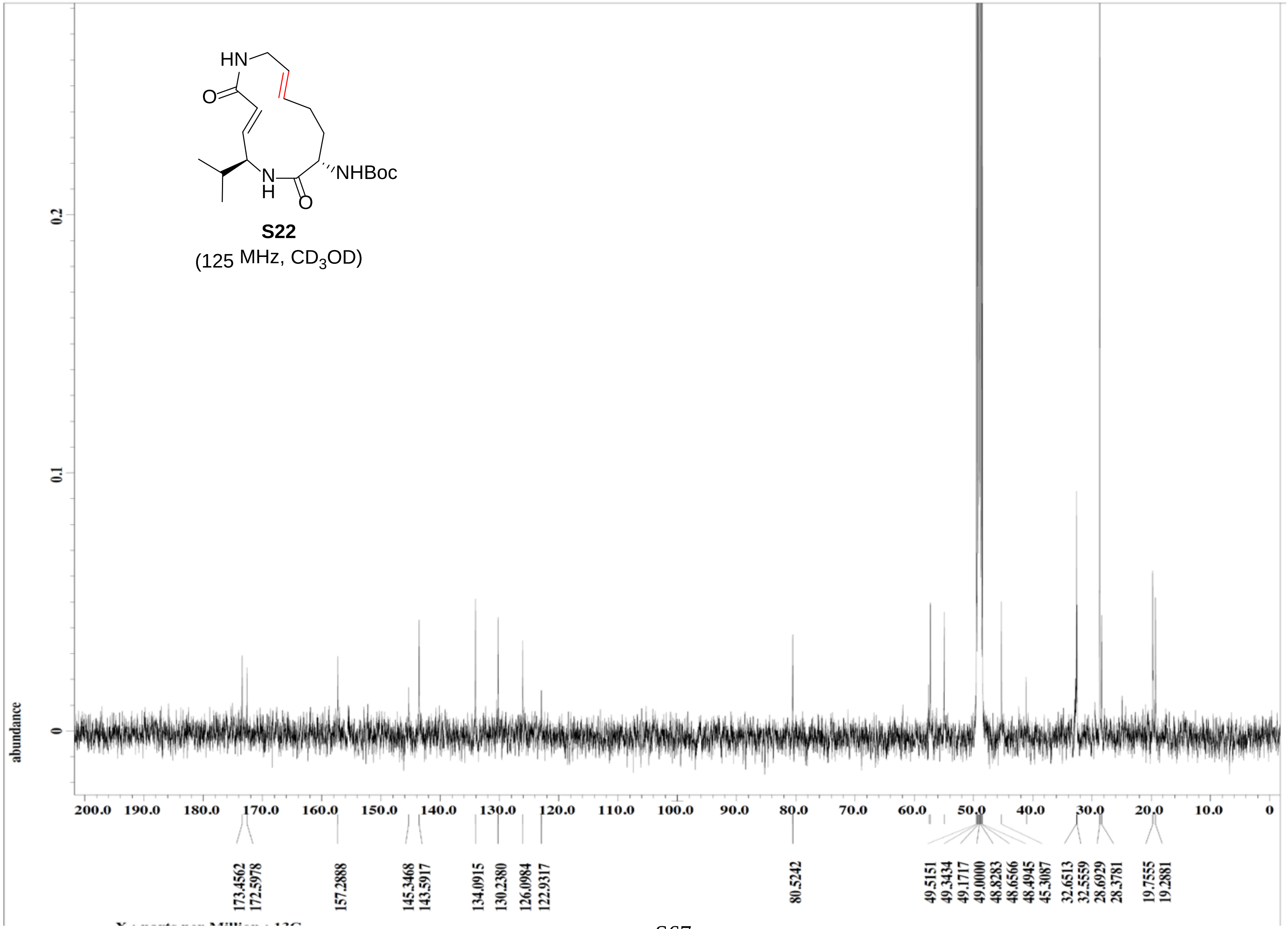
HN
O
NHBoc
N
H
O
S22
(125 MHz, CD3OD)
abundance
0.2
0.1
0
200.0 190.0 180.0 170.0 160.0 150.0 140.0 130.0 120.0 110.0 100.0 90.0 80.0 70.0 60.0 50.0 40.0 30.0 20.0 10.0 0
173.4562
172.5978
157.2888
145.3468
143.5917
134.0915
130.2380
126.0984
122.9317
80.5242
49.5151
49.3434
49.1717
49.0000
48.8283
48.6566
48.4945
45.3087
32.6513
32.5559
28.6929
28.3781
19.7555
19.2881

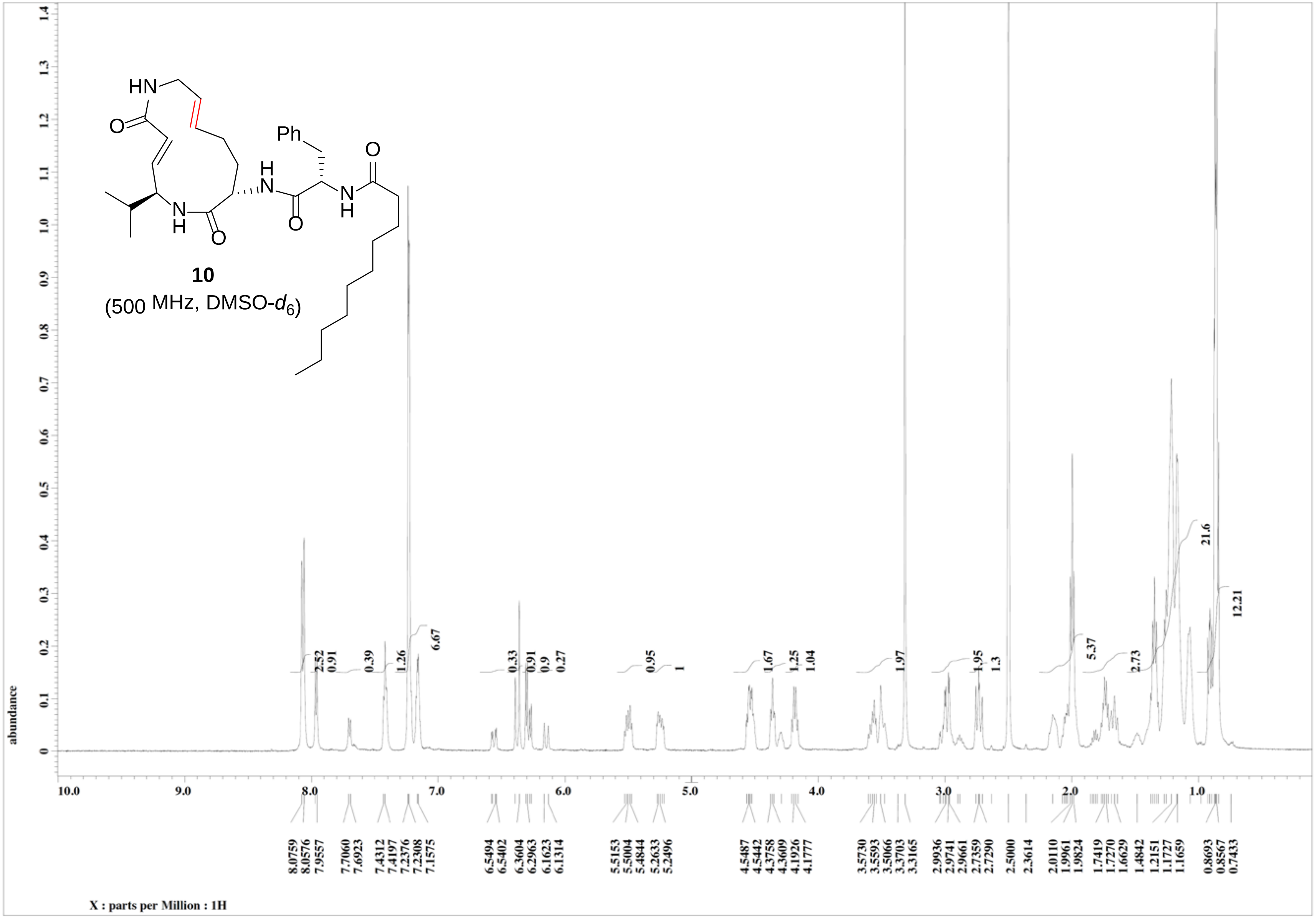

10
(500 MHz, DMSO-d6)
abundance
X : parts per Million : 1H

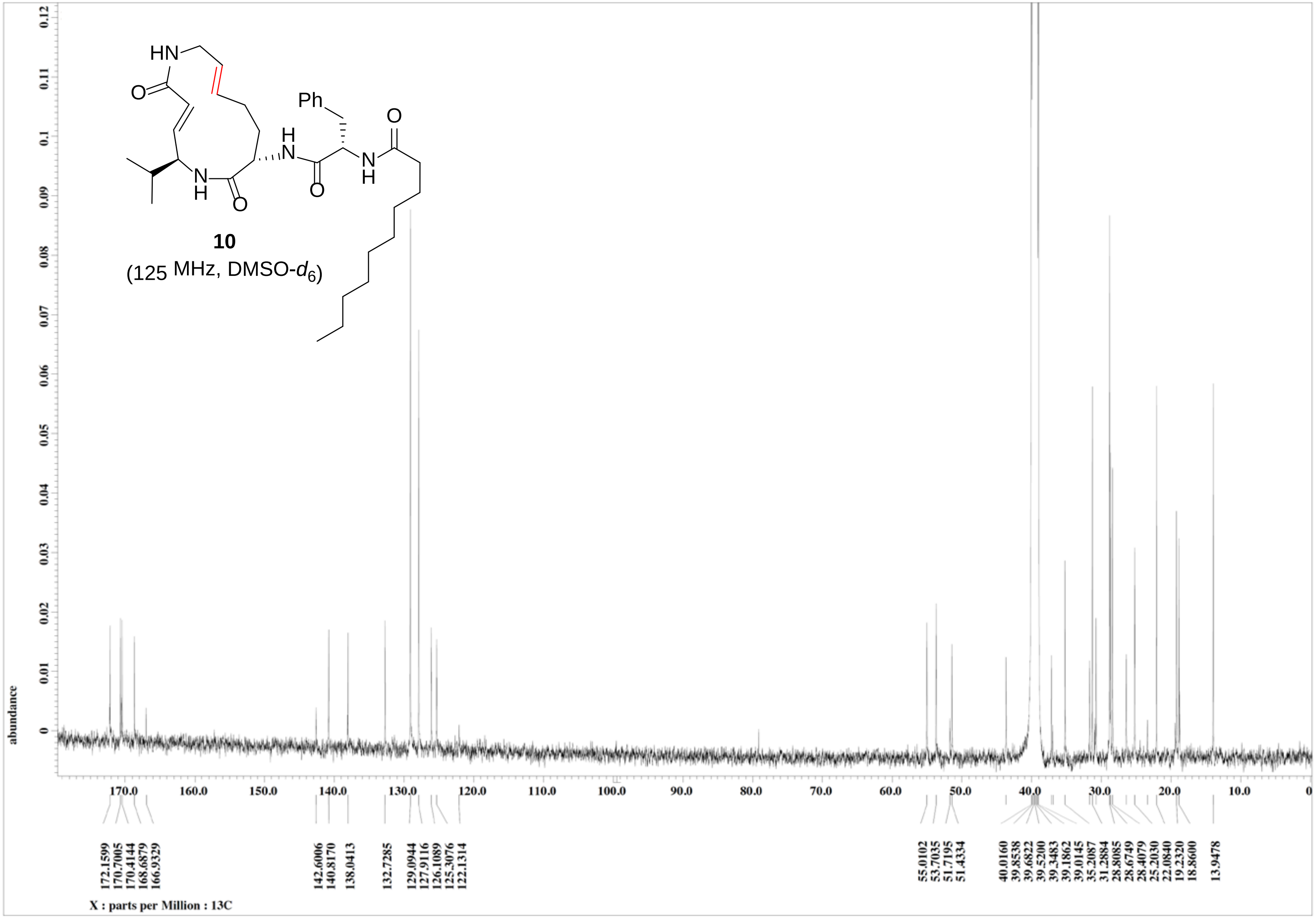
HN
O
Ph
H
N
O
N
H
N
H
O
O
10
(125 MHz, DMSO-d_6)
abundance
X : parts per Million : 13C
172.1599
170.7005
170.4144
168.6879
166.9329
142.6006
140.8170
138.0413
132.7285
129.0944
127.9116
126.1089
125.3076
122.1314
55.0102
53.7035
51.7195
51.4334
40.0160
39.8538
39.6822
39.5200
39.3483
39.1862
39.0145
35.2087
31.2884
28.8085
28.6749
28.4079
25.2030
22.0840
19.2320
18.8600
13.9478

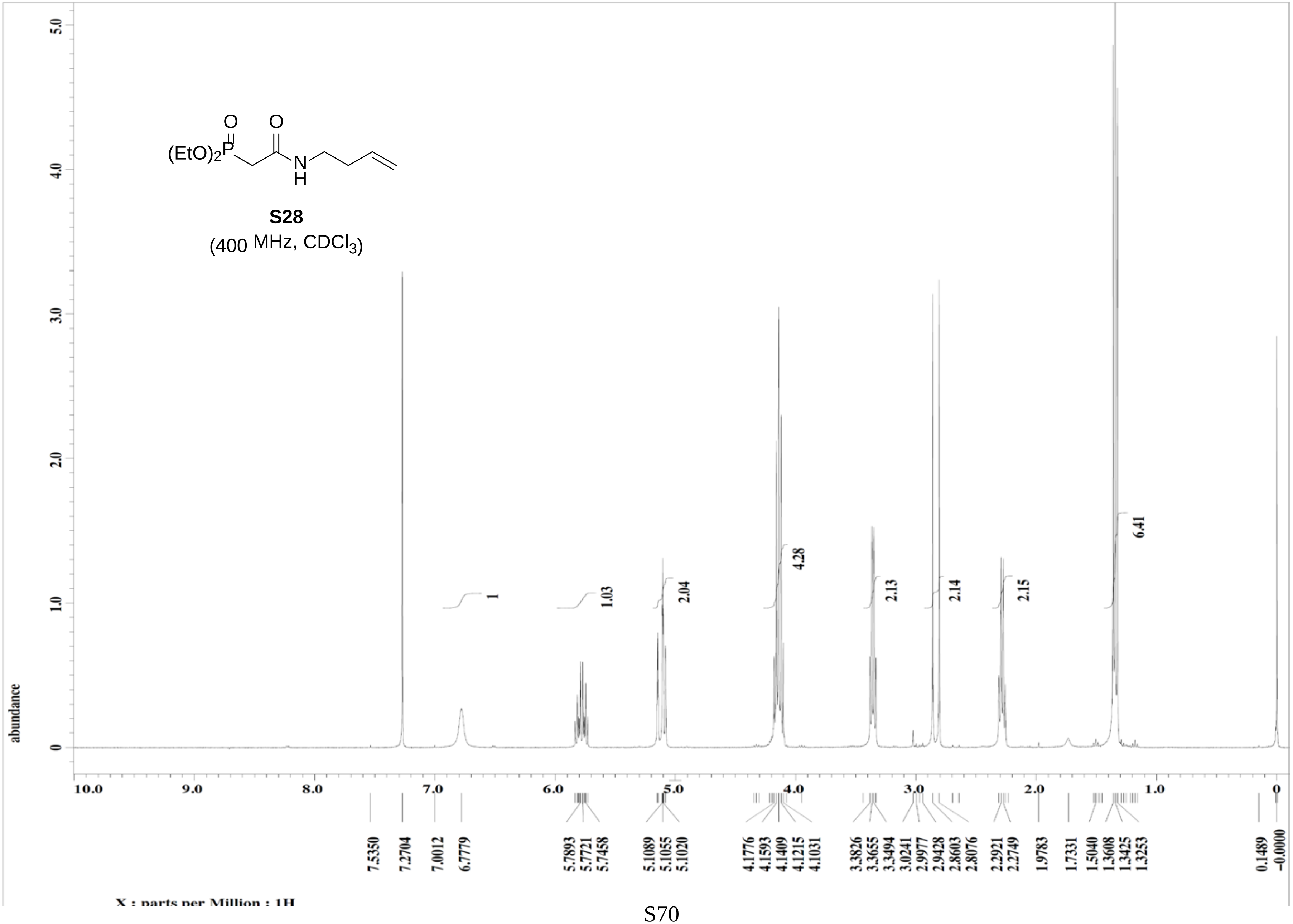
(EtO)2P
O
O
N
H
S28
(400 MHz, CDCl3)
abundance
X : parts per Million : 1H
1
1.03
2.04
4.28
2.13
2.14
2.15
6.41
7.5350
7.2704
7.0012
6.7779
5.7893
5.7721
5.7458
5.1089
5.1055
5.1020
4.1776
4.1593
4.1409
4.1215
4.1031
3.3826
3.3655
3.3494
3.0241
2.9977
2.9428
2.8603
2.8076
2.2921
2.2749
1.9783
1.7331
1.5040
1.3608
1.3425
1.3253
0.1489
-0.0000

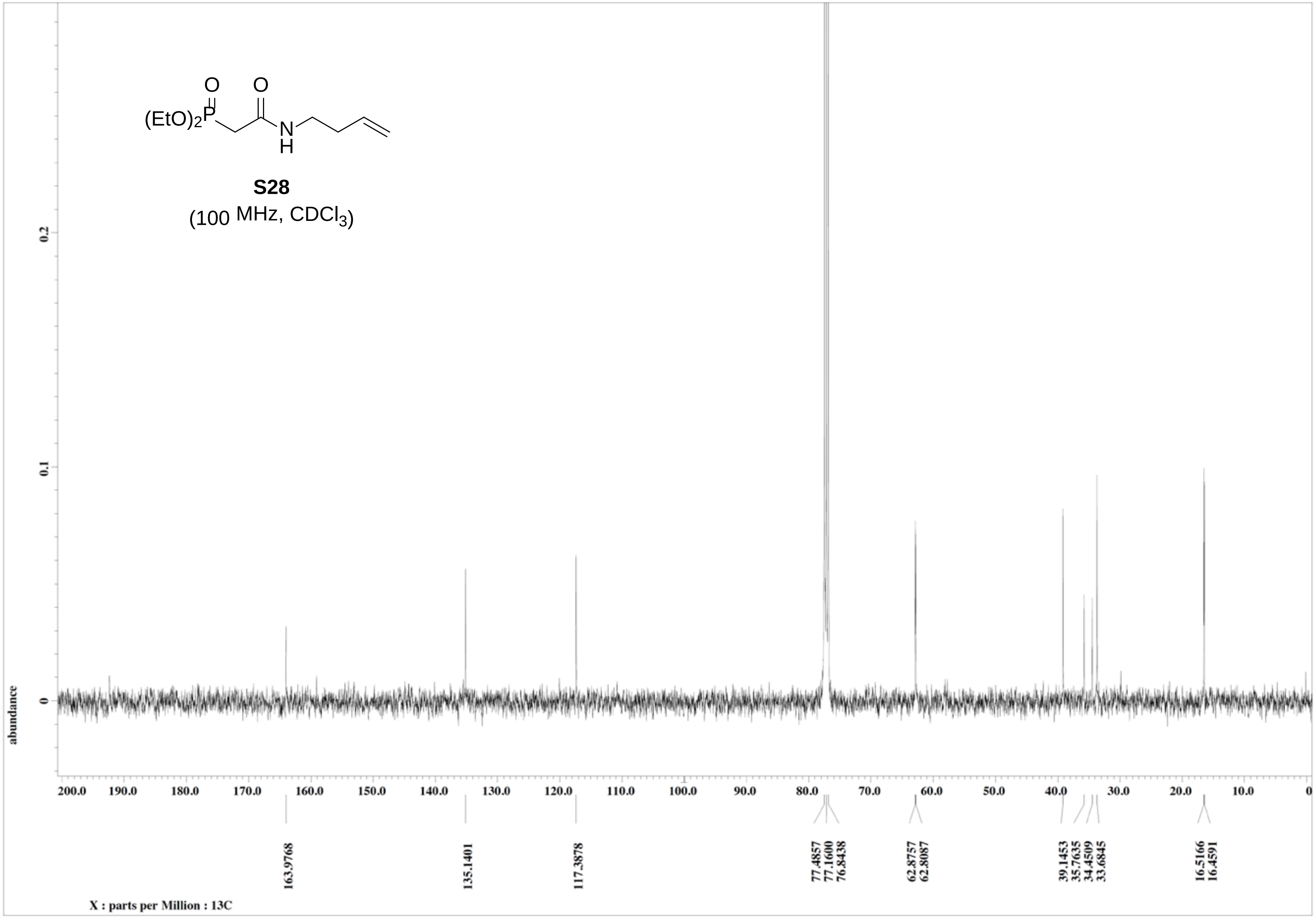
(EtO)2P
O
O
N
H
S28
(100 MHz, CDCl3)
abundance
0.2
0.1
0
200.0 190.0 180.0 170.0 160.0 150.0 140.0 130.0 120.0 110.0 100.0 90.0 80.0 70.0 60.0 50.0 40.0 30.0 20.0 10.0 0
163.9768
135.1401
117.3878
77.4857
77.1600
76.8438
62.8757
62.8087
39.1453
35.7635
34.4509
33.6845
16.5166
16.4591
X : parts per Million : 13C

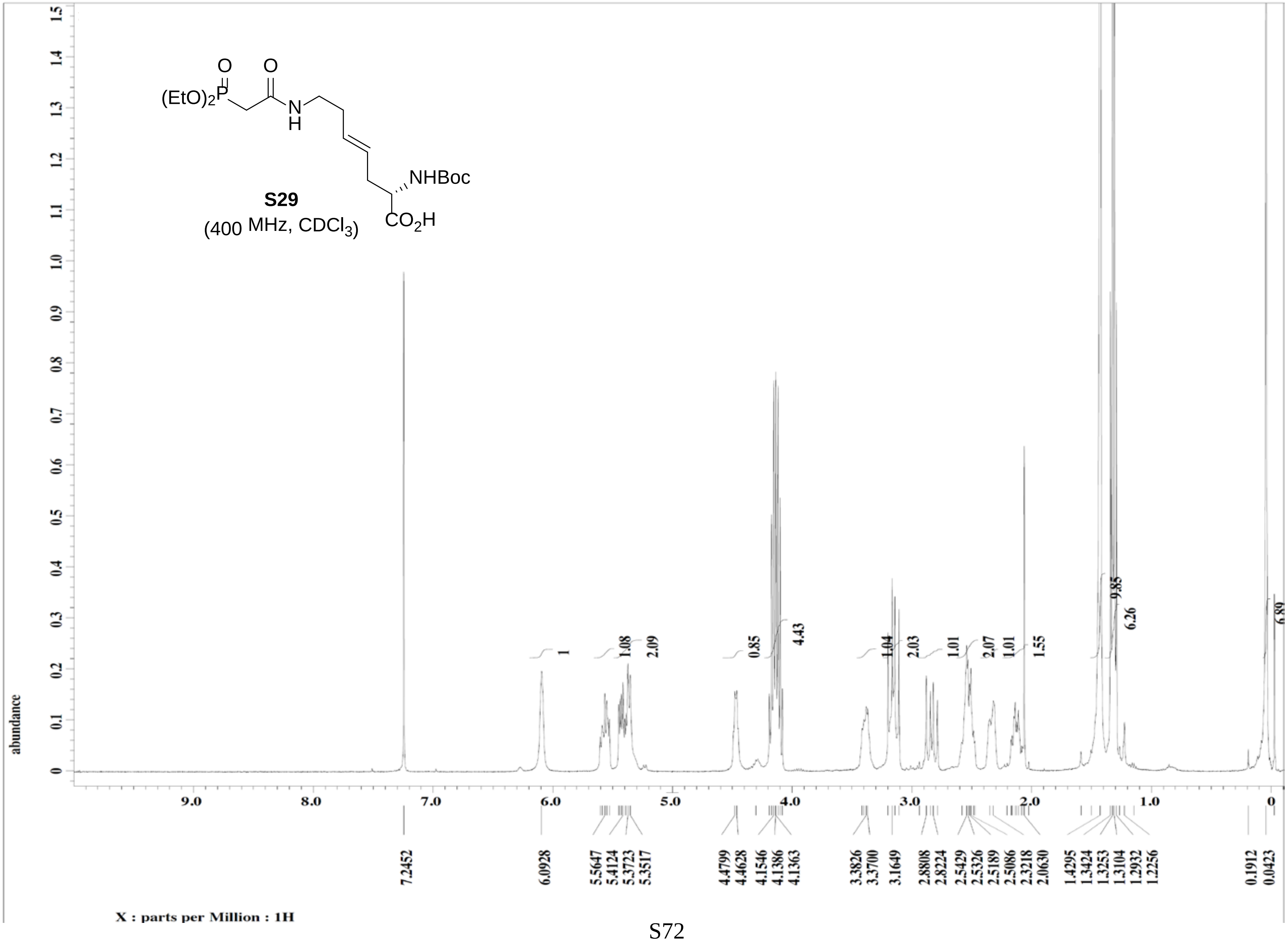
S29
(400 MHz, CDCl3)
(EtO)2P
NHBoc
CO2H
abundance
X : parts per Million : 1H
7.2452
6.0928
5.5647
5.4124
5.3723
5.3517
4.4799
4.4628
4.1546
4.1386
4.1363
3.3826
3.3700
3.1649
2.8808
2.8224
2.5429
2.5326
2.5189
2.5086
2.3218
2.0630
1.4295
1.3424
1.3253
1.3104
1.2932
1.2256
0.1912
0.0423

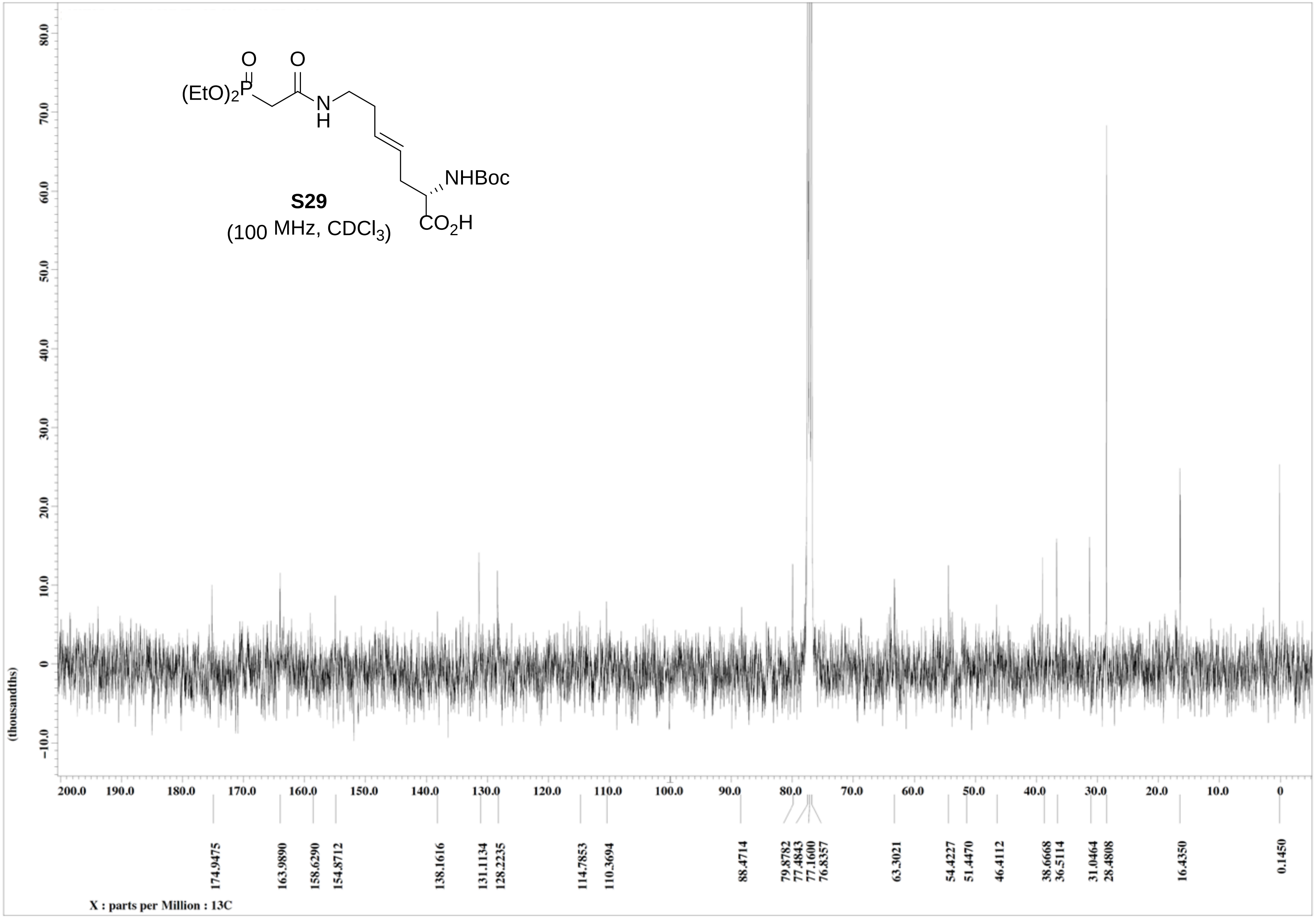

S29
(100 MHz, CDCl3)
(thousandths)
X : parts per Million : 13C
174.9475
163.9890
158.6290
154.8712
138.1616
131.1134
128.2235
114.7853
110.3694
88.4714
79.8782
77.4843
77.1600
76.8357
63.3021
54.4227
51.4470
46.4112
38.6668
36.5114
31.0464
28.4808
16.4350
0.1450

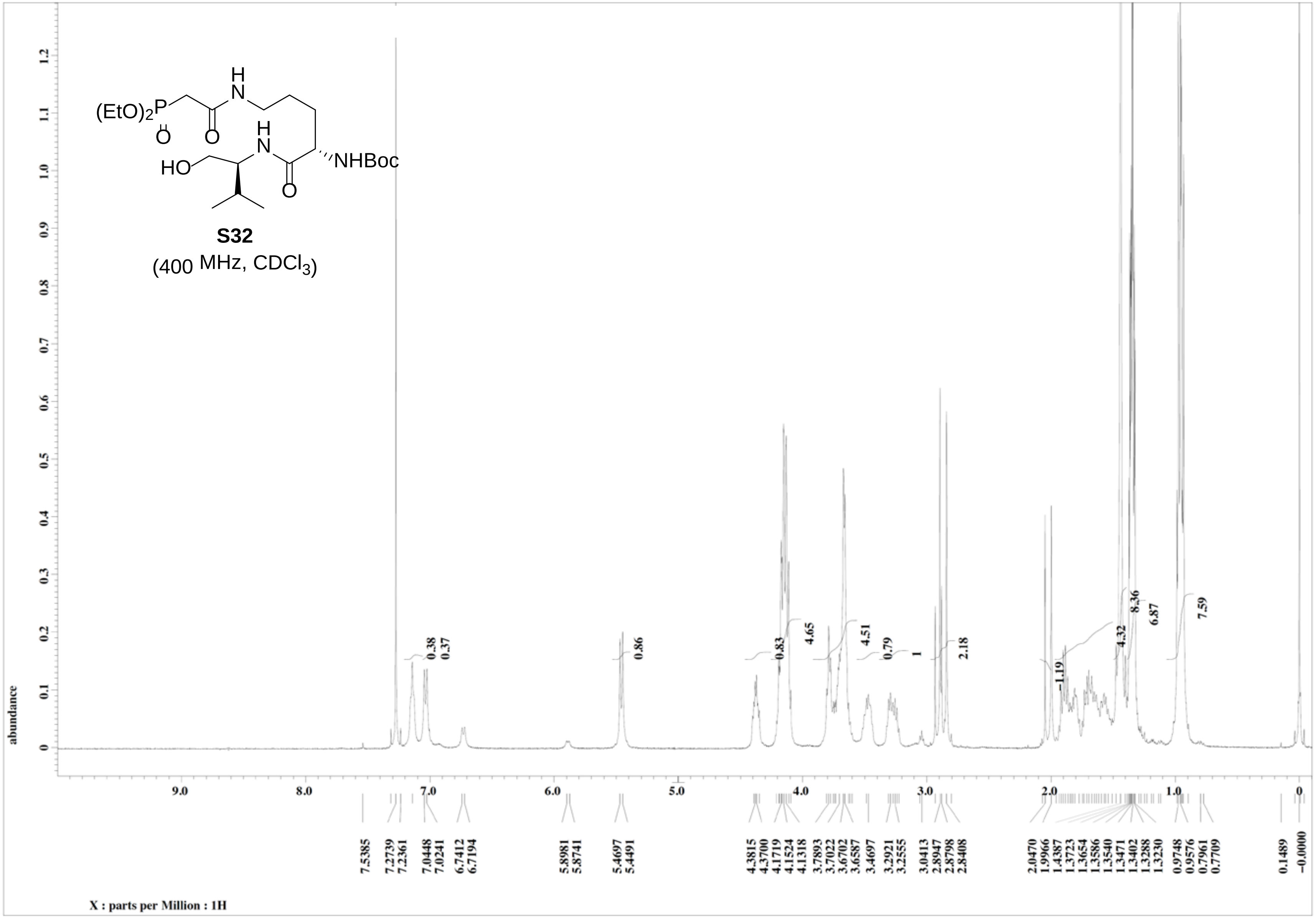

(EtO)2P
O
H
N
O
H
N
HO
O
NHBoc
S32
(400 MHz, CDCl3)
abundance
X : parts per Million : 1H

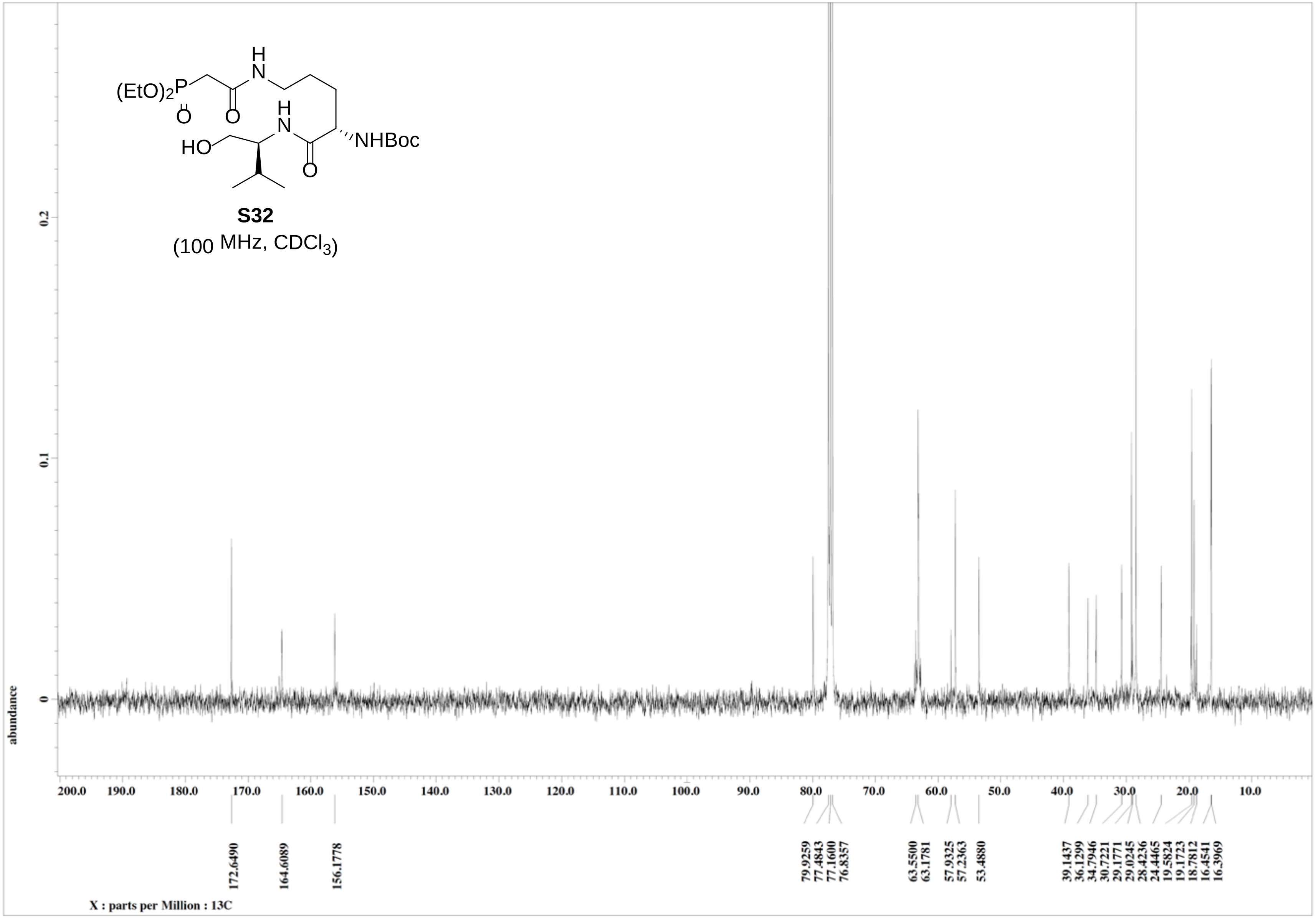

(EtO)2P
O
O
H
N
H
N
HO
O
NHBoc
S32
(100 MHz, CDCl3)
abundance
0.2
0.1
0
200.0
190.0
180.0
170.0
160.0
150.0
140.0
130.0
120.0
110.0
100.0
90.0
80.0
70.0
60.0
50.0
40.0
30.0
20.0
10.0
172.6490
164.6089
156.1778
79.9259
77.4843
77.1600
76.8357
63.5500
63.1781
57.9325
57.2363
53.4880
39.1437
36.1299
34.7946
30.7221
29.1771
29.0245
28.4236
24.4465
19.5824
19.1723
18.7812
16.4541
16.3969
X : parts per Million : 13C

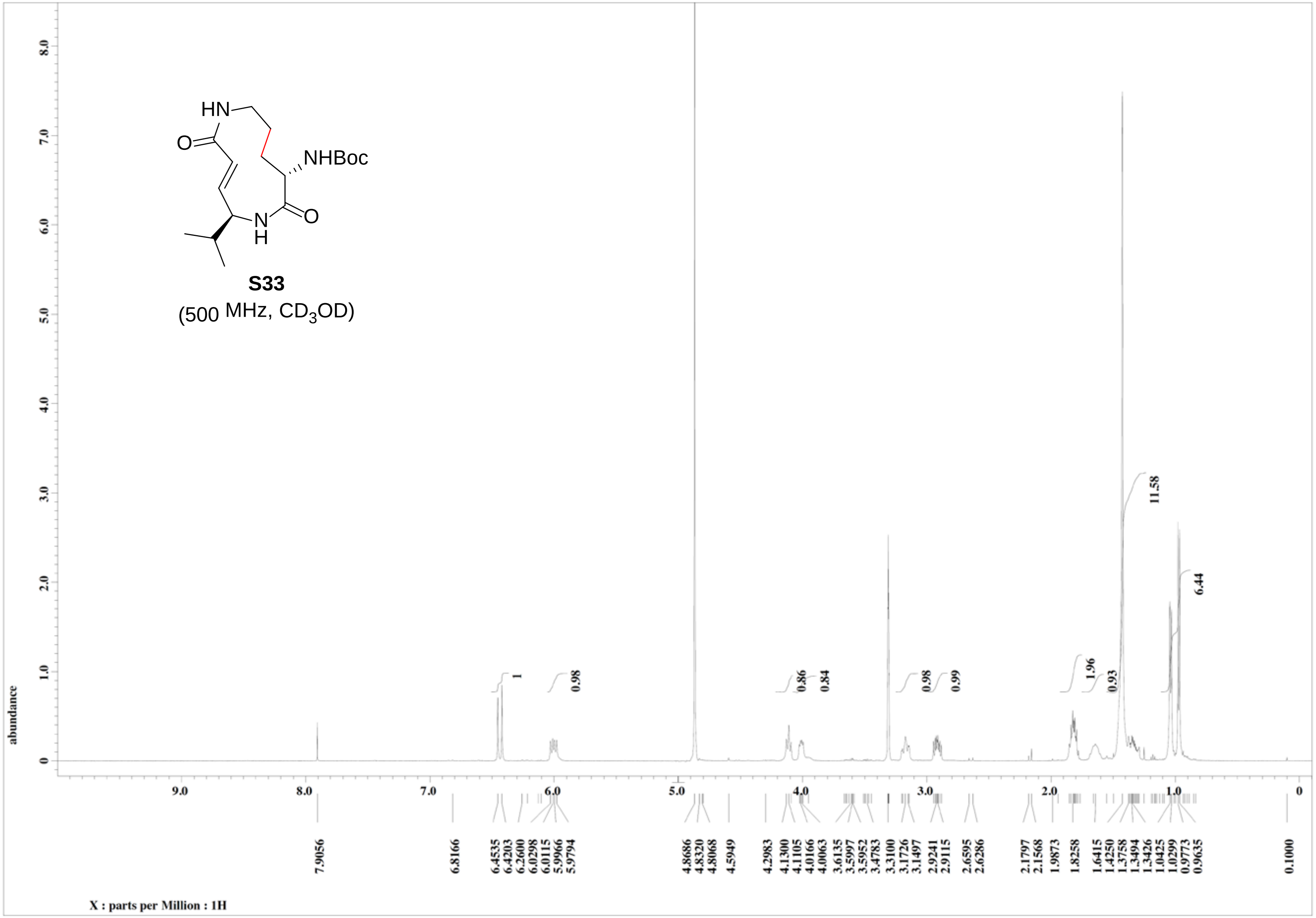
HN
O
NHBoc
N
H
O
S33
(500 MHz, CD3OD)
abundance
X : parts per Million : 1H
1
0.98
0.86
0.84
0.98
0.99
1.96
0.93
11.58
6.44
7.9056
6.8166
6.4535
6.4203
6.2600
6.0298
6.0115
5.9966
5.9794
4.8686
4.8320
4.8068
4.5949
4.2983
4.1300
4.1105
4.0166
4.0063
3.6135
3.5997
3.5952
3.4783
3.3100
3.1726
3.1497
2.9241
2.9115
2.6595
2.6286
2.1797
2.1568
1.9873
1.8258
1.6415
1.4250
1.3758
1.3494
1.3426
1.0425
1.0299
0.9773
0.9635
0.1000

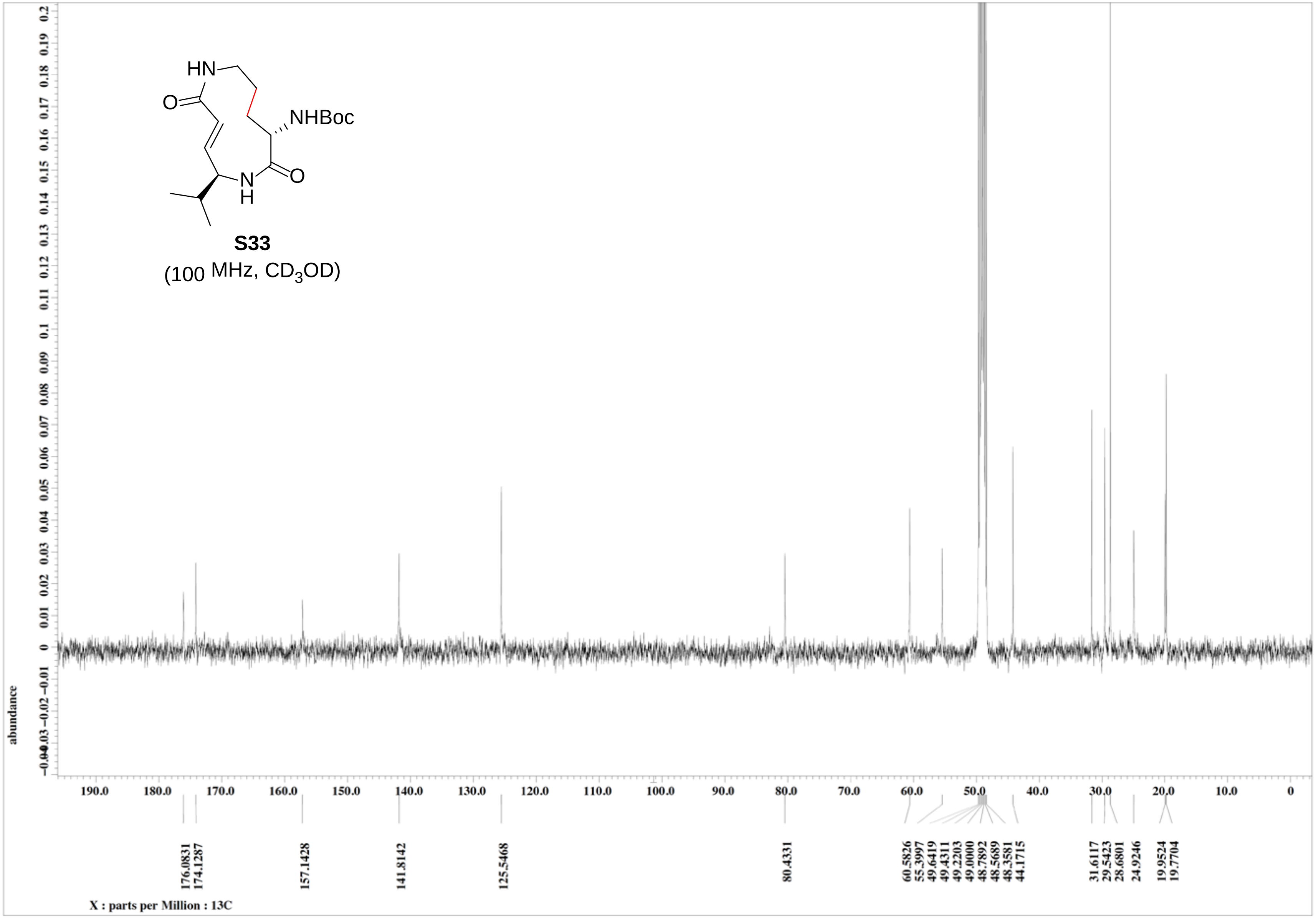

HN
O
NHBoc
N
H
O
S33
(100 MHz, CD3OD)
abundance
X : parts per Million : 13C
176.0831
174.1287
157.1428
141.8142
125.5468
80.4331
60.5826
55.3997
49.6419
49.4311
49.2203
49.0000
48.7892
48.5689
48.3581
44.1715
31.6117
29.5423
28.6801
24.9246
19.9524
19.7704

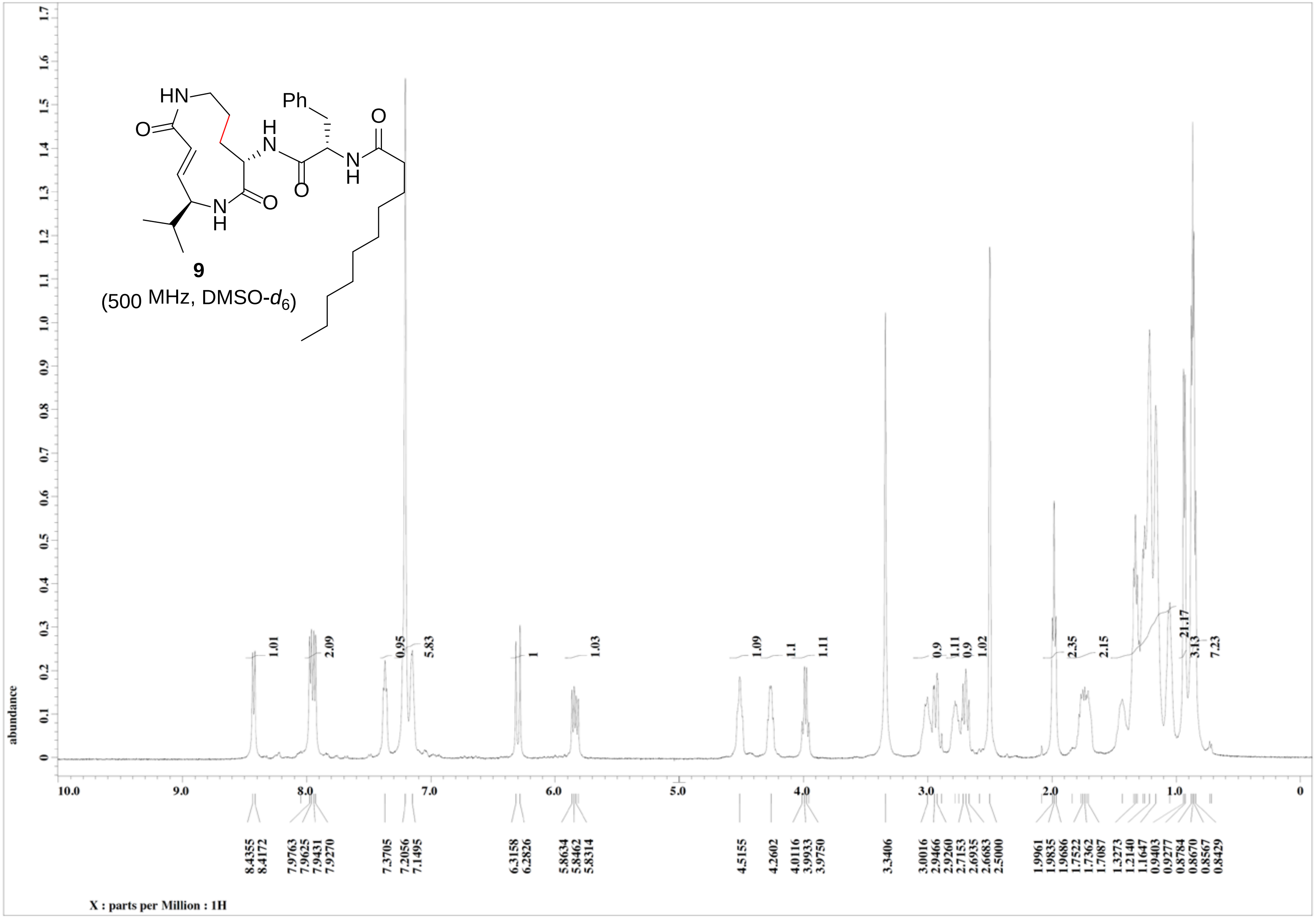

9
(500 MHz, DMSO-d_6)
abundance
X : parts per Million : 1H

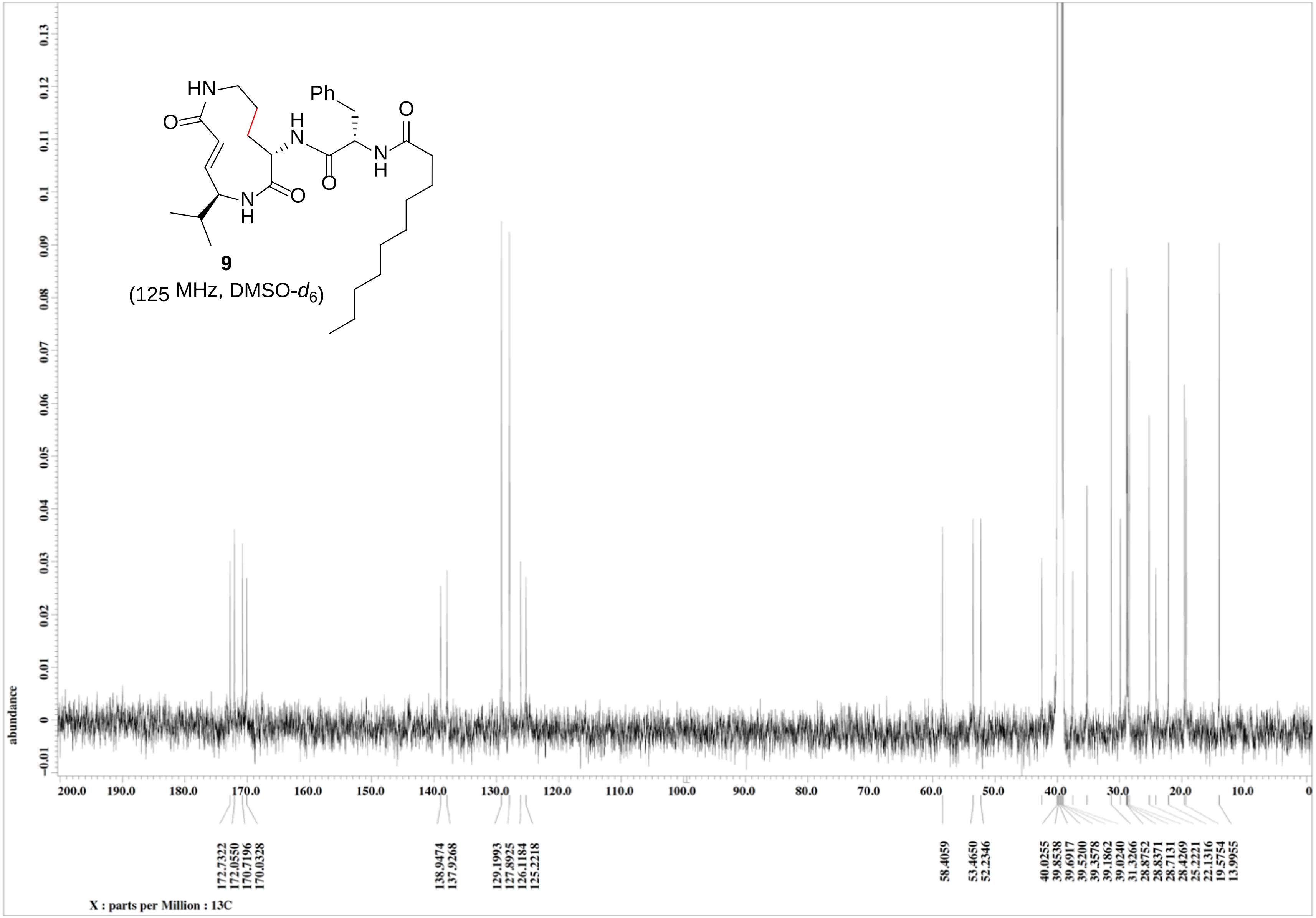
HN
O
Ph
O
H
N
O
N
H
N
H
O
9
(125 MHz, DMSO-d_6)
abundance
0.13
0.12
0.11
0.1
0.09
0.08
0.07
0.06
0.05
0.04
0.03
0.02
0.01
0
−0.01
200.0 190.0 180.0 170.0 160.0 150.0 140.0 130.0 120.0 110.0 100.0 90.0 80.0 70.0 60.0 50.0 40.0 30.0 20.0 10.0 0
172.7322
172.0550
170.7196
170.0328
138.9474
137.9268
129.1993
127.8925
126.1184
125.2218
58.4059
53.4650
52.2346
40.0255
39.8538
39.6917
39.5200
39.3578
39.1862
39.0240
31.3266
28.8752
28.8371
28.7131
28.4269
25.2221
22.1316
19.5754
13.9955
X : parts per Million : 13C

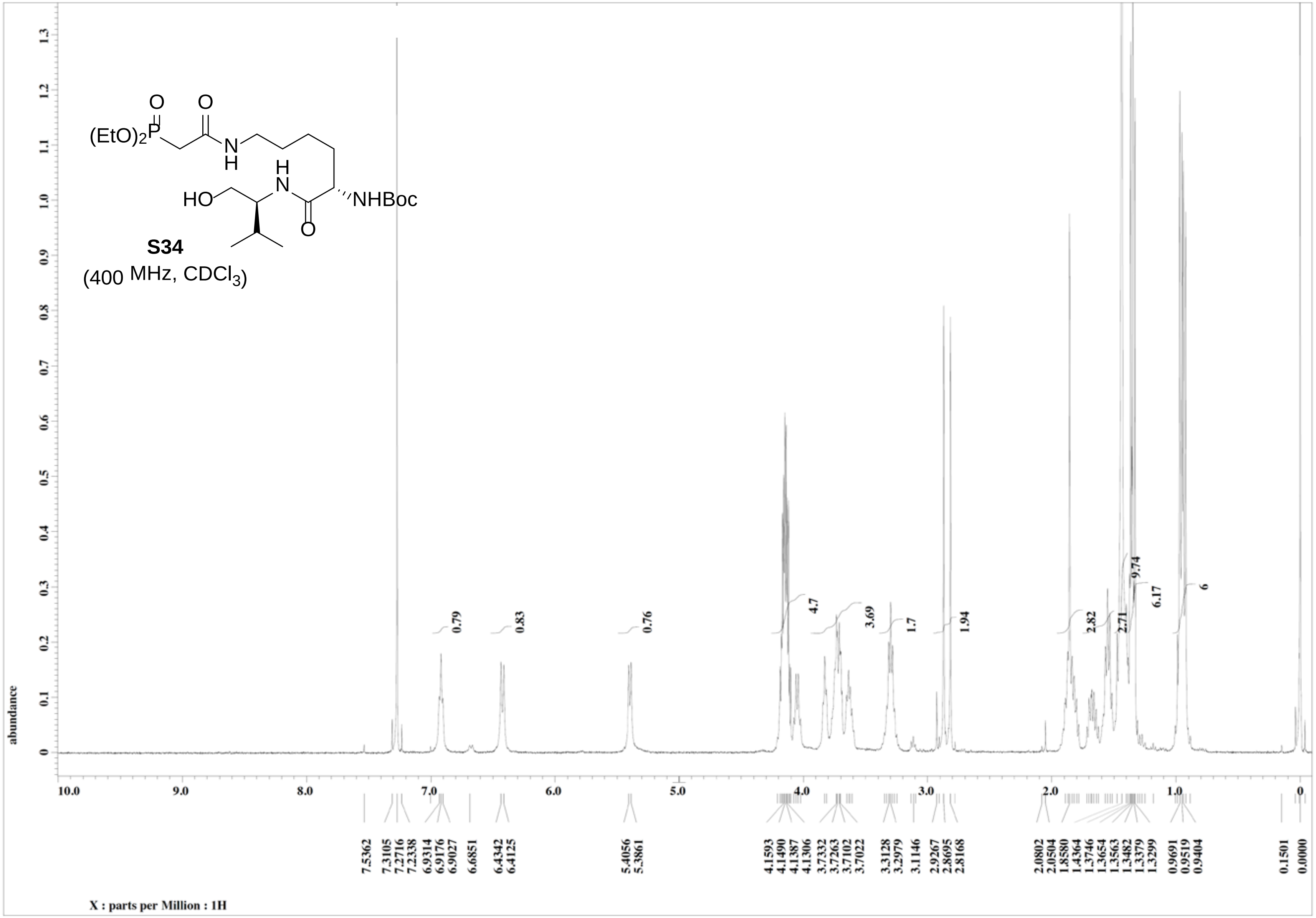
(EtO)2P
O
O
N
H
H
N
HO
NHBoc
O
S34
(400 MHz, CDCl3)
abundance
X : parts per Million : 1H
0.79
0.83
0.76
4.7
3.69
1.7
1.94
2.82
2.71
9.74
6.17
6
7.5362
7.3105
7.2716
7.2338
6.9314
6.9176
6.9027
6.6851
6.4342
6.4125
5.4056
5.3861
4.1593
4.1490
4.1387
4.1306
3.7332
3.7263
3.7102
3.7022
3.3128
3.2979
3.1146
2.9267
2.8695
2.8168
2.0802
2.0504
1.8580
1.4364
1.3746
1.3654
1.3563
1.3482
1.3379
1.3299
0.9691
0.9519
0.9404
0.1501
0.0000

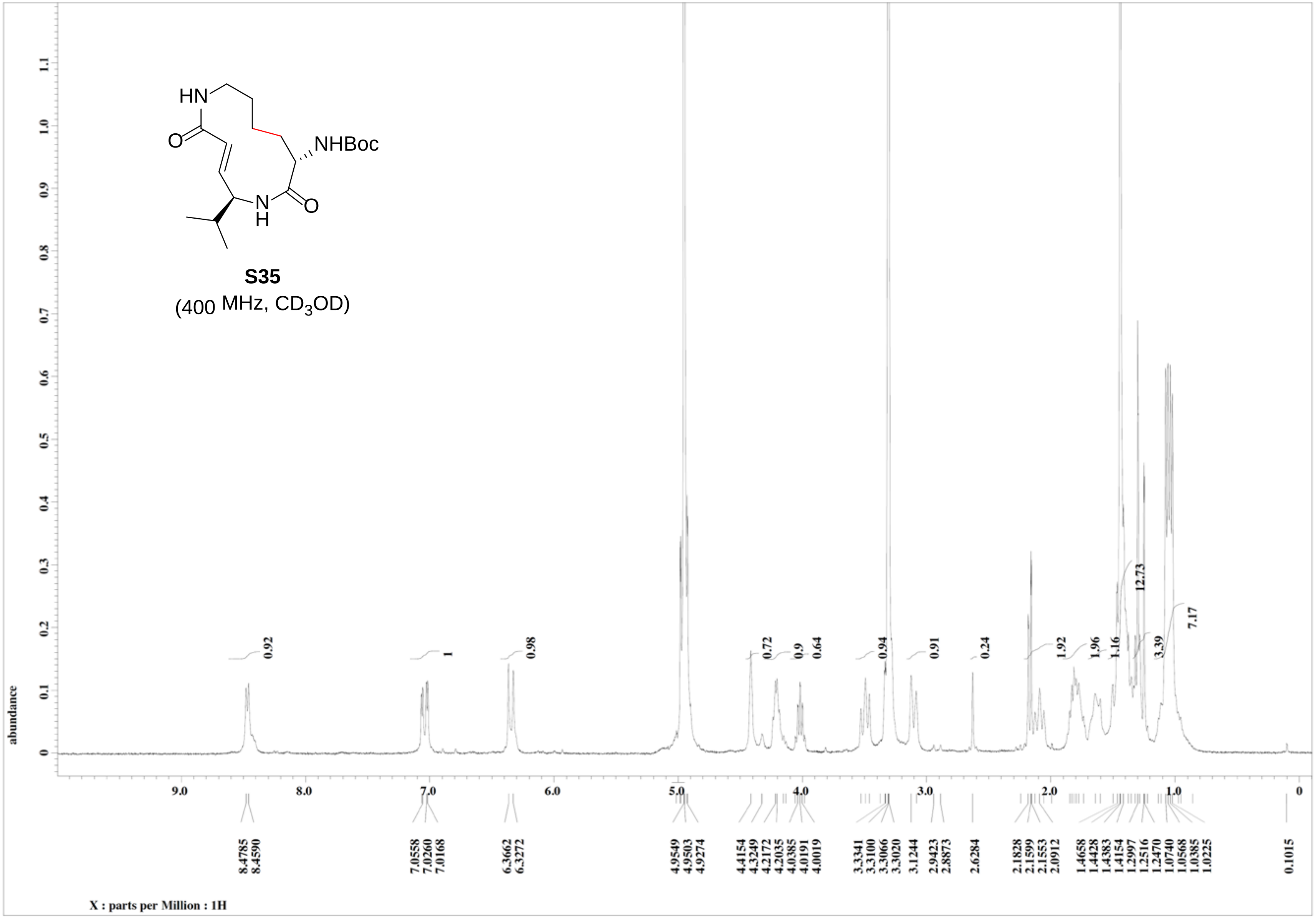
HN
NHBoc
O
N
H
O
S35
(400 MHz, CD3OD)
abundance
X : parts per Million : 1H

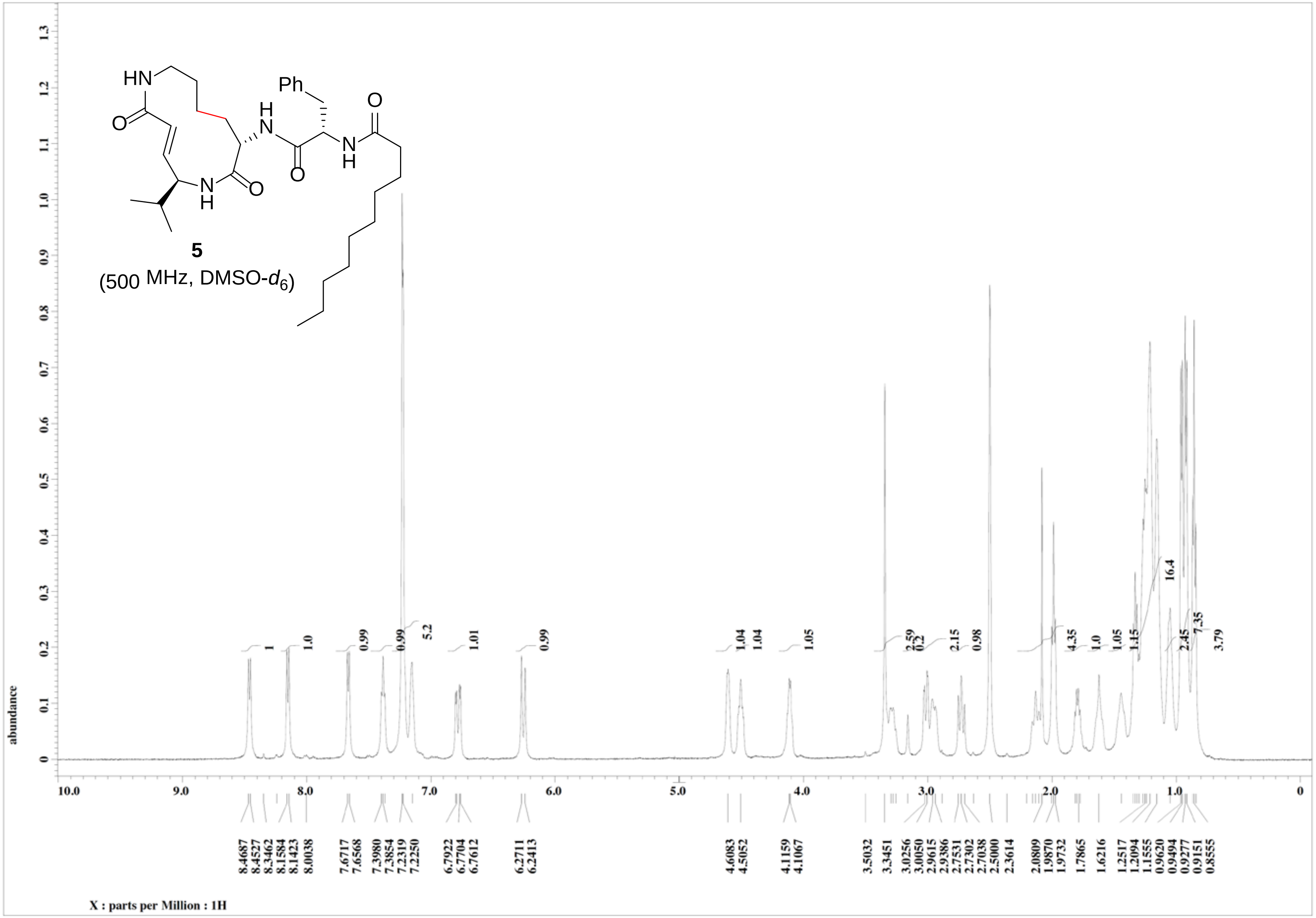

5
(500 MHz, DMSO-d_6)
abundance
X : parts per Million : 1H

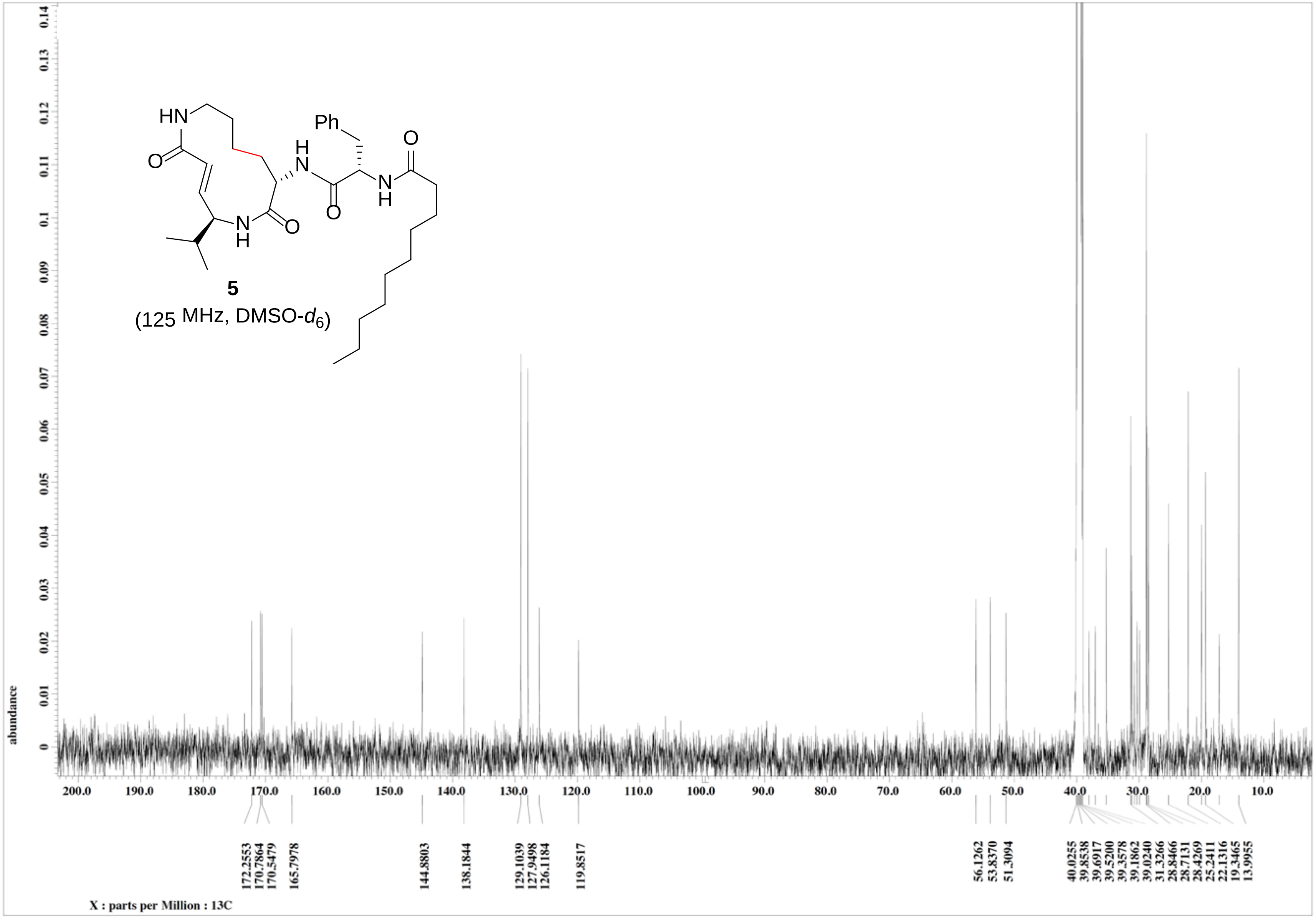

5
(125 MHz, DMSO-d_6)
abundance
X : parts per Million : 13C
172.2553
170.7864
170.5479
165.7978
144.8803
138.1844
129.1039
127.9498
126.1184
119.8517
56.1262
53.8370
51.3094
40.0255
39.8538
39.6917
39.5200
39.3578
39.1862
39.0240
31.3266
28.8466
28.7131
28.4269
25.2411
22.1316
19.3465
13.9955

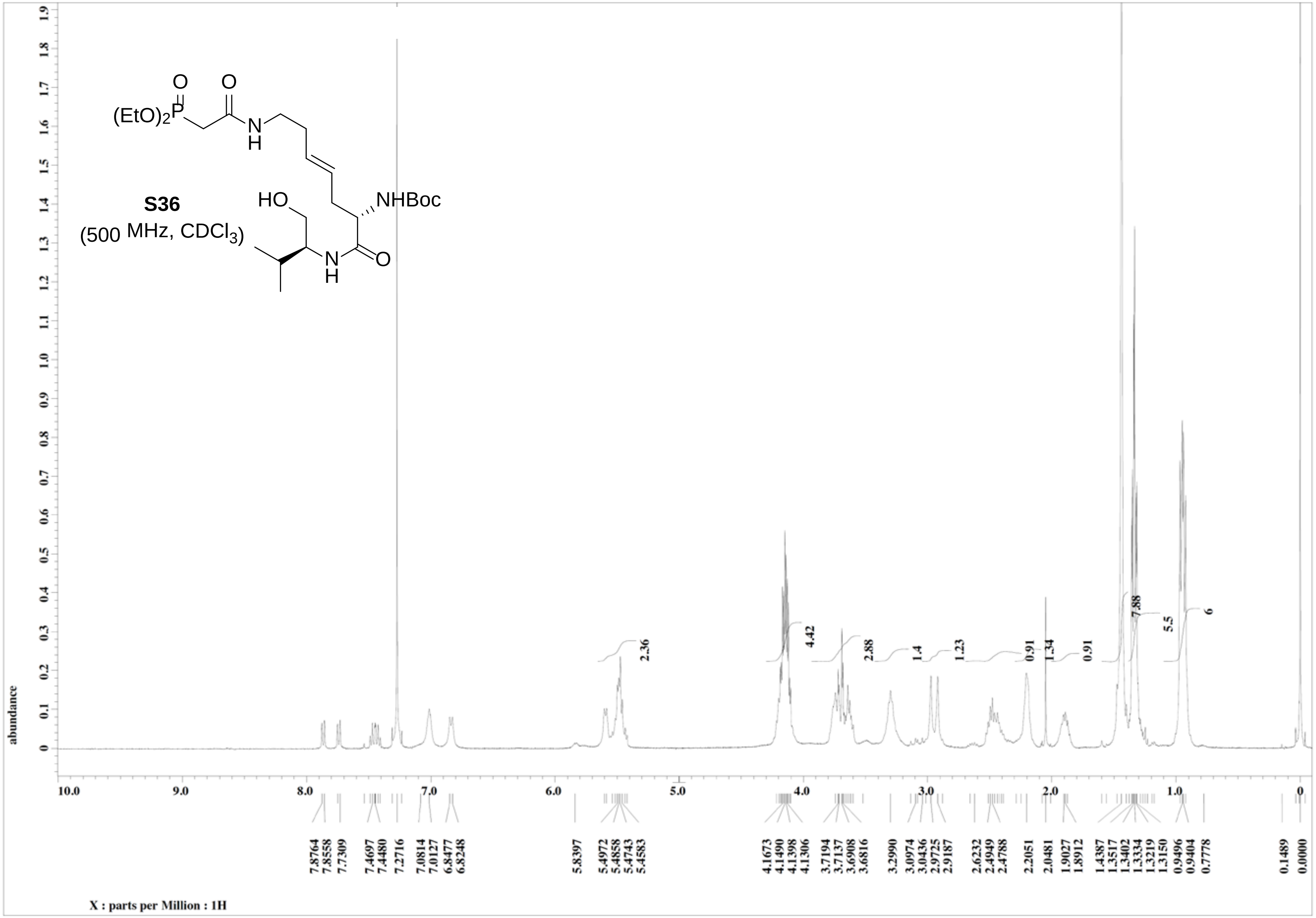

(EtO)2P
O
O
N
H
HO
NHBoc
N
H
O
S36
(500 MHz, CDCl3)
abundance
X : parts per Million : 1H

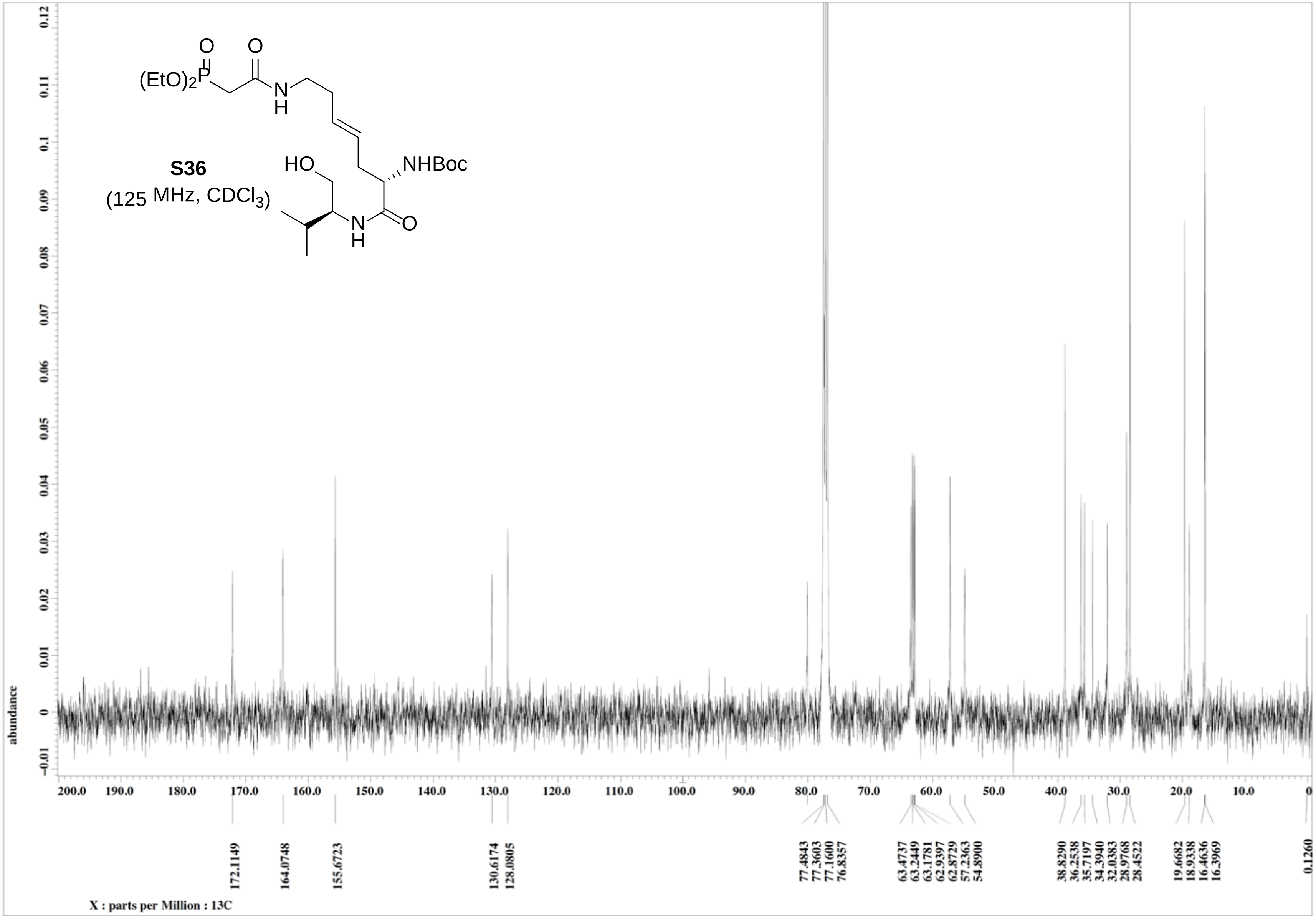

(EtO)2P
O
O
N
H
HO
NHBoc
N
H
O
S36
(125 MHz, CDCl3)
abundance
X : parts per Million : 13C
172.1149
164.0748
155.6723
130.6174
128.0805
77.4843
77.3603
77.1600
76.8357
63.4737
63.2449
63.1781
62.9397
62.8729
57.2363
54.8900
38.8290
36.2538
35.7197
34.3940
32.0383
28.9768
28.4522
19.6682
18.9338
16.4636
16.3969
0.1260

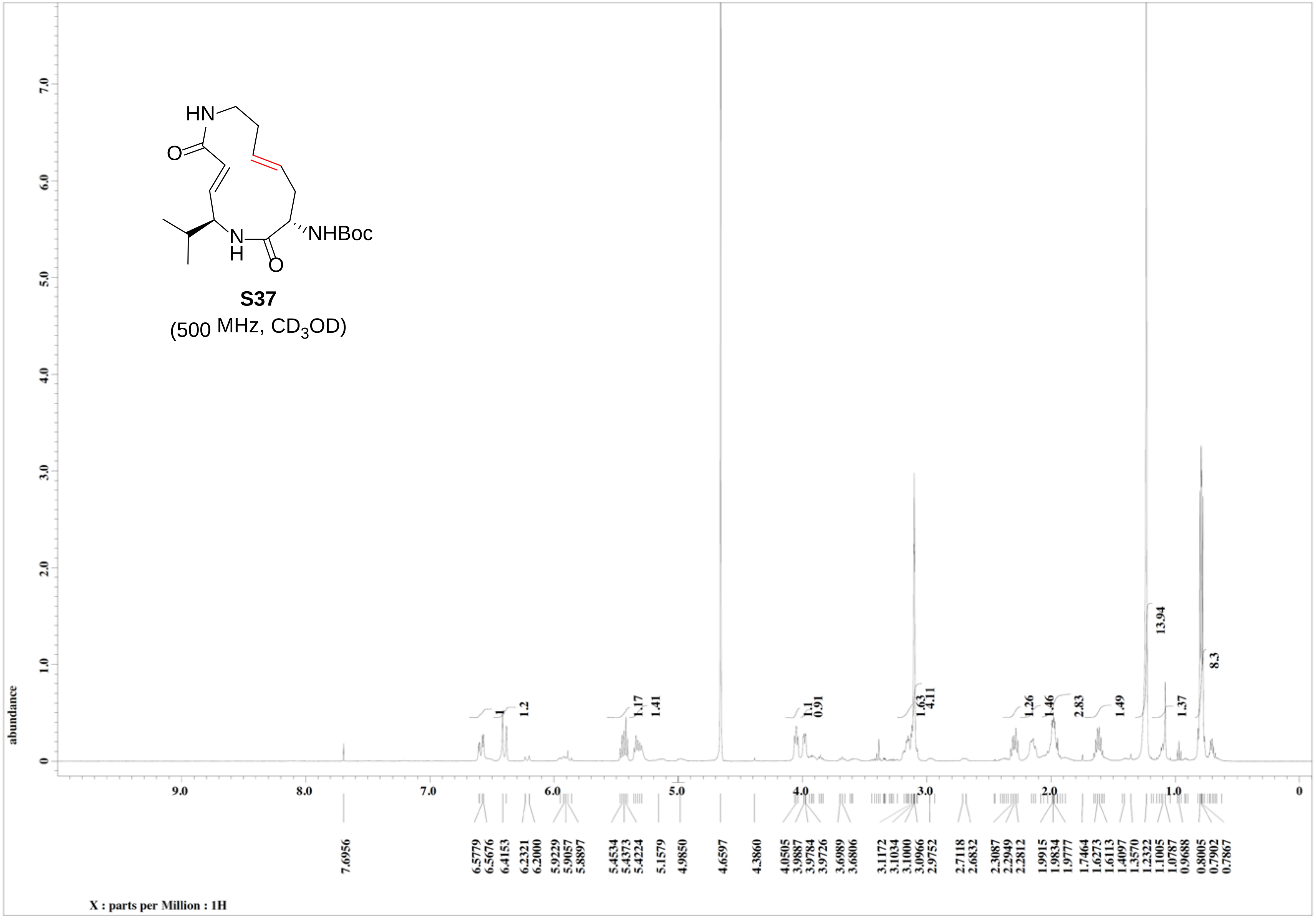
HN
O
NH
H
O
NHBoc
S37
(500 MHz, CD3OD)
abundance
X : parts per Million : 1H
1
1.2
1.17
1.41
1.1
0.91
1.63
4.11
1.26
1.46
2.83
1.49
13.94
1.37
8.3
7.6956
6.5779
6.5676
6.4153
6.2321
6.2000
5.9229
5.9057
5.8897
5.4534
5.4373
5.4224
5.1579
4.9850
4.6597
4.3860
4.0505
3.9887
3.9784
3.9726
3.6989
3.6806
3.1172
3.1034
3.1000
3.0966
2.9752
2.7118
2.6832
2.3087
2.2949
2.2812
1.9915
1.9834
1.9777
1.7464
1.6273
1.6113
1.4097
1.3570
1.2322
1.1005
1.0787
0.9688
0.8005
0.7902
0.7867

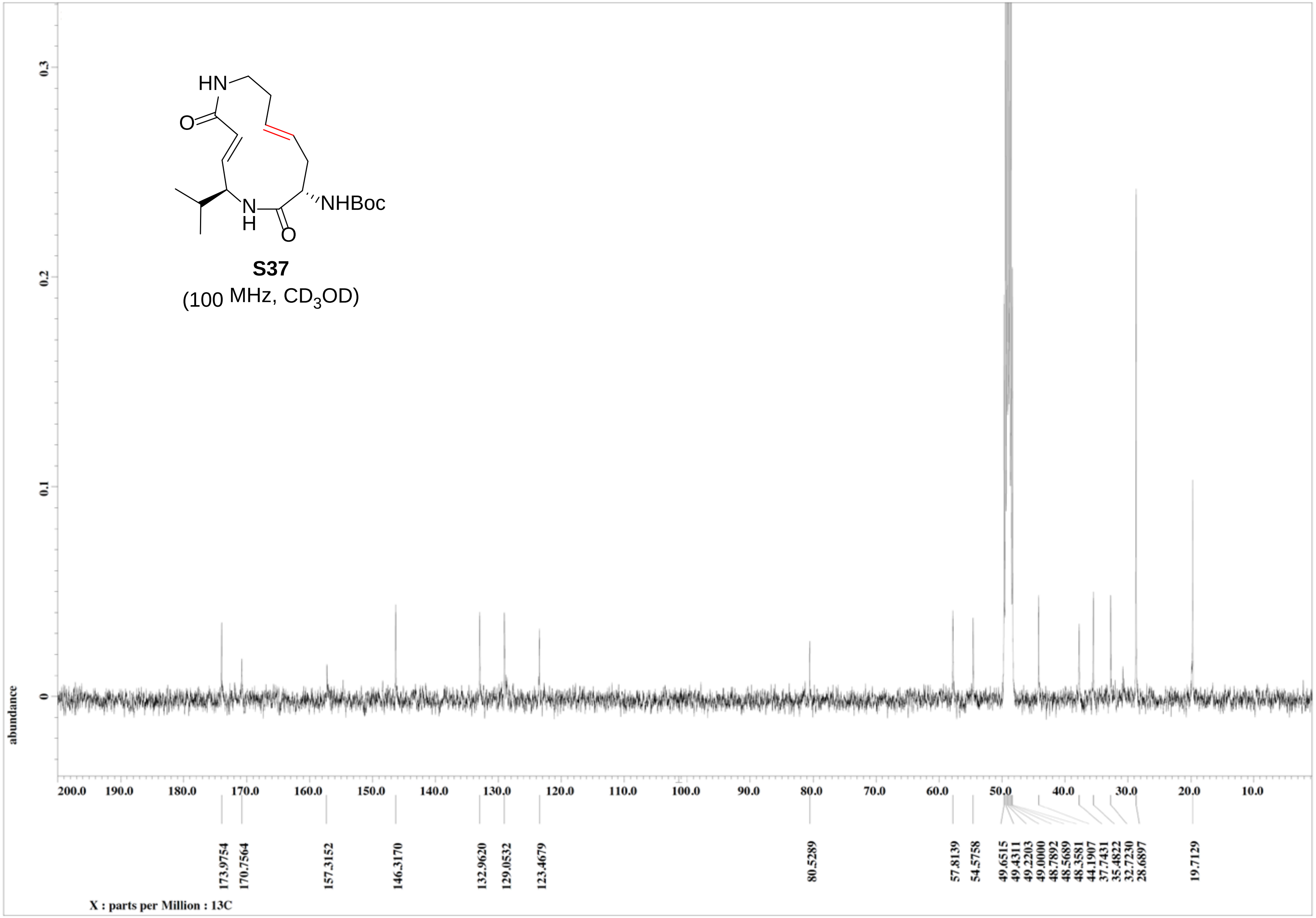
HN
O
N
H
O
NHBoc
S37
(100 MHz, CD3OD)
0.3
0.2
0.1
0
abundance
200.0
190.0
180.0
170.0
160.0
150.0
140.0
130.0
120.0
110.0
100.0
90.0
80.0
70.0
60.0
50.0
40.0
30.0
20.0
10.0
173.9754
170.7564
157.3152
146.3170
132.9620
129.0532
123.4679
80.5289
57.8139
54.5758
49.6515
49.4311
49.2203
49.0000
48.7892
48.5689
48.3581
44.1907
37.7431
35.4822
32.7230
28.6897
19.7129
X : parts per Million : 13C

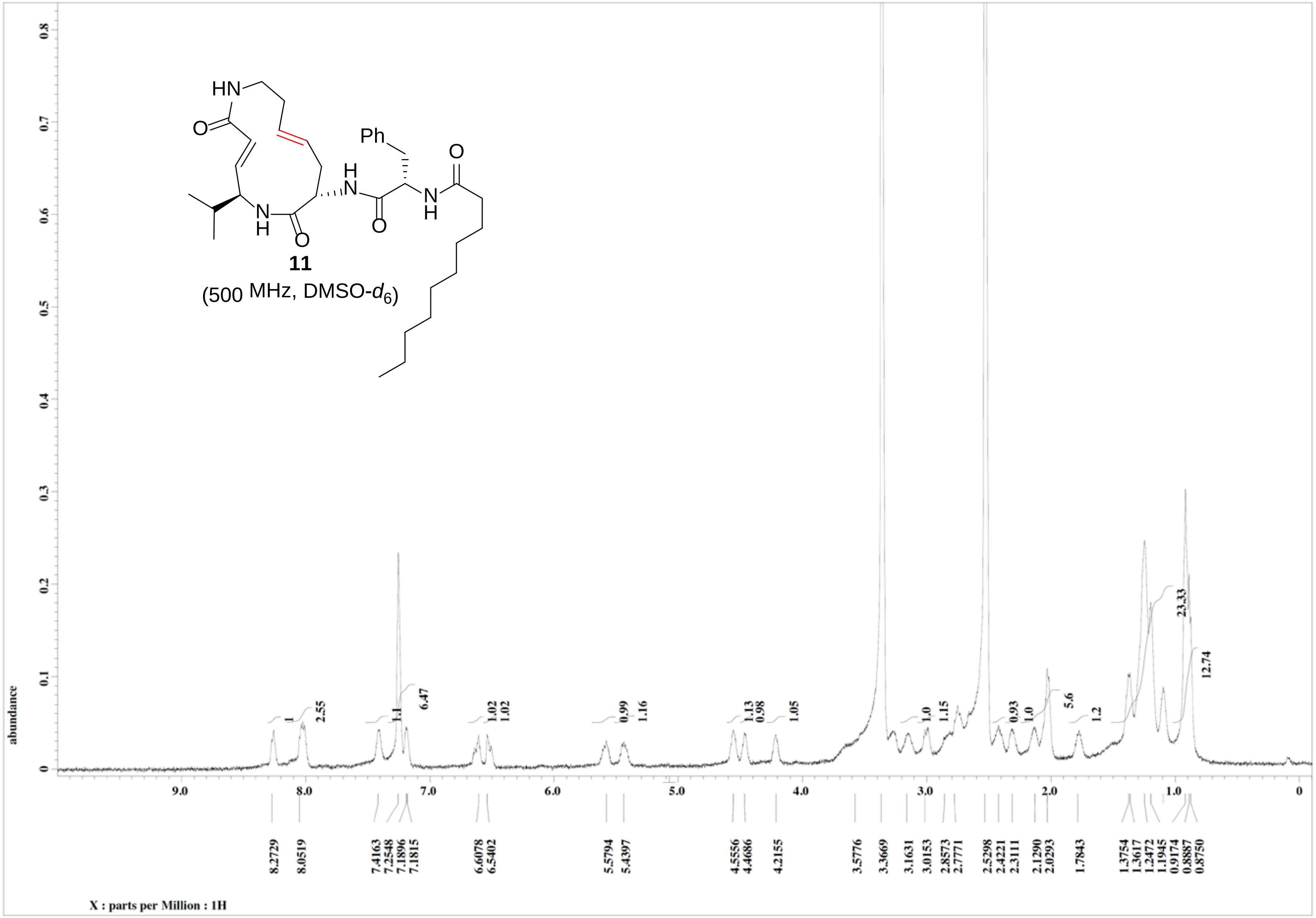
HN
O
Ph
O
H
N
N
H
N
H
O
O
11
(500 MHz, DMSO-d_6)
abundance
X : parts per Million : 1H
9.0
8.0
7.0
6.0
5.0
4.0
3.0
2.0
1.0
0
0
0.1
0.2
0.3
0.4
0.5
0.6
0.7
0.8
1
2.55
1.1
6.47
1.02
1.02
0.99
1.16
1.13
0.98
1.05
1.0
1.15
0.93
1.0
5.6
1.2
23.33
12.74
8.2729
8.0519
7.4163
7.2548
7.1896
7.1815
6.6078
6.5402
5.5794
5.4397
4.5556
4.4686
4.2155
3.5776
3.3669
3.1631
3.0153
2.8573
2.7771
2.5298
2.4221
2.3111
2.1290
2.0293
1.7843
1.3754
1.3617
1.2472
1.1945
0.9174
0.8887
0.8750

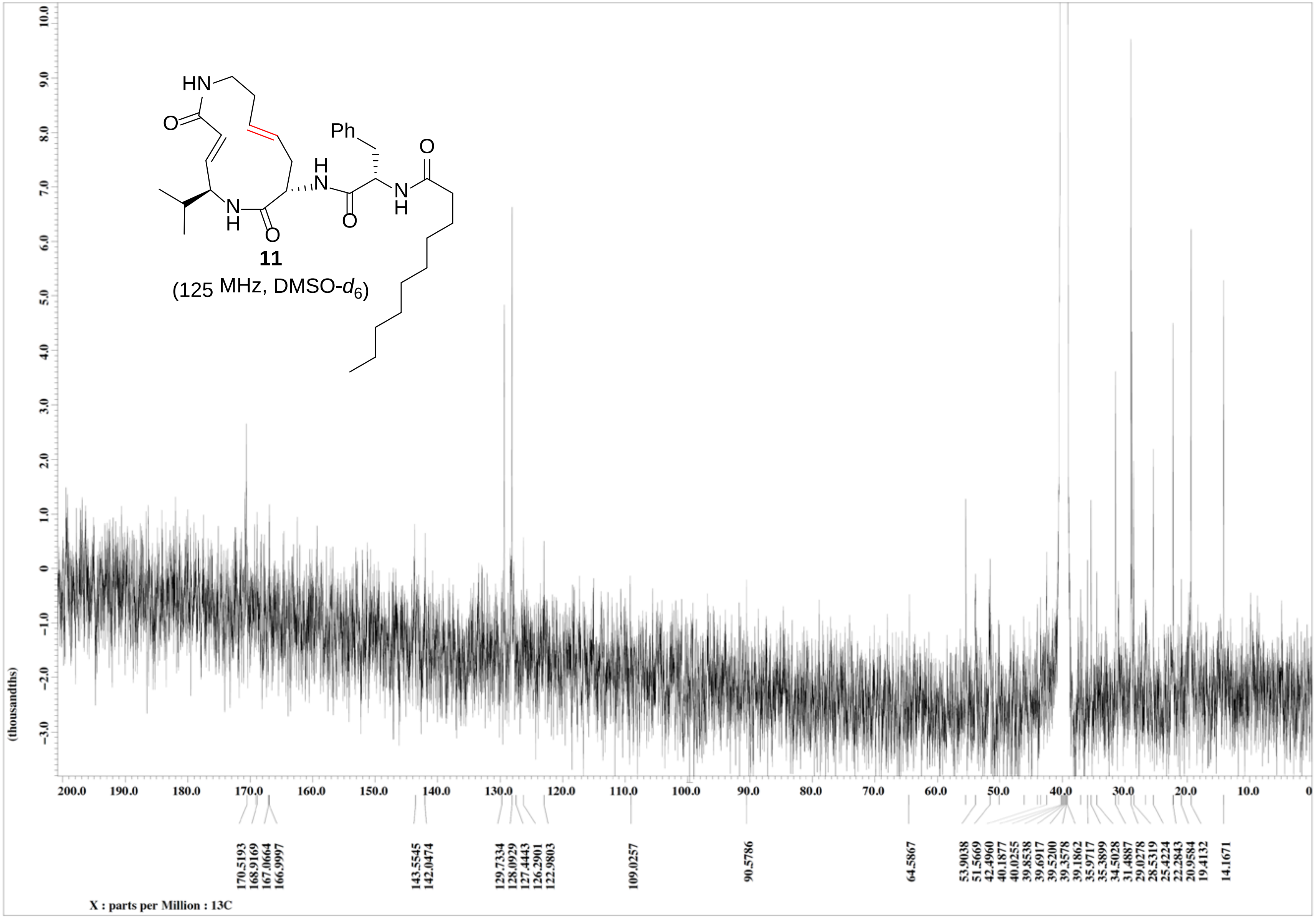

HN
O
Ph
O
H
N
N
H
N
H
O
O
11
(125 MHz, DMSO-d_6)
(thousandths)
170.5193
168.9169
167.0664
166.9997
143.5545
142.0474
129.7334
128.0929
127.4443
126.2901
122.9803
109.0257
90.5786
64.5867
53.9038
51.5669
42.4960
40.1877
40.0255
39.8538
39.6917
39.5200
39.3578
39.1862
35.9717
35.3899
34.5028
31.4887
29.0278
28.5319
25.4224
22.2843
20.9584
19.4132
14.1671
X : parts per Million : 13C

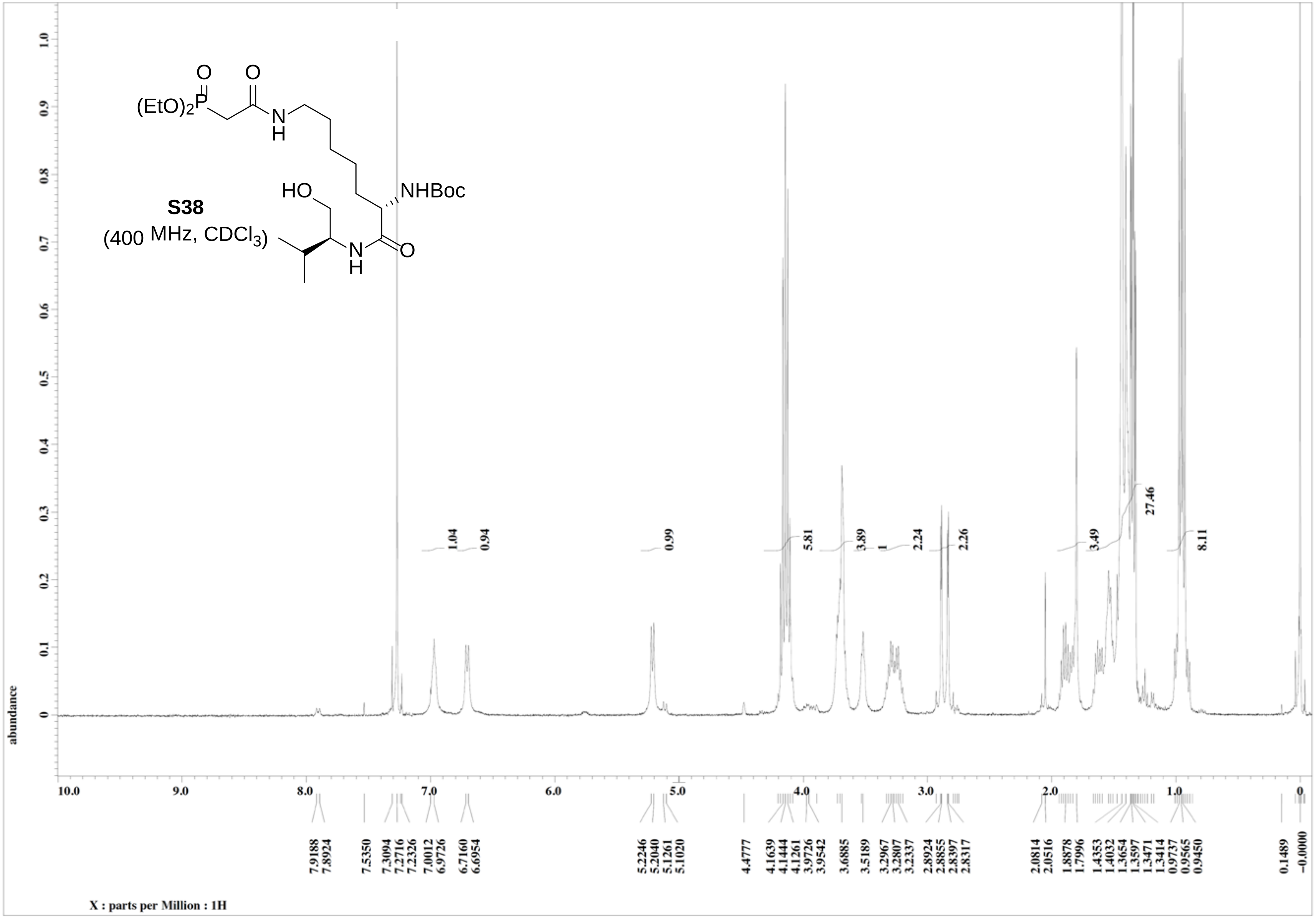
(EtO)2P
O
O
N
H
NHBoc
HO
N
H
O
S38
(400 MHz, CDCl3)
abundance
X : parts per Million : 1H
1.04
0.94
0.99
5.81
3.89
1
2.24
2.26
3.49
27.46
8.11
7.9188
7.8924
7.5350
7.3094
7.2716
7.2326
7.0012
6.9726
6.7160
6.6954
5.2246
5.2040
5.1261
5.1020
4.4777
4.1639
4.1444
4.1261
3.9726
3.9542
3.6885
3.5189
3.2967
3.2807
3.2337
2.8924
2.8855
2.8397
2.8317
2.0814
2.0516
1.8878
1.7996
1.4353
1.4032
1.3654
1.3597
1.3471
1.3414
0.9737
0.9565
0.9450
0.1489
-0.0000

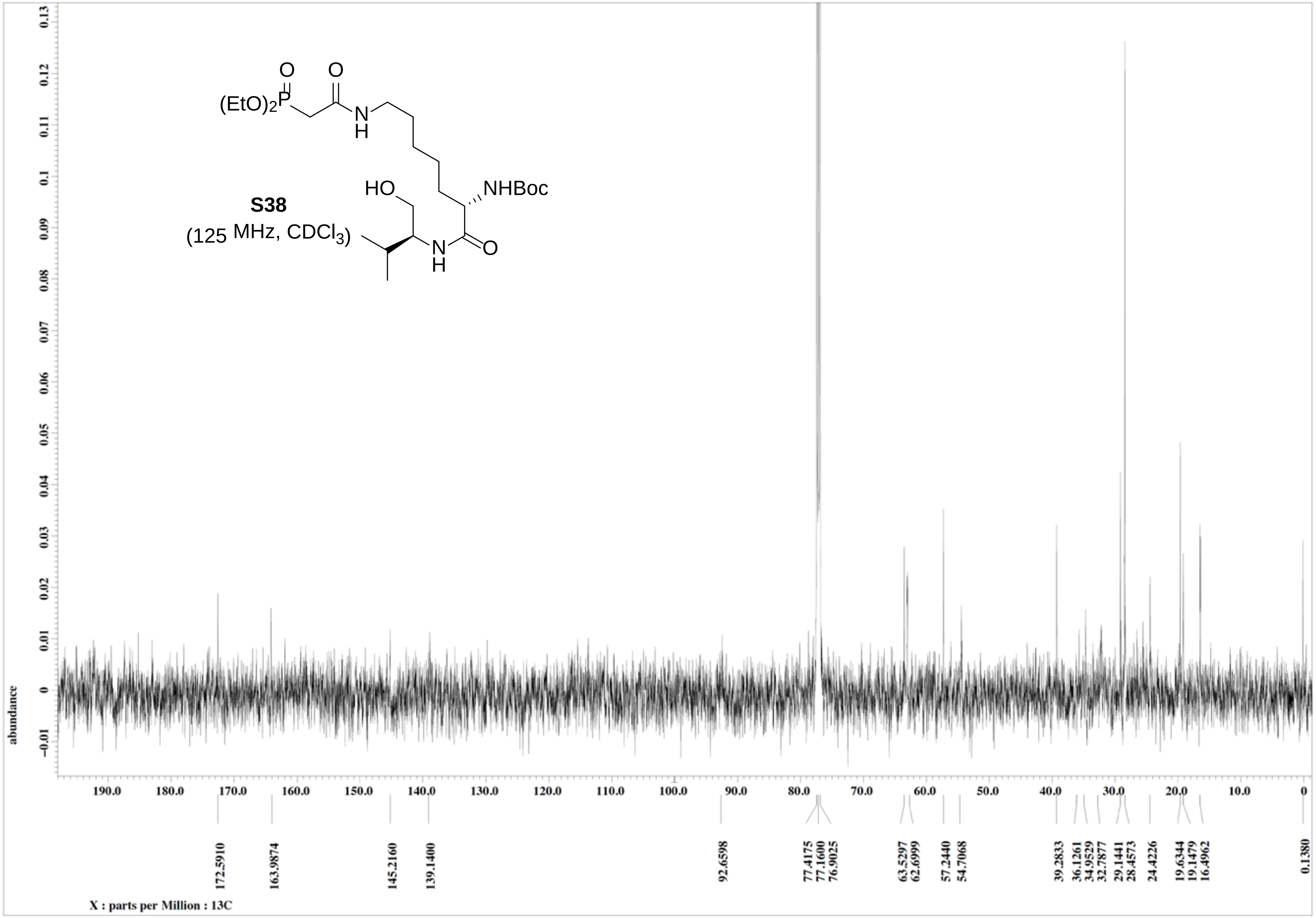
(EtO)2P
NHBoc
HO
S38
(125 MHz, CDCl3)
abundance
X : parts per Million : 13C
172.5910
163.9874
145.2160
139.1400
92.6598
77.4175
77.1600
76.9025
63.5297
62.6999
57.2440
54.7068
39.2833
36.1261
34.9529
32.7877
29.1441
28.4573
24.4226
19.6344
19.1479
16.4962
0.1380

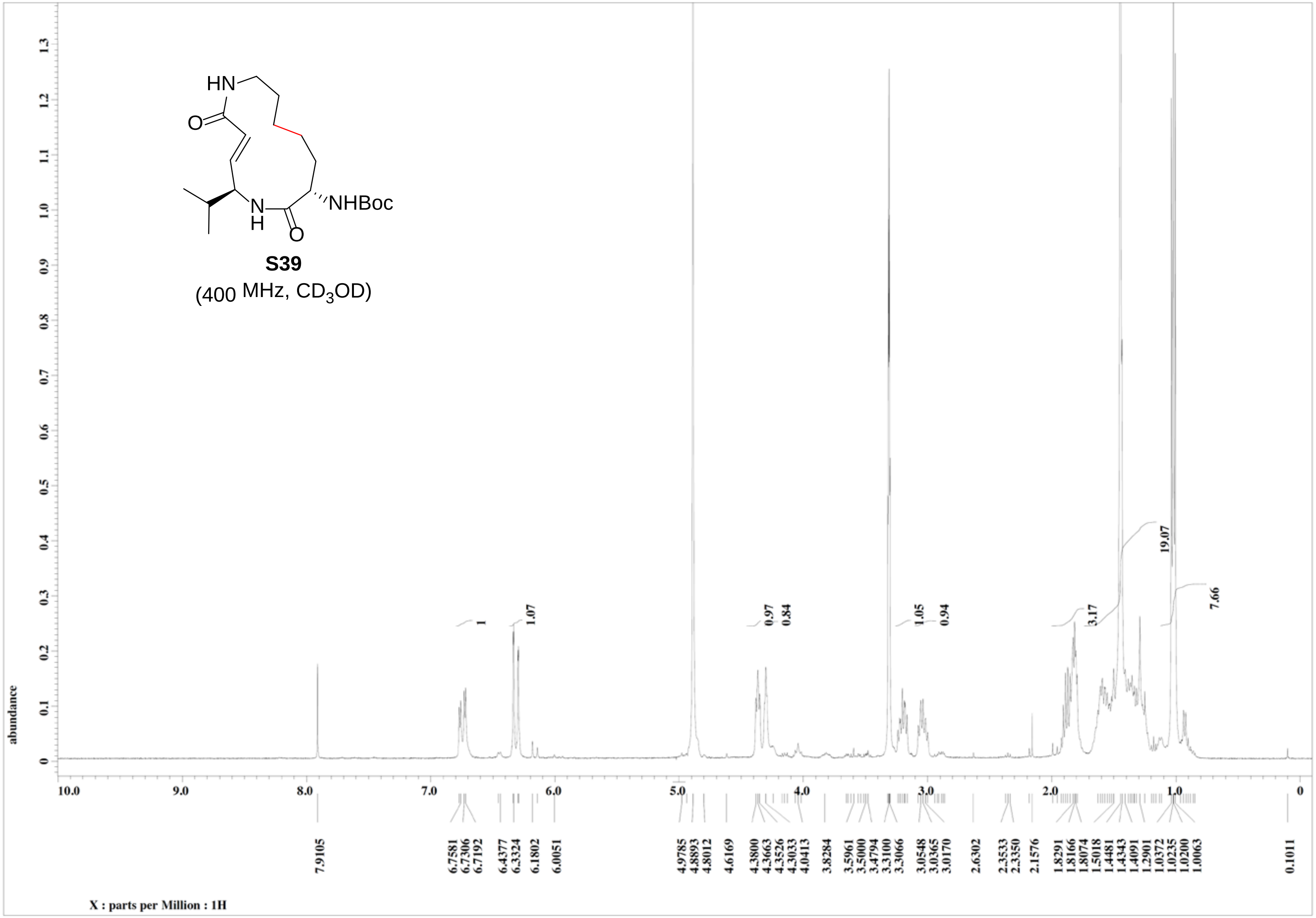
HN
O
NHBoc
N
H
O
S39
(400 MHz, CD3OD)
abundance
X : parts per Million : 1H
7.9105
6.7581
6.7306
6.7192
6.4377
6.3324
6.1802
6.0051
4.9785
4.8893
4.8012
4.6169
4.3800
4.3663
4.3526
4.3033
4.0413
3.8284
3.5961
3.5000
3.4794
3.3100
3.3066
3.0548
3.0365
3.0170
2.6302
2.3533
2.3350
2.1576
1.8291
1.8166
1.8074
1.5018
1.4481
1.4343
1.4091
1.2901
1.0372
1.0235
1.0200
1.0063
0.1011
1
1.07
0.97
0.84
1.05
0.94
3.17
19.07
7.66

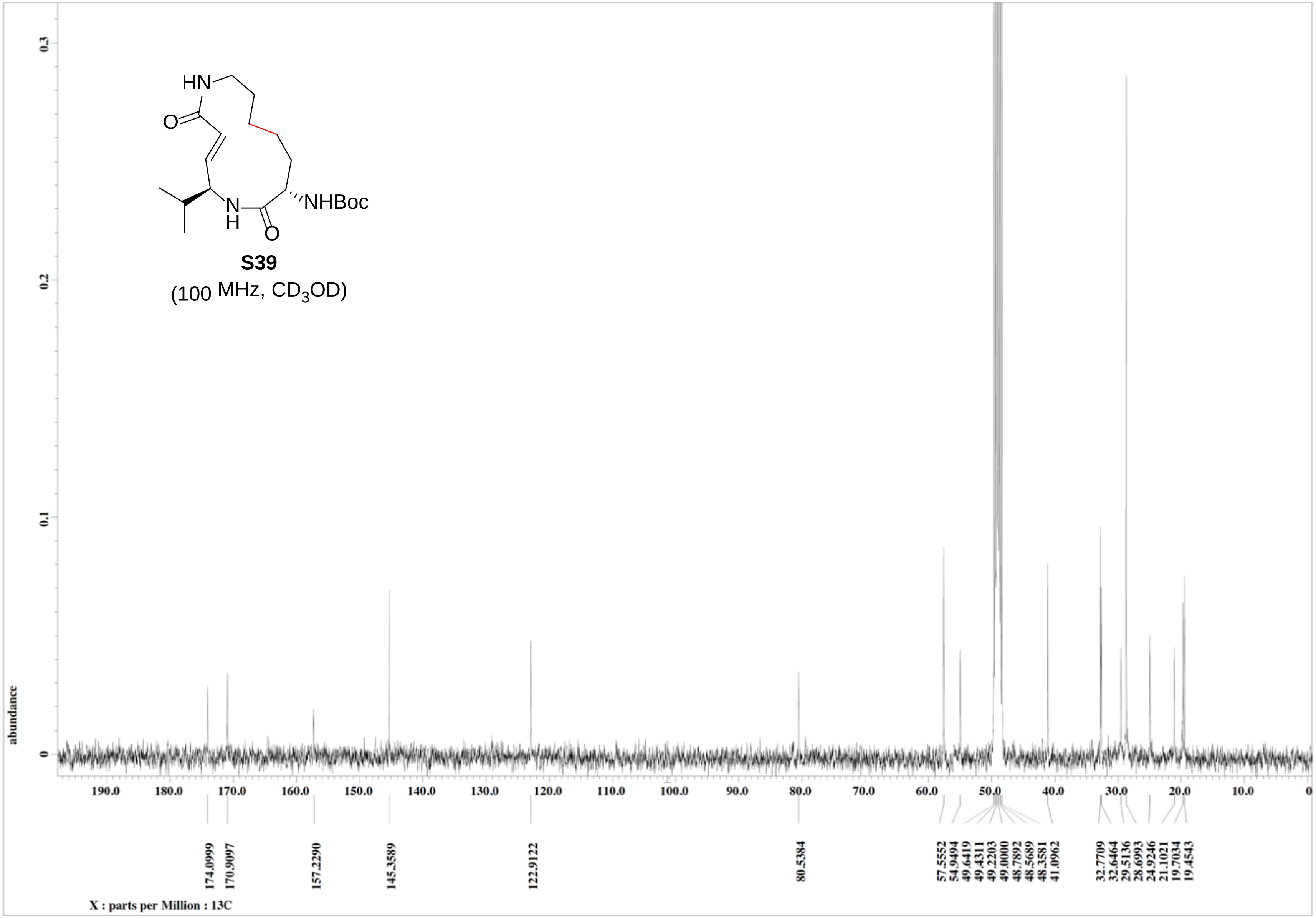
HN
O
NHBoc
N
H
O
S39
(100 MHz, CD3OD)
abundance
0.3
0.2
0.1
0
190.0
180.0
170.0
160.0
150.0
140.0
130.0
120.0
110.0
100.0
90.0
80.0
70.0
60.0
50.0
40.0
30.0
20.0
10.0
0
174.0999
170.9097
157.2290
145.3589
122.9122
80.5384
57.5552
54.9494
49.6419
49.4311
49.2203
49.0000
48.7892
48.5689
48.3581
41.0962
32.7709
32.6464
29.5136
28.6993
24.9246
21.1021
19.7034
19.4543
X : parts per Million : 13C

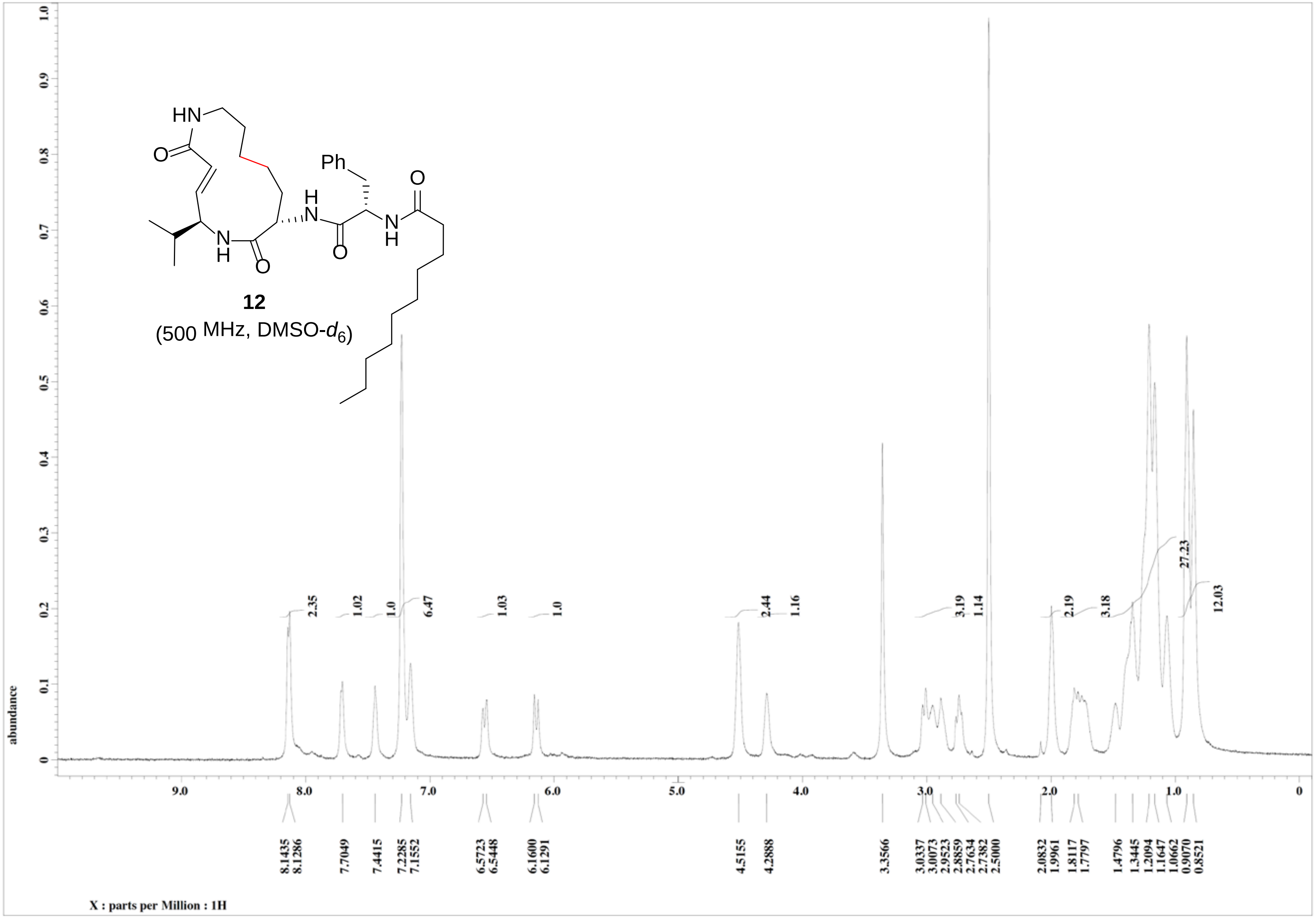
HN
O
Ph
O
H
N
N
H
N
H
O
O
12
(500 MHz, DMSO-d6)
abundance
X : parts per Million : 1H
2.35
1.02
1.0
6.47
1.03
1.0
2.44
1.16
3.19
1.14
2.19
3.18
27.23
12.03
8.1435
8.1286
7.7049
7.4415
7.2285
7.1552
6.5723
6.5448
6.1600
6.1291
4.5155
4.2888
3.3566
3.0337
3.0073
2.9523
2.8859
2.7634
2.7382
2.5000
2.0832
1.9961
1.8117
1.7797
1.4796
1.3445
1.2094
1.1647
1.0662
0.9070
0.8521

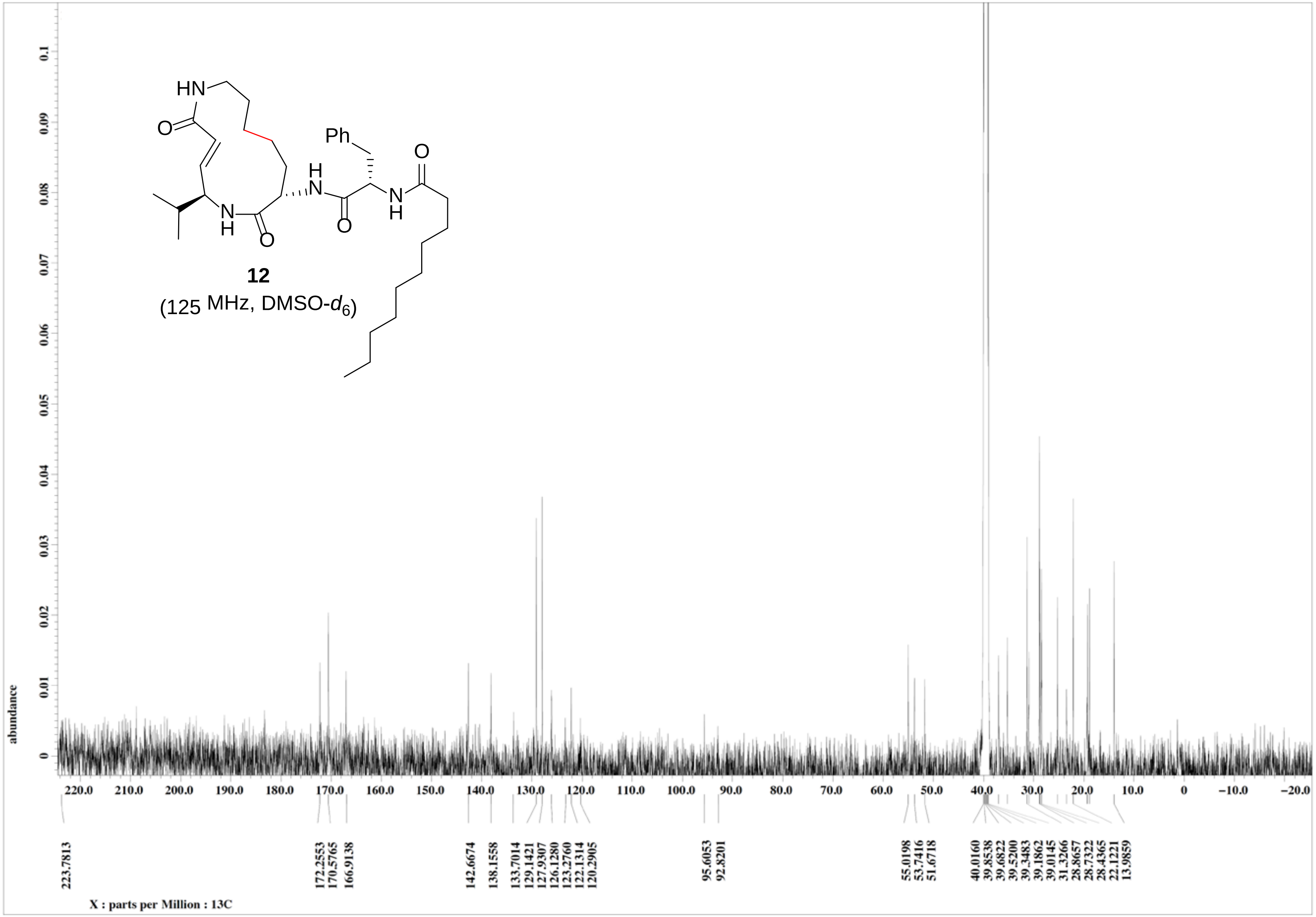

HN
O
Ph
O
H
N
N
H
N
H
O
O
12
(125 MHz, DMSO-d_6)
abundance
X : parts per Million : 13C
223.7813
172.2553
170.5765
166.9138
142.6674
138.1558
133.7014
129.1421
127.9307
126.1280
123.2760
122.1314
120.2905
95.6053
92.8201
55.0198
53.7416
51.6718
40.0160
39.8538
39.6822
39.5200
39.3483
39.1862
39.0145
31.3266
28.8657
28.7322
28.4365
22.1221
13.9859

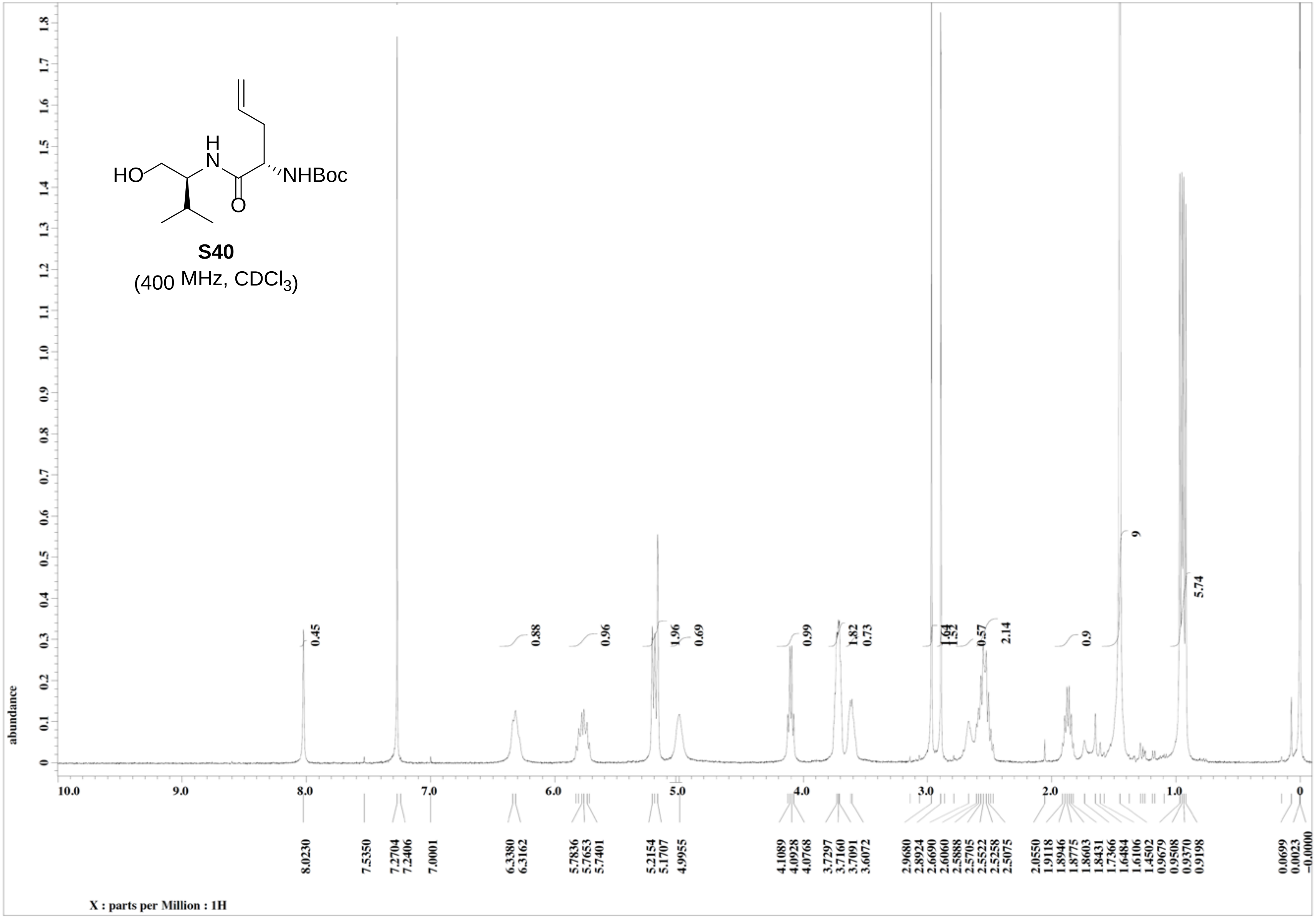

HO
H
N
NHBoc
O
S40
(400 MHz, CDCl3)
abundance
X : parts per Million : 1H
0.45
0.88
0.96
1.96
0.69
0.99
1.82
0.73
0.57
2.14
0.9
9
5.74
8.0230
7.5350
7.2704
7.2406
7.0001
6.3380
6.3162
5.7836
5.7653
5.7401
5.2154
5.1707
4.9955
4.1089
4.0928
4.0768
3.7297
3.7160
3.7091
3.6072
2.9680
2.8924
2.6690
2.6060
2.5888
2.5705
2.5522
2.5258
2.5075
2.0550
1.9118
1.8946
1.8775
1.8603
1.8431
1.7366
1.6484
1.6106
1.4502
0.9679
0.9508
0.9370
0.9198
0.0699
0.0023
-0.0000

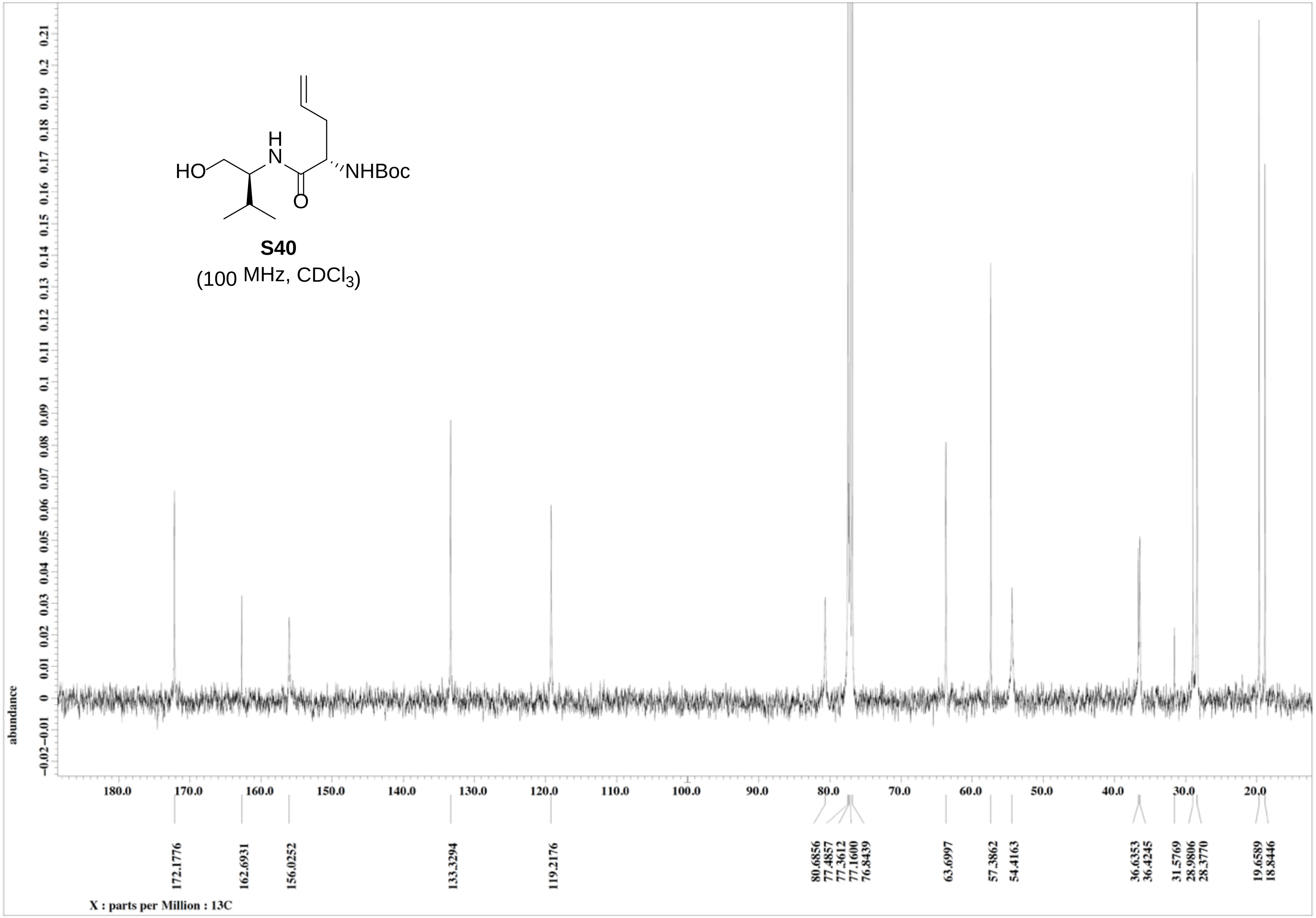

HO
H
N
NHBoc
O
S40
(100 MHz, CDCl3)
abundance
X : parts per Million : 13C
172.1776
162.6931
156.0252
133.3294
119.2176
80.6856
77.4857
77.3612
77.1600
76.8439
63.6997
57.3862
54.4163
36.6353
36.4245
31.5769
28.9806
28.3770
19.6589
18.8446

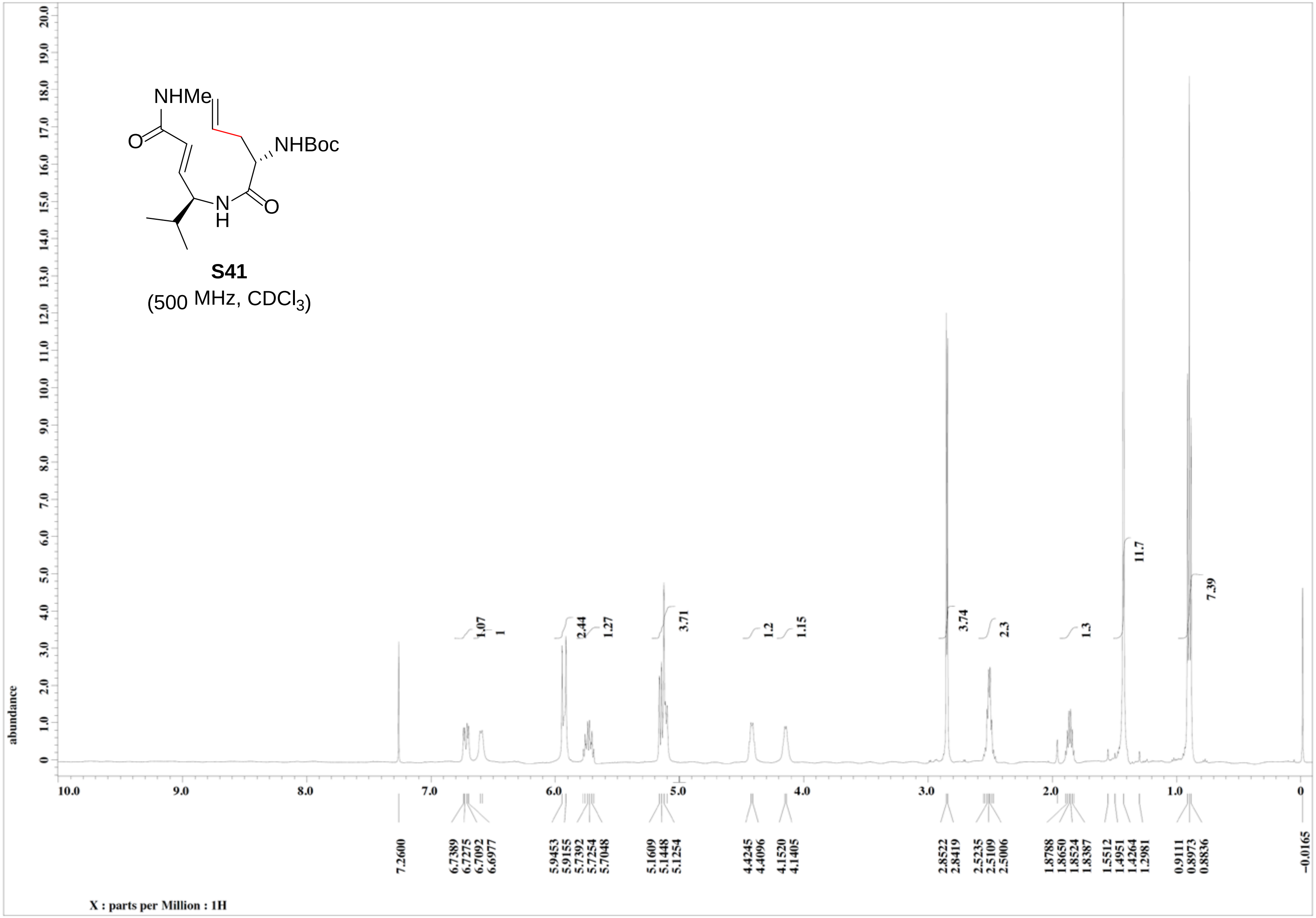
NHMe
O
NHBoc
N
H
O
S41
(500 MHz, CDCl3)
abundance
X : parts per Million : 1H

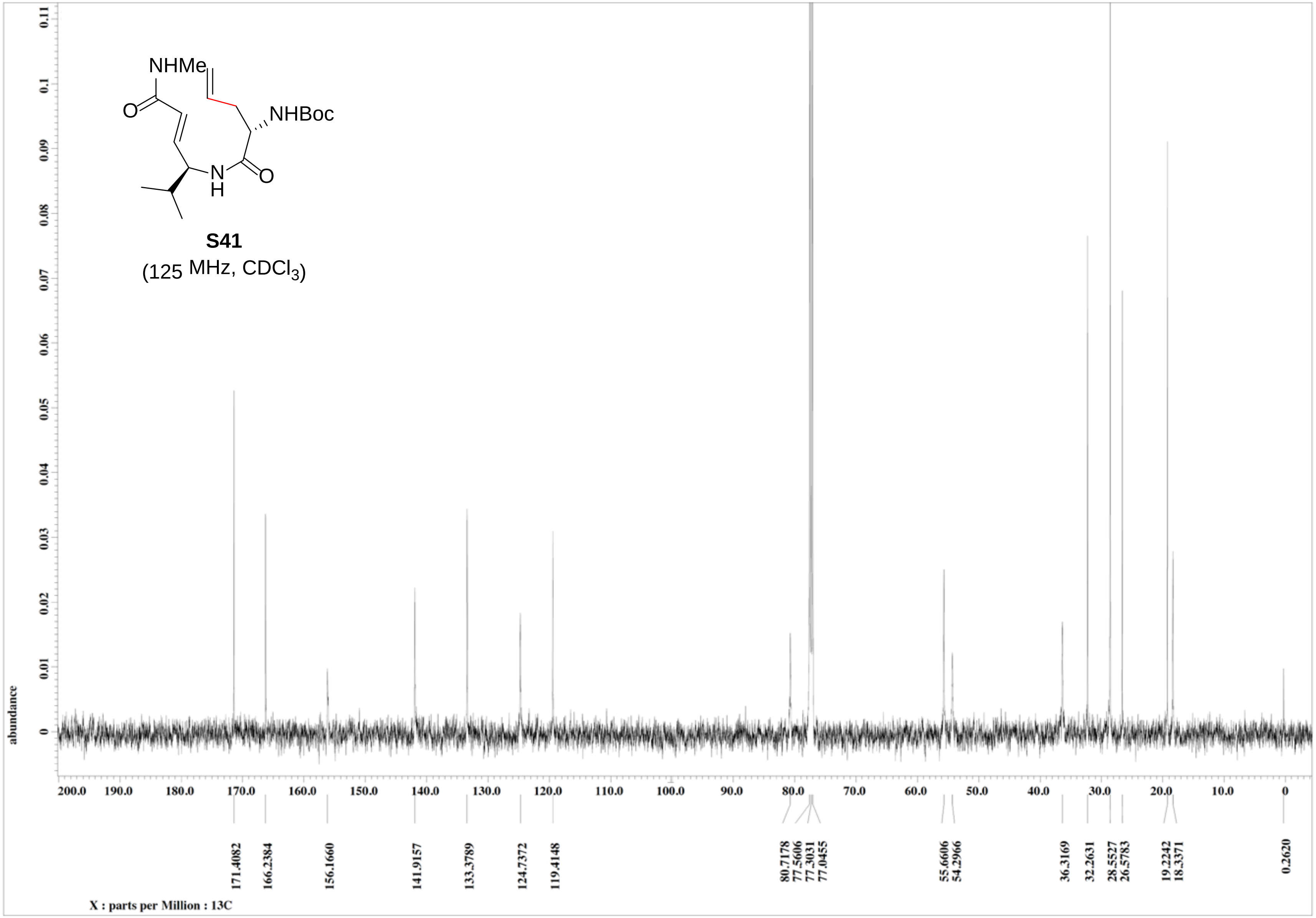
NHMe
O
NHBoc
N
H
O
S41
(125 MHz, CDCl3)
abundance
X : parts per Million : 13C
171.4082
166.2384
156.1660
141.9157
133.3789
124.7372
119.4148
80.7178
77.5606
77.3031
77.0455
55.6606
54.2966
36.3169
32.2631
28.5527
26.5783
19.2242
18.3371
0.2620

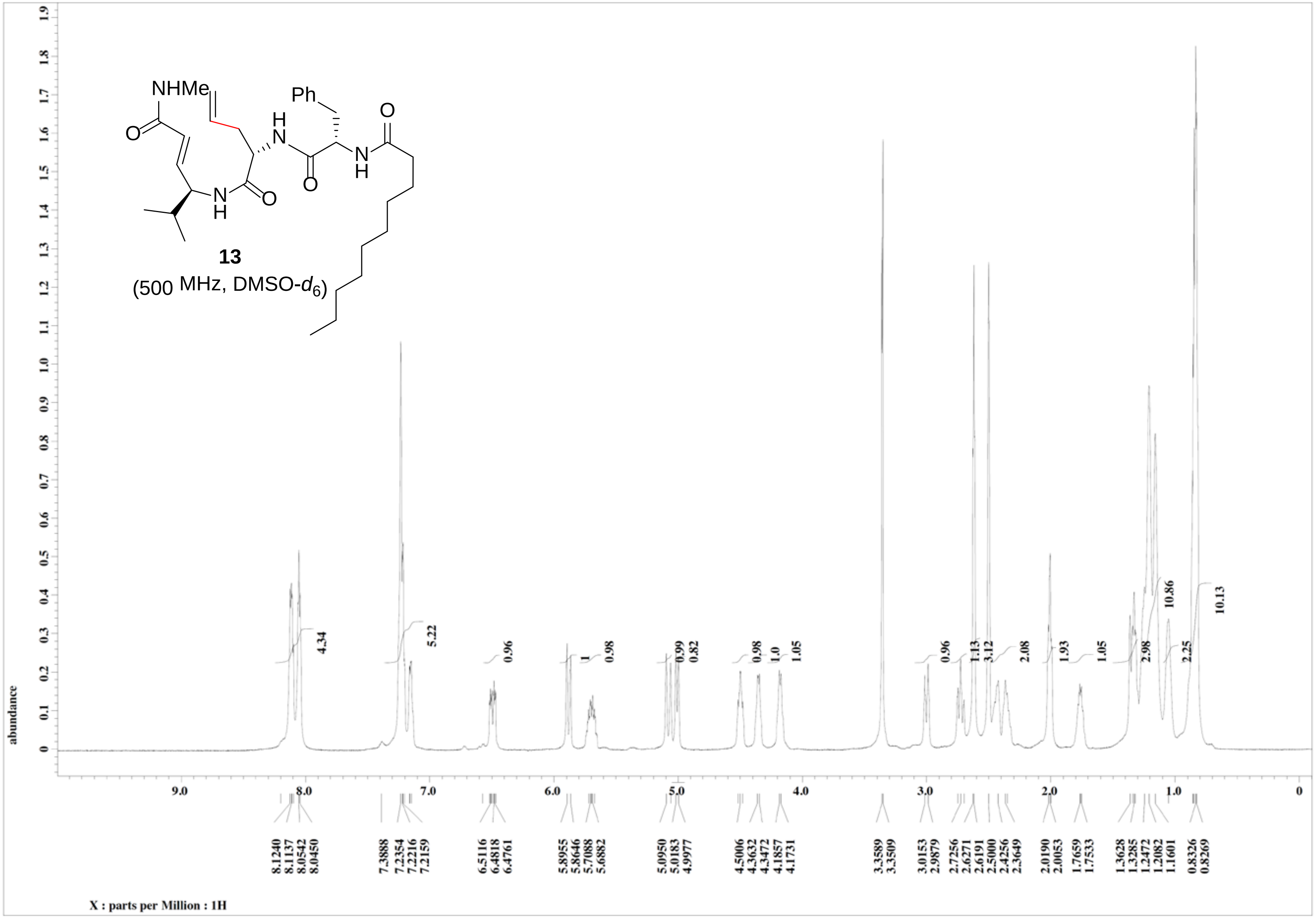
NHMe
Ph
O
H
N
O
N
H
N
H
O
O
13
(500 MHz, DMSO-d6)
abundance
X : parts per Million : 1H

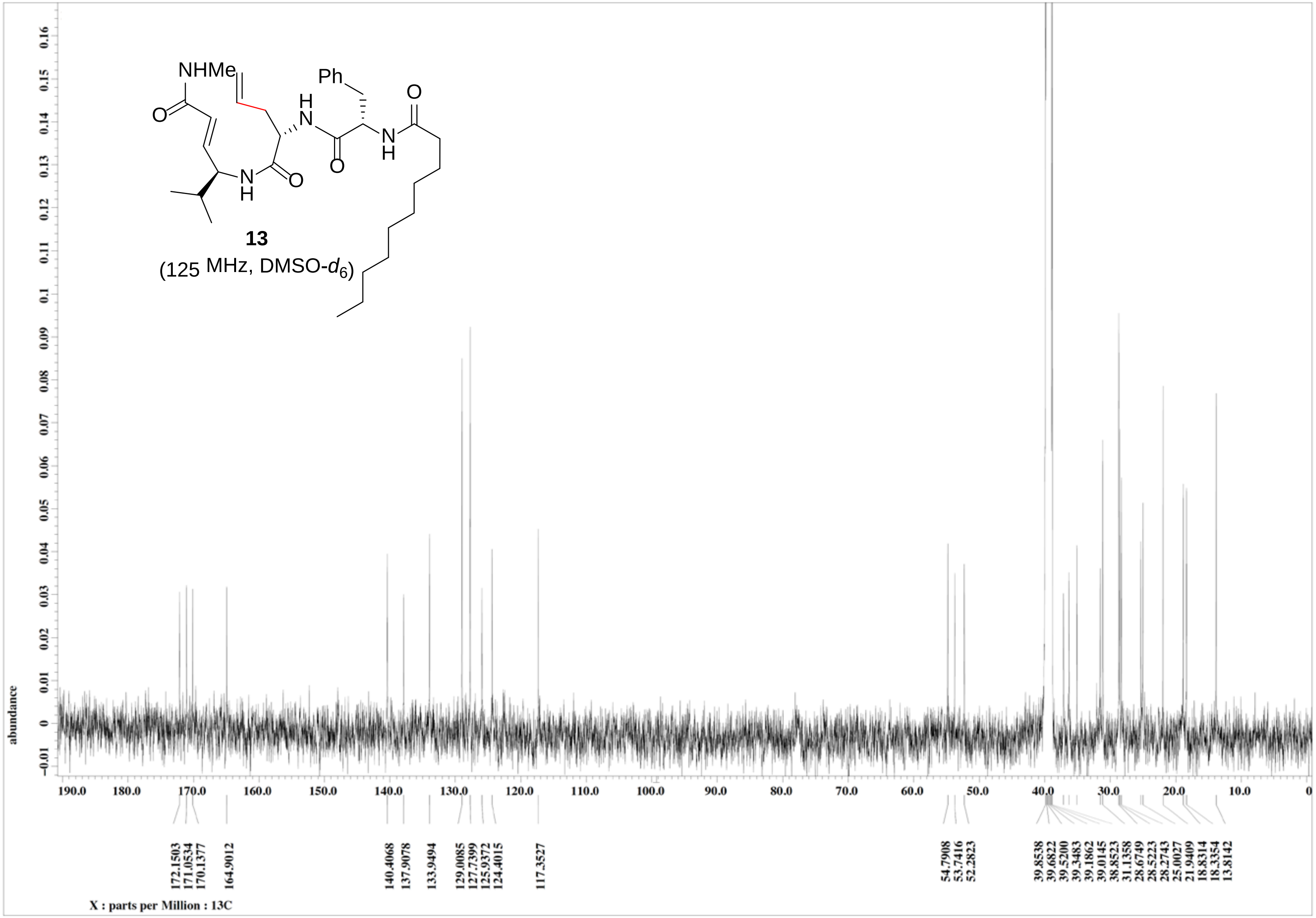

NHMe
Ph
O
H
N
N
H
O
O
N
H
O
13
(125 MHz, DMSO-d6)
abundance
X : parts per Million : 13C
172.1503
171.0534
170.1377
164.9012
140.4068
137.9078
133.9494
129.0085
127.7399
125.9372
124.4015
117.3527
54.7908
53.7416
52.2823
39.8538
39.6822
39.5200
39.3483
39.1862
39.0145
38.8523
31.1358
28.6749
28.5223
28.2743
25.0027
21.9409
18.8314
18.3354
13.8142

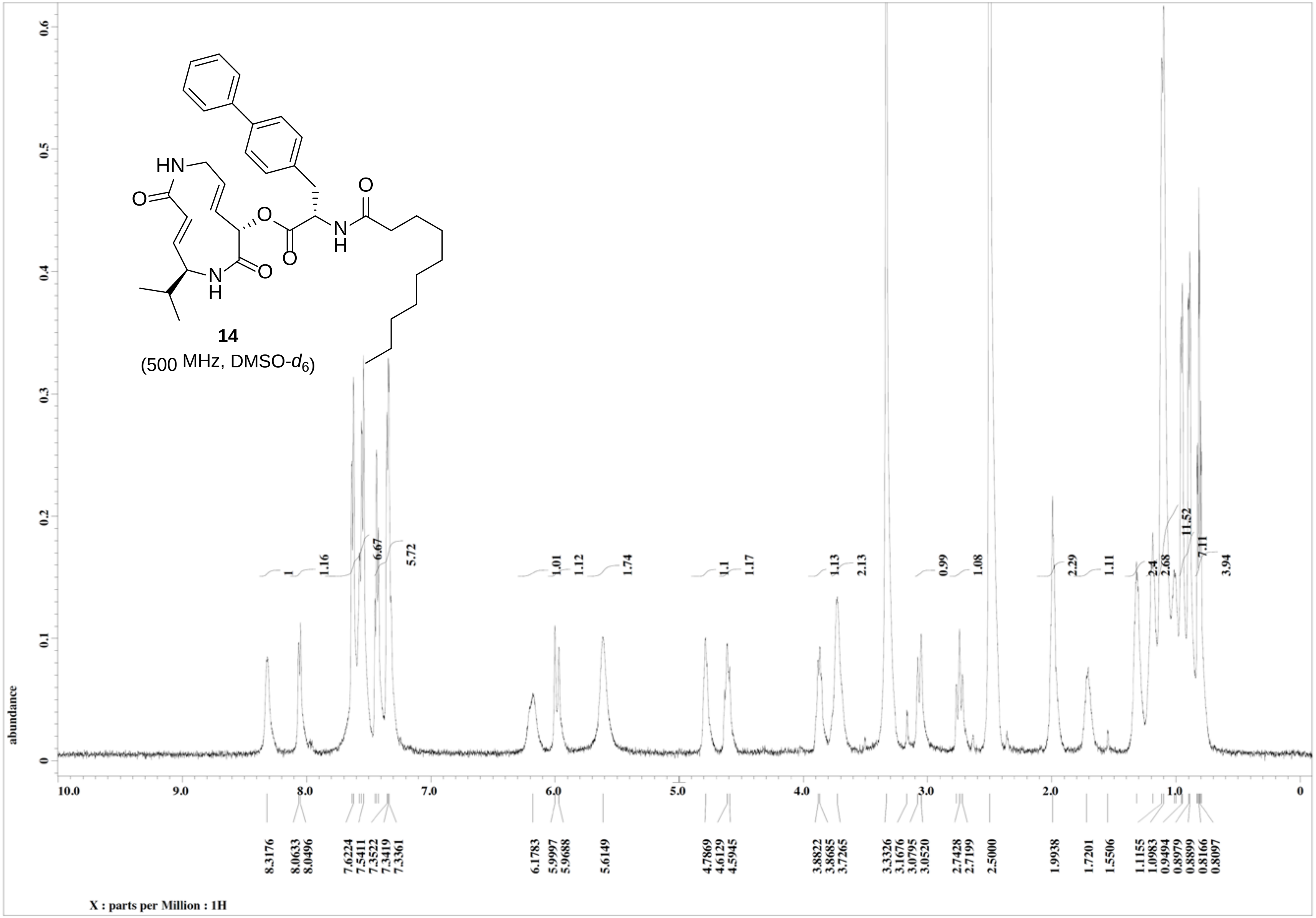
HN
O
O
O
N
H
N
H
O
O
14
(500 MHz, DMSO-d_6)
abundance
X : parts per Million : 1H
1
1.16
6.67
5.72
1.01
1.12
1.74
1.1
1.17
1.13
2.13
0.99
1.08
2.29
1.11
2.4
2.68
11.52
7.11
3.94
8.3176
8.0633
8.0496
7.6224
7.5411
7.3522
7.3419
7.3361
6.1783
5.9997
5.9688
5.6149
4.7869
4.6129
4.5945
3.8822
3.8685
3.7265
3.3326
3.1676
3.0795
3.0520
2.7428
2.7199
2.5000
1.9938
1.7201
1.5506
1.1155
1.0983
0.9494
0.8979
0.8899
0.8166
0.8097

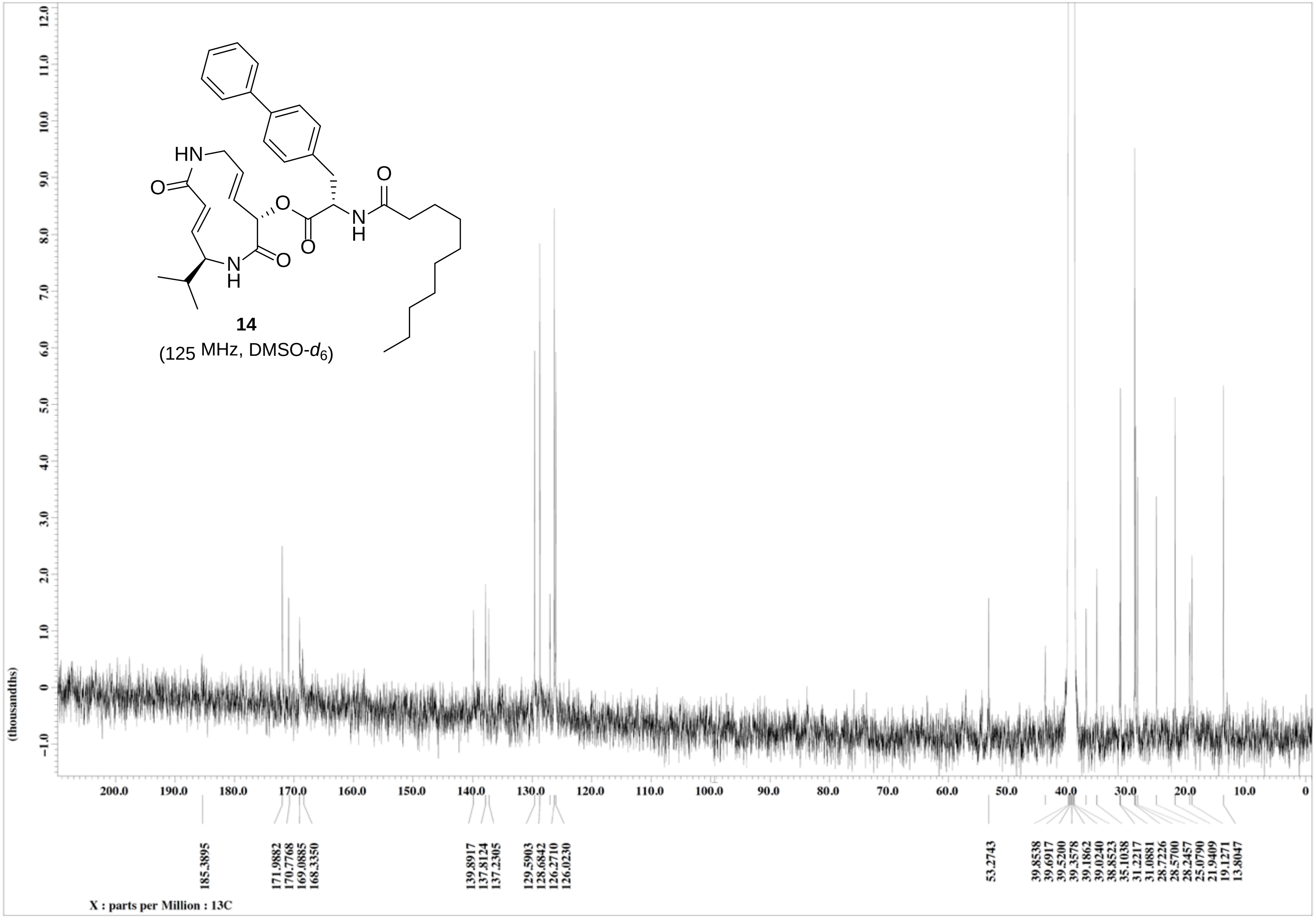
14
(125 MHz, DMSO-d_6)
(thousandths)
X : parts per Million : 13C
185.3895
171.9882
170.7768
169.0885
168.3350
139.8917
137.8124
137.2305
129.5903
128.6842
126.2710
126.0230
53.2743
39.8538
39.6917
39.5200
39.3578
39.1862
39.0240
38.8523
35.1038
31.2217
31.0881
28.7226
28.5700
28.2457
25.0790
21.9409
19.1271
13.8047

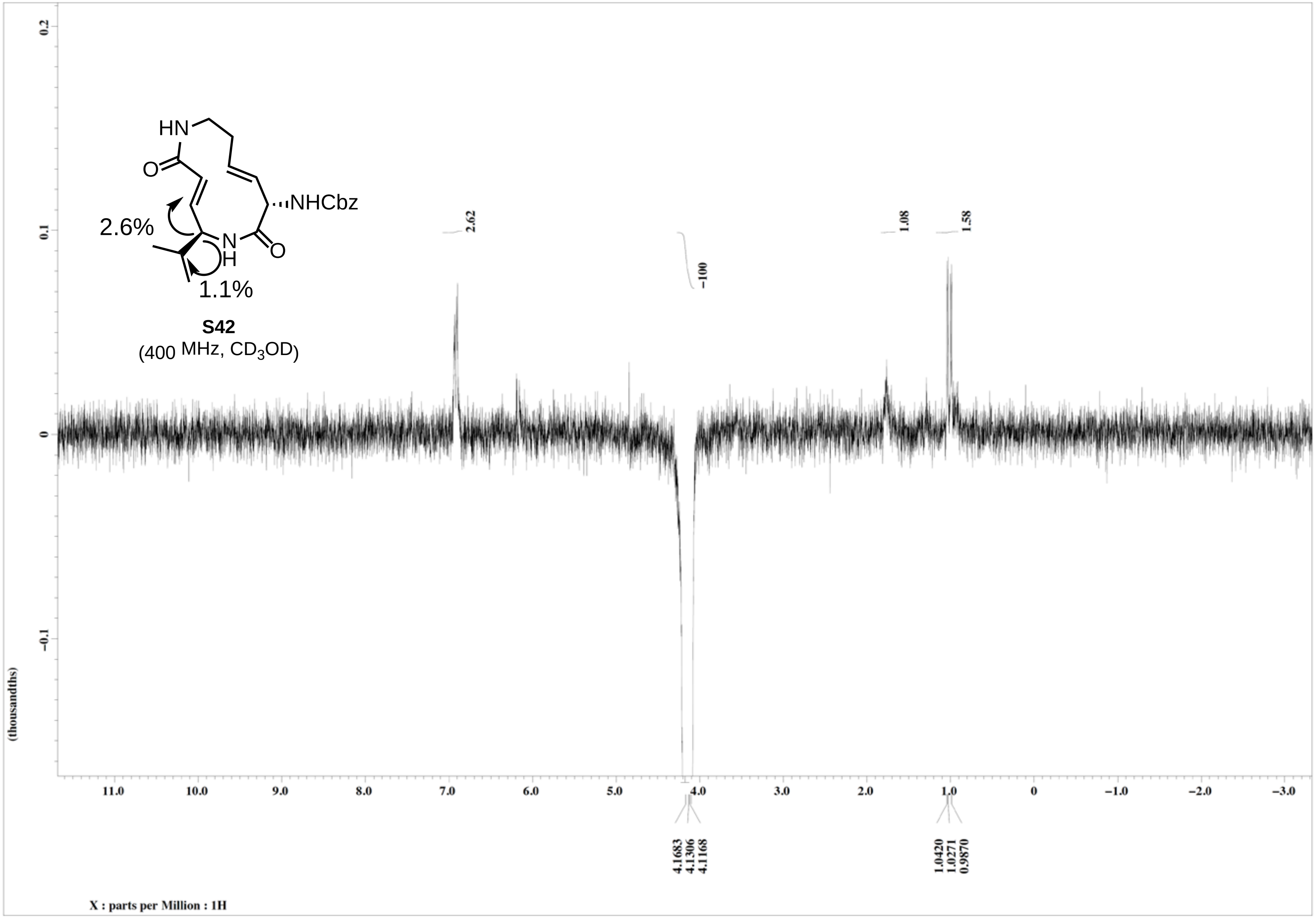
HN
O
NHCbz
2.6%
N
H
O
1.1%
S42
(400 MHz, CD3OD)
2.62
−100
1.08
1.58
0.2
0.1
0
−0.1
(thousandths)
11.0
10.0
9.0
8.0
7.0
6.0
5.0
4.0
3.0
2.0
1.0
0
−1.0
−2.0
−3.0
4.1683
4.1306
4.1168
1.0420
1.0271
0.9870
X : parts per Million : 1H

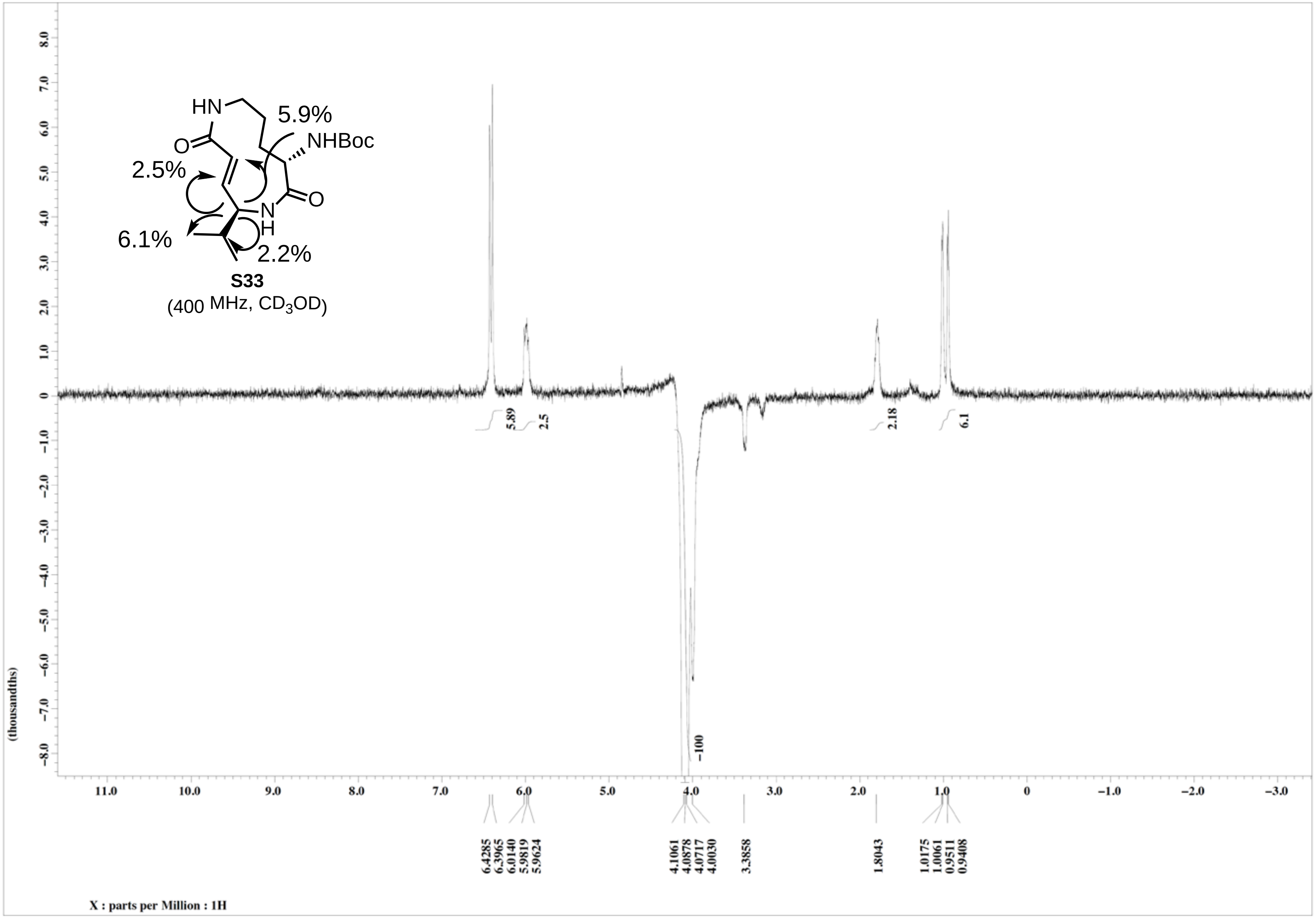

HN
5.9%
NHBoc
O
2.5%
N
H
O
6.1%
2.2%
S33
(400 MHz, CD3OD)
5.89
2.5
−100
2.18
6.1
6.4285
6.3965
6.0140
5.9819
5.9624
4.1061
4.0878
4.0717
4.0030
3.3858
1.8043
1.0175
1.0061
0.9511
0.9408
(thousandths)
X : parts per Million : 1H
